# Global influenza seasonality to inform country-level vaccine programs: An analysis of WHO FluNet influenza surveillance data between 2011 and 2016

**DOI:** 10.1371/journal.pone.0193263

**Published:** 2018-02-21

**Authors:** Laura P. Newman, Niranjan Bhat, Jessica A. Fleming, Kathleen M. Neuzil

**Affiliations:** 1 Department of Global Health, University of Washington, Seattle, Washington, United States of America; 2 Center for Vaccine Innovation and Access, PATH, Seattle, Washington, United States of America; 3 Center for Vaccine Development, University of Maryland School of Medicine, Baltimore, Maryland, United States of America; University of Hong Kong, HONG KONG

## Abstract

By analyzing publicly available surveillance data from 2011–2016, we produced country-specific estimates of seasonal influenza activity for 118 countries in the six World Health Organization regions. Overall, the average country influenza activity period was 4.7 months. Our analysis characterized 100 countries (85%) with one influenza peak season, 13 (11%) with two influenza peak seasons, and five (4%) with year-round influenza activity. Surveillance data were limited for many countries. These data provide national estimates of influenza activity, which may guide planning for influenza vaccination implementation, program timing and duration, and policy development.

## Introduction

Seasonal influenza is a significant public health problem, and vaccination is the primary tool to reduce influenza morbidity and mortality[1,2]. The World Health Organization (WHO) recommends influenza vaccination of high risk groups, including pregnant women, children aged <5 years, adults aged ≥ 65 years, persons of all ages with chronic medical conditions, and health care workers[3,4]. In 2013, 40% of countries worldwide recommend influenza vaccination in their National Immunization Programs, although influenza vaccine dose distribution varies[5–7]. Between 2004 and 2013, influenza dose distribution rates were the highest in the Region of the Americas and European Region, and lowest in South-East Asia and African Regions[7].

While all vaccination programs have logistical challenges, influenza immunization programs must take into account disease seasonality[8–10], and the continuous evolution of influenza virus strains, necessitating biannual assessments of the need for vaccine formulation changes[11]. Defining the time period with the greatest influenza virus activity in a country can contribute to determination of vaccination strategy. Influenza seasonality is most thoroughly characterized in high-income countries located in temperate regions, where influenza activity typically peaks during winter months. Influenza activity and epidemics generally occur between November and March in the temperate northern hemisphere, and between April and September in the temperate southern hemisphere [12]. While recent efforts by WHO and the US Centers for Disease Control and Prevention (CDC) to scale up influenza surveillance provide broader, more comprehensive data in several parts of the world, influenza activity in low- and low-to-middle-income countries, particularly in tropical and subtropical regions, remains relatively unexamined [3,13]. Country-specific influenza surveillance data are essential in guiding decision making about national influenza vaccine policy and program implementation, including timing of vaccine administration [14].

The objective of this work was to utilize publicly available influenza surveillance data collected over multiple years to define typical periods of influenza activity in countries reporting to WHO FluNet. We presented a similar analysis using data from 2010 through 2014 at an April 2015 WHO expert working group meeting on seasonal influenza vaccine composition and vaccination timing for the tropics and subtropics[15]. Our country-specific results informed a collaborative recommendation of influenza vaccination timing for groupings of countries in vaccination zones[16]. The following analysis includes more recent influenza seasons and provides additional detailed and graphical representation of country-specific influenza activity. In addition, we exclude calendar year 2010, which was atypical in many countries due to the 2009–2010 influenza pandemic and may have skewed the prior findings. These country-specific influenza seasonality estimates may be useful to inform vaccination program implementation, timing, and duration.

## Materials and methods

### Data source

Influenza surveillance data were compiled from FluNet, a publicly available, online database of the WHO Global Influenza Surveillance Network for laboratory-confirmed influenza confirmed samples[17]. FluNet is a global data collection and reporting tool for influenza virological surveillance. It reports weekly influenza surveillance data that are provided from over 140 National Influenza Centres of the Global Influenza Surveillance and Response System (GISRS), national influenza reference laboratories, and WHO regional databases[18]. Per the WHO Global Epidemiological Surveillance Standards for Influenza guidance, influenza testing is conducted on specimens collected from persons presenting for medical care at participating surveillance sites who meet a clinical definition for influenza-like illness (defined as an acute respiratory infection with measured fever of ≥ 38^°^C, and cough, with onset within the last 10 days), or severe acute respiratory infection (defined as an acute respiratory infection with history of fever or measured fever of ≥ 38^°^C, and cough, with onset within the last 10 days, and requiring hospitalization)[18]. Influenza is confirmed by accepted laboratory diagnostic methods and actively reported by the reference laboratory to FluNet[19].

### Data analysis

Surveillance data are reported by countries into FluNet on a weekly basis, though some countries only conduct surveillance during weeks with high influenza activity. To identify months with the largest relative frequency of influenza cases in comparison with other times throughout the year, weekly data were used to calculate a monthly case proportion, defined as the number of influenza test-positive cases reported in a given month as a percentage of the total number of influenza cases reported in the calendar year per country. Raw data of country-reported laboratory test-positive influenza cases were summarized into weekly totals, beginning on January 1 of each year, and converted to monthly data by determining the number of weeks in each month and summing the number of weekly influenza cases reported during that time. For example, the 31 days in January translates into 4.4 weeks, and the sum of influenza cases reported from weeks 1–4.4 represents the total number of influenza confirmed samples reported in January for a given year. No differentiation was made between weeks with no reported influenza data and weeks with zero influenza cases reported. A month was characterized as having influenza activity if the cases represented greater than or equal to a fixed threshold [20] of 10% of total reported influenza positive cases in the year in two or more years from 2011 through 2016 (January-December). To improve data quality, the year was excluded in any given country if fewer than 50 influenza cases were reported that year.

Countries were categorized according to influenza seasonal patterns: 1) one influenza peak, defined as one to seven months of continuous influenza activity; 2) two influenza peaks, defined as two sets of one or more months of influenza activity separated by at least two months of non-influenza activity; or, 3) year-round influenza activity, defined as 8 or more months of influenza activity at any time during the year, or three or more influenza peaks, each separated by two months of non-influenza activity. Data analysis was performed in Microsoft Excel (2010).

### Data visualization

Twelve global maps of influenza activity were created to depict monthly patterns of influenza activity. The maps were created using the free desktop version of StatPlanet Plus [StatSilk (2015). StatPlanet: Interactive Data Visualization and Mapping Software. http://www.statsilk.com].

## Results

One hundred thirty two countries reported influenza surveillance data to FluNet between 2011 and 2016. Overall, 118 (89%) of the 132 countries reported more than 50 cases per year for two or more years between 2011 and 2016 and were included in the analyses. In 2011, 109 (83%) of the 132 countries reported more than 50 cases; this fluctuated between 110 (83%) in 2012, 114 (86%) in 2013, 109 (83%) in 2014, 105 (80%) in 2015, and 119 (90%) in 2016. Some countries did not report influenza surveillance data to WHO FluNet in every year. The greatest increase in the number of countries reporting at least 50 positive influenza cases to WHO FluNet occurred in the African Region, where influenza surveillance expanded from 14 countries in 2010 to 22 countries in 2016[17,21].

**Fig 1:**
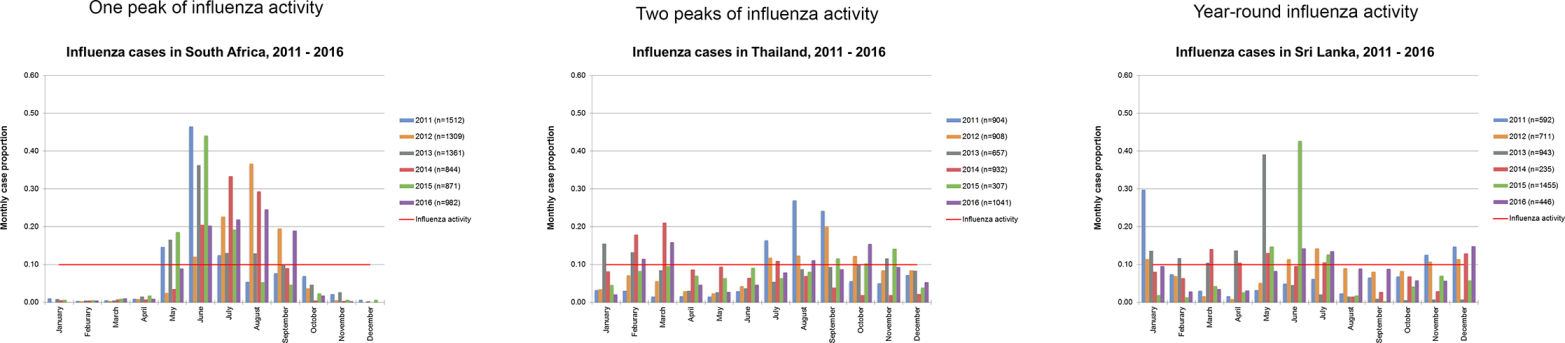
Illustrative examples of influenza seasonality classifications, 2011–2016.

Bar charts of the influenza monthly case proportion by country are listed alphabetically in S1 Appendix. These charts were used to characterize influenza activity patterns by country into one peak, two peaks, or year-round influenza activity. Of the 118 countries, 100 (85%) had one influenza peak, 13 (11%) had two influenza peaks, and five (4%) had year-round influenza activity (Table 1). Examples of countries within each category of influenza circulation are depicted in Fig 1: South Africa had one influenza peak between May and September, Thailand had two influenza peaks in February through March and July through November, and Sri Lanka had year-round influenza activity. Among countries with one or two influenza peaks, the average yearly influenza activity was 4.5 and 5.3 months long, respectively. Country-level information about the specific months with influenza activity, in addition to the number of months and influenza pattern for each of the 118 countries, is listed in S1 Table.

**Table 1:** Influenza activity^1^ seasonality by World Health Organization region, 2011–2016.

**African Region**		
Influenza activity pattern^2^	Number of countries (%)	Countries
One peak	13 (59)	Algeria, Cameroon, Central African Republic, Côte d'Ivoire, Democratic Republic of the Congo, Ethiopia, Madagascar, Mauritius, Niger, Rwanda, Sierra Leone, South Africa, Uganda
Two peaks	5 (23)	Mali, Senegal, Togo, United Republic of Tanzania, Zambia
Year-round activity	4 (18)	Burkina Faso, Ghana, Kenya, Nigeria
**Eastern Mediterranean Region**		
Influenza activity pattern	Number of countries (%)	Countries
One peak	10 (0)	Bahrain, Egypt, Iraq, Islamic Republic of Iran, Jordan, Morocco, Oman, Pakistan, Qatar, Tunisia
Two peaks	0 (0)	
Year-round activity	0 (0)	
**European Region**		
Influenza activity pattern	Number of countries (%)	Countries
One peak	42 (100)	Albania, Austria, Belarus, Belgium, Bosnia and Herzegovina, Bulgaria, Croatia, Czech Republic, Denmark, Estonia, Finland, France, Georgia, Germany, Greece, Hungary, Iceland, Ireland, Israel, Italy, Kazakhstan, Kyrgyzstan, Latvia, Lithuania, Malta, Netherlands, Norway, Poland, Portugal, Republic of Moldova, Romania, Russian Federation, Serbia, Slovakia, Slovenia, Spain, Sweden, Switzerland, Turkey, Ukraine, United Kingdom of Great Britain and Northern Ireland, Uzbekistan
Two peaks	0 (0)	
Year-round activity	0 (0)	
**Region of the Americas**		
Influenza activity pattern	Number of countries (%)	Countries
One peak	18 (86)	Argentina, Bolivia, Brazil, Canada, Chile, Columbia, Costa Rica, Cuba, Dominican Republic, Ecuador, Guatemala, Honduras, Mexico, Nicaragua, Paraguay, Peru, United States of America, Uruguay
Two peaks	3 (14)	El Salvador, Jamaica, Panama
Year-round activity	0 (0)	
**South-East Asia Region**		
Influenza activity pattern	Number of countries (%)	Countries
One peak	4 (57)	Bangladesh, Bhutan, India, Indonesia
Two peaks	2 (29)	Nepal, Thailand
Year-round activity	1 (14)	Sri Lanka
**Western Pacific Region**		
Influenza activity pattern	Number of countries (%)	Countries
One peak	10 (83)	Australia, Cambodia, China, Japan, Republic of Korea, Lao People's Democratic Republic, Malaysia, Mongolia, New Zealand, Viet Nam
Two peaks	2 (17)	Philippines, Singapore
Year-round activity	0 (0)	
**Non-member states**		
Influenza activity pattern	Number of countries (%)	Countries
One peak	3 (75)	French Guiana, Guadeloupe, Martinique
Two peaks	1 (25)	New Caledonia
Year-round activity	0 (0)	

^1^A month was considered to have influenza activity if it had ≥10% total reported yearly cases of influenza for two or more years between 2011 and 2016. Countries that reported <50 influenza cases in a year were excluded for that year. No differentiation was made between countries that reported zero influenza cases and reported no data.

^2^1 peak: One to seven consecutive months of influenza activity; 2 peaks: Two sets of influenza activity separated by ≥2 months of non-activity; year-round activity: Eight or more months of flu activity, or 3+ sets of influenza activity each separated by ≥2 months

### African Region

In the African Region (AFR), 22 (47%) of 47 countries reported influenza surveillance data to WHO FluNet between 2011 and 2016. The average influenza peak was 5.7 months long. This region was the only region with countries characterized as having all three types of influenza patterns: year-round influenza activity in four countries (18%), one influenza peak in 13 countries (59%), and two influenza peaks in five countries (23%). Influenza activity in the northern AFR countries was generally similar to temperate northern hemisphere patterns, while activity in the southern AFR countries exhibited influenza patterns more indicative of southern hemisphere activity. Influenza activity varied in tropical countries. Our analysis demonstrated variability in peak frequency in western Africa. For example, Senegal had one influenza peak between August and November; Mali had two influenza peaks in February through April and in September through October; and Nigeria had year-round influenza activity. In eastern Africa, Kenya had year round influenza activity, and Tanzania had two influenza peaks in November through January and April through June. Influenza seasonality was well defined in South Africa, which reported activity from May through September, similar to other temperate southern hemisphere countries.

### Eastern Mediterranean Region

In the 10 (48%) of 21 countries that reported influenza surveillance data from Eastern Mediterranean Region (EMR), influenza activity lasted an average of 4.8 months. Bahrain, Egypt, Iraq, Islamic Republic of Iran, Jordan, Morocco, Oman, Pakistan, Qatar, and Tunisia were characterized as having one influenza peak, typically from November or December through February or March.

### European Region

Seventy-nine percent of European Region (EUR) countries (42/53) reported to FluNet between 2011 and 2016. All 42 (100%) countries were characterized as having one influenza peak, which lasted an average of 3.8 months in duration. Influenza activity in this region typically was observed in December or January through March or April.

### Region of the Americas

Twenty-one (60%) of 35 countries in the Region of the Americas (AMR), which spans both the northern and southern hemisphere, reported influenza surveillance data to WHO FluNet. These countries had influenza activity averaging 4.7 months, and 18 (86%) countries had one influenza peak. Canada, the United States, and Mexico largely follow a one-season northern hemisphere influenza activity pattern, with primary transmission months between December and March or April. Three countries (14%) had two influenza peaks: El Salvador had influenza peaks in May through July and again in December; Jamaica had influenza peaks in February and October through November; and Panama had influenza peaks in May through August and in November.

### South-East Asia Region

Seven (64%) of 11 countries from the South-East Asia Region (SEAR) reported influenza surveillance data between 2011 and 2016. Influenza activity lasted an average of 6.1 months. Influenza activity did not follow a pattern in this geographically dispersed region, as single influenza peaks did not have consistent yearly timing observed in four of 11 (36%) countries: Bangladesh, Bhutan, India, Indonesia. Two countries (18%) had two influenza peaks: Nepal influenza peaks were defined in March through April and July through August; and Thailand’s influenza peaks were defined in February through March and July through November. One country (9%), Sri Lanka, had year-round influenza activity.

### Western Pacific Region

Twelve (32%) of 37 countries and areas in the Western Pacific Region (WPR) reported surveillance data between 2011 and 2016. Average influenza peaks lasted 5.2 months; 10 (83%) countries had one influenza peak and two (17%) had two influenza peaks. One peak of influenza activity was characterized in Australia and New Zealand during typical southern hemisphere timing between July and October. China, Japan, Korea, and Mongolia reported one peak of influenza activity typical of northern hemisphere countries in December or January through March or April. One peak of influenza activity was characterized in Lao People’s Democratic Republic between September and February and in Cambodia between June and December. Two peaks of influenza activity were characterized in Singapore in December through February and June through August.

### Global influenza activity

**Fig 2:**
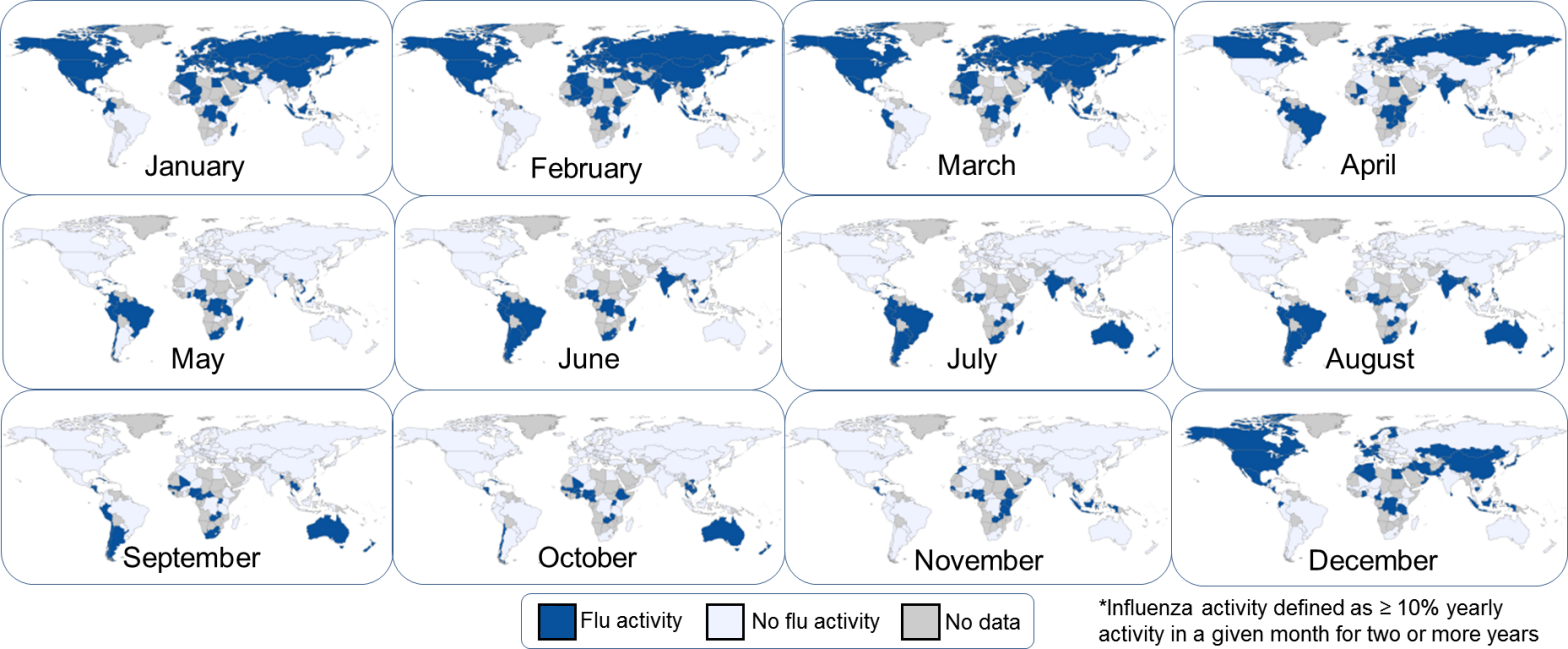
Global maps of monthly influenza activity, 2011–2016.

Seasonal influenza activity began in the northern hemisphere during November and moved progressively to the southern hemisphere by May. In June through October, influenza activity was concentrated in the southern hemisphere. Influenza activity was reported from countries near the equator throughout the year in various patterns. (Fig 2)

## Discussion

In this report, using predefined inclusion criteria, we present an analysis of WHO FluNet influenza surveillance data from 118 countries between 2011 and 2016 to characterize seasonal patterns of influenza activity. Our methods can be easily adapted for individual country needs.

Many countries in temperate climates have robust influenza surveillance, and as such, have well-characterized seasonal influenza activity. Our analyses support previously cited literature from European countries that the influenza season in these areas generally occurs between December and March, during the cool winter season[11,22], although there is evidence for variability across the continent[23,24]. Similarly, influenza activity across the Americas is well characterized, as the United States, Canada and the member countries of the Pan American Health Organization have established high quality surveillance systems.

In equatorial countries, large data gaps remain regarding seasonal influenza activity. Identification of clear influenza trends is challenging due to a paucity of data and inconsistency of reported influenza circulation, particularly in equatorial Africa. While influenza surveillance on the continent has improved over recent years, fewer than half of African countries have influenza surveillance laboratories reporting to WHO FluNet[17]. Many of the discrepancies between published literature and our analysis using the WHO FluNet database in equatorial Africa can be attributed to the limited amount of data available, either in annual influenza cases reported, or the number of years of collected data. For example, a study using sentinel surveillance data from five public hospitals in Rwanda between 2008 and 2010 determined that the percentage of positive influenza samples was highest in October through November and February through March[25]. Our analysis showed influenza activity in February through June during the period from 2011–2016. In the sentinel surveillance study, fewer than 400 influenza cases were reported over two years, and reported cases to WHO FluNet varied between 31 and 151 per year between 2011 and 2016. Though there are published studies of influenza activity from Tanzania[26], Uganda[27], Nigeria[28], and Madagascar[29–31], they all utilized influenza data from or before 2010, which may reflect atypical seasonality due to the 2009 H1N1 pandemic. Kenya is one equatorial African country with multiple recent influenza trend analyses. Three studies describing results between 2007 and 2013 corroborate our findings of year-round circulation[32–34]. As influenza surveillance is strengthened, it will be important to periodically review collected data to update and improve estimates of influenza seasonality in equatorial countries.

The main implications of our analysis relate to timing of vaccination and availability of vaccines. The current strain selection and vaccine distribution mechanisms were designed for countries with typical Northern Hemisphere and Southern Hemisphere single-peak influenza seasons. For other countries, influenza vaccination may need to occur year-round, or at times when vaccine is not readily available. This is especially challenging in tropical and subtropical countries, where influenza seasonality is less well defined. While a recent manuscript explored geographical groupings of tropical countries into vaccination zones to guide influenza timing[16], our analysis provides descriptive national level data that can be utilized to guide individual country influenza vaccination program planning. Additionally, the methods used in our analysis can be easily replicated without advanced statistical analysis. Efforts are underway by the WHO and other partners to understand how to best implement influenza vaccination programs in countries with atypical influenza seasons[35][36].

Strengths of this study include the analysis of data across multiple years to help define patterns of influenza seasonality and exclusion of data from years where influenza reporting was very low in an effort to determine influenza activity based on sufficient reporting. To provide assurance that results would not be biased by a single anomalous month of sporadic influenza activity, specific months were only considered part of the season if influenza activity was present during two or more years. Our analysis incorporates data starting in 2011 in order to limit the influence of residual influenza activity from the influenza pandemic of 2009–2010. Additionally, the classification of influenza activity was based on a relative frequency of confirmed influenza cases, and was not dependent on the overall number of influenza cases tested, which may be influenced by increased testing during periods of high incidence of other respiratory illnesses. There are a few limitations of this analysis. WHO FluNet is the largest global database of influenza surveillance data, yet may be subject to under reporting or misclassification of influenza cases by national influenza centres. The validity of influenza activity estimates in this analysis is dependent upon the surveillance data quality as reported to WHO. Some countries are large and geographically diverse, thus country-wide summation of influenza cases may oversimplify sub-national trends in seasonality. For instance, influenza seasonality variation by latitude, population density, and climate has been documented in Brazil[37–39], India[40–42], and China[43,44]. Additionally, there was high variability in reporting between years in several countries, especially in the African Region, resulting in influenza activity estimates based on limited data. To address this variability, we restricted the analysis to countries that reported 50 or more positive influenza cases per year for at least two years and listed the number of years that a country reported sufficient data to WHO FluNet (S1 Table). Though virologic strain specifications are reported on FluNet, these data were not considered robust enough for inclusion in our analysis at this time.

## Conclusion

We characterized patterns of influenza activity in 118 countries across all six WHO regions using influenza surveillance data reported between 2011 and 2016. By using transparent methods and publicly available data, our analysis can be replicated on an individual country basis and updated as local surveillance systems mature. The timing, duration, and peak of influenza activity is an essential component in planning an influenza program and can be used to guide or refine national vaccination strategies. Overall, this country-specific characterization may be useful for countries aiming to introduce, scale-up, or align influenza vaccination programs with seasonal influenza trends.

## Supporting information

S1 Appendix Monthly case proportion of influenza cases in 2011–2016, by country. (PDF)

S1 Table Country-specific list of months with influenza activity, number of months with influenza activity, and influenza patterns in 2011–2016, by World Health Organization. (DOCX)
