# Supplementary material for: Global influenza seasonality to inform country-level vaccine programs: An analysis of WHO FluNet influenza surveillance data between 2011 and 2016
Source: PLoS One. 2018 Feb 21;13(2):e0193263. doi: 10.1371/journal.pone.0193263 (PMC5821378; doi:10.1371/journal.pone.0193263)
Supplement: S1 Appendix — (PDF) [file pone.0193263.s001.pdf]

Influenza cases in Albania, 2011 - 2016

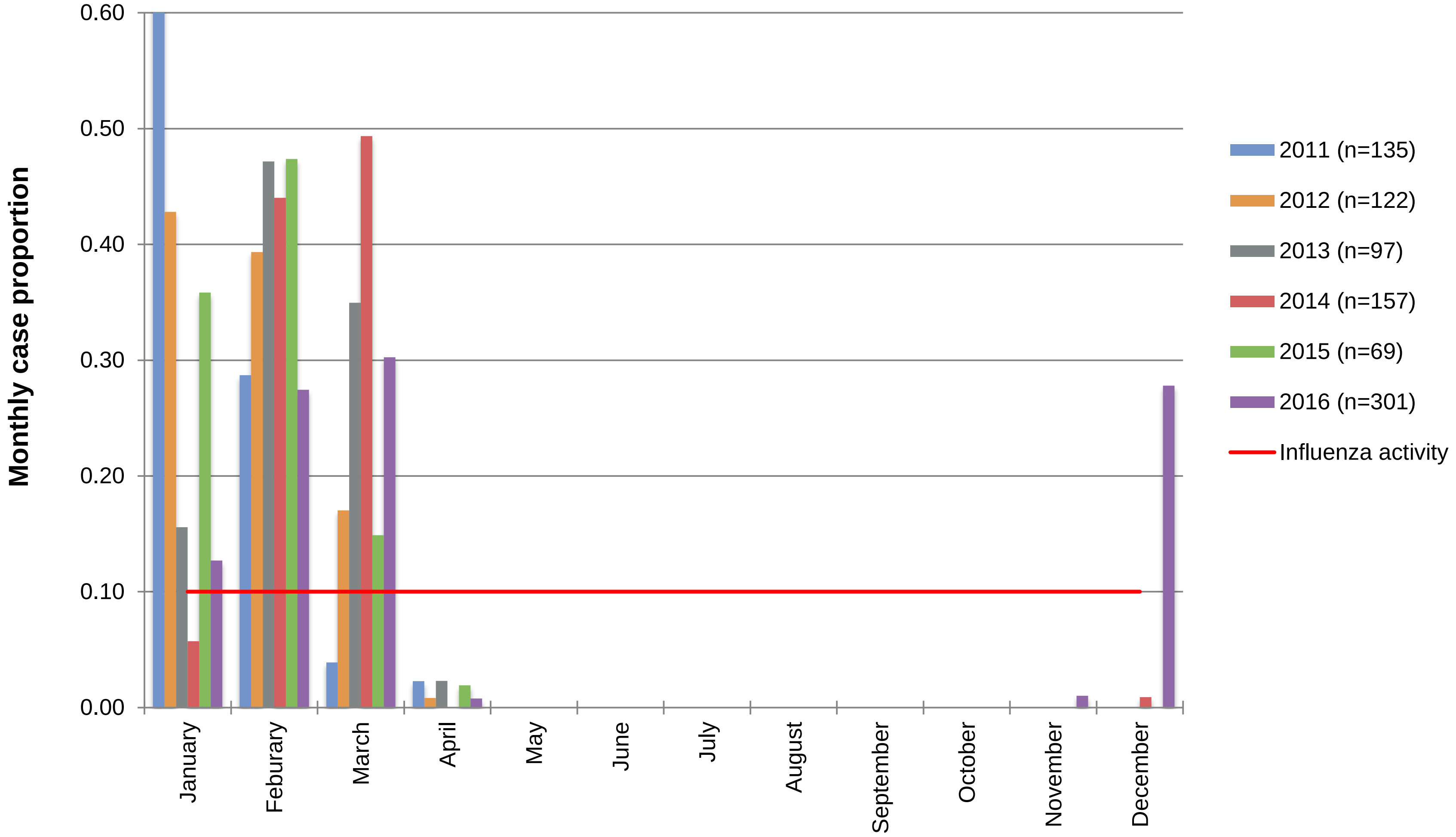

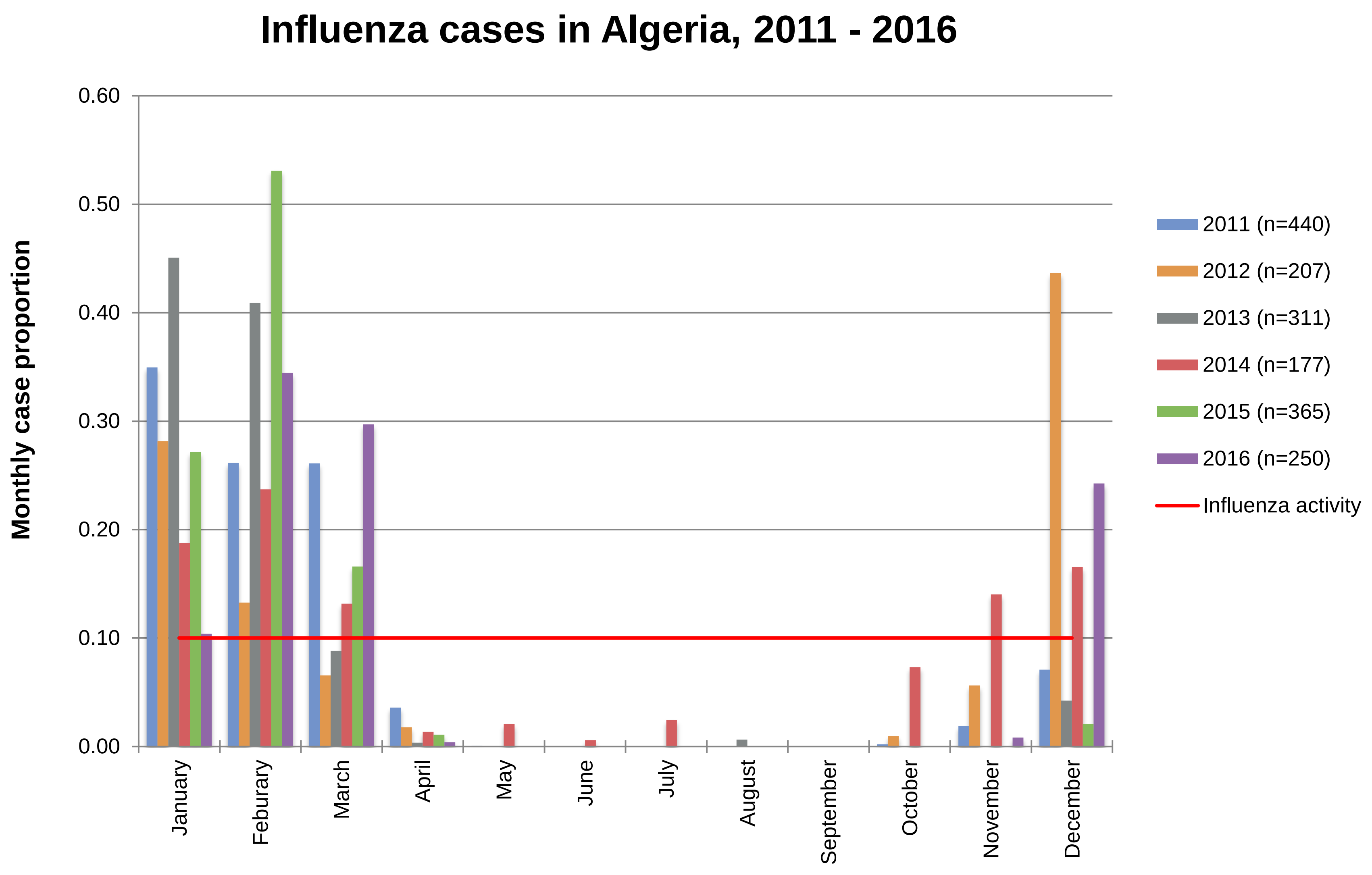
Influenza cases in Algeria, 2011 - 2016
Monthly case proportion
0.60
0.50
0.40
0.30
0.20
0.10
0.00
January
Feburary
March
April
May
June
July
August
September
October
November
December
2011 (n=440)
2012 (n=207)
2013 (n=311)
2014 (n=177)
2015 (n=365)
2016 (n=250)
Influenza activity

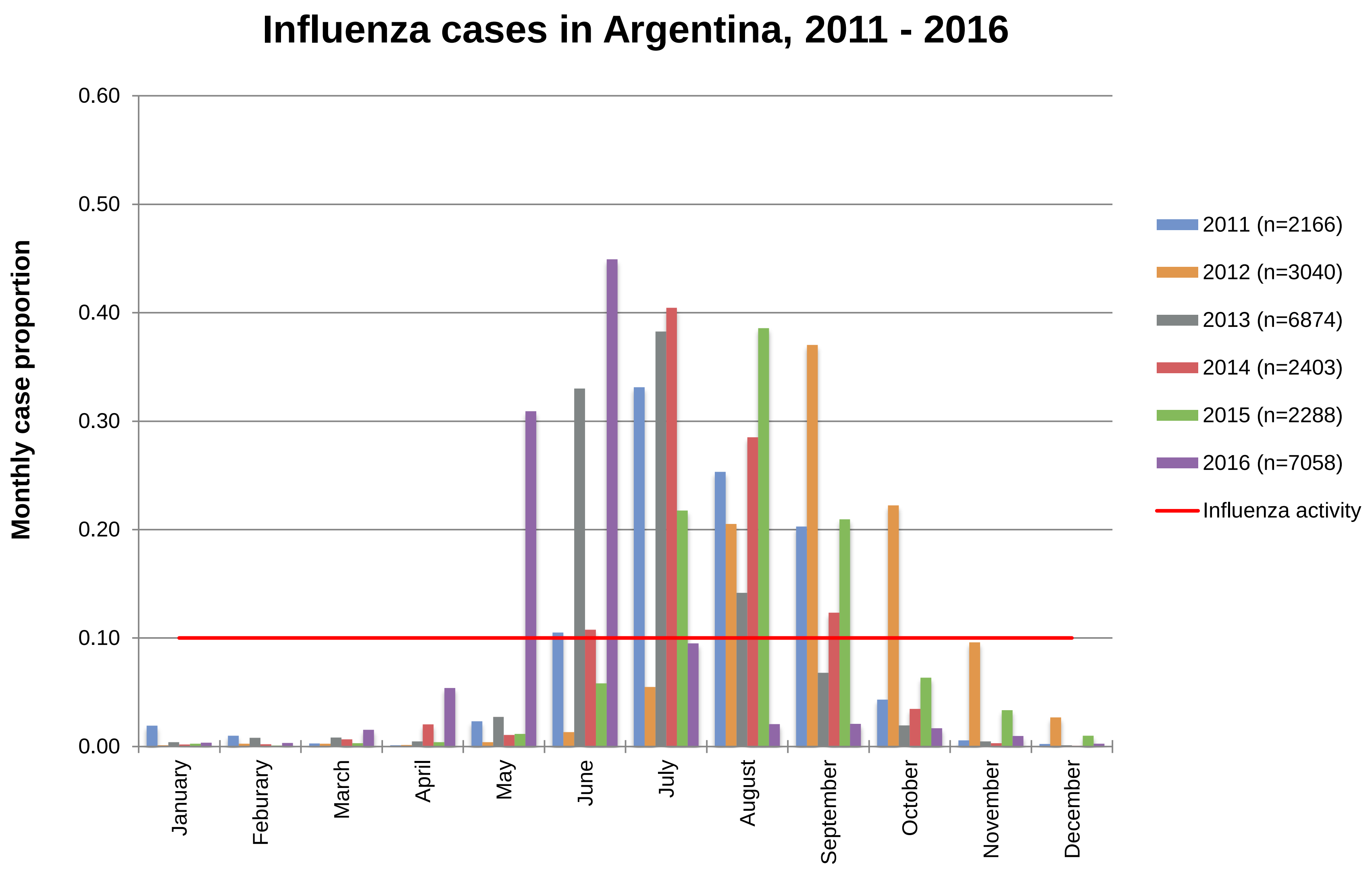
Influenza cases in Argentina, 2011 - 2016
Monthly case proportion
0.60
0.50
0.40
0.30
0.20
0.10
0.00
January
Feburary
March
April
May
June
July
August
September
October
November
December
2011 (n=2166)
2012 (n=3040)
2013 (n=6874)
2014 (n=2403)
2015 (n=2288)
2016 (n=7058)
Influenza activity

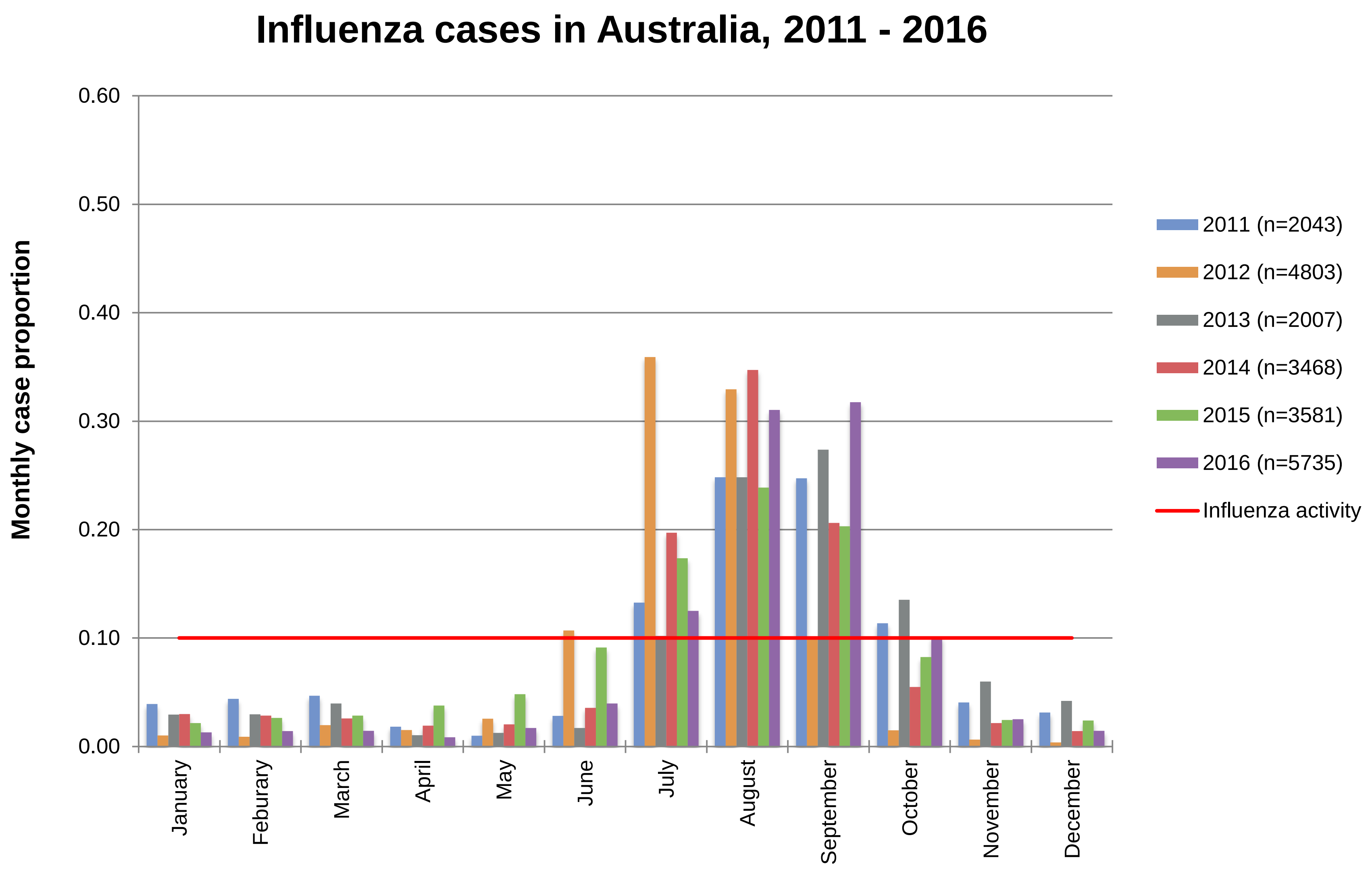
Influenza cases in Australia, 2011 - 2016
Monthly case proportion
0.60
0.50
0.40
0.30
0.20
0.10
0.00
January
Feburary
March
April
May
June
July
August
September
October
November
December
2011 (n=2043)
2012 (n=4803)
2013 (n=2007)
2014 (n=3468)
2015 (n=3581)
2016 (n=5735)
Influenza activity

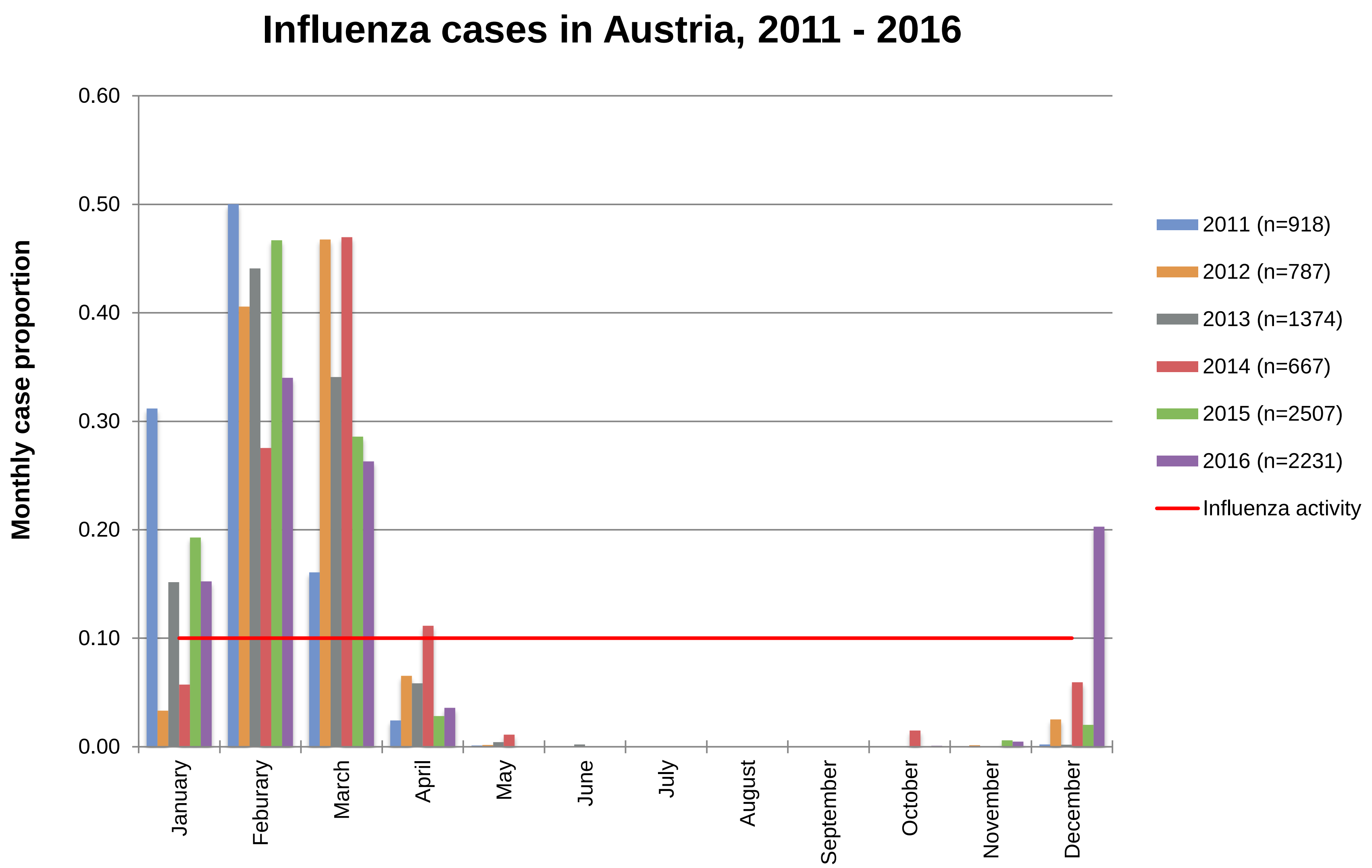

Influenza cases in Austria, 2011 - 2016
Monthly case proportion
0.60
0.50
0.40
0.30
0.20
0.10
0.00
January
Feburary
March
April
May
June
July
August
September
October
November
December
2011 (n=918)
2012 (n=787)
2013 (n=1374)
2014 (n=667)
2015 (n=2507)
2016 (n=2231)
Influenza activity

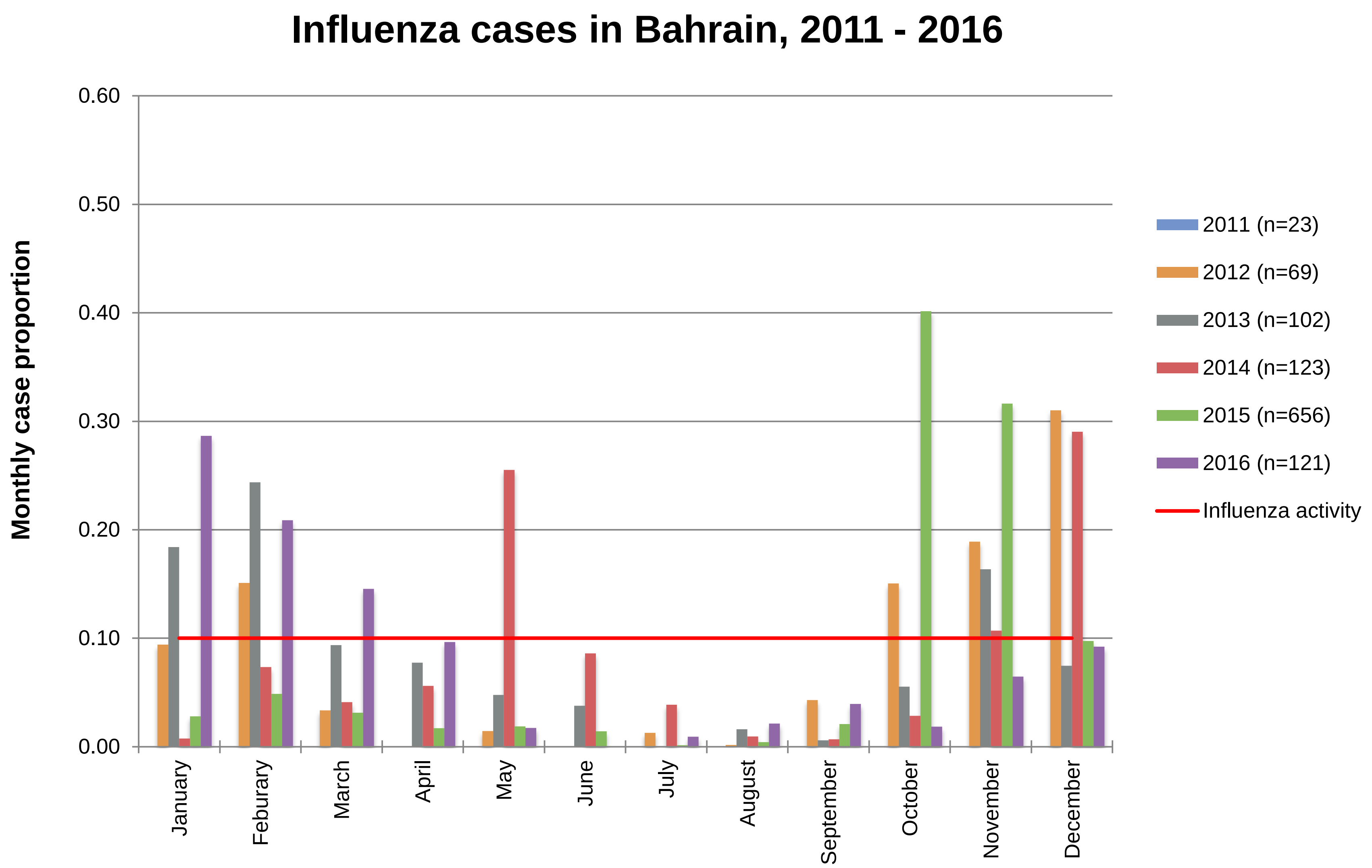
Influenza cases in Bahrain, 2011 - 2016
Monthly case proportion
0.60
0.50
0.40
0.30
0.20
0.10
0.00
January
Feburary
March
April
May
June
July
August
September
October
November
December
2011 (n=23)
2012 (n=69)
2013 (n=102)
2014 (n=123)
2015 (n=656)
2016 (n=121)
Influenza activity

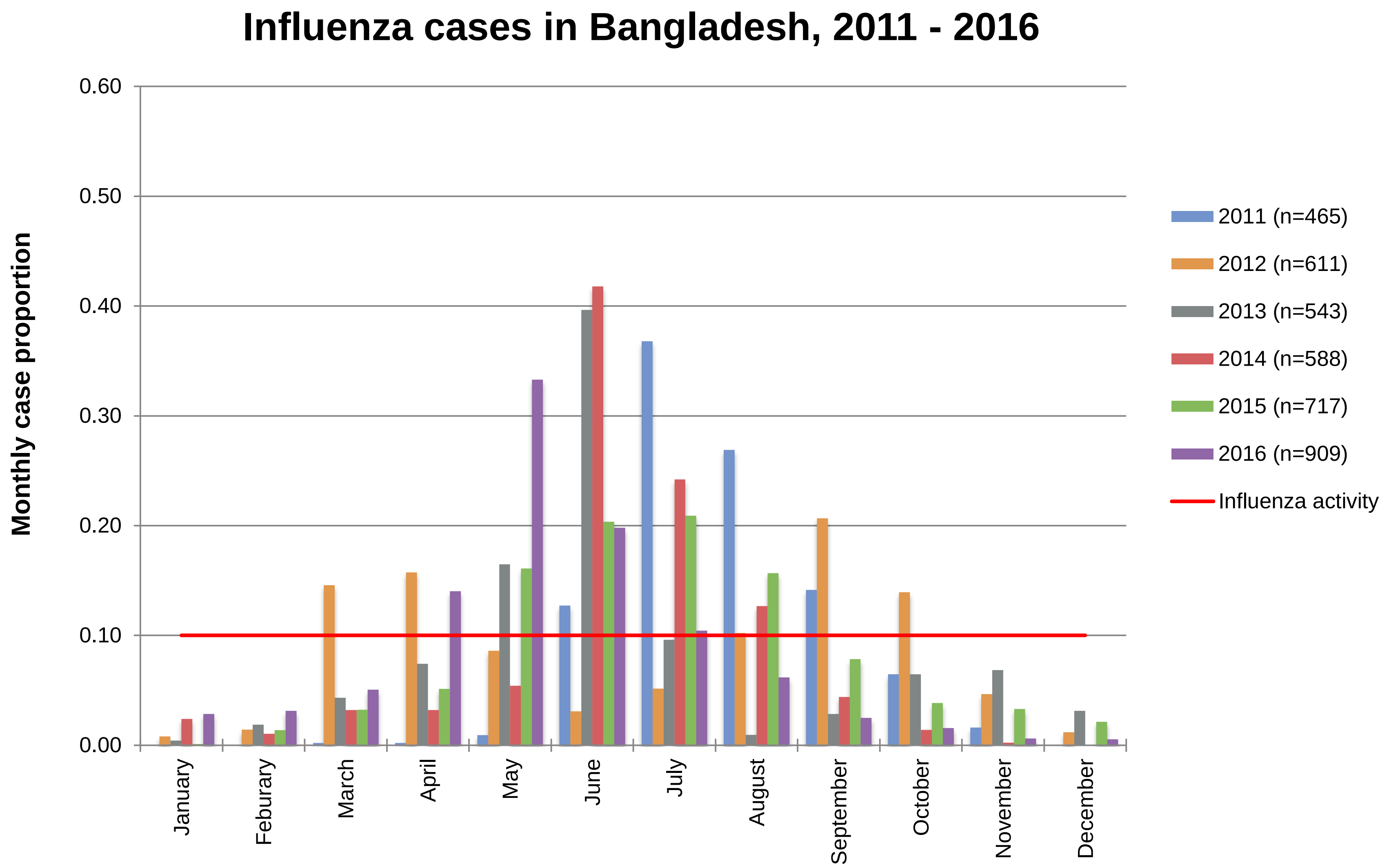
Influenza cases in Bangladesh, 2011 - 2016
Monthly case proportion
0.60
0.50
0.40
0.30
0.20
0.10
0.00
January
Feburary
March
April
May
June
July
August
September
October
November
December
2011 (n=465)
2012 (n=611)
2013 (n=543)
2014 (n=588)
2015 (n=717)
2016 (n=909)
Influenza activity

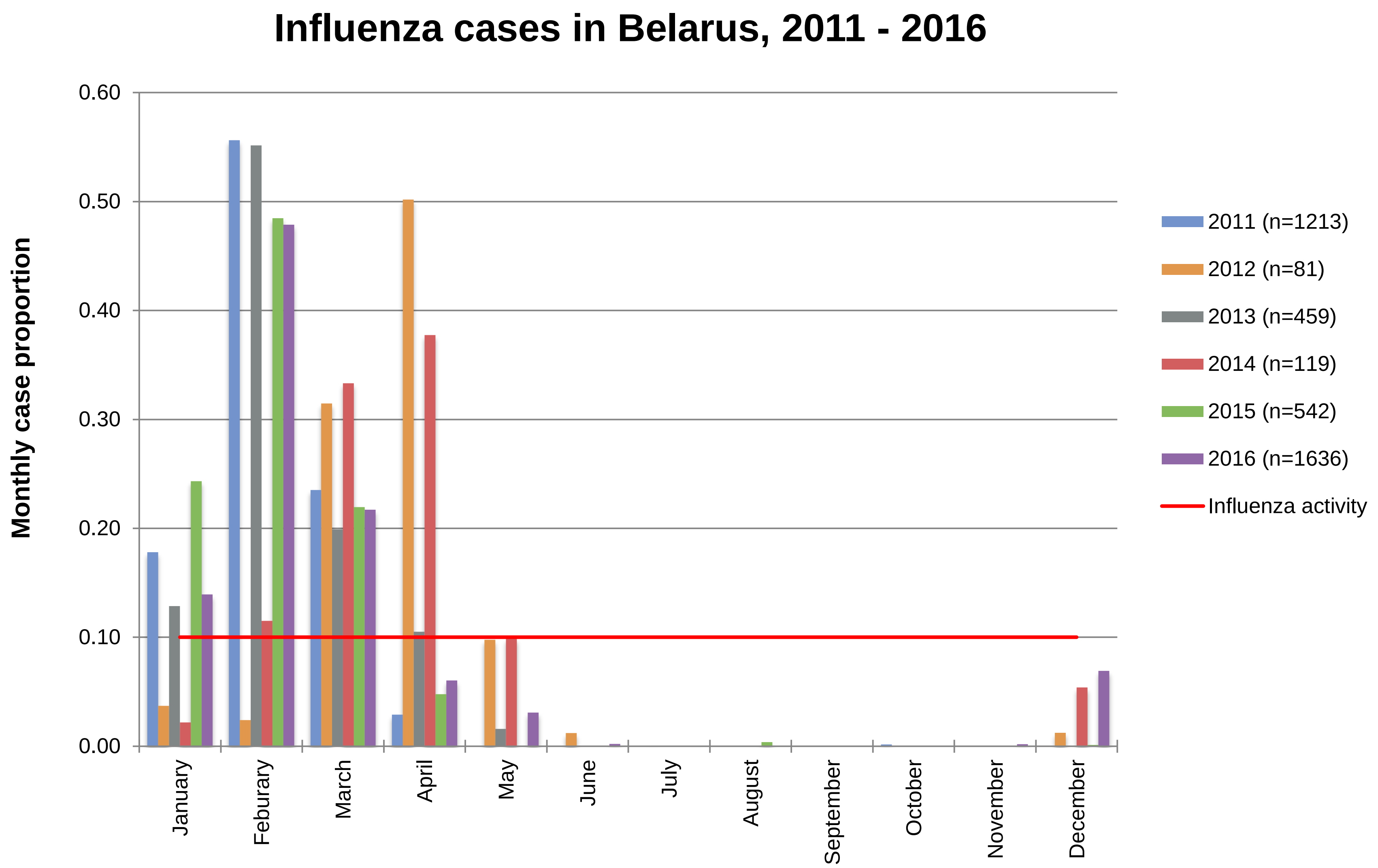

Influenza cases in Belarus, 2011 - 2016
Monthly case proportion
0.60
0.50
0.40
0.30
0.20
0.10
0.00
January
Feburary
March
April
May
June
July
August
September
October
November
December
2011 (n=1213)
2012 (n=81)
2013 (n=459)
2014 (n=119)
2015 (n=542)
2016 (n=1636)
Influenza activity

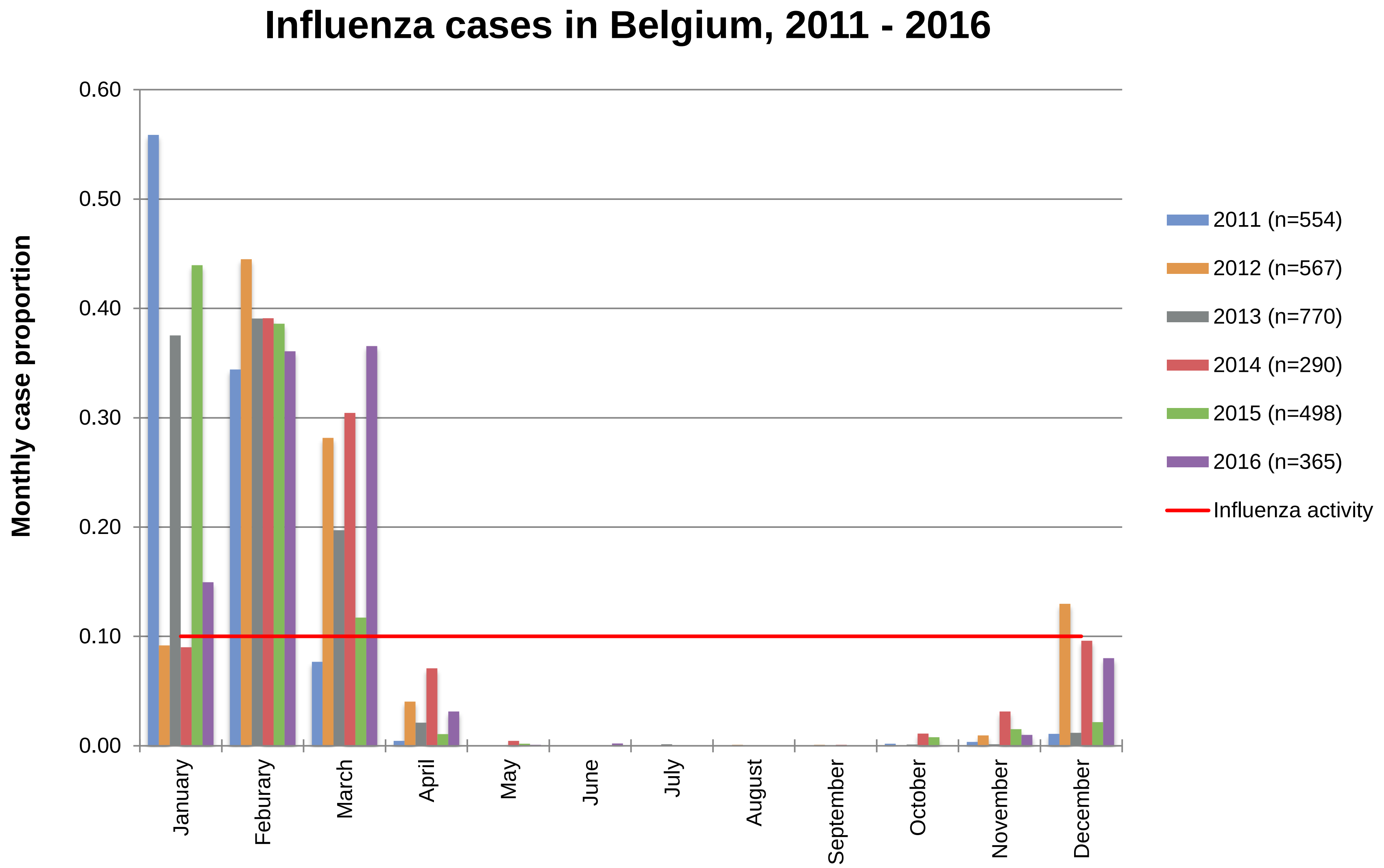
Influenza cases in Belgium, 2011 - 2016
Monthly case proportion
0.60
0.50
0.40
0.30
0.20
0.10
0.00
January
Feburary
March
April
May
June
July
August
September
October
November
December
2011 (n=554)
2012 (n=567)
2013 (n=770)
2014 (n=290)
2015 (n=498)
2016 (n=365)
Influenza activity

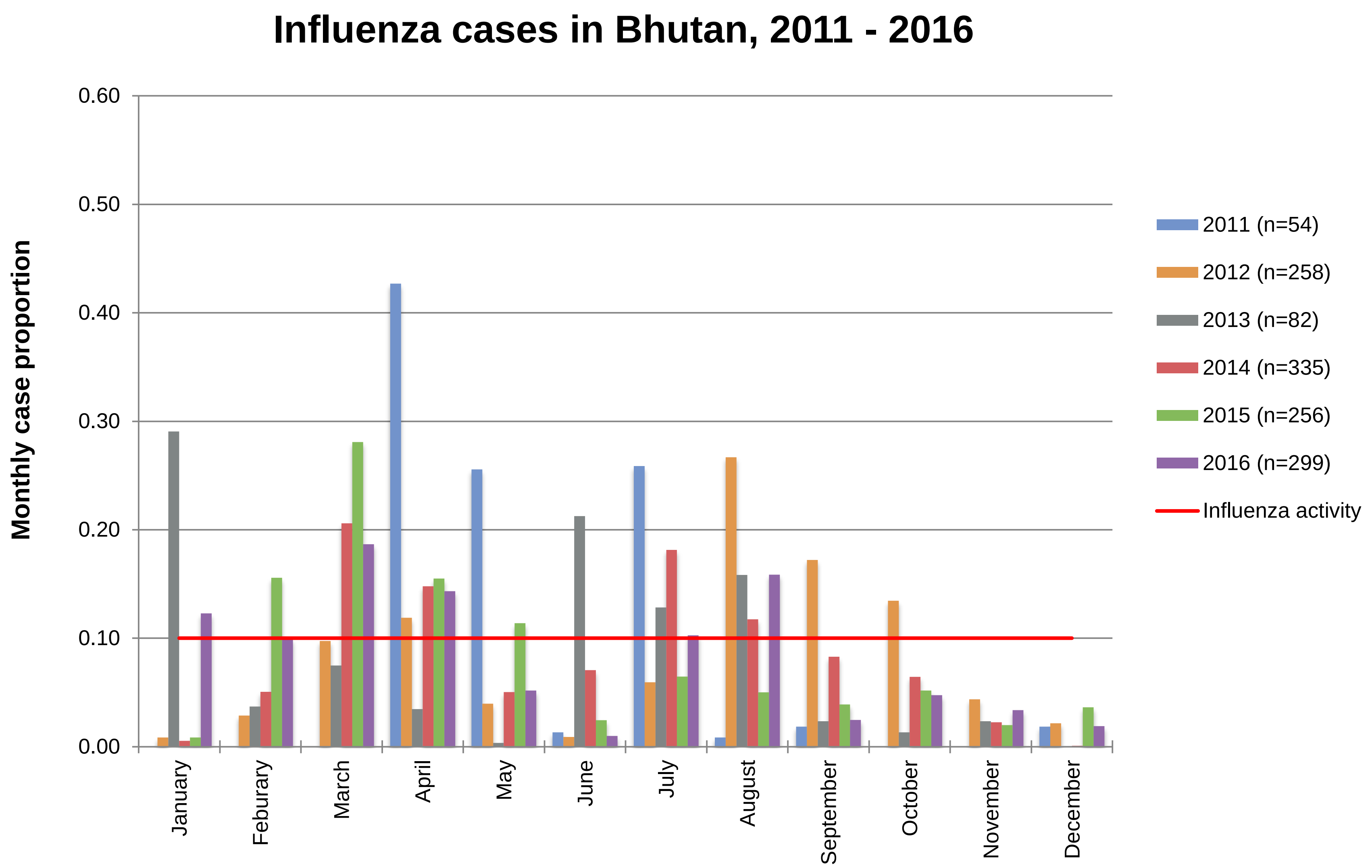

Influenza cases in Bhutan, 2011 - 2016
Monthly case proportion
0.60
0.50
0.40
0.30
0.20
0.10
0.00
January
Feburary
March
April
May
June
July
August
September
October
November
December
2011 (n=54)
2012 (n=258)
2013 (n=82)
2014 (n=335)
2015 (n=256)
2016 (n=299)
Influenza activity

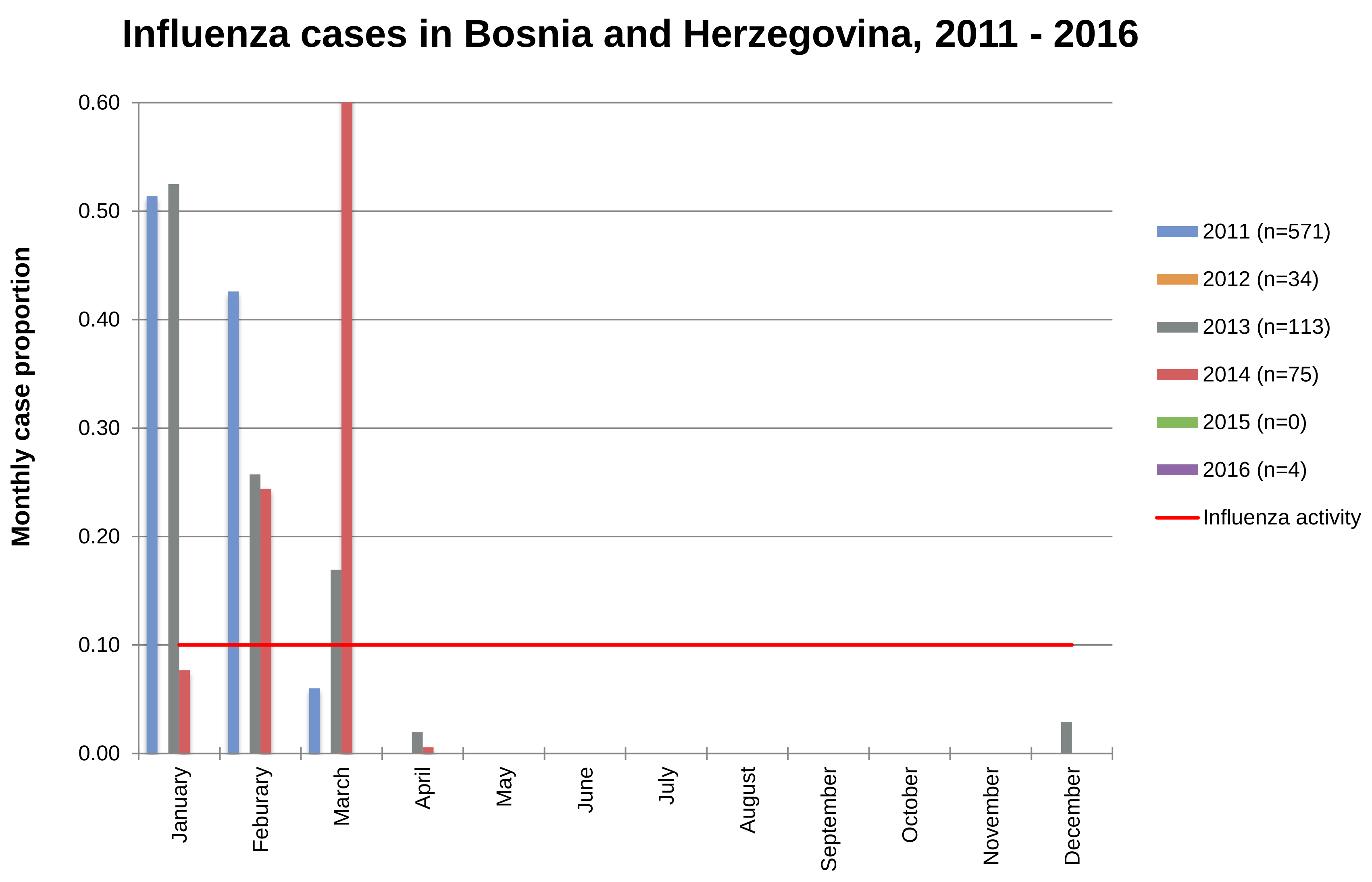
Influenza cases in Bosnia and Herzegovina, 2011 - 2016
Monthly case proportion
0.60
0.50
0.40
0.30
0.20
0.10
0.00
January
Feburary
March
April
May
June
July
August
September
October
November
December
2011 (n=571)
2012 (n=34)
2013 (n=113)
2014 (n=75)
2015 (n=0)
2016 (n=4)
Influenza activity

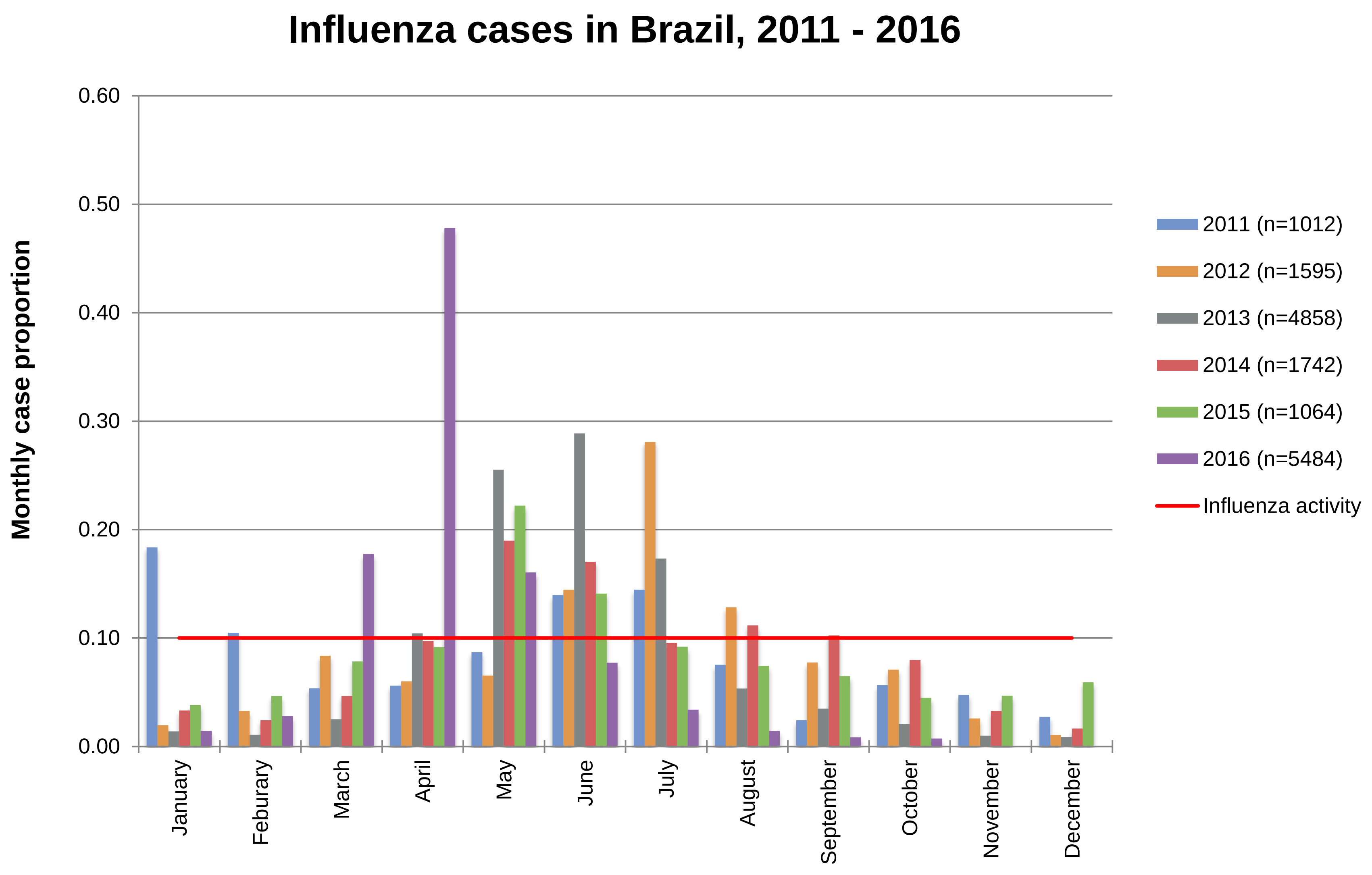

Influenza cases in Brazil, 2011 - 2016
Monthly case proportion
0.60
0.50
0.40
0.30
0.20
0.10
0.00
January
Feburary
March
April
May
June
July
August
September
October
November
December
2011 (n=1012)
2012 (n=1595)
2013 (n=4858)
2014 (n=1742)
2015 (n=1064)
2016 (n=5484)
Influenza activity

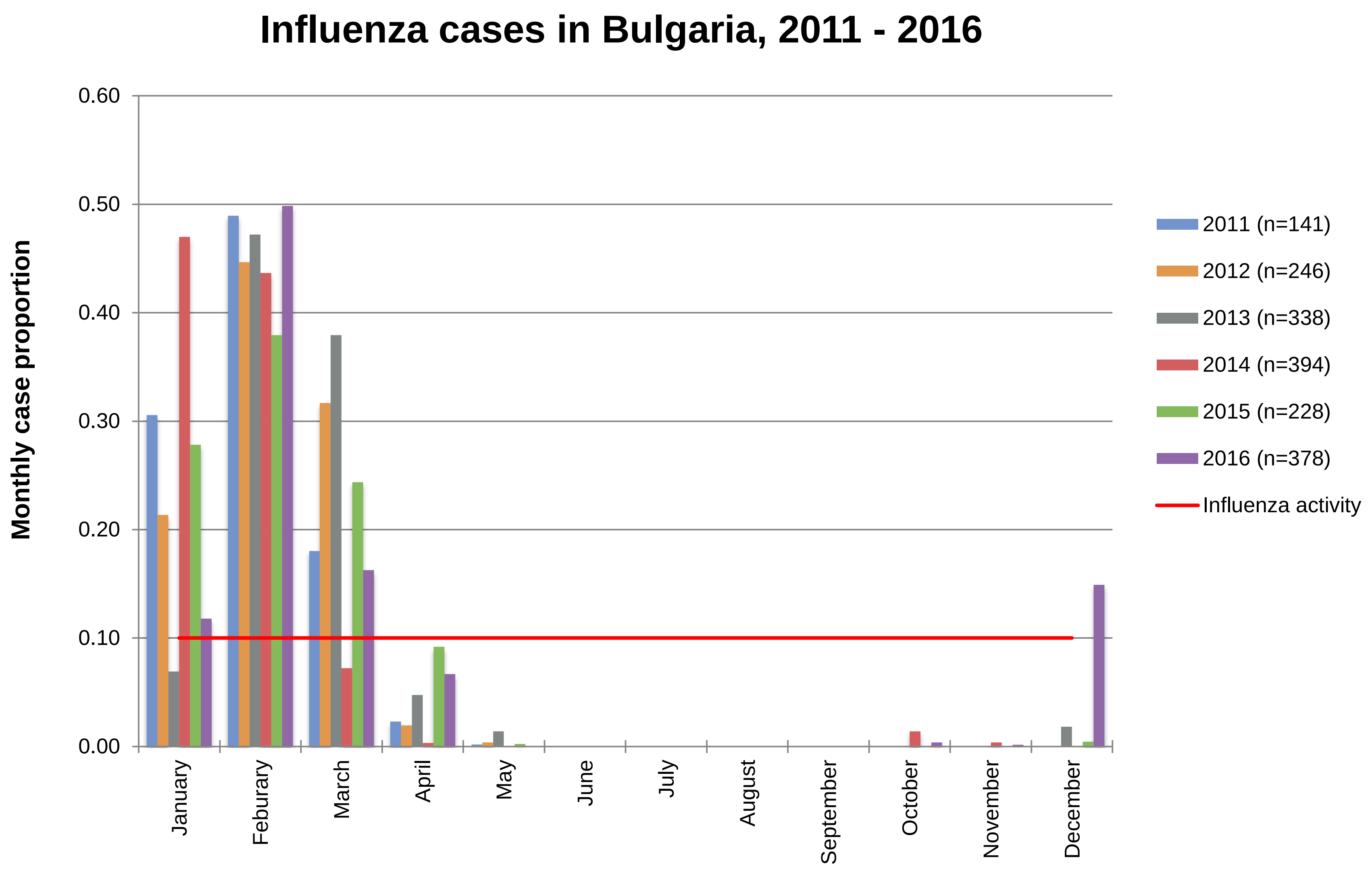
Influenza cases in Bulgaria, 2011 - 2016
Monthly case proportion
0.60
0.50
0.40
0.30
0.20
0.10
0.00
January
Feburary
March
April
May
June
July
August
September
October
November
December
2011 (n=141)
2012 (n=246)
2013 (n=338)
2014 (n=394)
2015 (n=228)
2016 (n=378)
Influenza activity

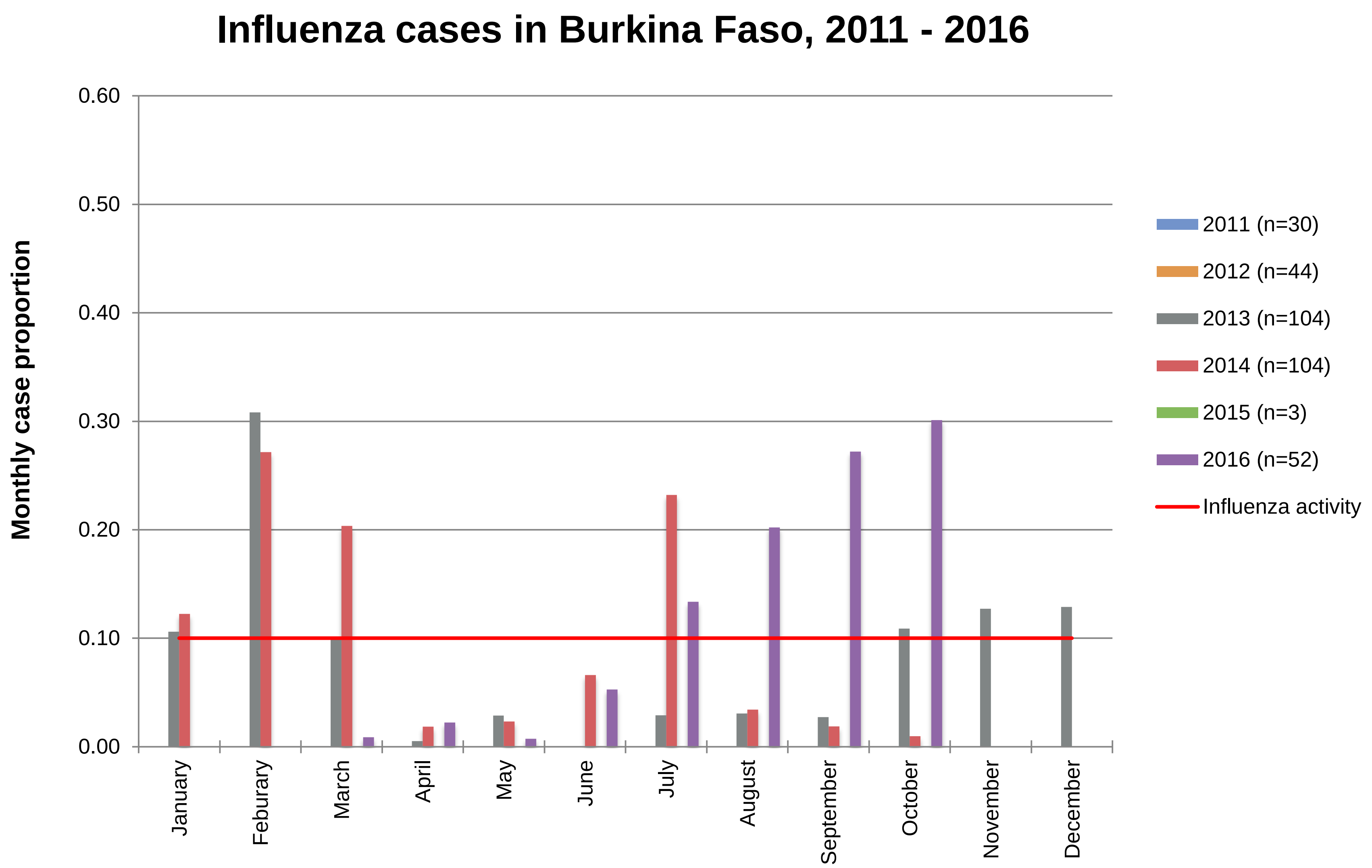

Influenza cases in Burkina Faso, 2011 - 2016
Monthly case proportion
0.60
0.50
0.40
0.30
0.20
0.10
0.00
January
Feburary
March
April
May
June
July
August
September
October
November
December
2011 (n=30)
2012 (n=44)
2013 (n=104)
2014 (n=104)
2015 (n=3)
2016 (n=52)
Influenza activity

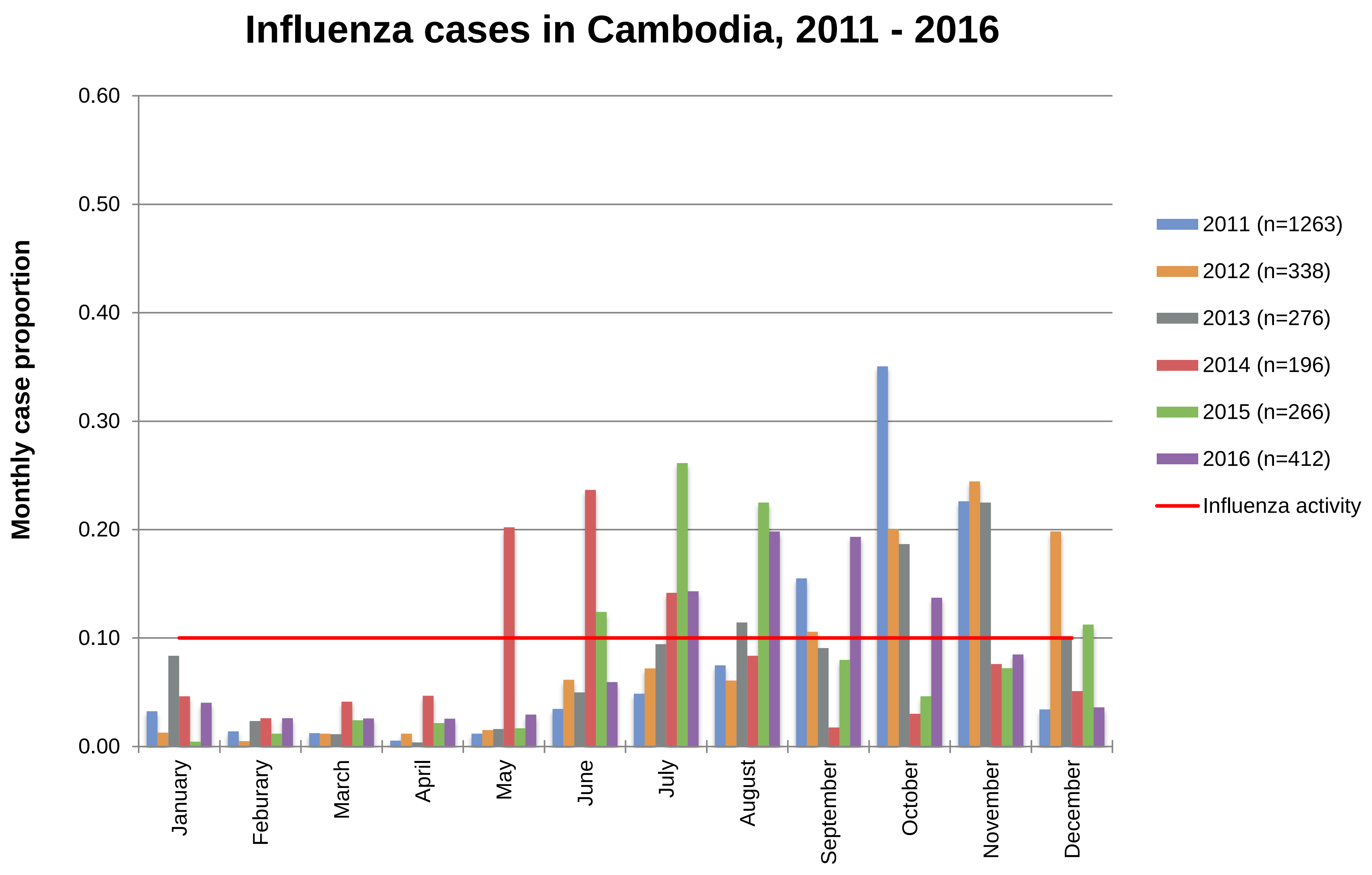
Influenza cases in Cambodia, 2011 - 2016
Monthly case proportion
0.60
0.50
0.40
0.30
0.20
0.10
0.00
January
Feburary
March
April
May
June
July
August
September
October
November
December
2011 (n=1263)
2012 (n=338)
2013 (n=276)
2014 (n=196)
2015 (n=266)
2016 (n=412)
Influenza activity

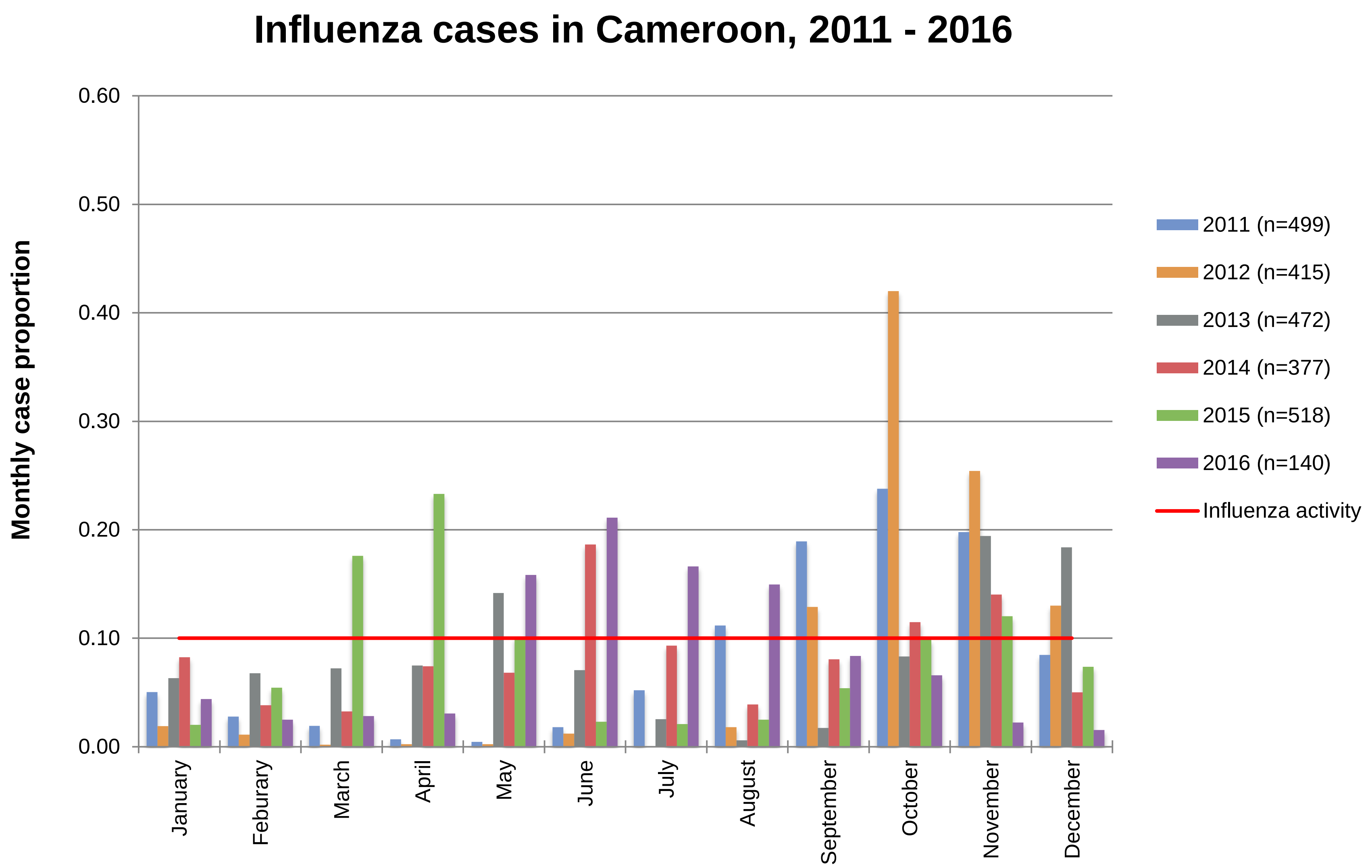
Influenza cases in Cameroon, 2011 - 2016
Monthly case proportion
0.60
0.50
0.40
0.30
0.20
0.10
0.00
January
Feburary
March
April
May
June
July
August
September
October
November
December
2011 (n=499)
2012 (n=415)
2013 (n=472)
2014 (n=377)
2015 (n=518)
2016 (n=140)
Influenza activity

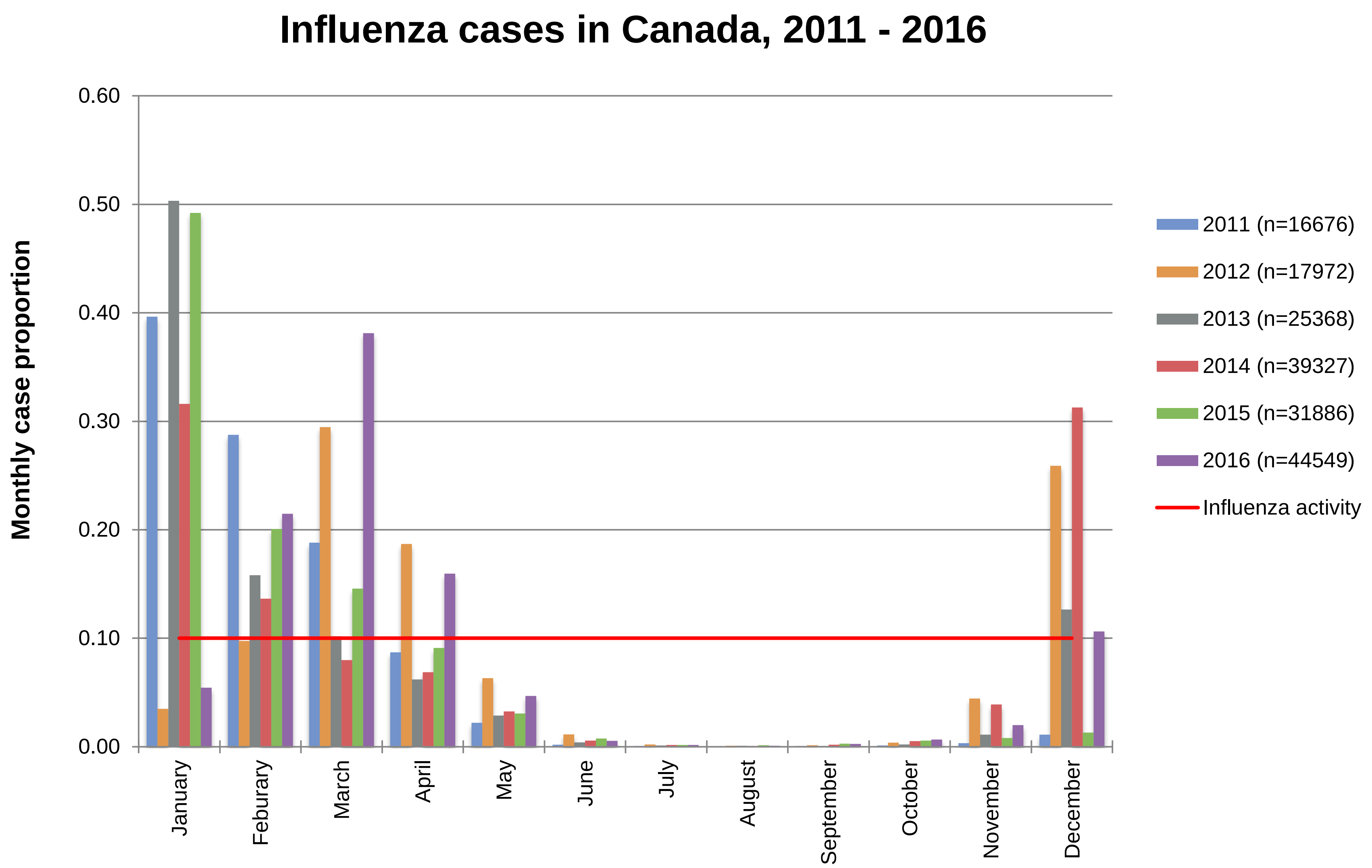
Influenza cases in Canada, 2011 - 2016
Monthly case proportion
0.60
0.50
0.40
0.30
0.20
0.10
0.00
January
Feburary
March
April
May
June
July
August
September
October
November
December
2011 (n=16676)
2012 (n=17972)
2013 (n=25368)
2014 (n=39327)
2015 (n=31886)
2016 (n=44549)
Influenza activity

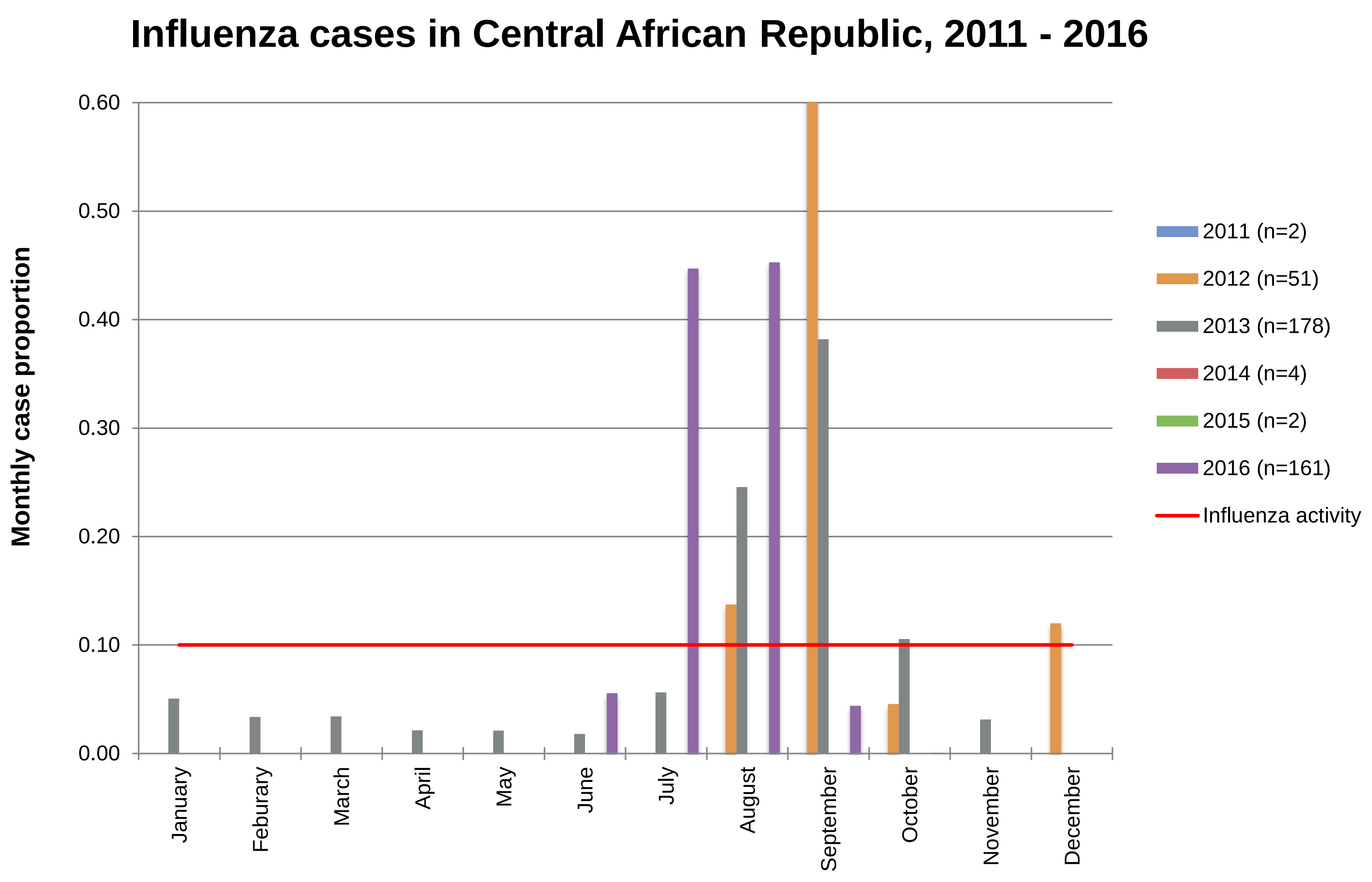
Influenza cases in Central African Republic, 2011 - 2016
Monthly case proportion
0.60
0.50
0.40
0.30
0.20
0.10
0.00
January
Feburary
March
April
May
June
July
August
September
October
November
December
2011 (n=2)
2012 (n=51)
2013 (n=178)
2014 (n=4)
2015 (n=2)
2016 (n=161)
Influenza activity

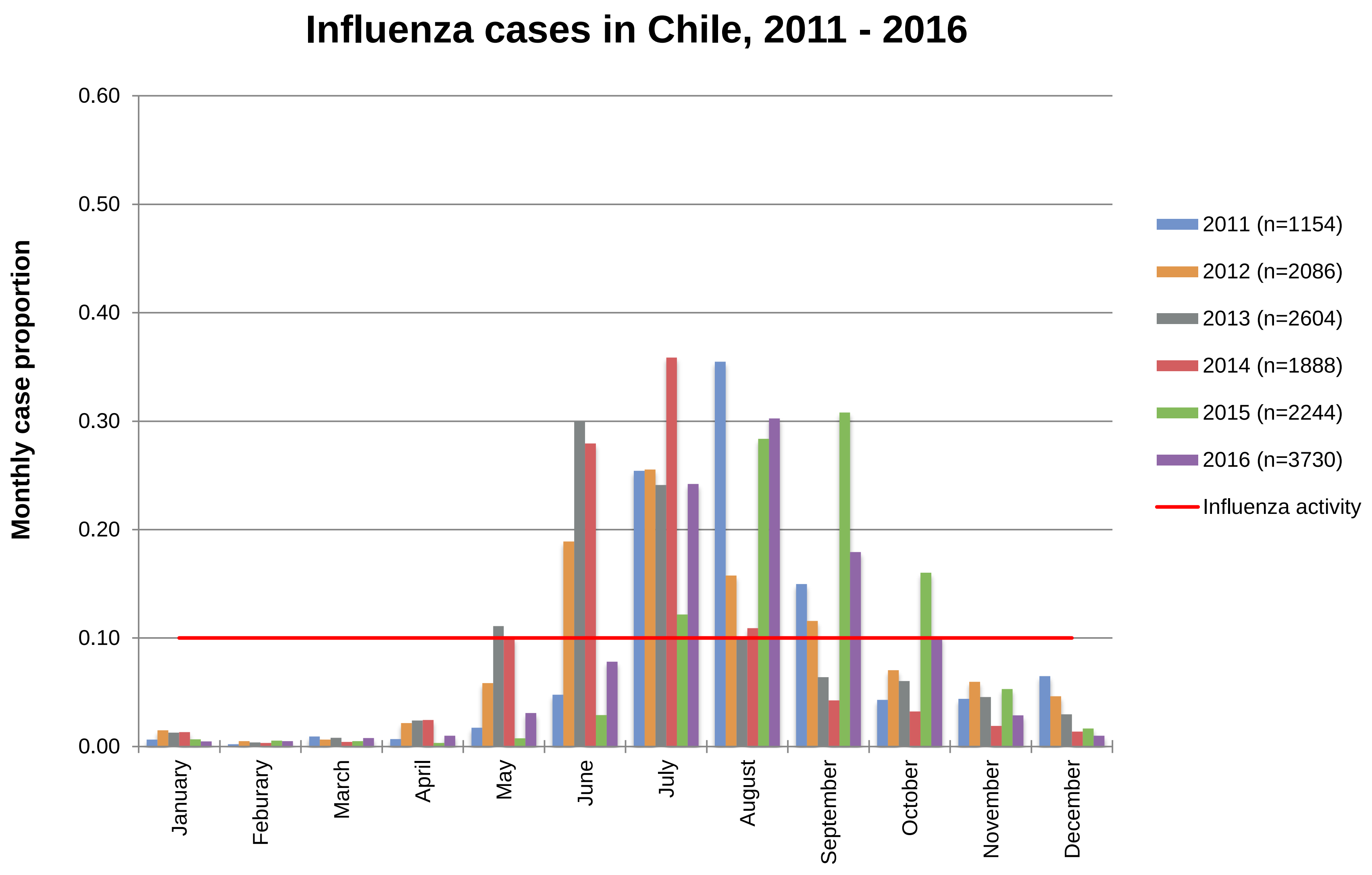

Influenza cases in Chile, 2011 - 2016
Monthly case proportion
0.60
0.50
0.40
0.30
0.20
0.10
0.00
January
Feburary
March
April
May
June
July
August
September
October
November
December
2011 (n=1154)
2012 (n=2086)
2013 (n=2604)
2014 (n=1888)
2015 (n=2244)
2016 (n=3730)
Influenza activity

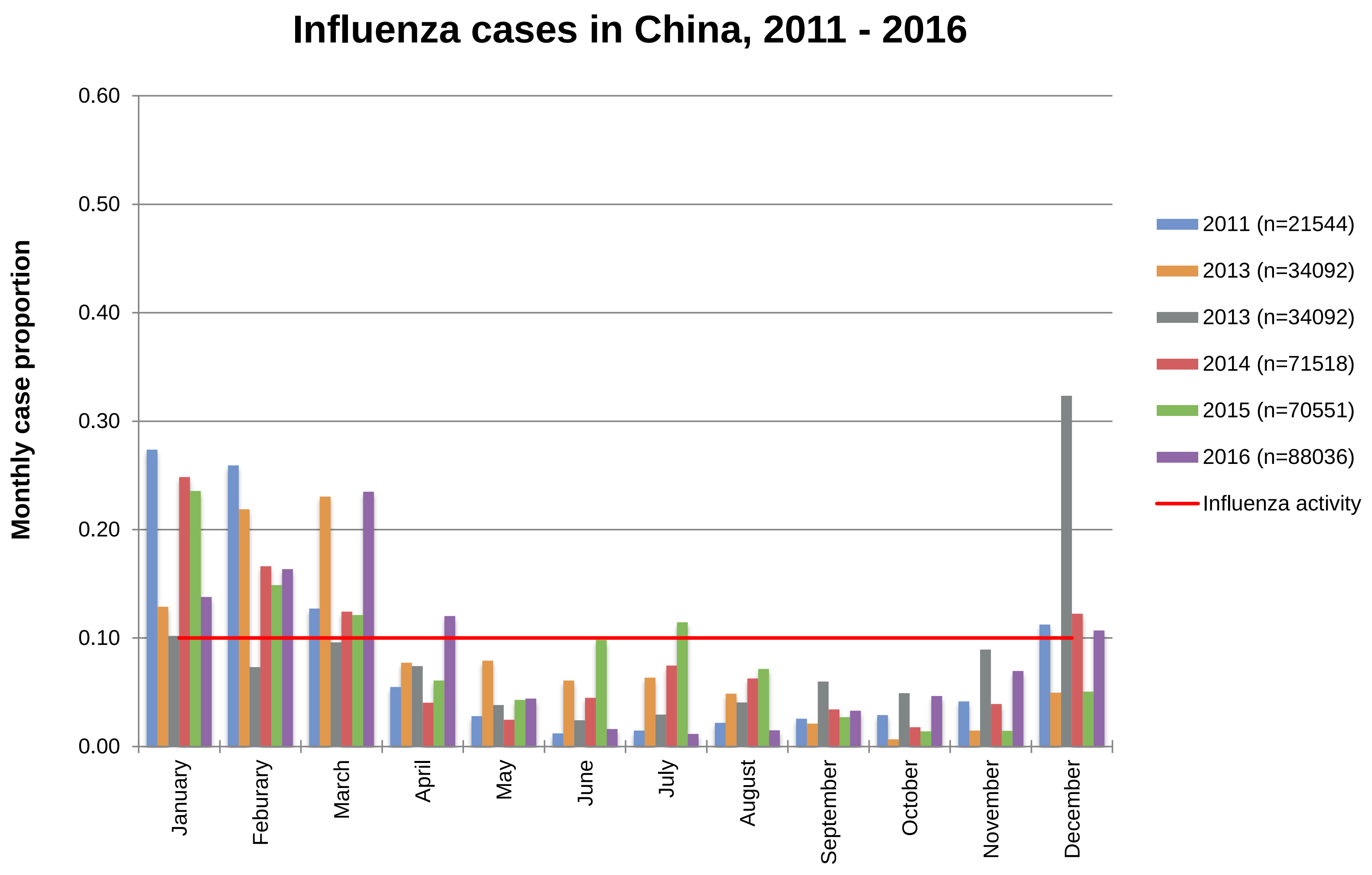

Influenza cases in China, 2011 - 2016
Monthly case proportion
0.60
0.50
0.40
0.30
0.20
0.10
0.00
January
Feburary
March
April
May
June
July
August
September
October
November
December
2011 (n=21544)
2013 (n=34092)
2013 (n=34092)
2014 (n=71518)
2015 (n=70551)
2016 (n=88036)
Influenza activity

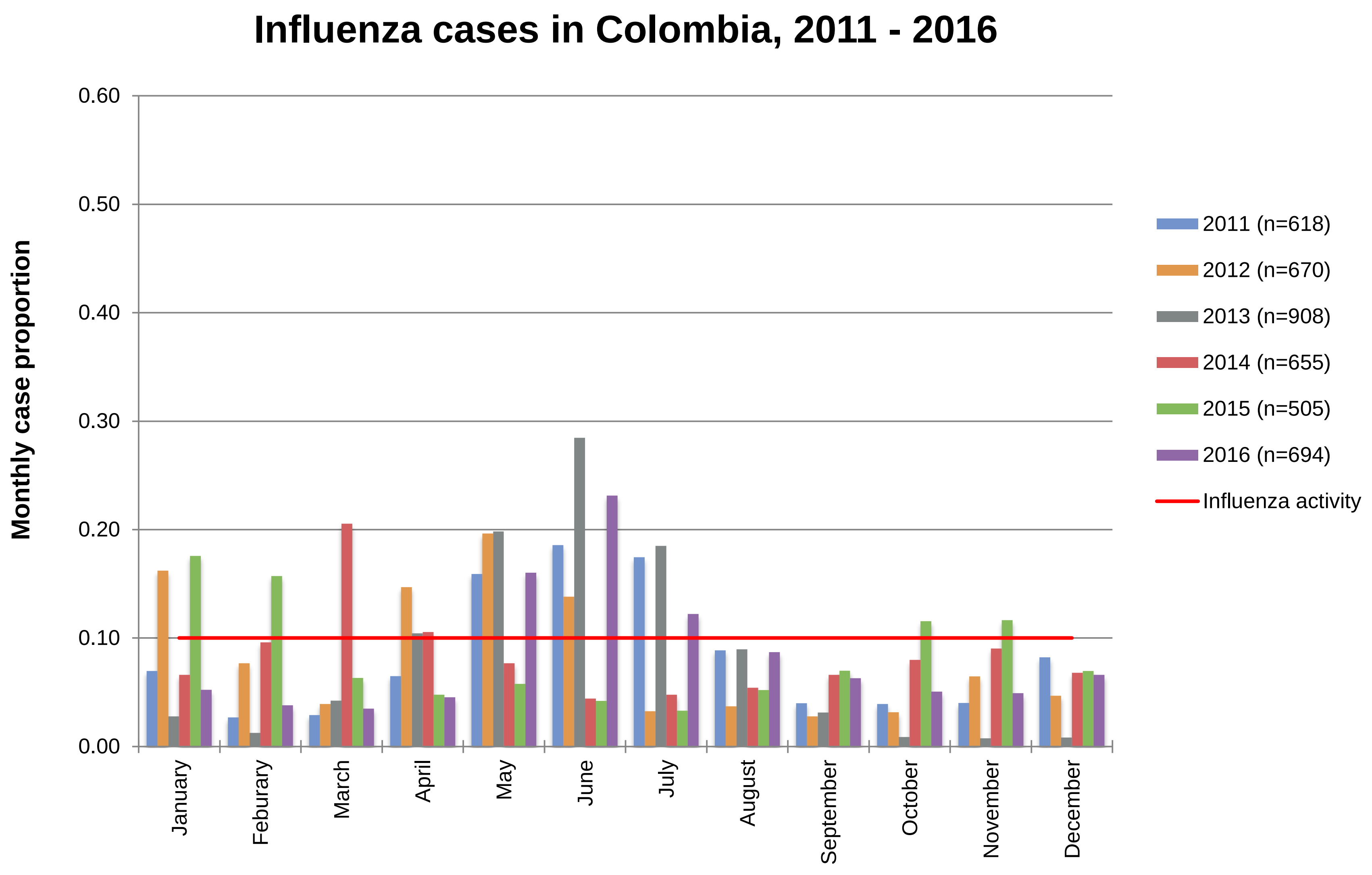
Influenza cases in Colombia, 2011 - 2016
Monthly case proportion
0.60
0.50
0.40
0.30
0.20
0.10
0.00
January
Feburary
March
April
May
June
July
August
September
October
November
December
2011 (n=618)
2012 (n=670)
2013 (n=908)
2014 (n=655)
2015 (n=505)
2016 (n=694)
Influenza activity

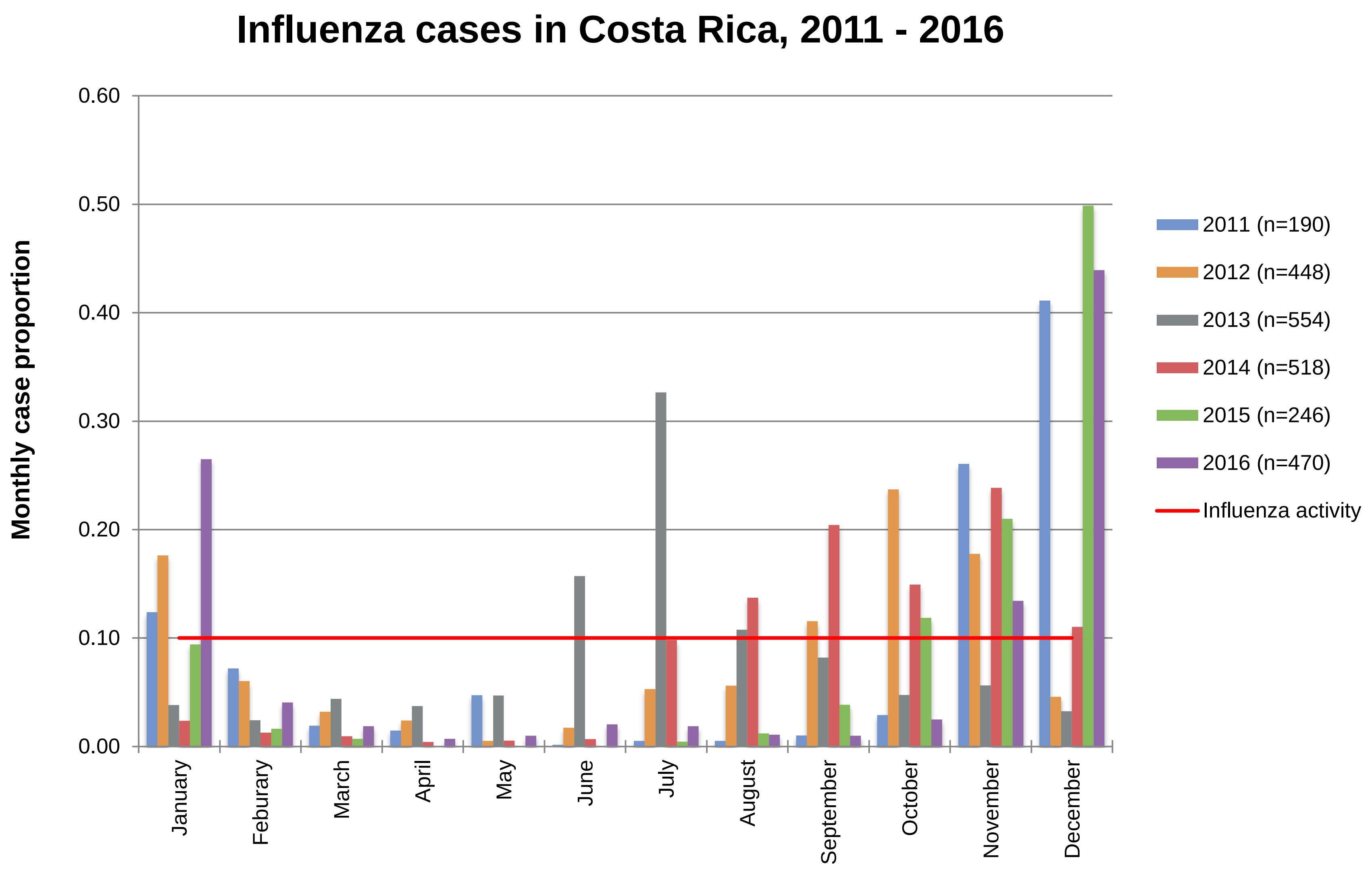
Influenza cases in Costa Rica, 2011 - 2016
Monthly case proportion
0.60
0.50
0.40
0.30
0.20
0.10
0.00
January
Feburary
March
April
May
June
July
August
September
October
November
December
2011 (n=190)
2012 (n=448)
2013 (n=554)
2014 (n=518)
2015 (n=246)
2016 (n=470)
Influenza activity

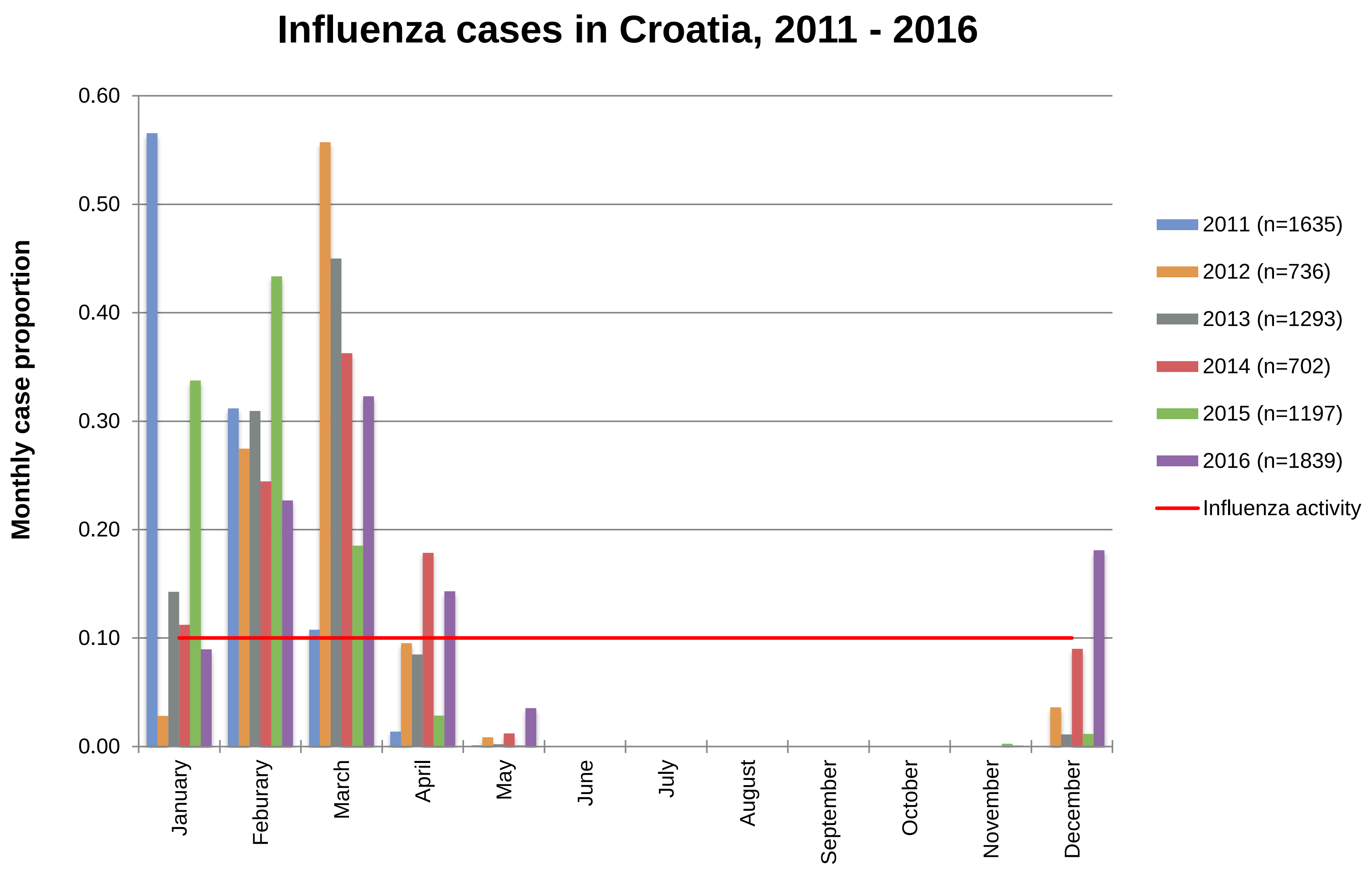

Influenza cases in Croatia, 2011 - 2016
Monthly case proportion
0.60
0.50
0.40
0.30
0.20
0.10
0.00
January
Feburary
March
April
May
June
July
August
September
October
November
December
2011 (n=1635)
2012 (n=736)
2013 (n=1293)
2014 (n=702)
2015 (n=1197)
2016 (n=1839)
Influenza activity

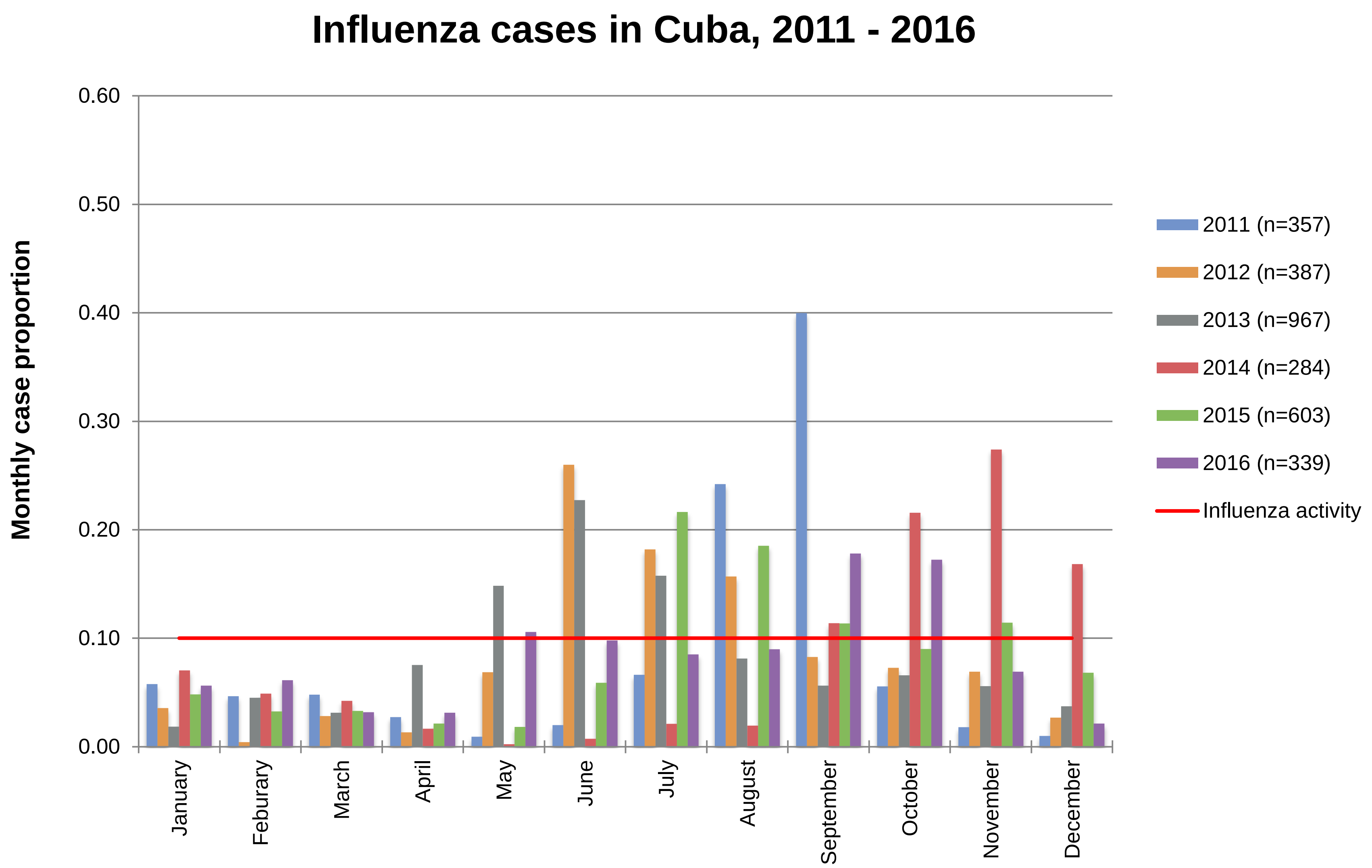
Influenza cases in Cuba, 2011 - 2016
Monthly case proportion
0.60
0.50
0.40
0.30
0.20
0.10
0.00
January
Feburary
March
April
May
June
July
August
September
October
November
December
2011 (n=357)
2012 (n=387)
2013 (n=967)
2014 (n=284)
2015 (n=603)
2016 (n=339)
Influenza activity

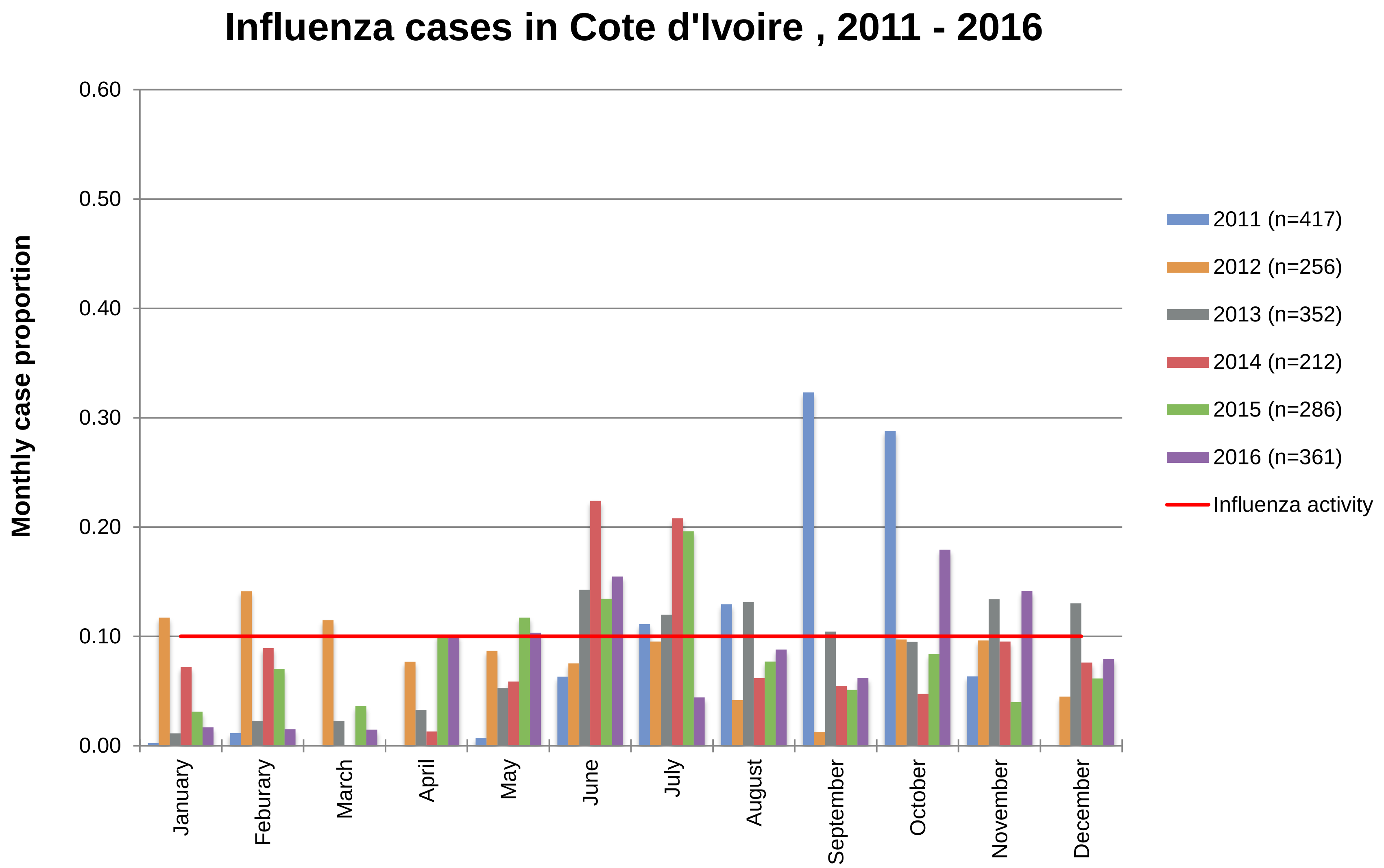

Influenza cases in Cote d'Ivoire , 2011 - 2016
Monthly case proportion
0.60
0.50
0.40
0.30
0.20
0.10
0.00
January
Feburary
March
April
May
June
July
August
September
October
November
December
2011 (n=417)
2012 (n=256)
2013 (n=352)
2014 (n=212)
2015 (n=286)
2016 (n=361)
Influenza activity

Influenza cases in Democratic Republic of the Congo, 2011 - 2016

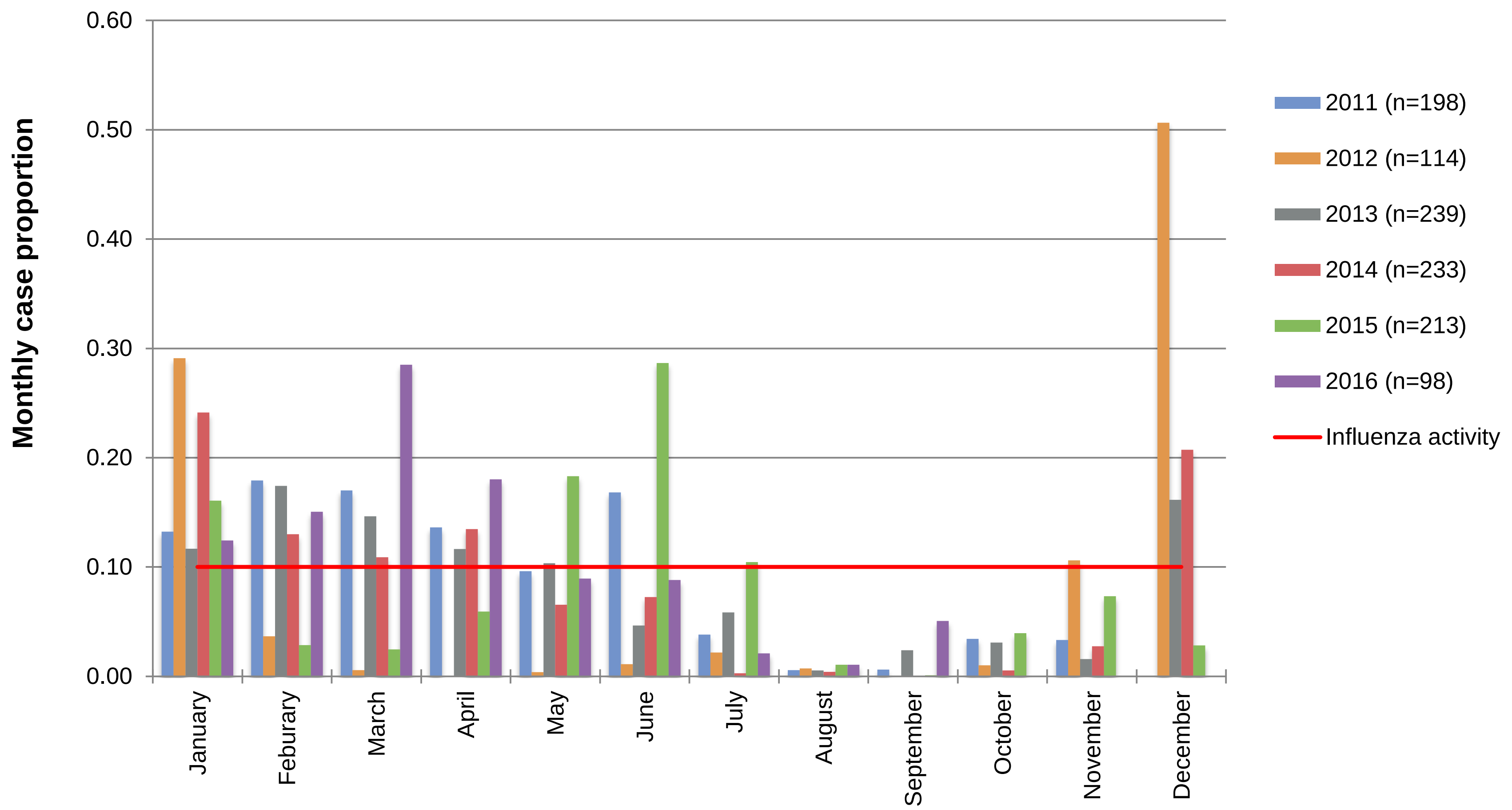

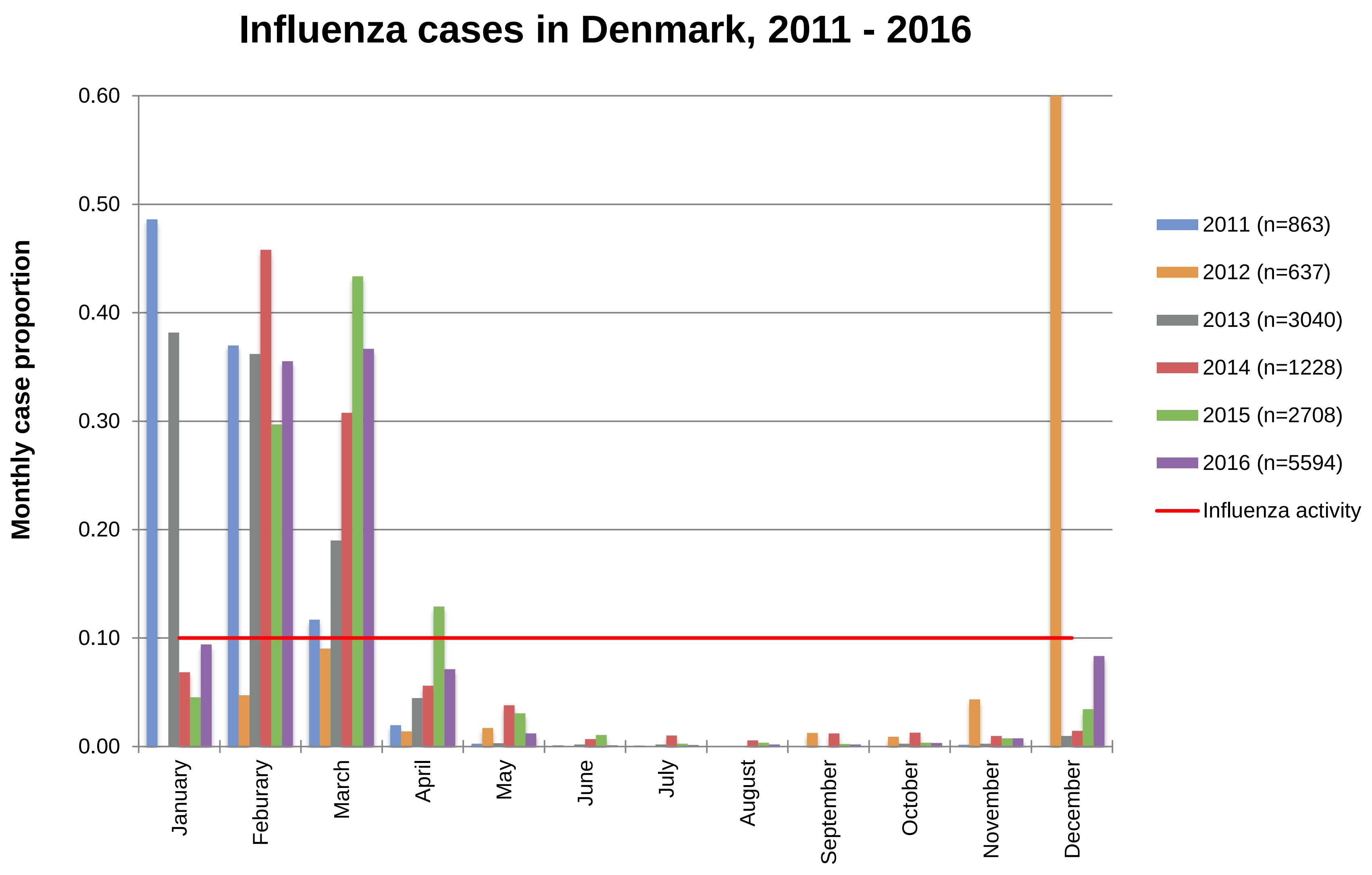
Influenza cases in Denmark, 2011 - 2016
Monthly case proportion
0.60
0.50
0.40
0.30
0.20
0.10
0.00
January
Feburary
March
April
May
June
July
August
September
October
November
December
2011 (n=863)
2012 (n=637)
2013 (n=3040)
2014 (n=1228)
2015 (n=2708)
2016 (n=5594)
Influenza activity

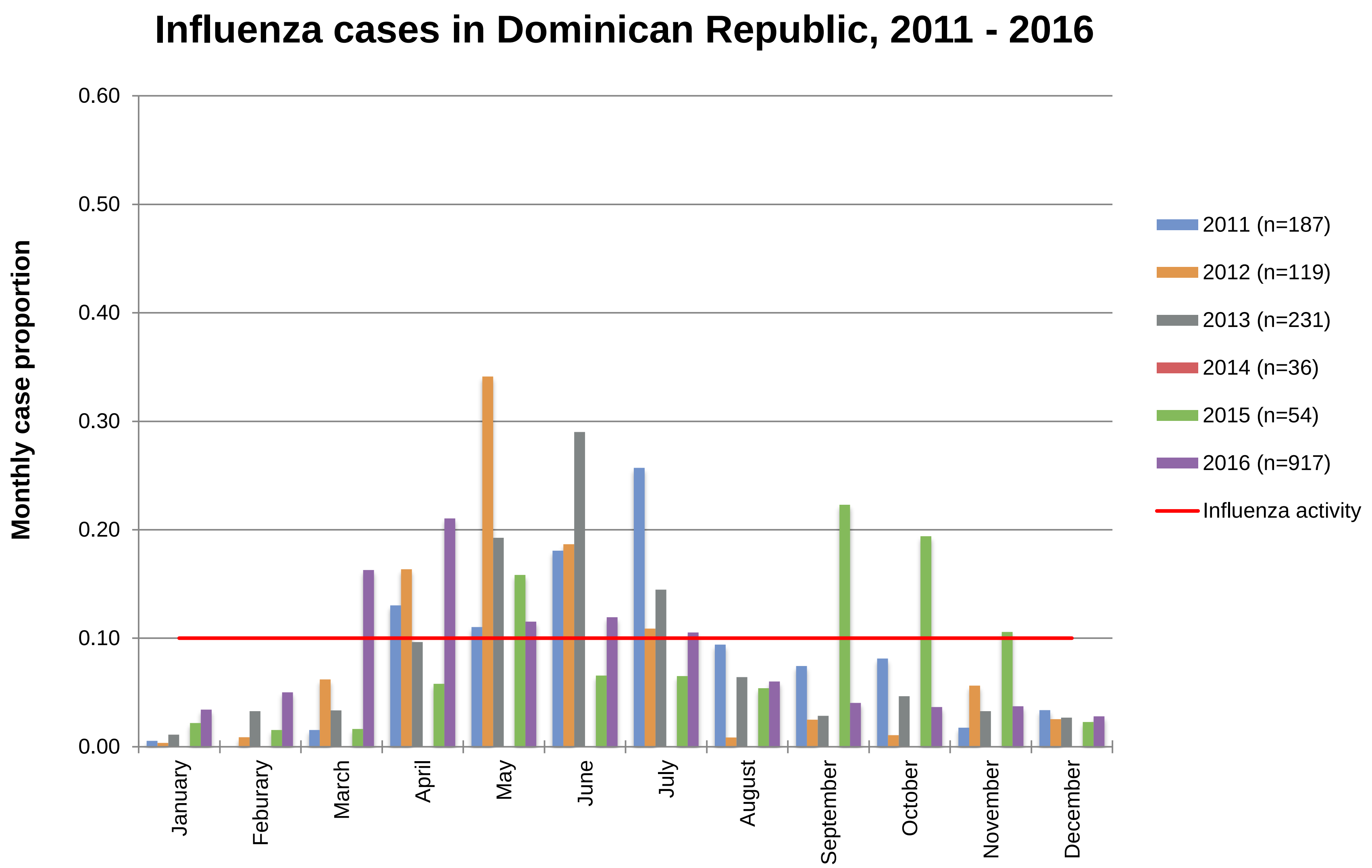

Influenza cases in Dominican Republic, 2011 - 2016
Monthly case proportion
0.60
0.50
0.40
0.30
0.20
0.10
0.00
January
Feburary
March
April
May
June
July
August
September
October
November
December
2011 (n=187)
2012 (n=119)
2013 (n=231)
2014 (n=36)
2015 (n=54)
2016 (n=917)
Influenza activity

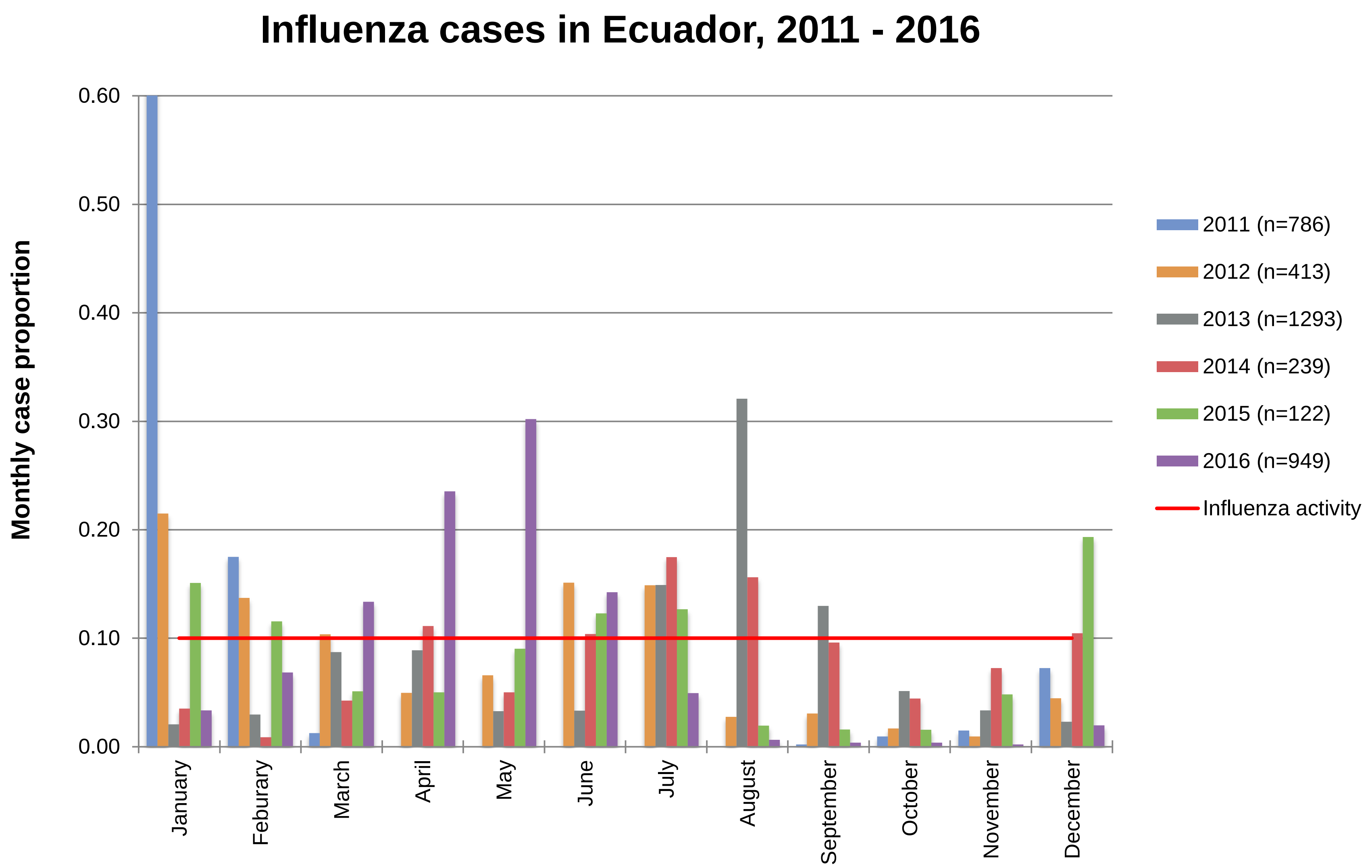

Influenza cases in Ecuador, 2011 - 2016
Monthly case proportion
0.60
0.50
0.40
0.30
0.20
0.10
0.00
January
Feburary
March
April
May
June
July
August
September
October
November
December
2011 (n=786)
2012 (n=413)
2013 (n=1293)
2014 (n=239)
2015 (n=122)
2016 (n=949)
Influenza activity

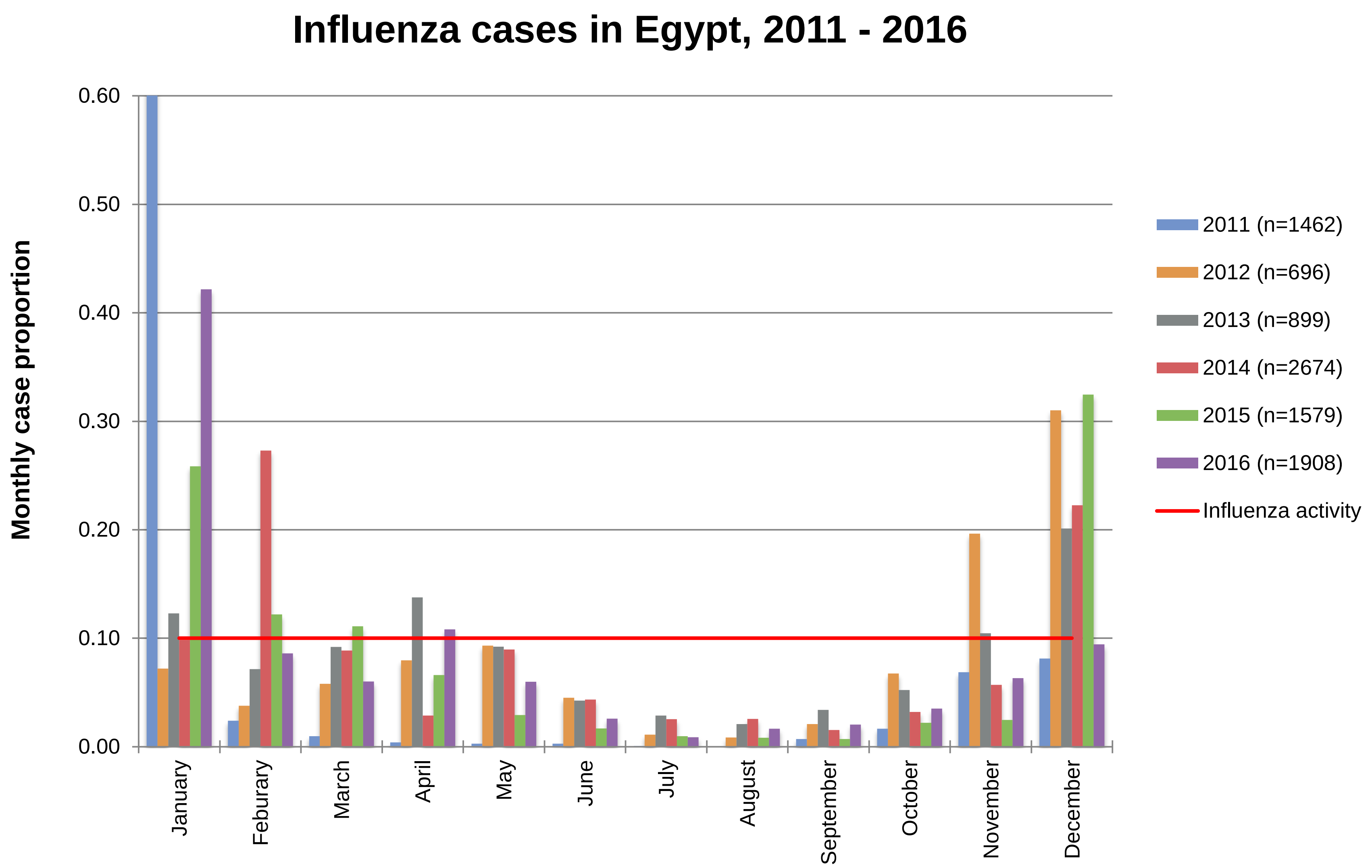
Influenza cases in Egypt, 2011 - 2016
Monthly case proportion
0.60
0.50
0.40
0.30
0.20
0.10
0.00
January
Feburary
March
April
May
June
July
August
September
October
November
December
2011 (n=1462)
2012 (n=696)
2013 (n=899)
2014 (n=2674)
2015 (n=1579)
2016 (n=1908)
Influenza activity

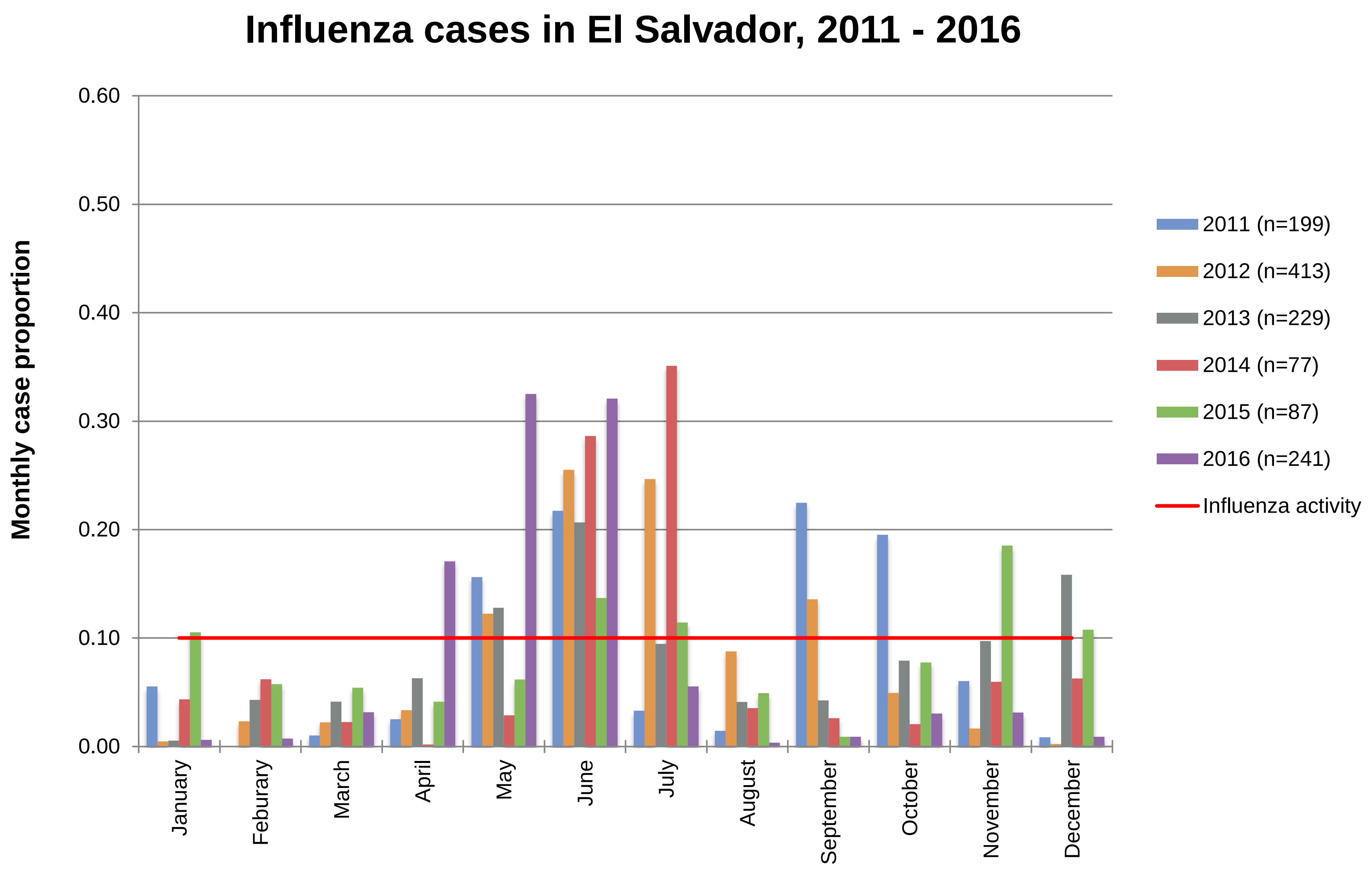
Influenza cases in El Salvador, 2011 - 2016
Monthly case proportion
0.60
0.50
0.40
0.30
0.20
0.10
0.00
January
Feburary
March
April
May
June
July
August
September
October
November
December
2011 (n=199)
2012 (n=413)
2013 (n=229)
2014 (n=77)
2015 (n=87)
2016 (n=241)
Influenza activity

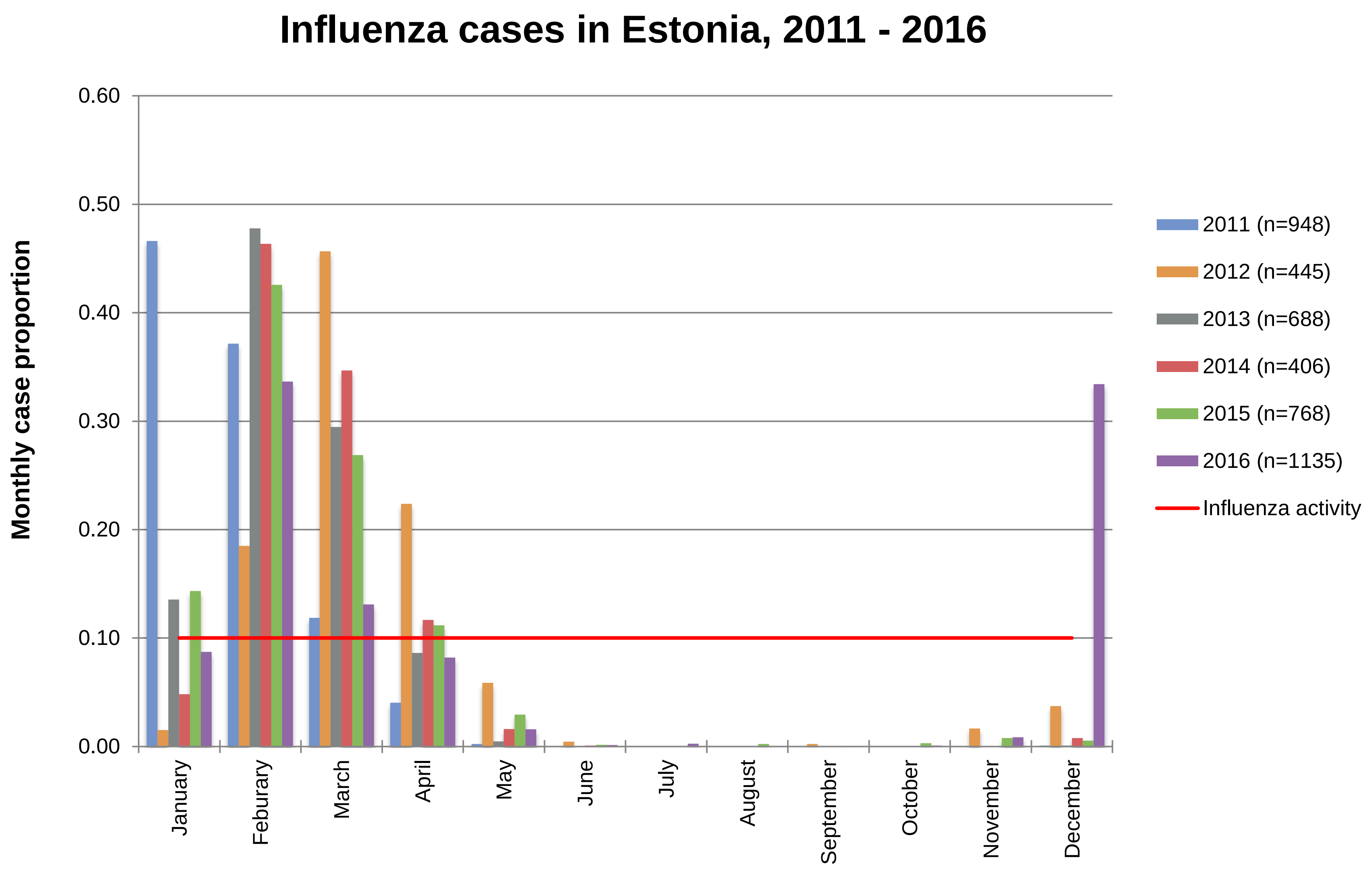

Influenza cases in Estonia, 2011 - 2016
Monthly case proportion
0.60
0.50
0.40
0.30
0.20
0.10
0.00
January
Feburary
March
April
May
June
July
August
September
October
November
December
2011 (n=948)
2012 (n=445)
2013 (n=688)
2014 (n=406)
2015 (n=768)
2016 (n=1135)
Influenza activity

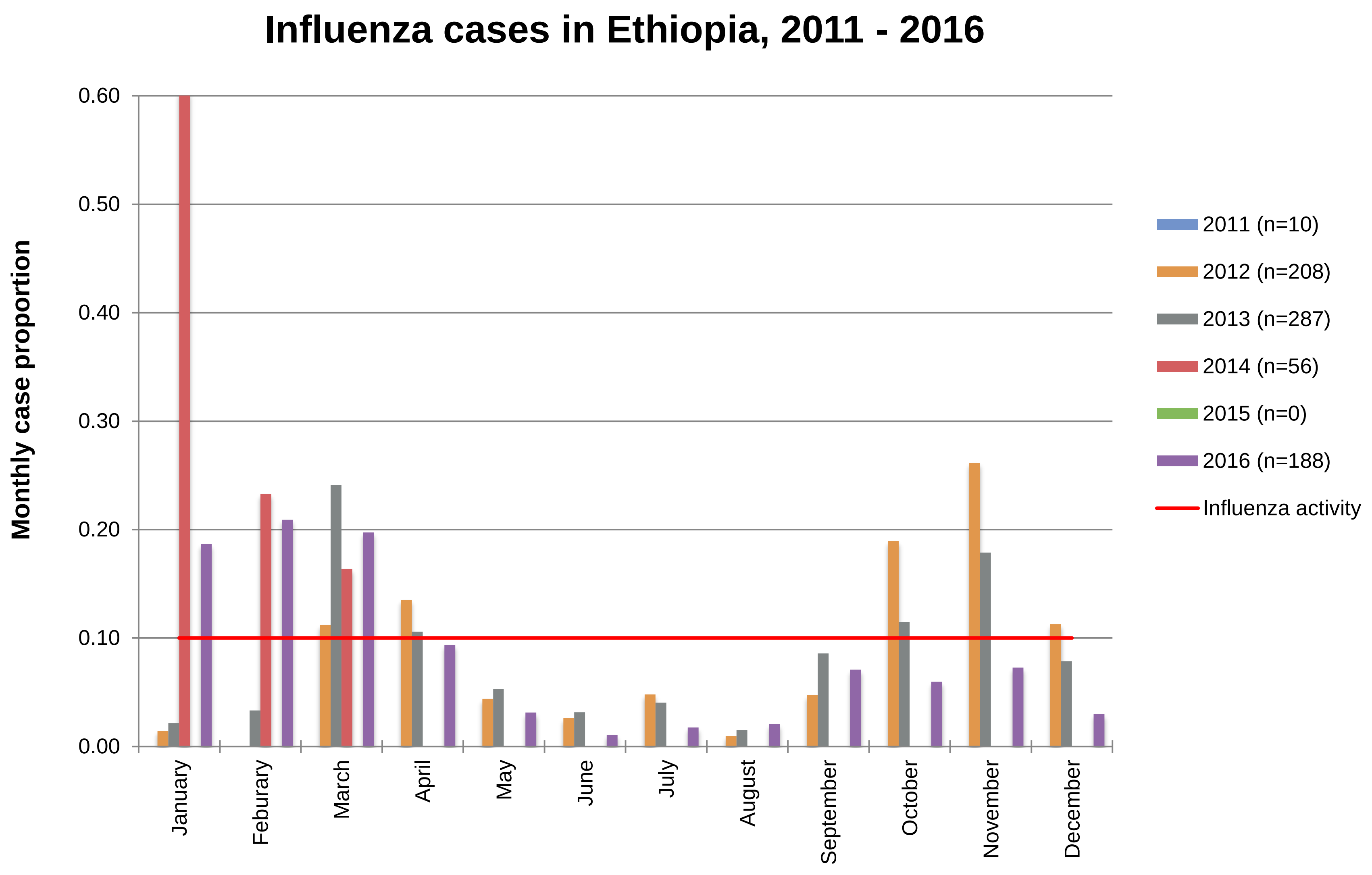
Influenza cases in Ethiopia, 2011 - 2016
Monthly case proportion
0.60
0.50
0.40
0.30
0.20
0.10
0.00
January
Feburary
March
April
May
June
July
August
September
October
November
December
2011 (n=10)
2012 (n=208)
2013 (n=287)
2014 (n=56)
2015 (n=0)
2016 (n=188)
Influenza activity

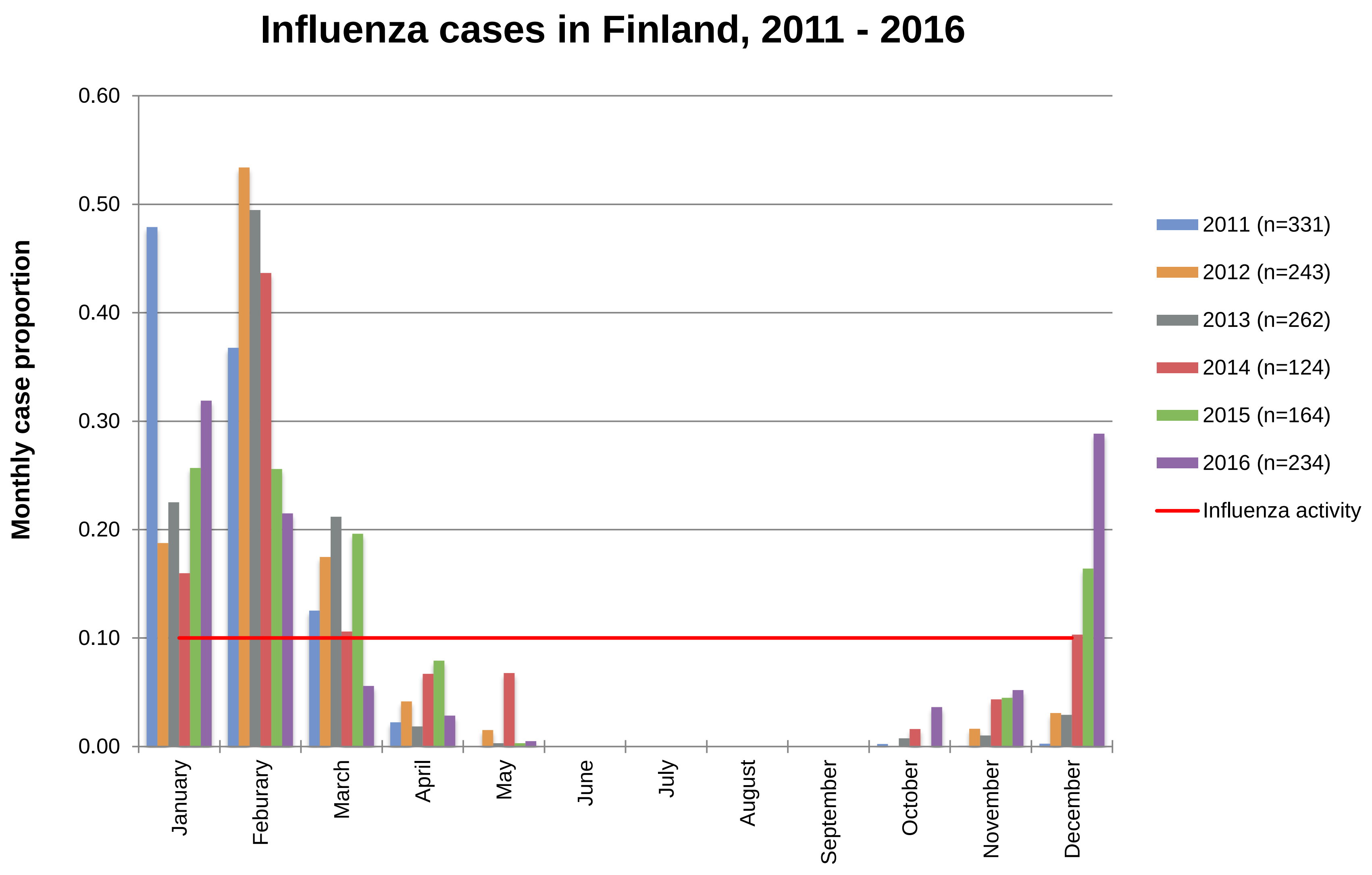

Influenza cases in Finland, 2011 - 2016
Monthly case proportion
0.60
0.50
0.40
0.30
0.20
0.10
0.00
January
Feburary
March
April
May
June
July
August
September
October
November
December
2011 (n=331)
2012 (n=243)
2013 (n=262)
2014 (n=124)
2015 (n=164)
2016 (n=234)
Influenza activity

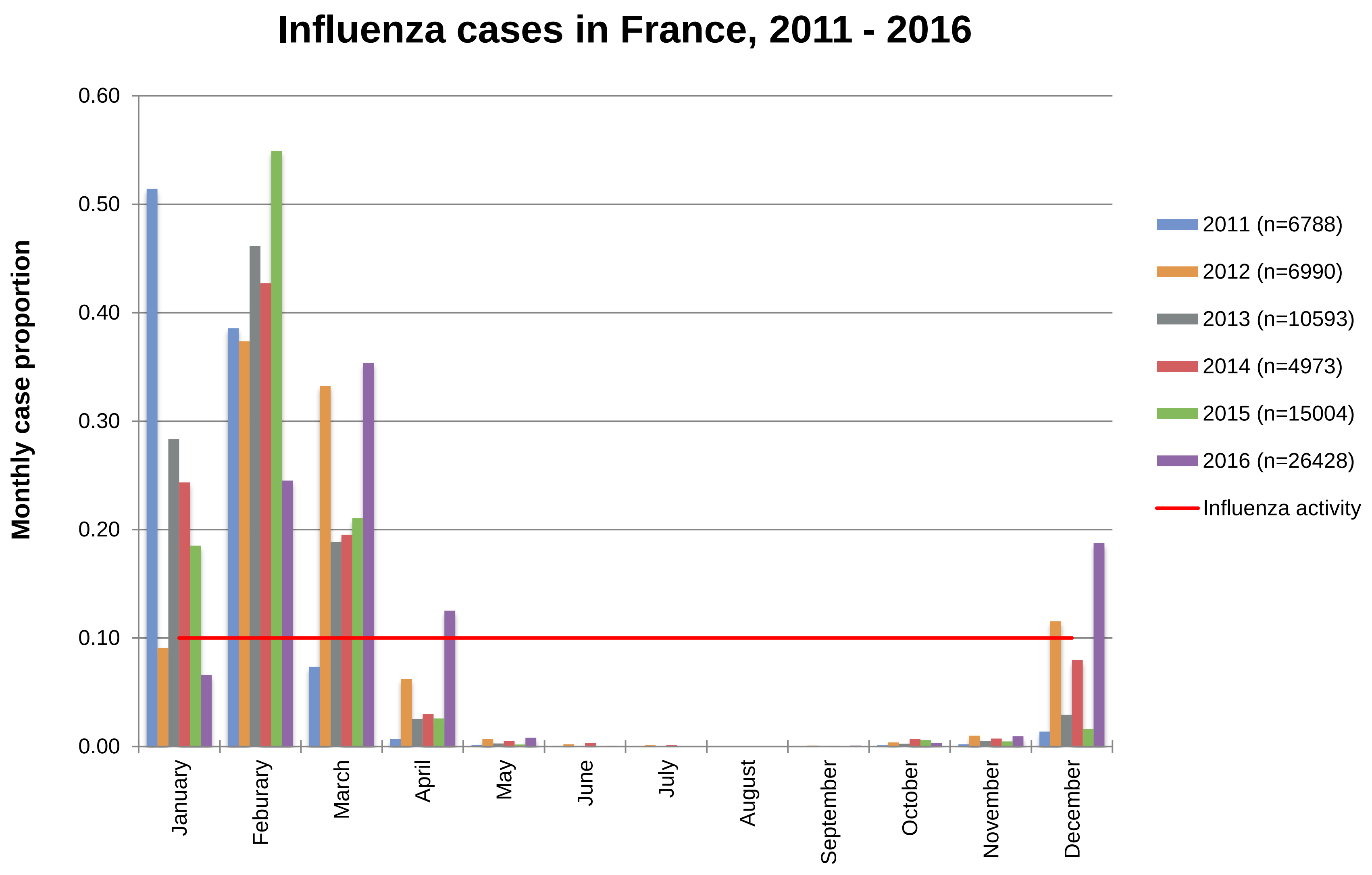

Influenza cases in France, 2011 - 2016
Monthly case proportion
0.60
0.50
0.40
0.30
0.20
0.10
0.00
January
Feburary
March
April
May
June
July
August
September
October
November
December
2011 (n=6788)
2012 (n=6990)
2013 (n=10593)
2014 (n=4973)
2015 (n=15004)
2016 (n=26428)
Influenza activity

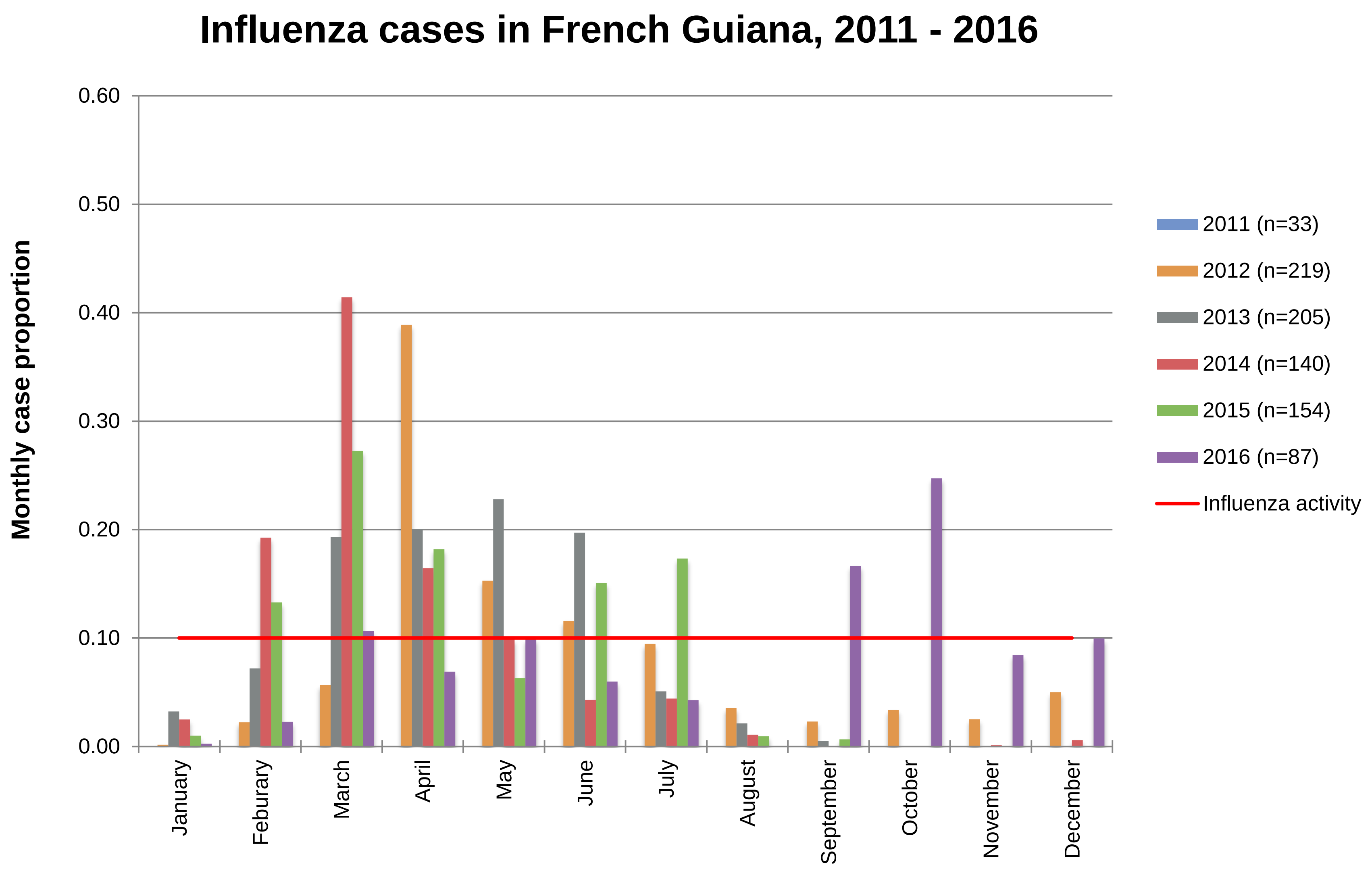
Influenza cases in French Guiana, 2011 - 2016
Monthly case proportion
0.60
0.50
0.40
0.30
0.20
0.10
0.00
January
Feburary
March
April
May
June
July
August
September
October
November
December
2011 (n=33)
2012 (n=219)
2013 (n=205)
2014 (n=140)
2015 (n=154)
2016 (n=87)
Influenza activity

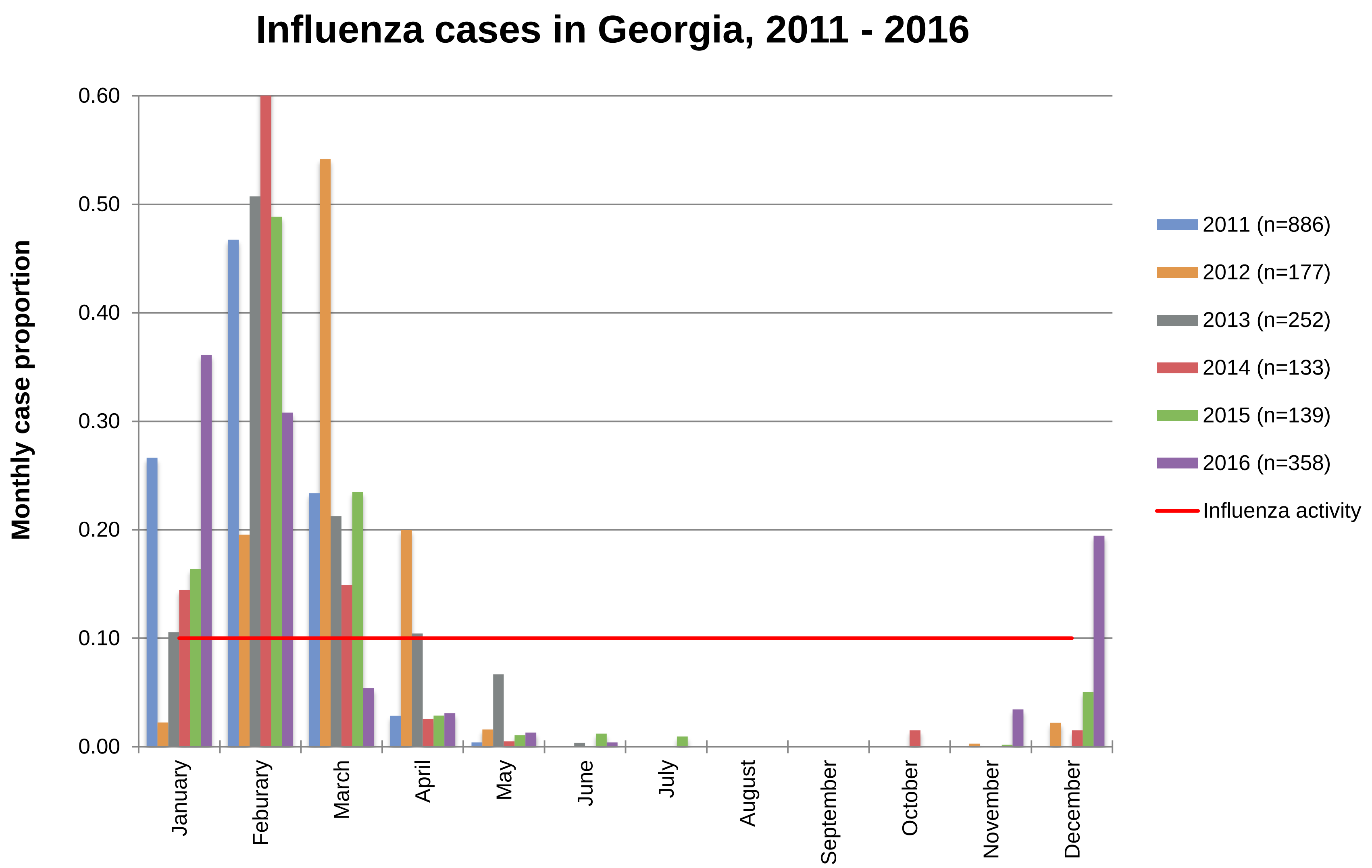

Influenza cases in Georgia, 2011 - 2016
Monthly case proportion
0.60
0.50
0.40
0.30
0.20
0.10
0.00
January
Feburary
March
April
May
June
July
August
September
October
November
December
2011 (n=886)
2012 (n=177)
2013 (n=252)
2014 (n=133)
2015 (n=139)
2016 (n=358)
Influenza activity

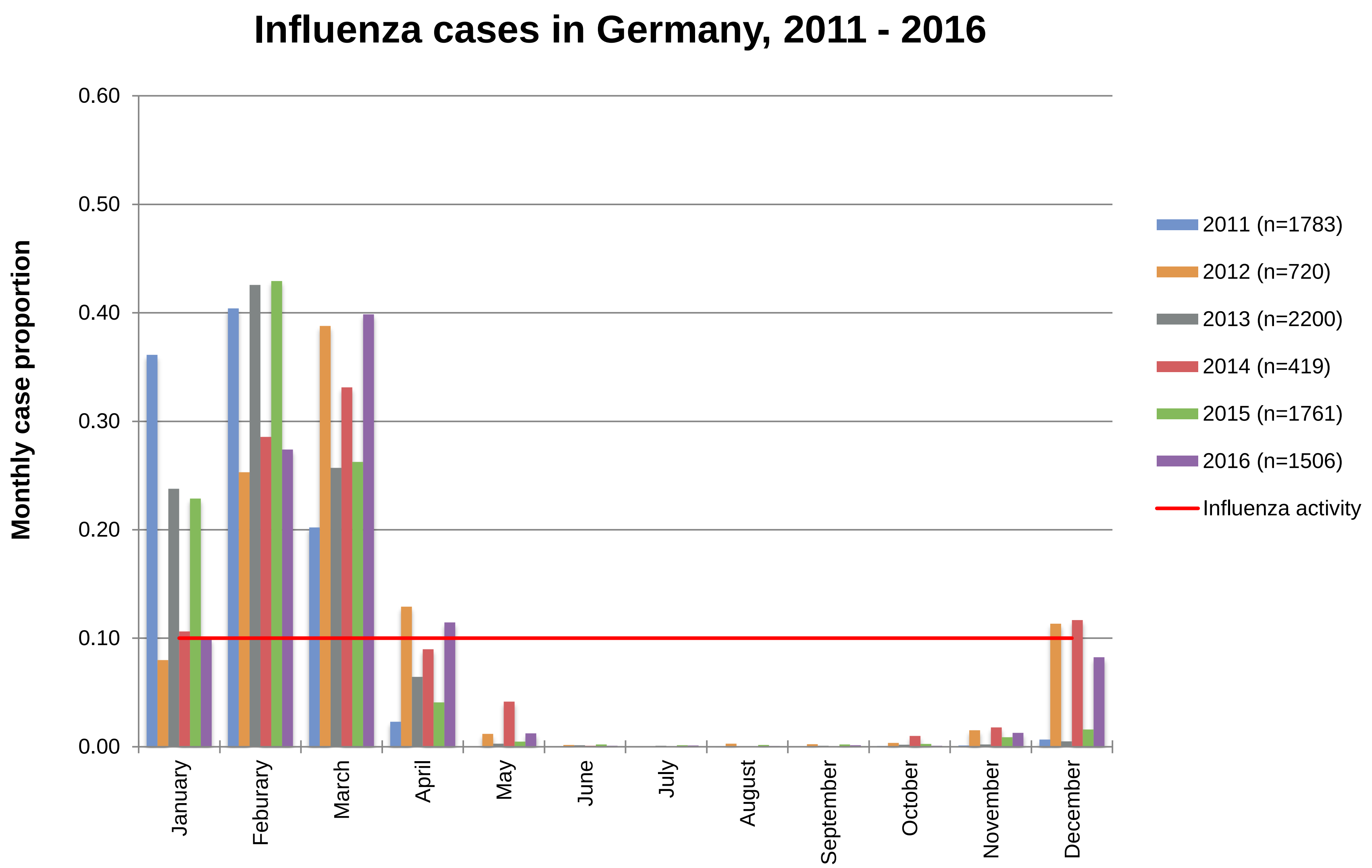

Influenza cases in Germany, 2011 - 2016
Monthly case proportion
0.60
0.50
0.40
0.30
0.20
0.10
0.00
January
Feburary
March
April
May
June
July
August
September
October
November
December
2011 (n=1783)
2012 (n=720)
2013 (n=2200)
2014 (n=419)
2015 (n=1761)
2016 (n=1506)
Influenza activity

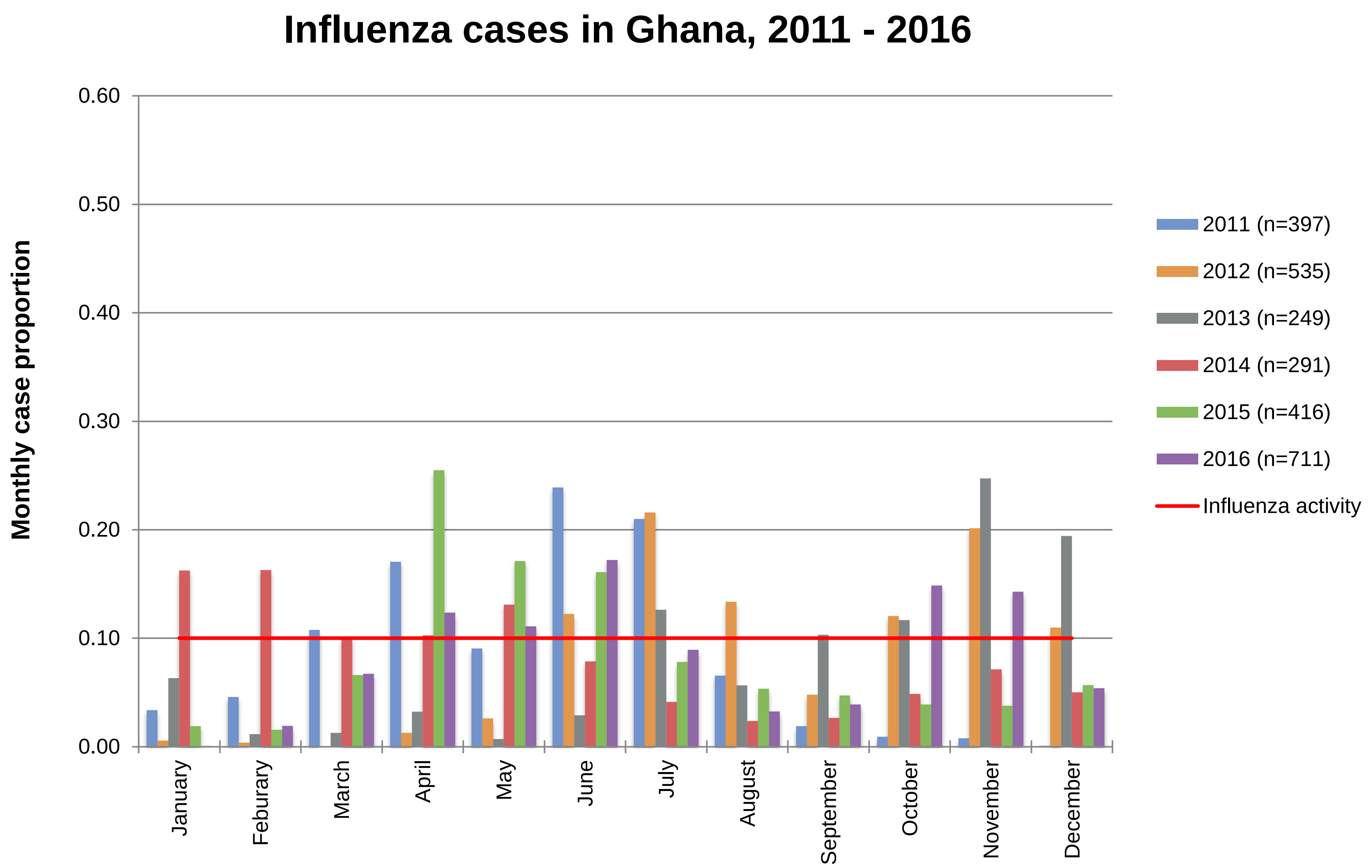

Influenza cases in Ghana, 2011 - 2016
Monthly case proportion
0.60
0.50
0.40
0.30
0.20
0.10
0.00
January
Feburary
March
April
May
June
July
August
September
October
November
December
2011 (n=397)
2012 (n=535)
2013 (n=249)
2014 (n=291)
2015 (n=416)
2016 (n=711)
Influenza activity

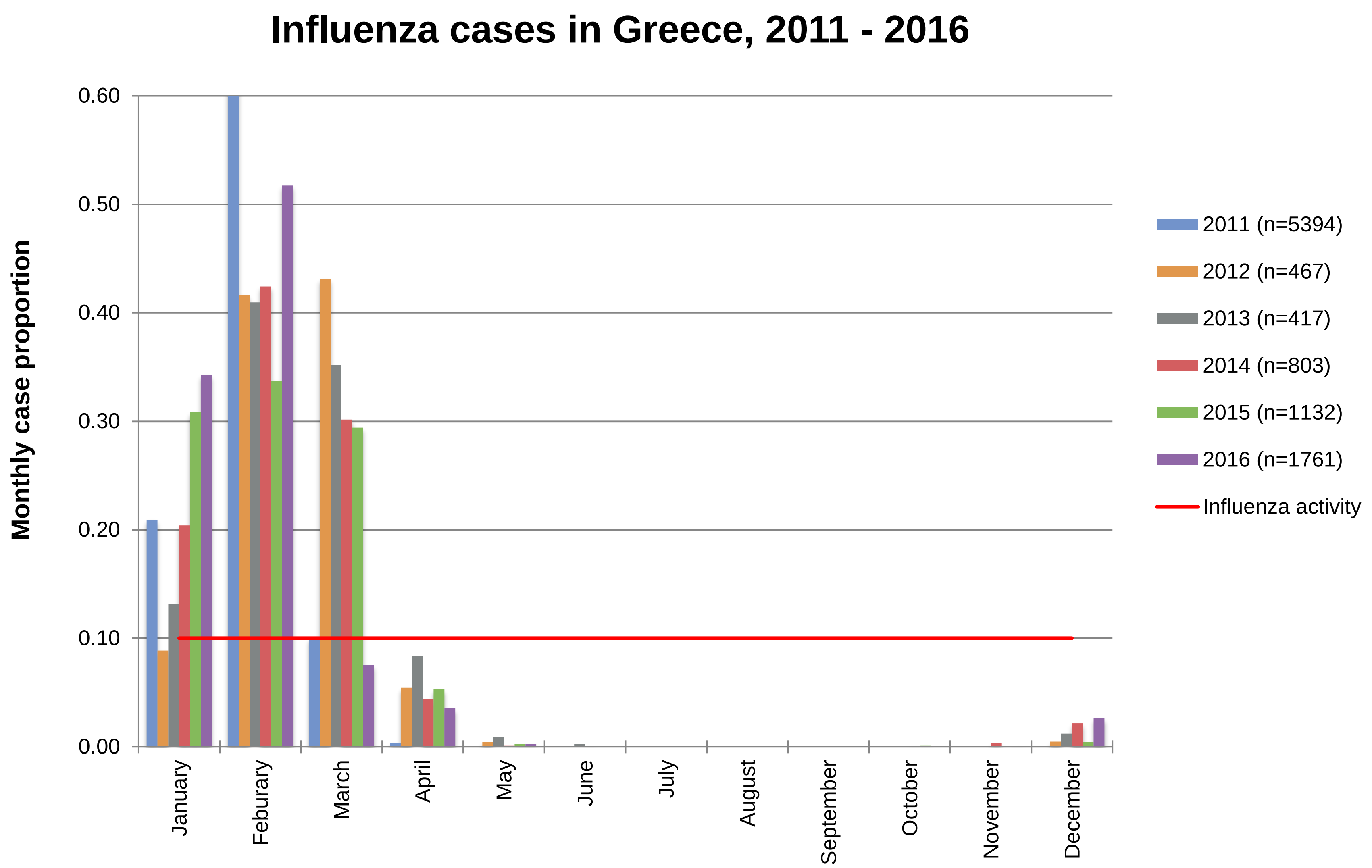

Influenza cases in Greece, 2011 - 2016
Monthly case proportion
0.60
0.50
0.40
0.30
0.20
0.10
0.00
January
Feburary
March
April
May
June
July
August
September
October
November
December
2011 (n=5394)
2012 (n=467)
2013 (n=417)
2014 (n=803)
2015 (n=1132)
2016 (n=1761)
Influenza activity

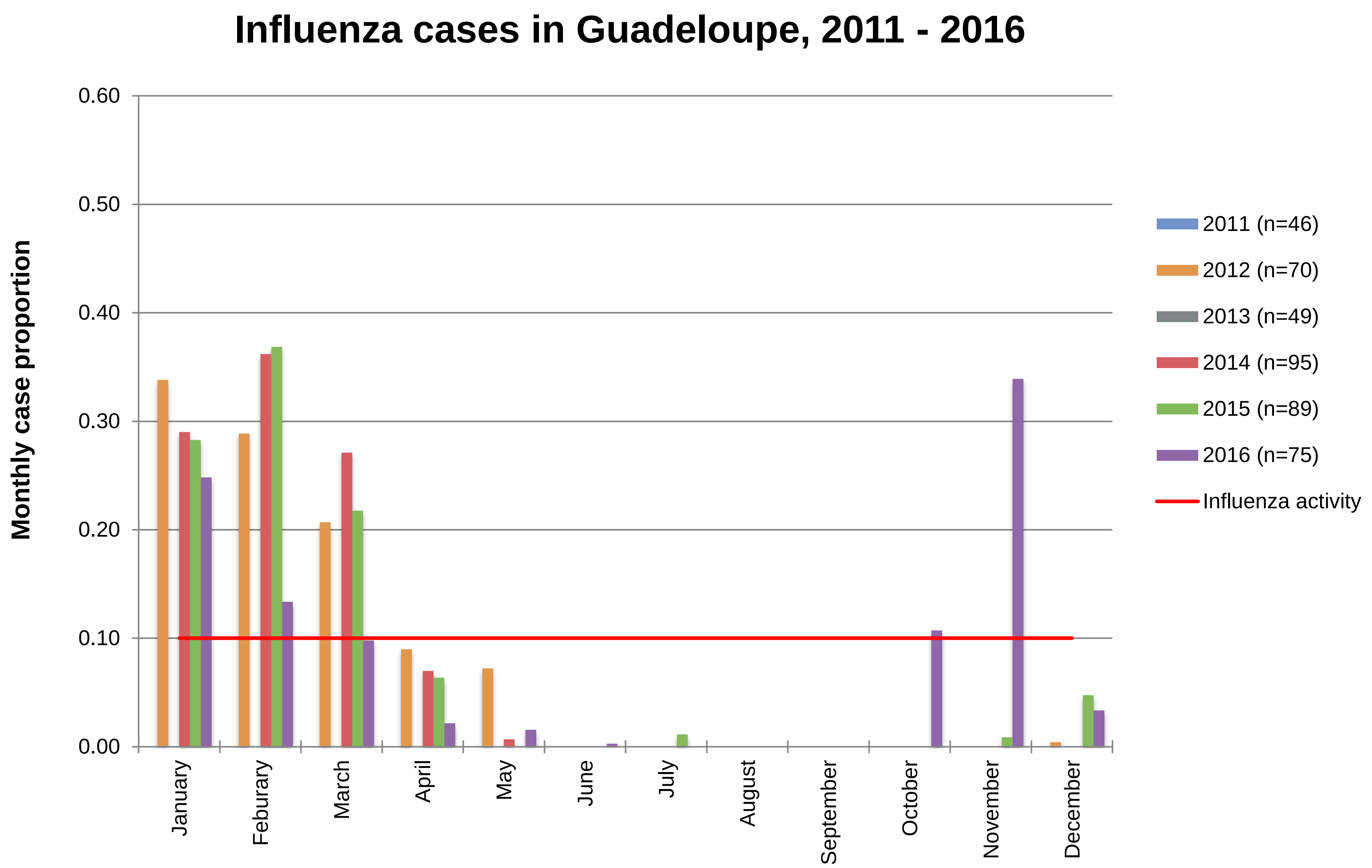
Influenza cases in Guadeloupe, 2011 - 2016
Monthly case proportion
0.60
0.50
0.40
0.30
0.20
0.10
0.00
January
Feburary
March
April
May
June
July
August
September
October
November
December
2011 (n=46)
2012 (n=70)
2013 (n=49)
2014 (n=95)
2015 (n=89)
2016 (n=75)
Influenza activity

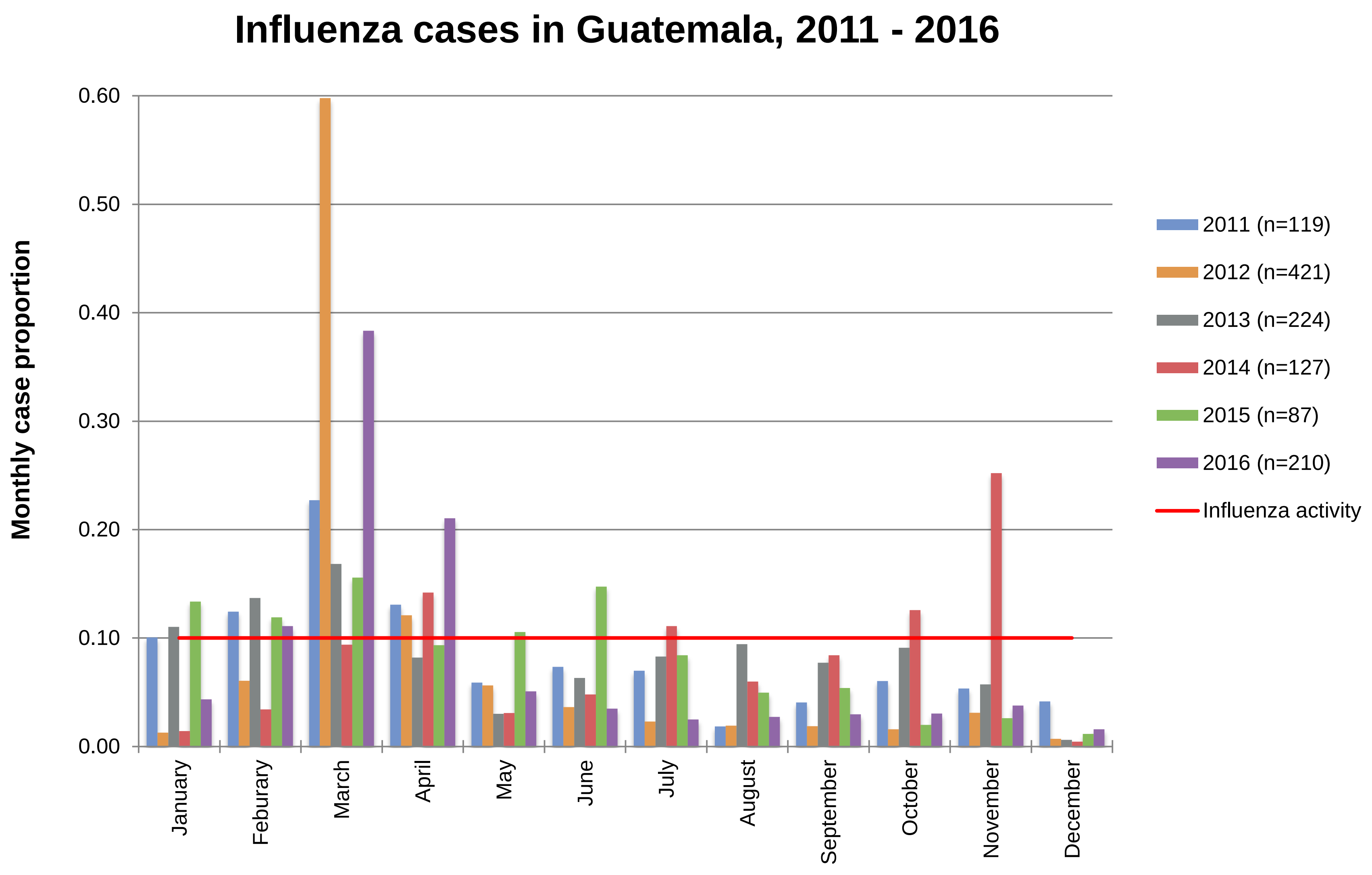
Influenza cases in Guatemala, 2011 - 2016
Monthly case proportion
0.60
0.50
0.40
0.30
0.20
0.10
0.00
January
Feburary
March
April
May
June
July
August
September
October
November
December
2011 (n=119)
2012 (n=421)
2013 (n=224)
2014 (n=127)
2015 (n=87)
2016 (n=210)
Influenza activity

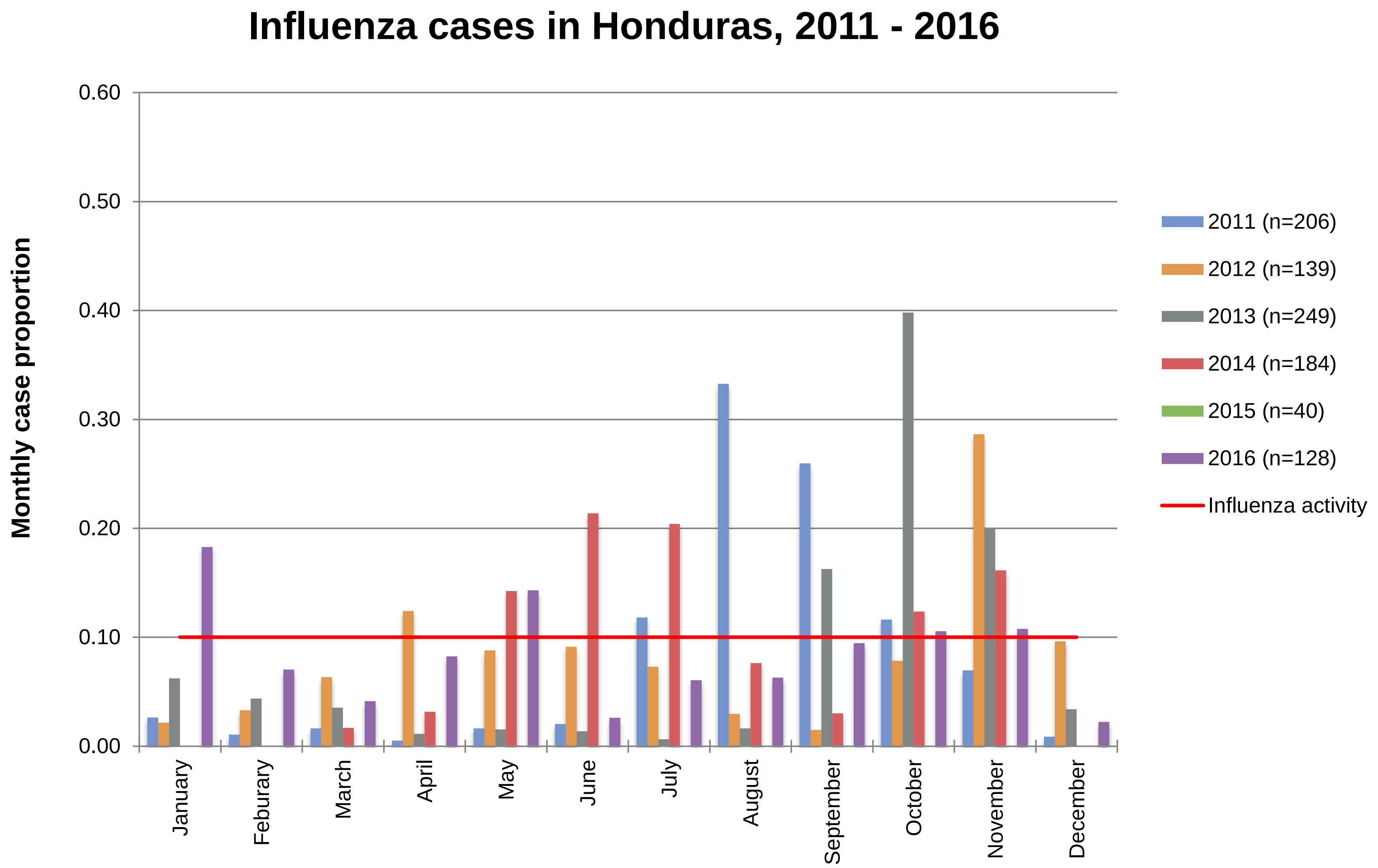

Influenza cases in Honduras, 2011 - 2016
Monthly case proportion
0.60
0.50
0.40
0.30
0.20
0.10
0.00
January
Feburary
March
April
May
June
July
August
September
October
November
December
2011 (n=206)
2012 (n=139)
2013 (n=249)
2014 (n=184)
2015 (n=40)
2016 (n=128)
Influenza activity

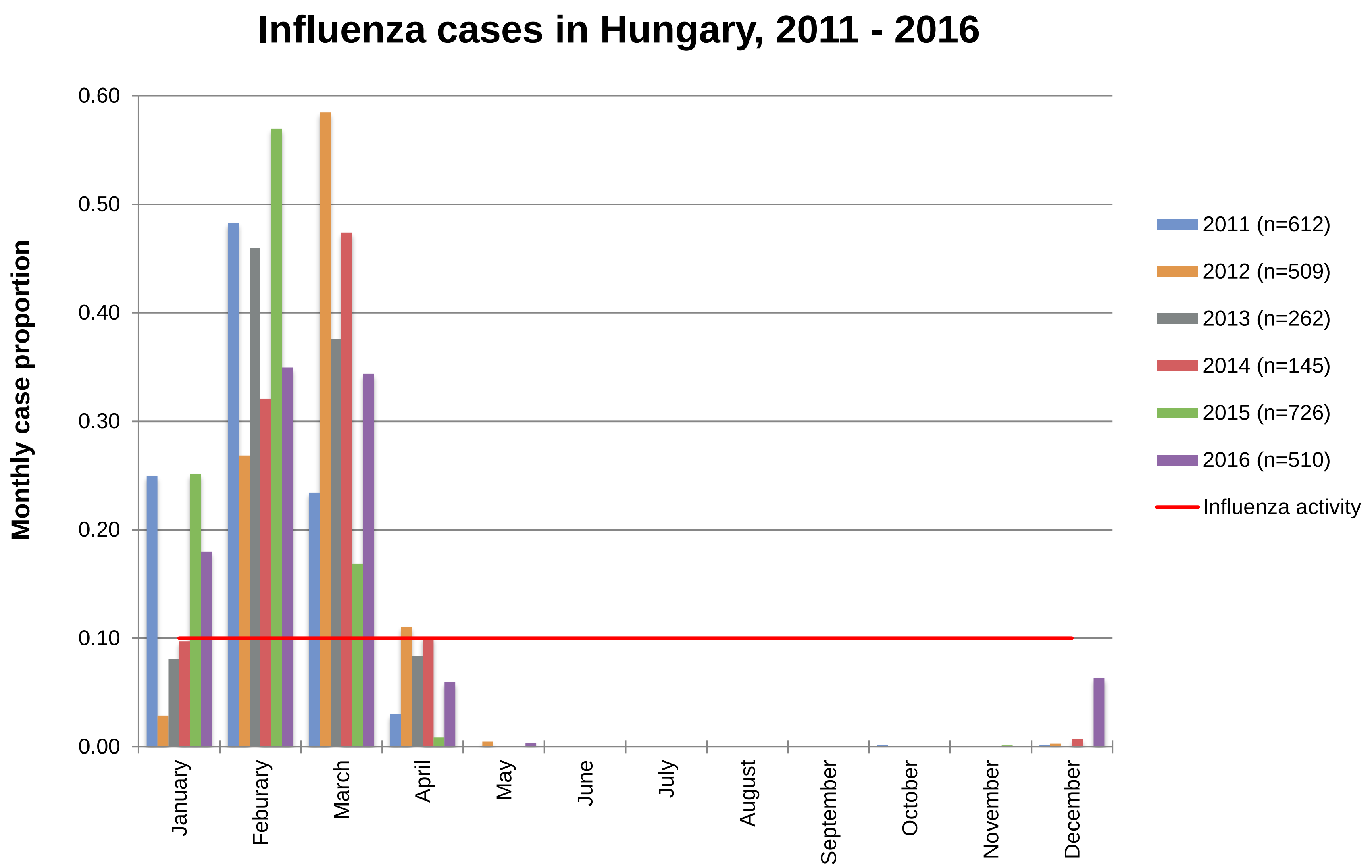

Influenza cases in Hungary, 2011 - 2016
Monthly case proportion
0.60
0.50
0.40
0.30
0.20
0.10
0.00
January
Feburary
March
April
May
June
July
August
September
October
November
December
2011 (n=612)
2012 (n=509)
2013 (n=262)
2014 (n=145)
2015 (n=726)
2016 (n=510)
Influenza activity

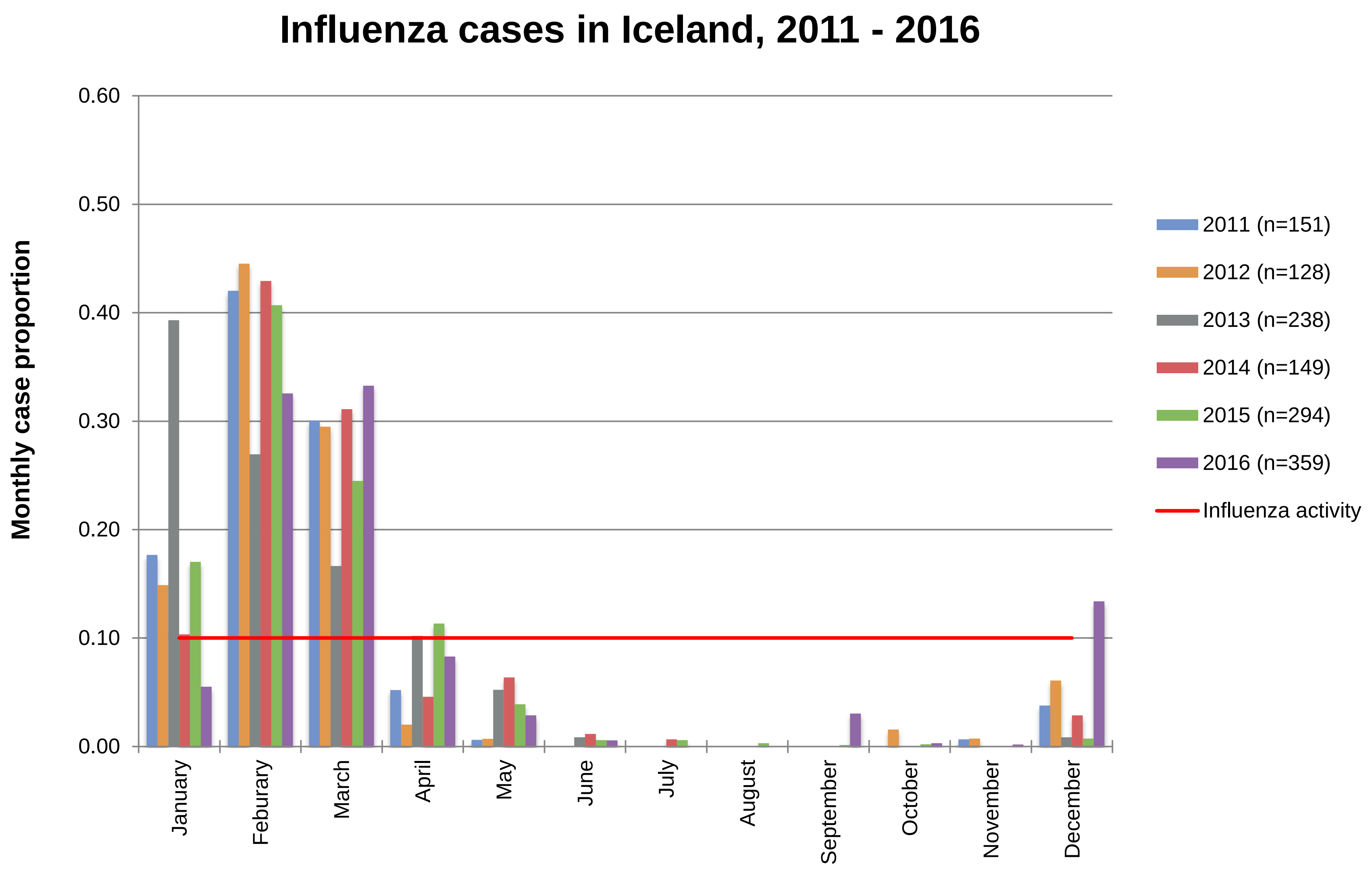

Influenza cases in Iceland, 2011 - 2016
Monthly case proportion
0.60
0.50
0.40
0.30
0.20
0.10
0.00
January
Feburary
March
April
May
June
July
August
September
October
November
December
2011 (n=151)
2012 (n=128)
2013 (n=238)
2014 (n=149)
2015 (n=294)
2016 (n=359)
Influenza activity

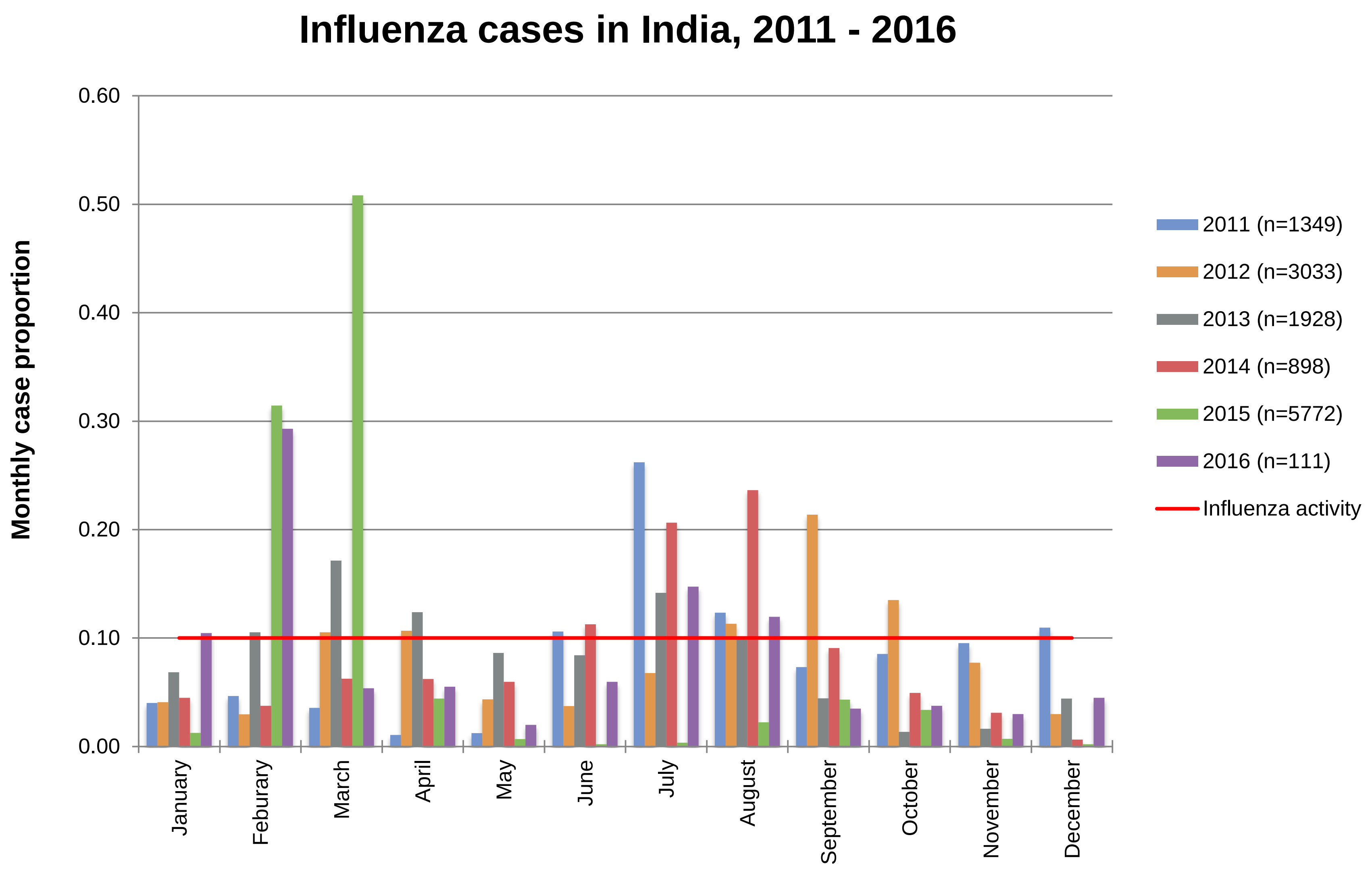
Influenza cases in India, 2011 - 2016
Monthly case proportion
0.60
0.50
0.40
0.30
0.20
0.10
0.00
January
Feburary
March
April
May
June
July
August
September
October
November
December
2011 (n=1349)
2012 (n=3033)
2013 (n=1928)
2014 (n=898)
2015 (n=5772)
2016 (n=111)
Influenza activity

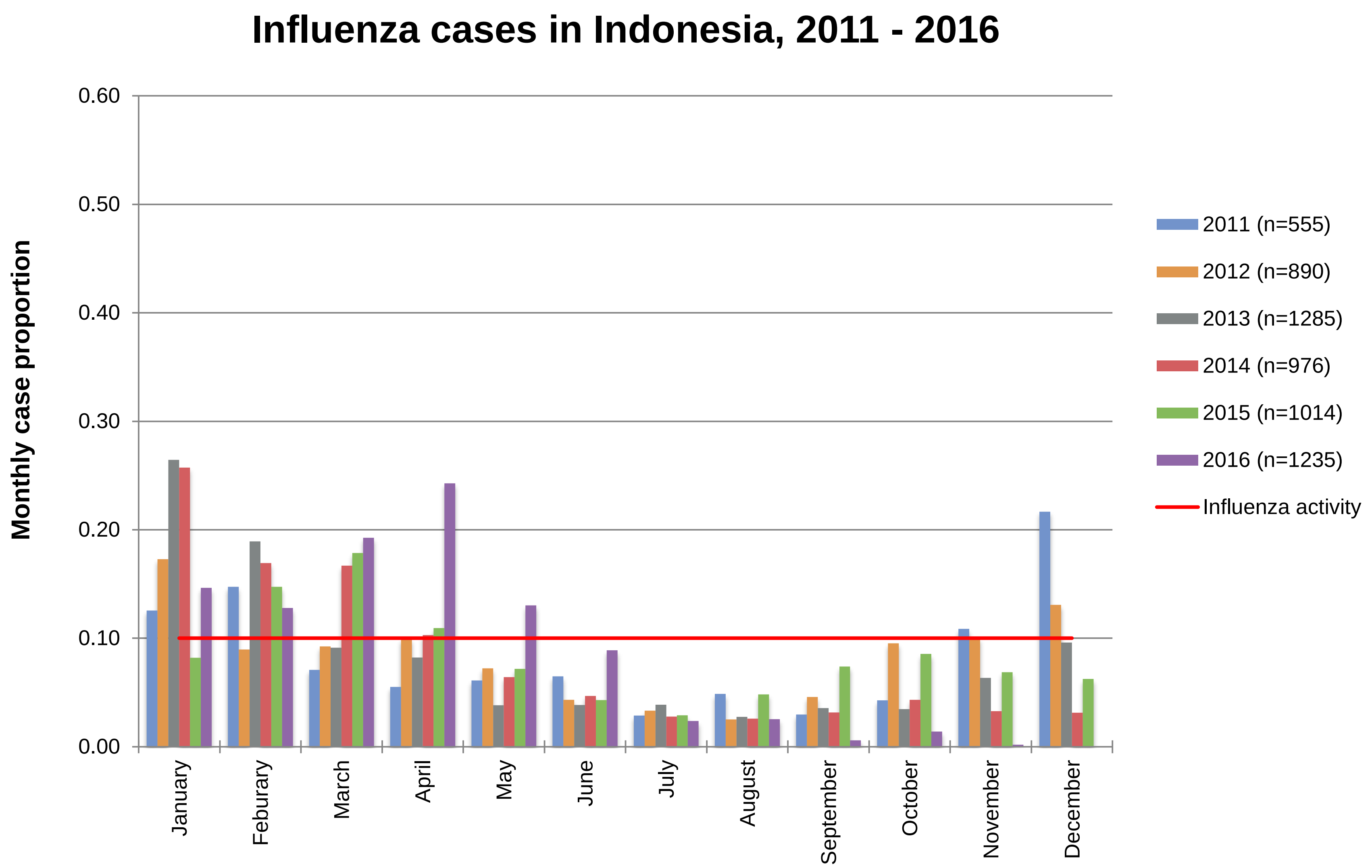

Influenza cases in Indonesia, 2011 - 2016
Monthly case proportion
0.60
0.50
0.40
0.30
0.20
0.10
0.00
January
Feburary
March
April
May
June
July
August
September
October
November
December
2011 (n=555)
2012 (n=890)
2013 (n=1285)
2014 (n=976)
2015 (n=1014)
2016 (n=1235)
Influenza activity

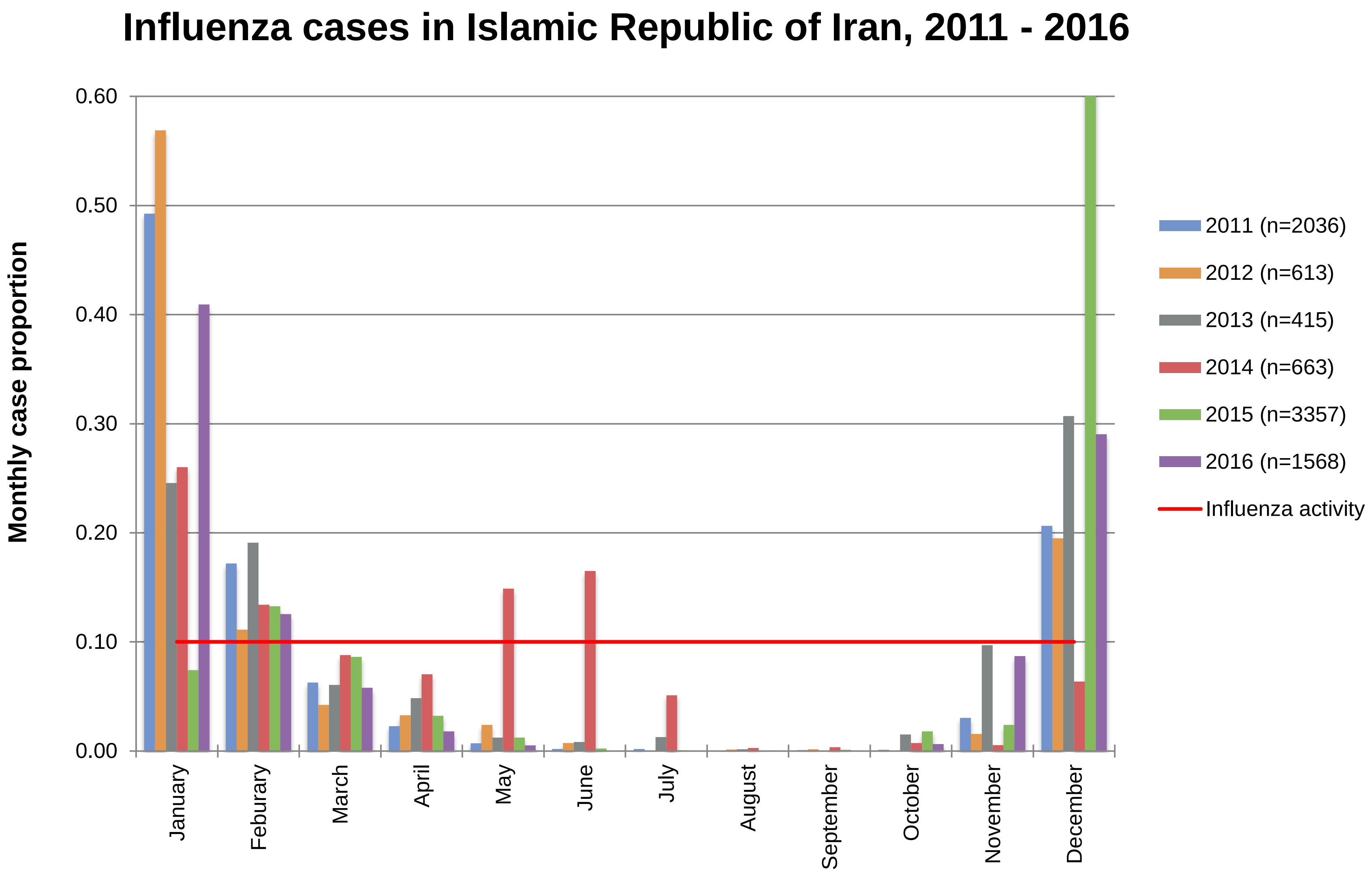
Influenza cases in Islamic Republic of Iran, 2011 - 2016
Monthly case proportion
0.60
0.50
0.40
0.30
0.20
0.10
0.00
January
Feburary
March
April
May
June
July
August
September
October
November
December
2011 (n=2036)
2012 (n=613)
2013 (n=415)
2014 (n=663)
2015 (n=3357)
2016 (n=1568)
Influenza activity

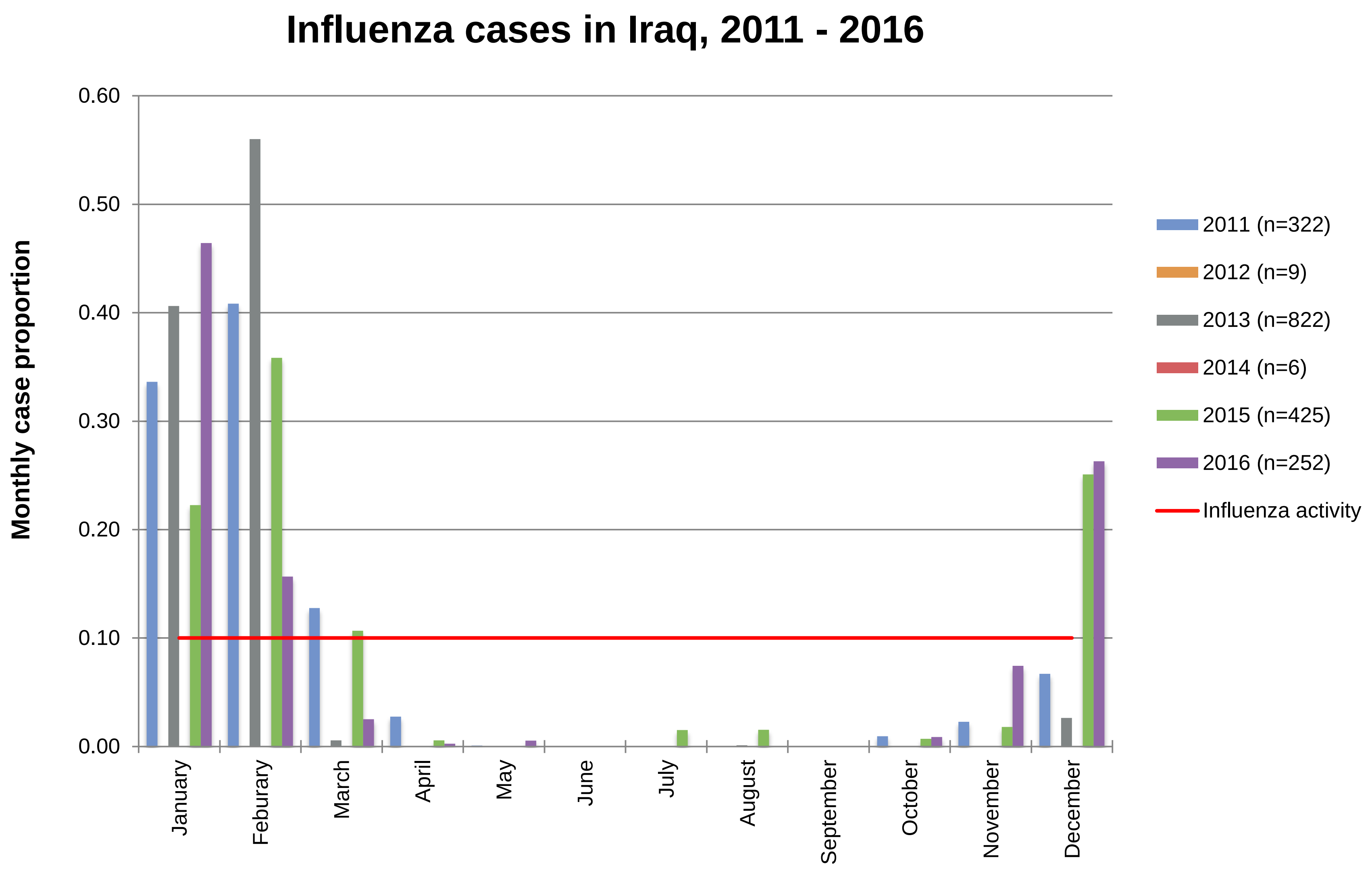

Influenza cases in Iraq, 2011 - 2016
Monthly case proportion
0.60
0.50
0.40
0.30
0.20
0.10
0.00
January
Feburary
March
April
May
June
July
August
September
October
November
December
2011 (n=322)
2012 (n=9)
2013 (n=822)
2014 (n=6)
2015 (n=425)
2016 (n=252)
Influenza activity

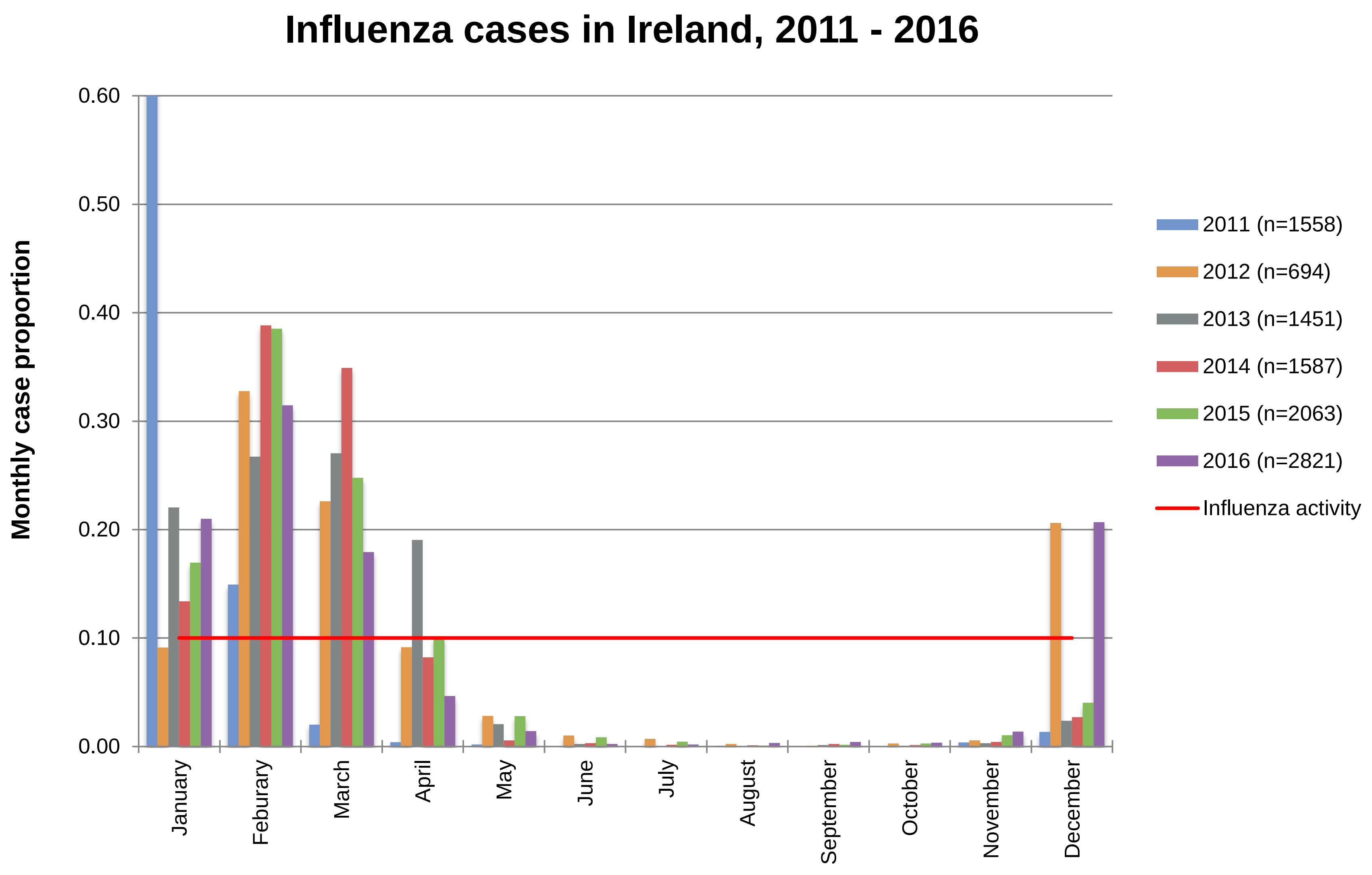

Influenza cases in Ireland, 2011 - 2016
Monthly case proportion
0.60
0.50
0.40
0.30
0.20
0.10
0.00
January
Feburary
March
April
May
June
July
August
September
October
November
December
2011 (n=1558)
2012 (n=694)
2013 (n=1451)
2014 (n=1587)
2015 (n=2063)
2016 (n=2821)
Influenza activity

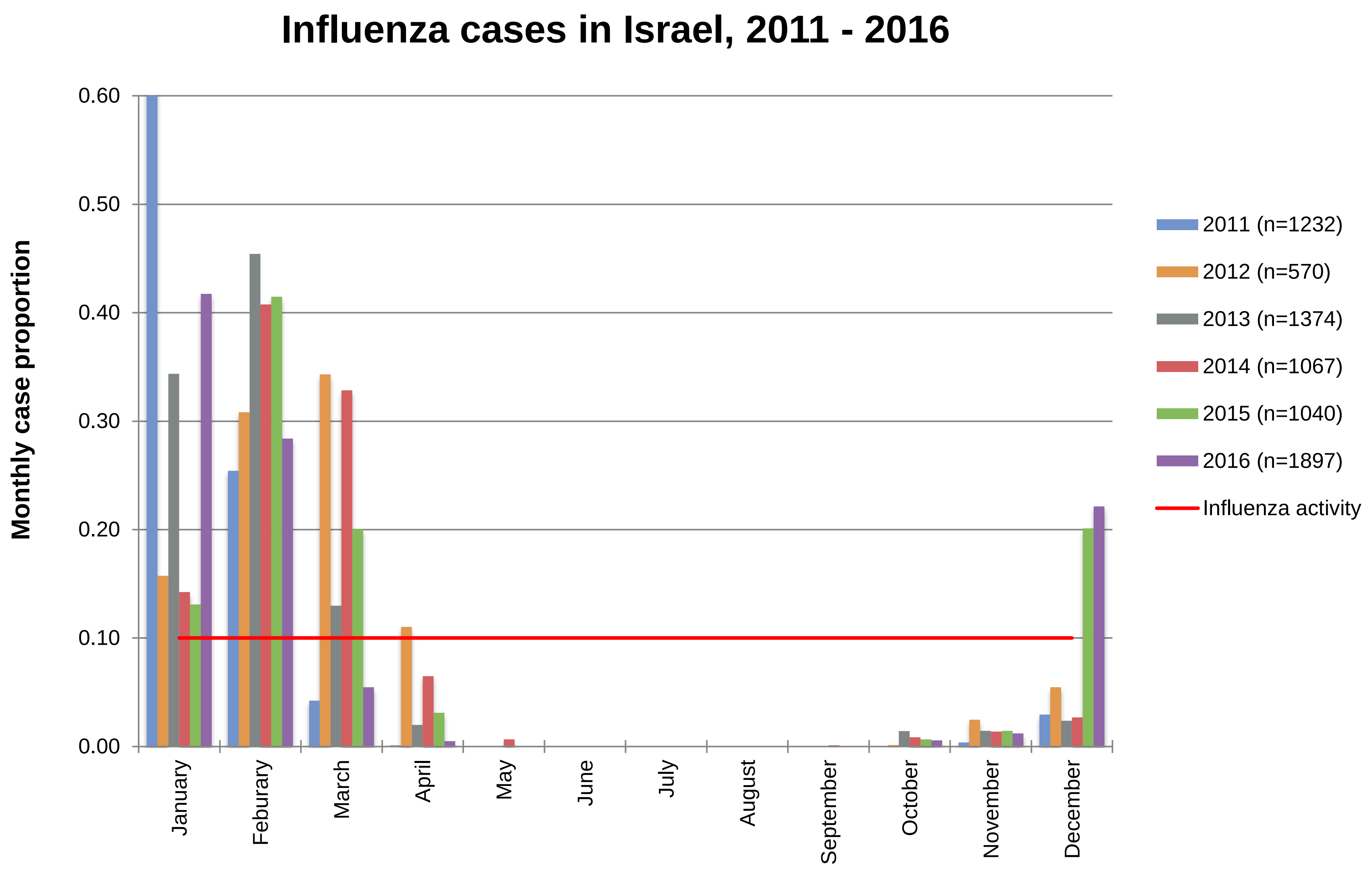

Influenza cases in Israel, 2011 - 2016
Monthly case proportion
0.60
0.50
0.40
0.30
0.20
0.10
0.00
January
Feburary
March
April
May
June
July
August
September
October
November
December
2011 (n=1232)
2012 (n=570)
2013 (n=1374)
2014 (n=1067)
2015 (n=1040)
2016 (n=1897)
Influenza activity

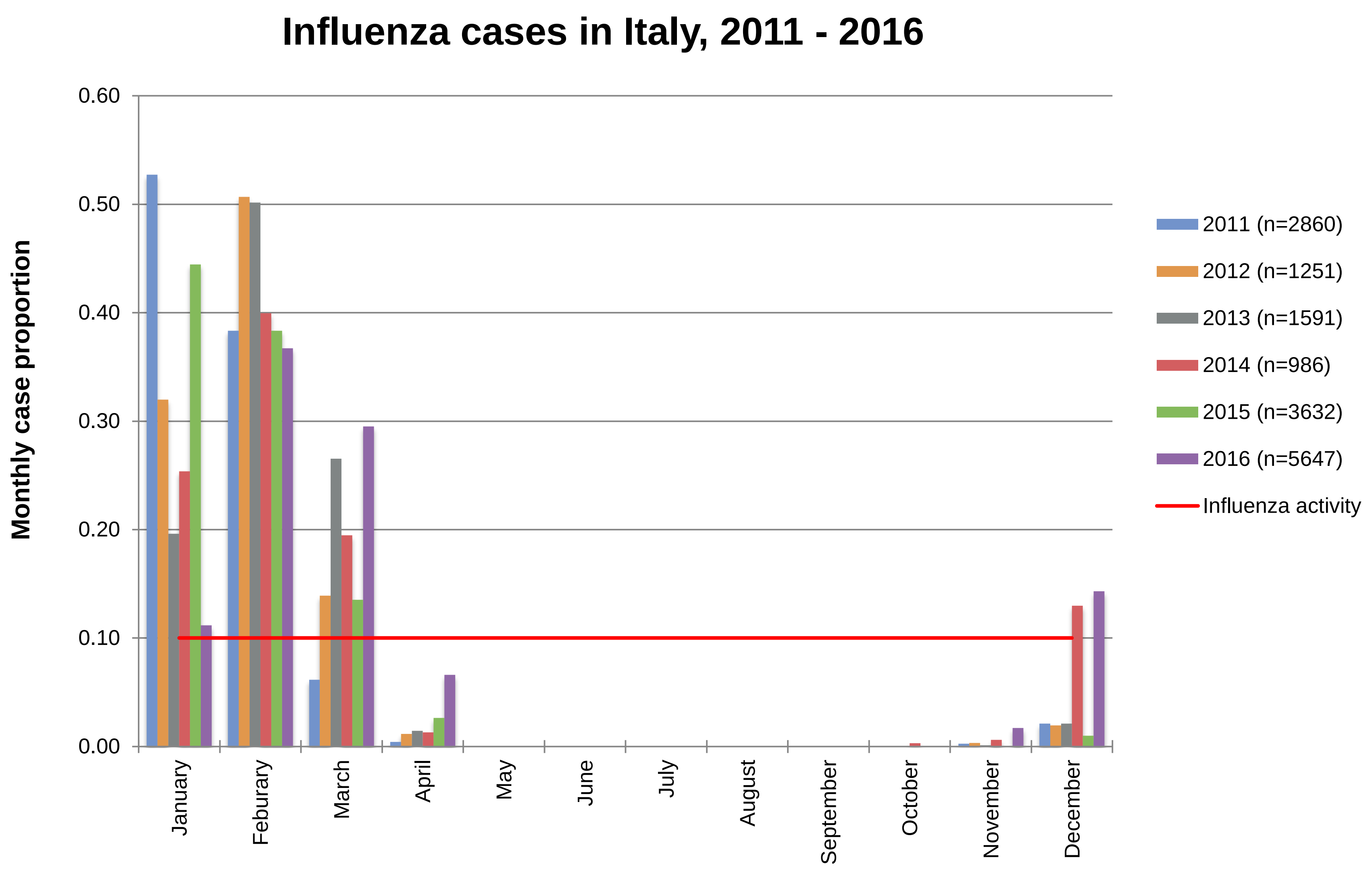

Influenza cases in Italy, 2011 - 2016
Monthly case proportion
0.60
0.50
0.40
0.30
0.20
0.10
0.00
January
Feburary
March
April
May
June
July
August
September
October
November
December
2011 (n=2860)
2012 (n=1251)
2013 (n=1591)
2014 (n=986)
2015 (n=3632)
2016 (n=5647)
Influenza activity

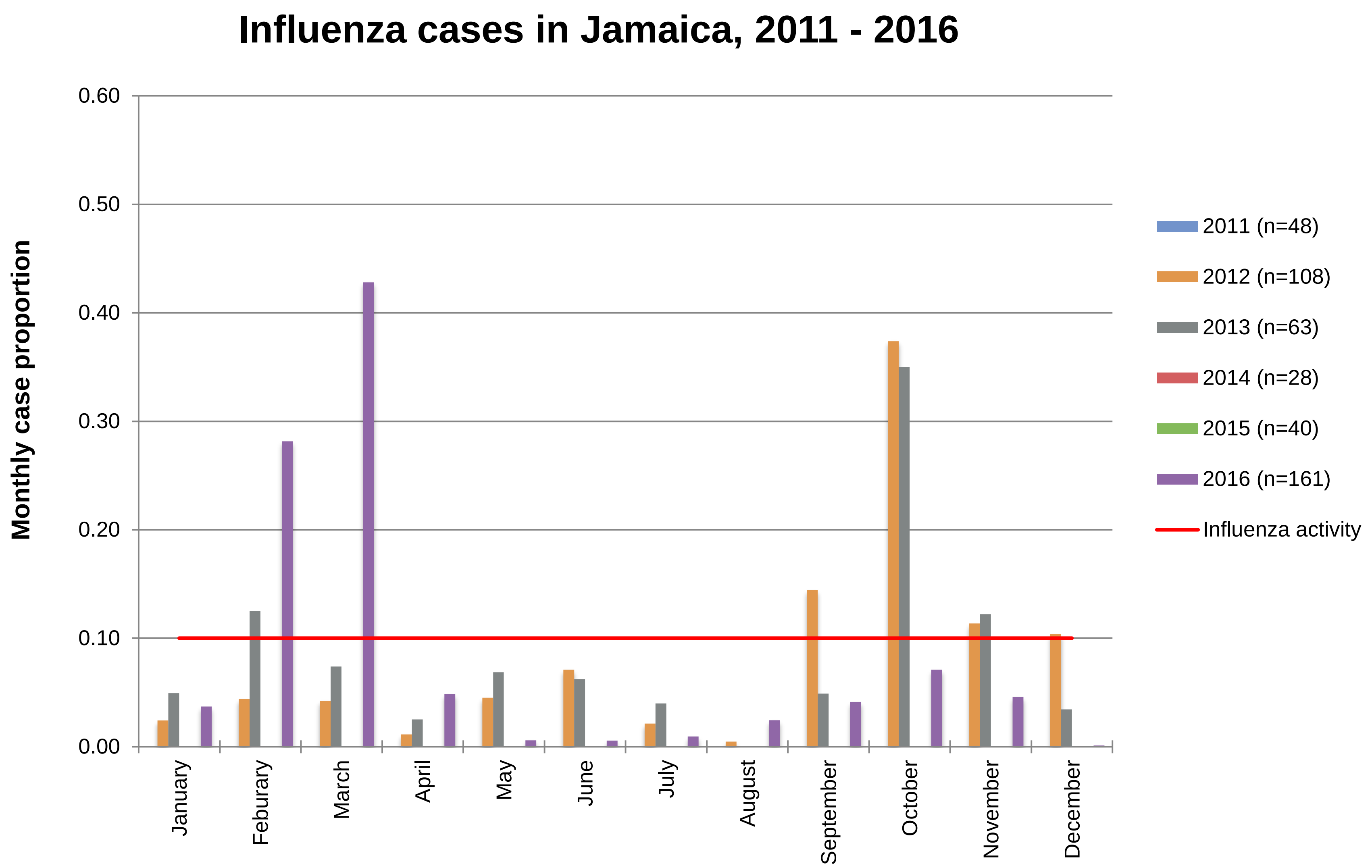
Influenza cases in Jamaica, 2011 - 2016
Monthly case proportion
0.60
0.50
0.40
0.30
0.20
0.10
0.00
January
Feburary
March
April
May
June
July
August
September
October
November
December
2011 (n=48)
2012 (n=108)
2013 (n=63)
2014 (n=28)
2015 (n=40)
2016 (n=161)
Influenza activity

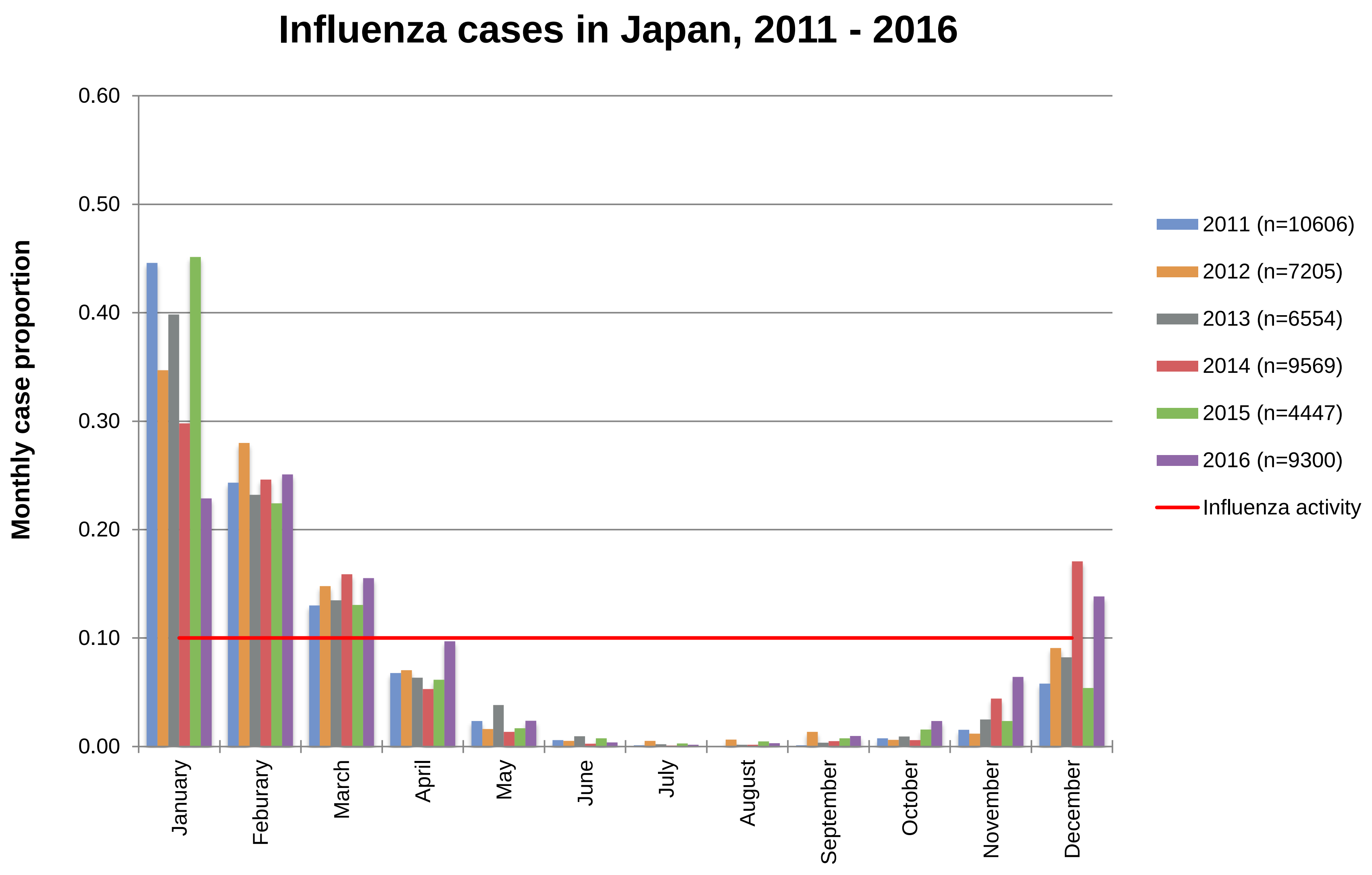
Influenza cases in Japan, 2011 - 2016
Monthly case proportion
0.60
0.50
0.40
0.30
0.20
0.10
0.00
January
Feburary
March
April
May
June
July
August
September
October
November
December
2011 (n=10606)
2012 (n=7205)
2013 (n=6554)
2014 (n=9569)
2015 (n=4447)
2016 (n=9300)
Influenza activity

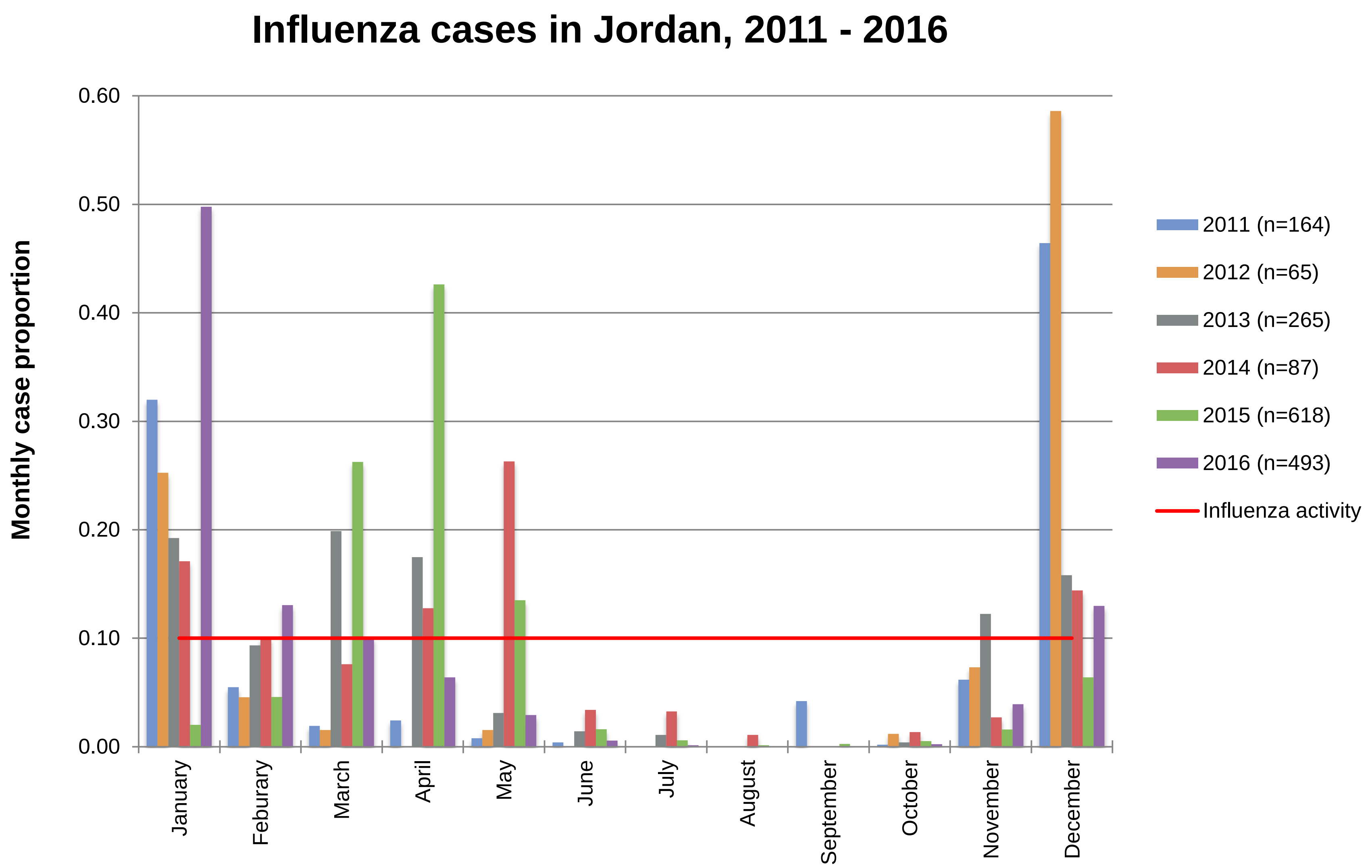

Influenza cases in Jordan, 2011 - 2016
Monthly case proportion
0.60
0.50
0.40
0.30
0.20
0.10
0.00
January
Feburary
March
April
May
June
July
August
September
October
November
December
2011 (n=164)
2012 (n=65)
2013 (n=265)
2014 (n=87)
2015 (n=618)
2016 (n=493)
Influenza activity

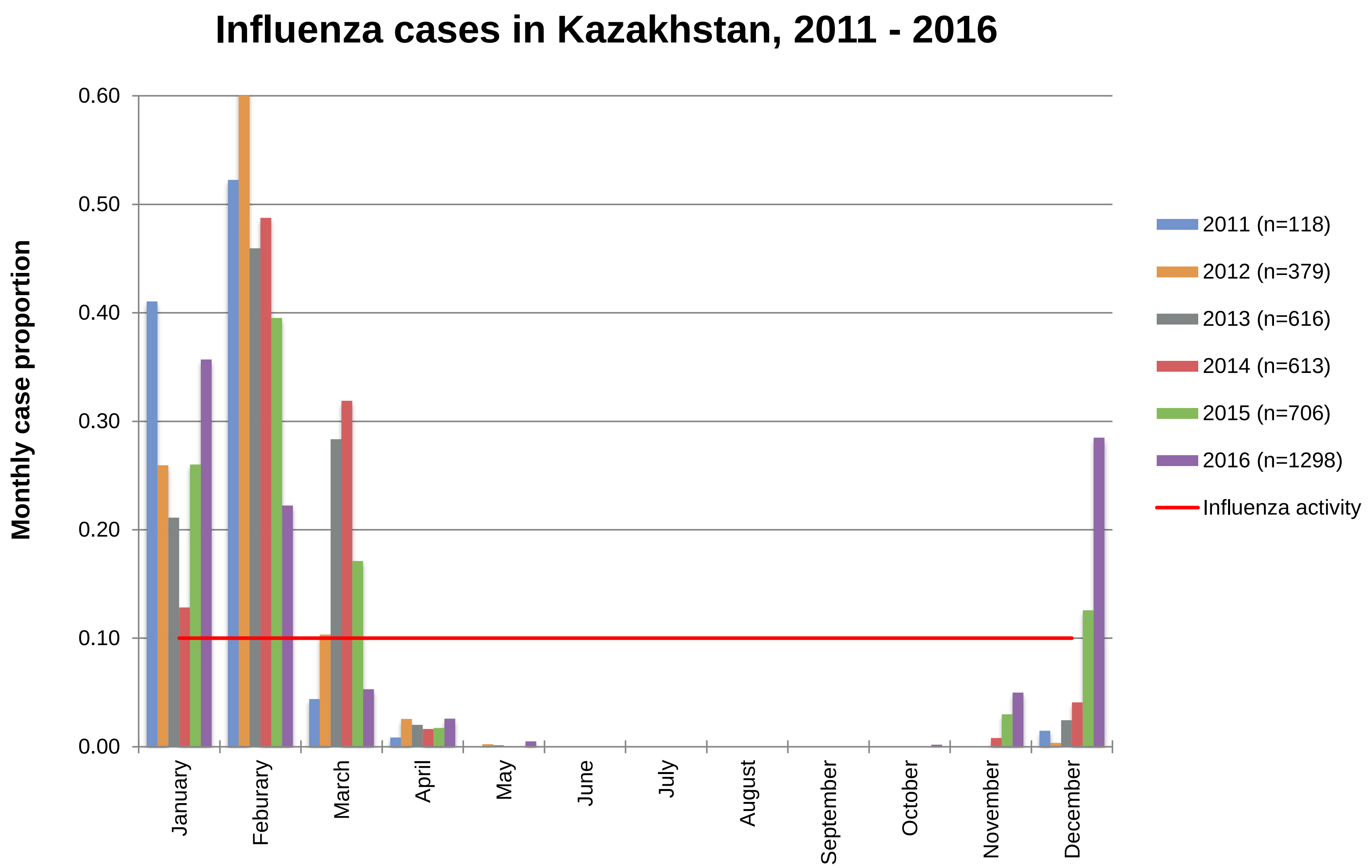

Influenza cases in Kazakhstan, 2011 - 2016
Monthly case proportion
0.60
0.50
0.40
0.30
0.20
0.10
0.00
January
Feburary
March
April
May
June
July
August
September
October
November
December
2011 (n=118)
2012 (n=379)
2013 (n=616)
2014 (n=613)
2015 (n=706)
2016 (n=1298)
Influenza activity

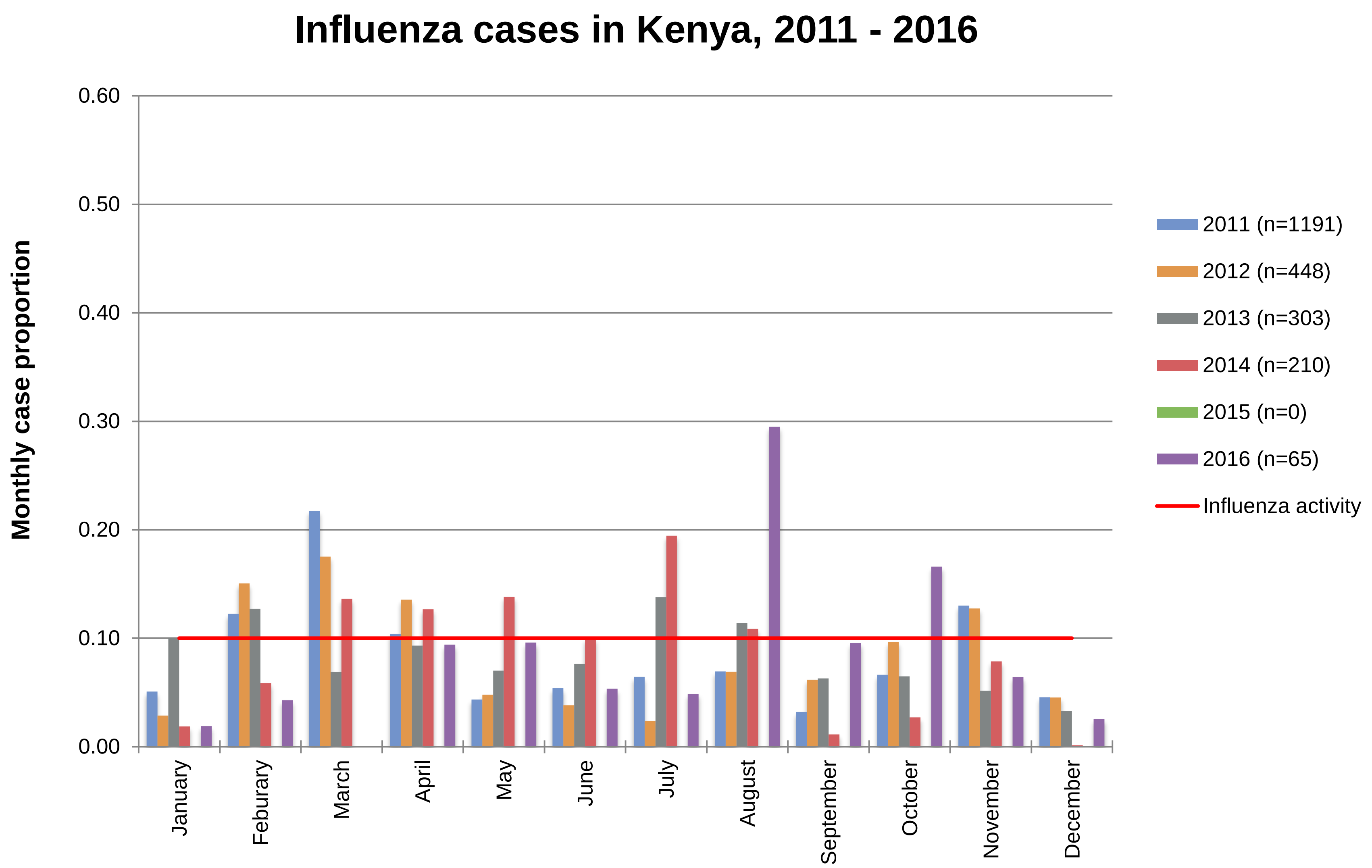
Influenza cases in Kenya, 2011 - 2016
Monthly case proportion
0.60
0.50
0.40
0.30
0.20
0.10
0.00
January
Feburary
March
April
May
June
July
August
September
October
November
December
2011 (n=1191)
2012 (n=448)
2013 (n=303)
2014 (n=210)
2015 (n=0)
2016 (n=65)
Influenza activity

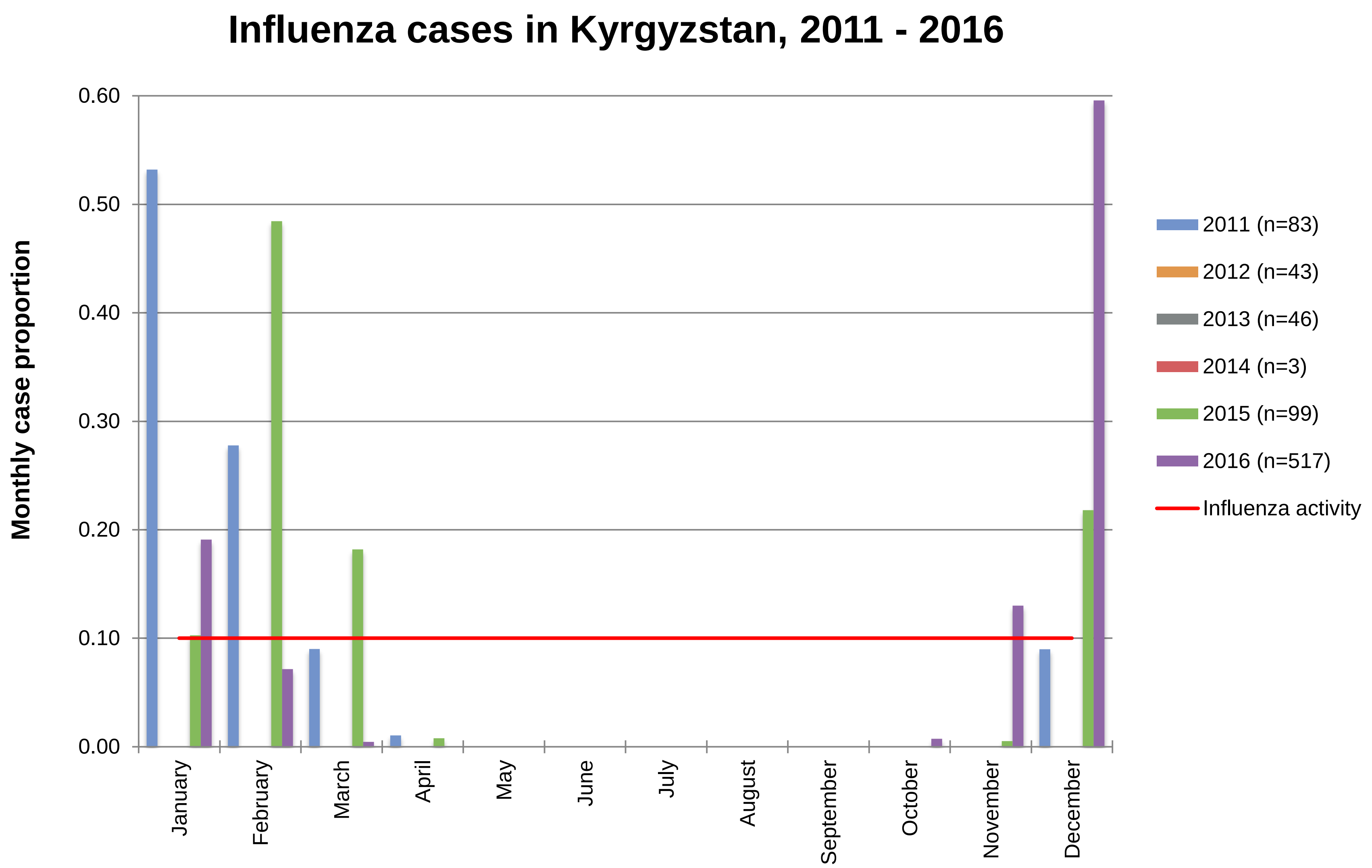

Influenza cases in Kyrgyzstan, 2011 - 2016
Monthly case proportion
0.60
0.50
0.40
0.30
0.20
0.10
0.00
January
February
March
April
May
June
July
August
September
October
November
December
2011 (n=83)
2012 (n=43)
2013 (n=46)
2014 (n=3)
2015 (n=99)
2016 (n=517)
Influenza activity

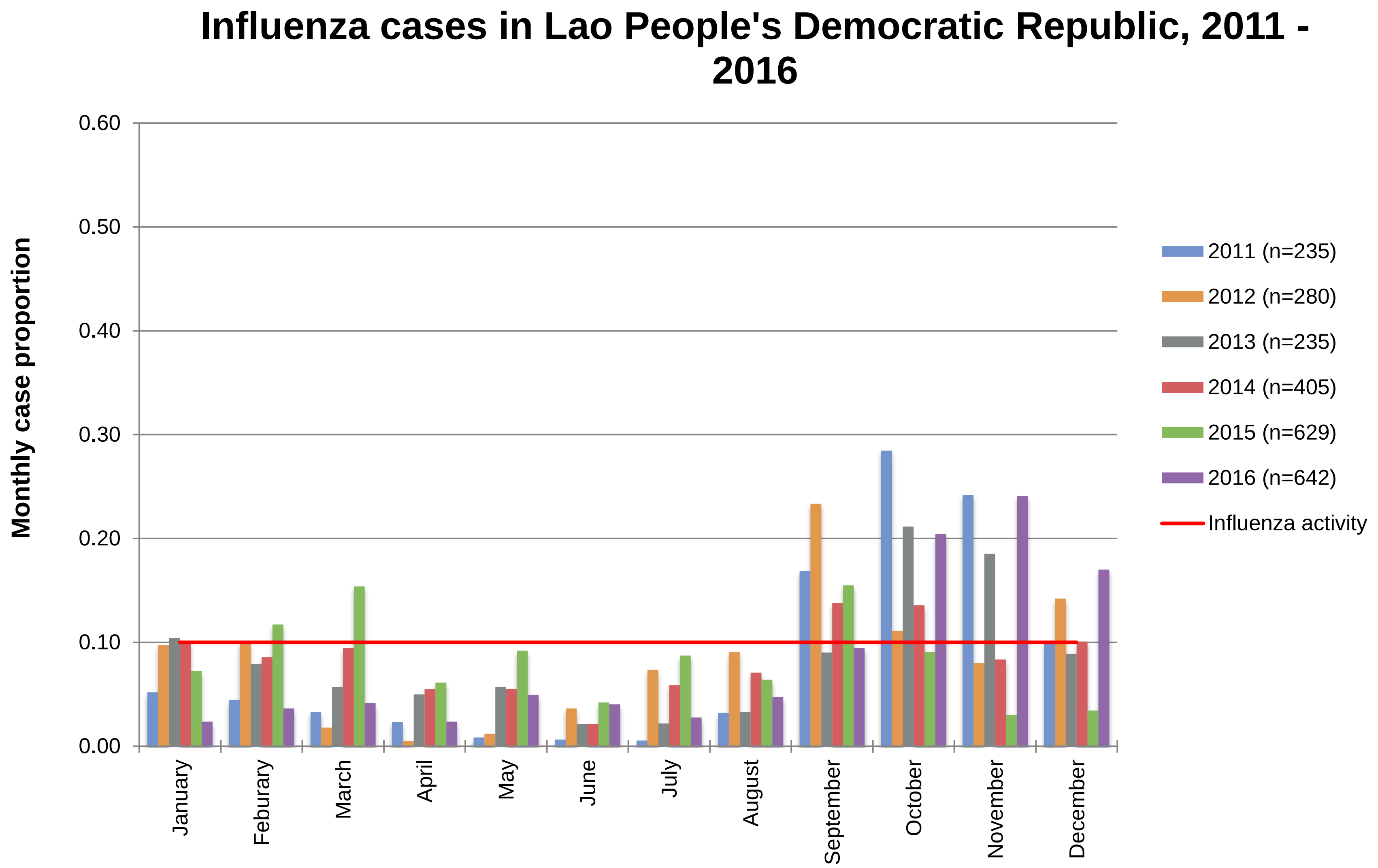

Influenza cases in Lao People's Democratic Republic, 2011 - 2016
Monthly case proportion
0.60
0.50
0.40
0.30
0.20
0.10
0.00
January
Feburary
March
April
May
June
July
August
September
October
November
December
2011 (n=235)
2012 (n=280)
2013 (n=235)
2014 (n=405)
2015 (n=629)
2016 (n=642)
Influenza activity

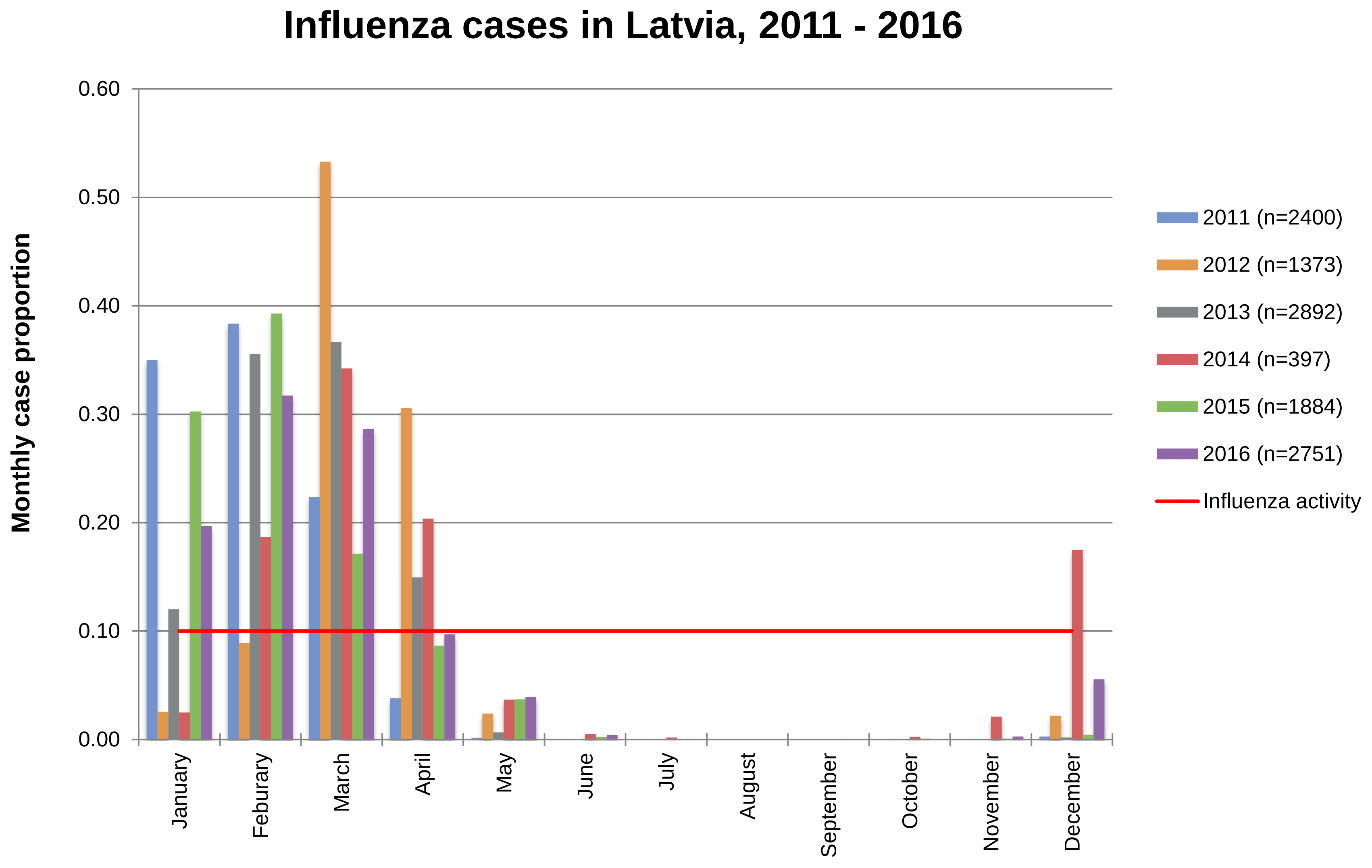

Influenza cases in Latvia, 2011 - 2016
Monthly case proportion
0.60
0.50
0.40
0.30
0.20
0.10
0.00
January
Feburary
March
April
May
June
July
August
September
October
November
December
2011 (n=2400)
2012 (n=1373)
2013 (n=2892)
2014 (n=397)
2015 (n=1884)
2016 (n=2751)
Influenza activity

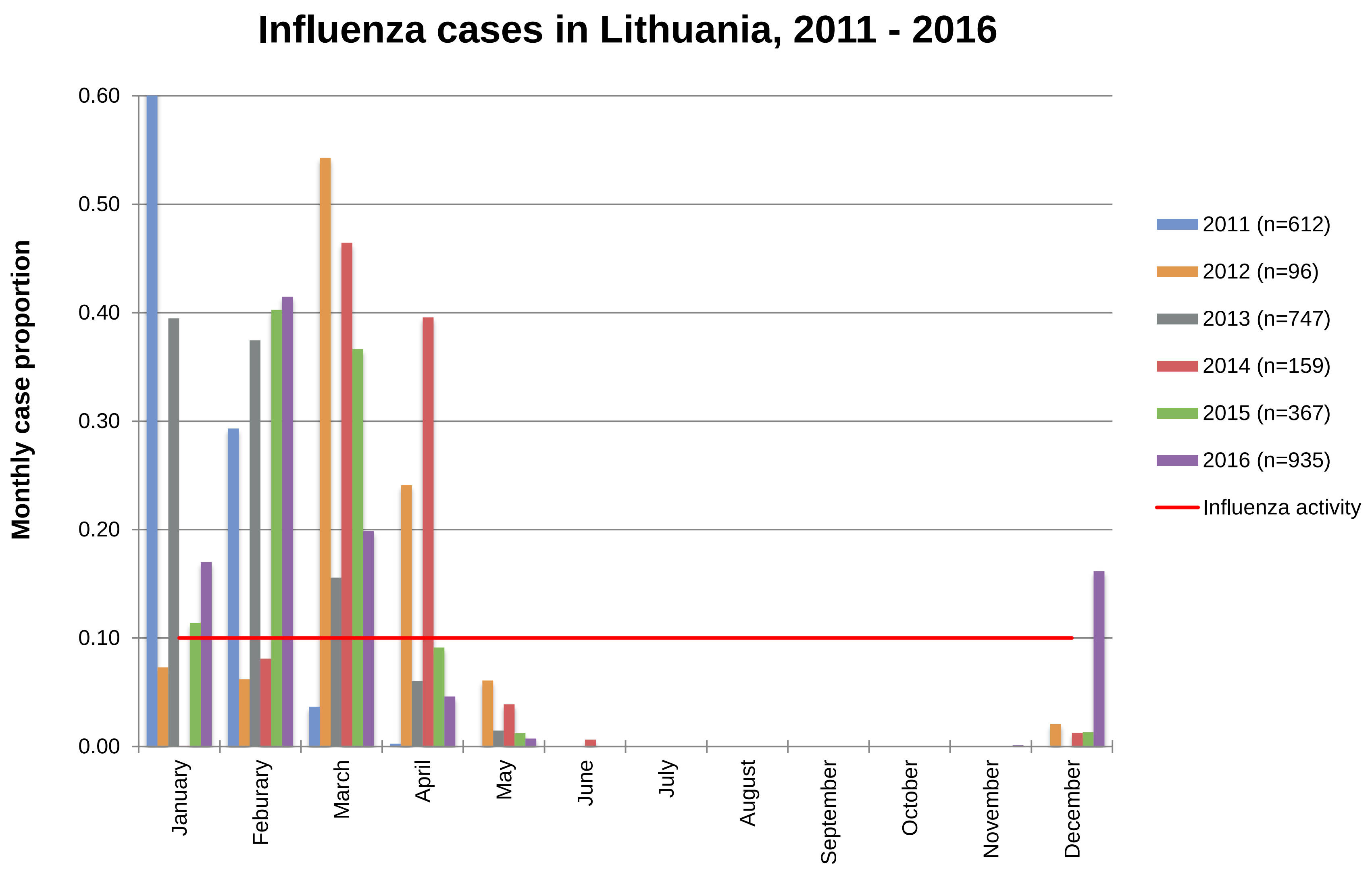
Influenza cases in Lithuania, 2011 - 2016
Monthly case proportion
0.60
0.50
0.40
0.30
0.20
0.10
0.00
January
Feburary
March
April
May
June
July
August
September
October
November
December
2011 (n=612)
2012 (n=96)
2013 (n=747)
2014 (n=159)
2015 (n=367)
2016 (n=935)
Influenza activity

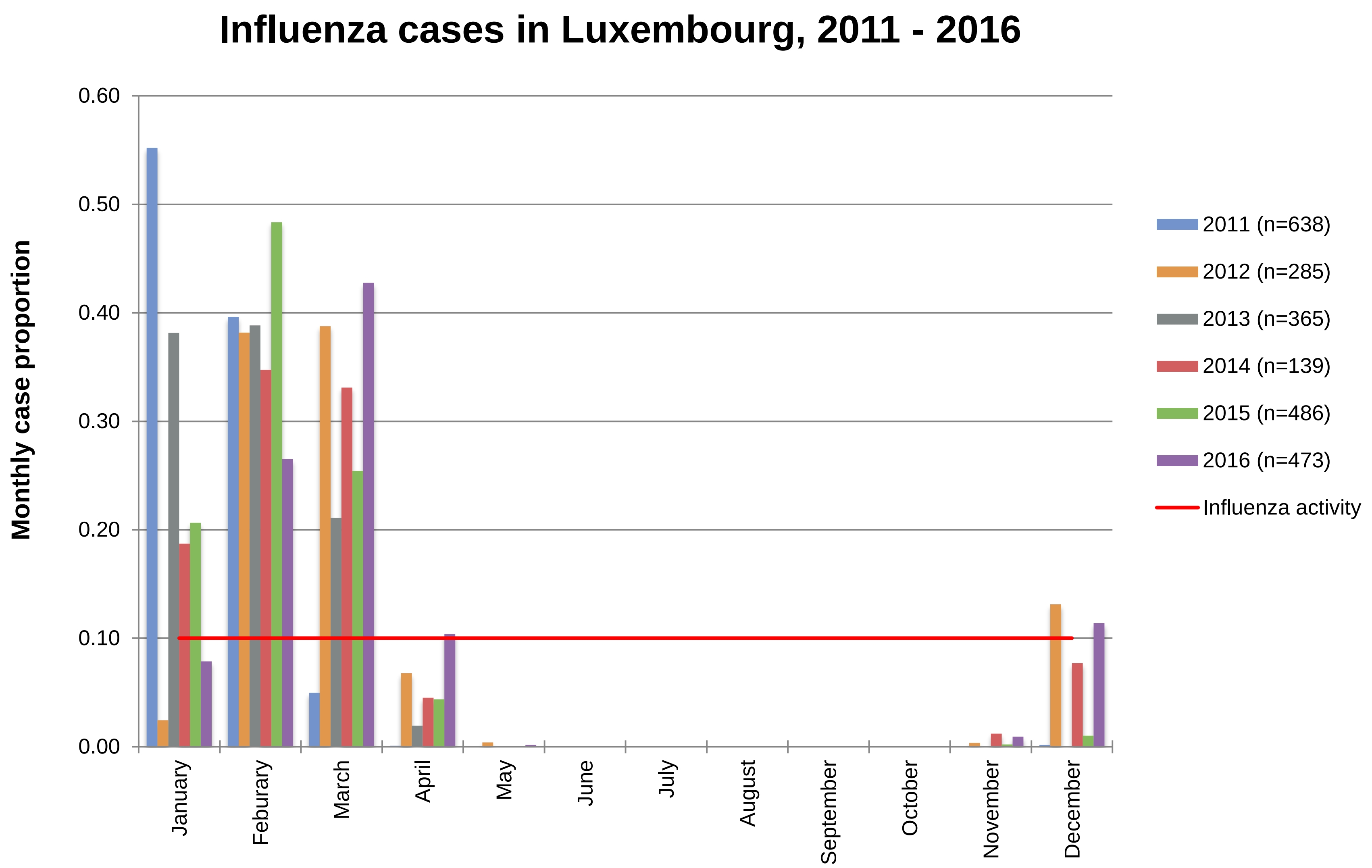
Influenza cases in Luxembourg, 2011 - 2016
Monthly case proportion
0.60
0.50
0.40
0.30
0.20
0.10
0.00
January
Feburary
March
April
May
June
July
August
September
October
November
December
2011 (n=638)
2012 (n=285)
2013 (n=365)
2014 (n=139)
2015 (n=486)
2016 (n=473)
Influenza activity

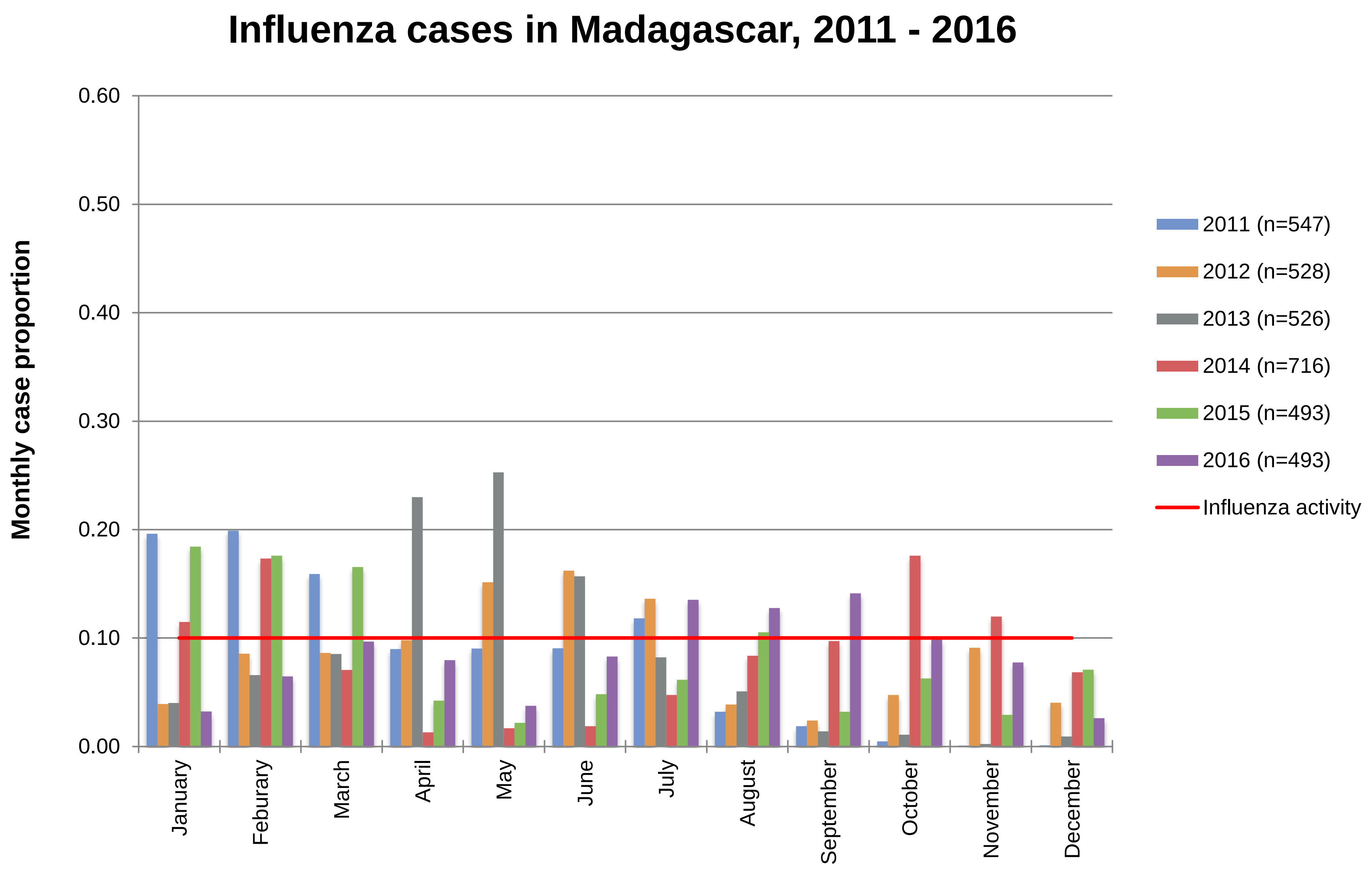
Influenza cases in Madagascar, 2011 - 2016
Monthly case proportion
0.60
0.50
0.40
0.30
0.20
0.10
0.00
January
Feburary
March
April
May
June
July
August
September
October
November
December
2011 (n=547)
2012 (n=528)
2013 (n=526)
2014 (n=716)
2015 (n=493)
2016 (n=493)
Influenza activity

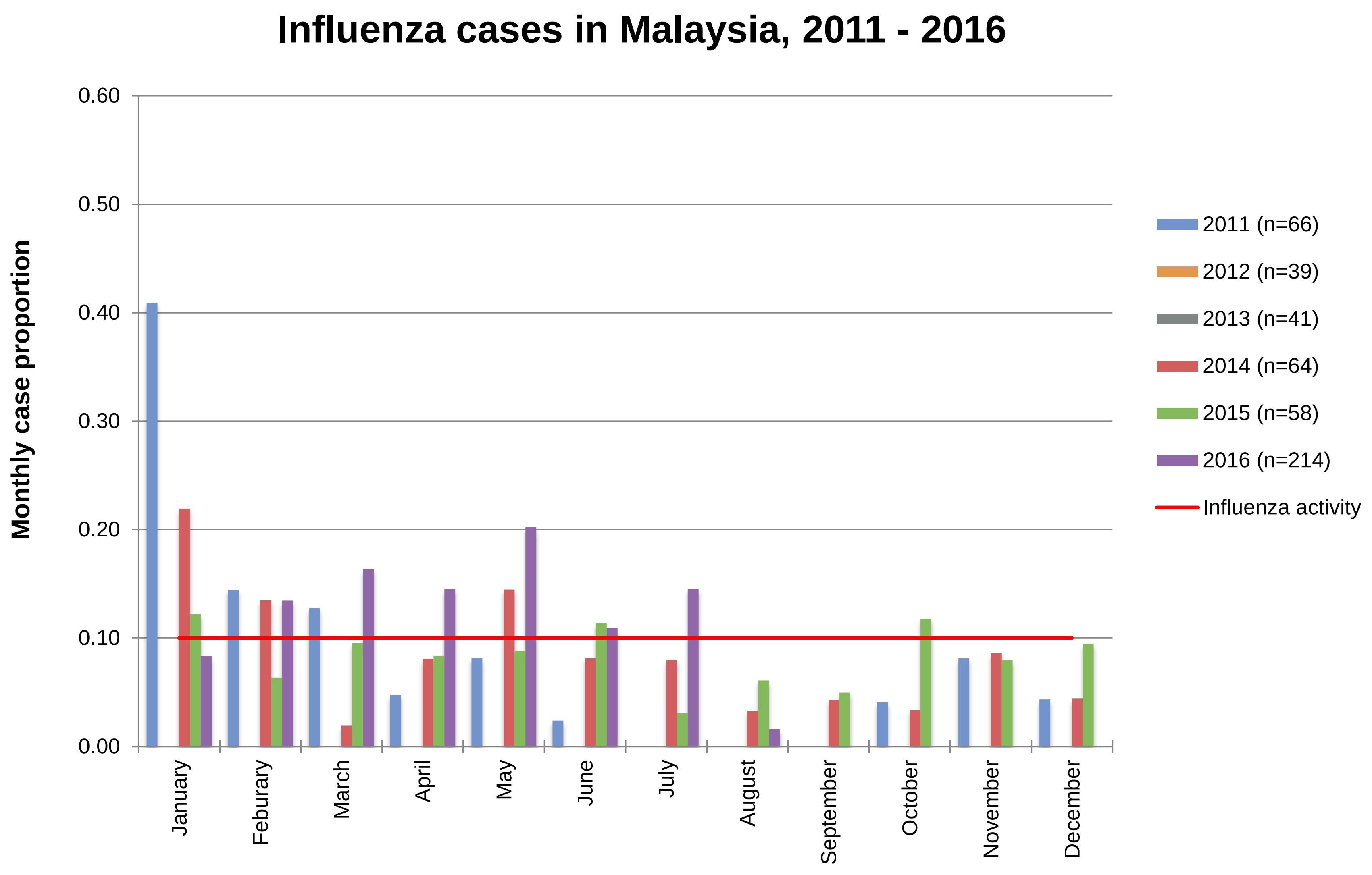

Influenza cases in Malaysia, 2011 - 2016
Monthly case proportion
0.60
0.50
0.40
0.30
0.20
0.10
0.00
January
Feburary
March
April
May
June
July
August
September
October
November
December
2011 (n=66)
2012 (n=39)
2013 (n=41)
2014 (n=64)
2015 (n=58)
2016 (n=214)
Influenza activity

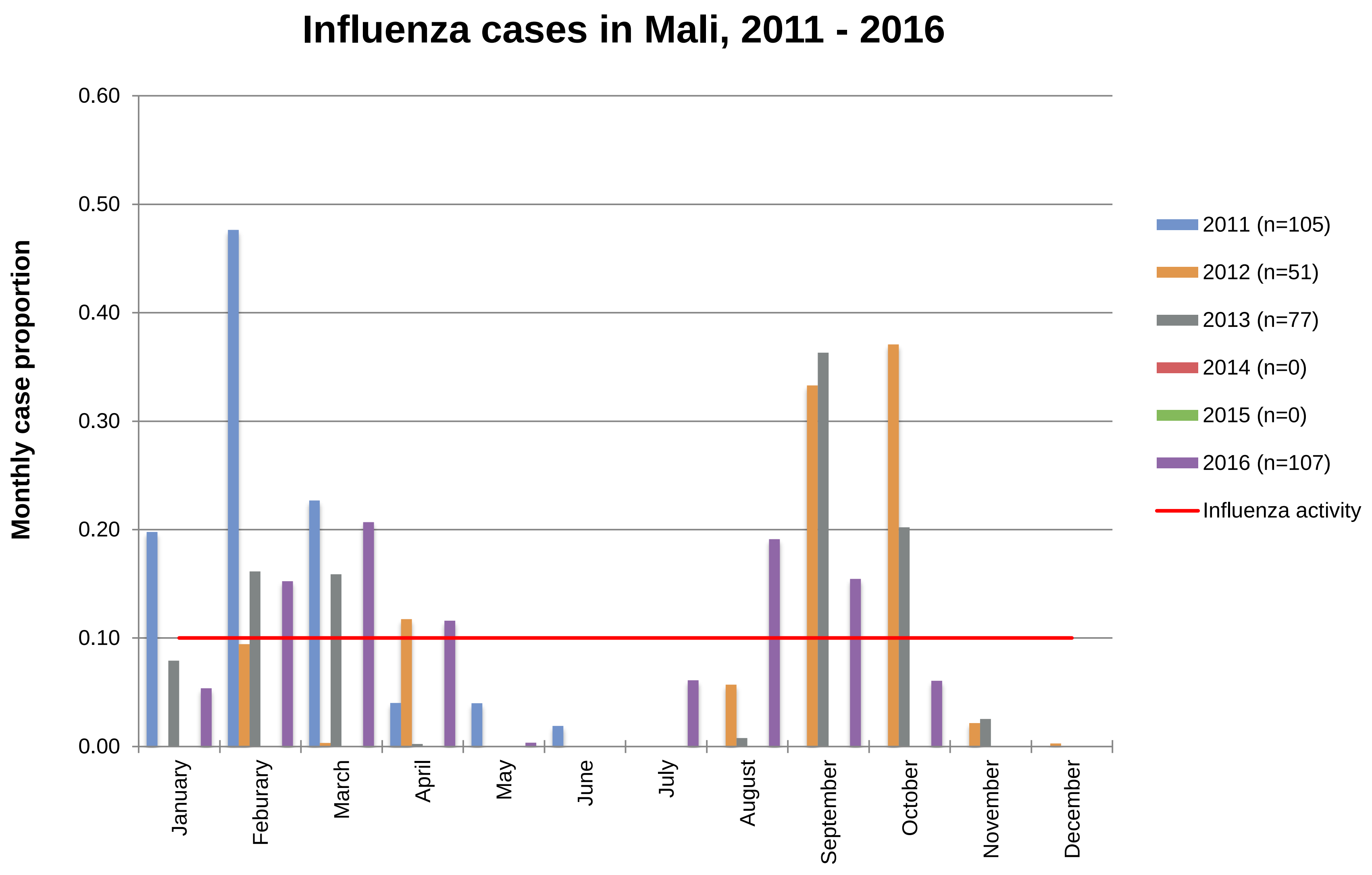
Influenza cases in Mali, 2011 - 2016
Monthly case proportion
0.60
0.50
0.40
0.30
0.20
0.10
0.00
January
Feburary
March
April
May
June
July
August
September
October
November
December
2011 (n=105)
2012 (n=51)
2013 (n=77)
2014 (n=0)
2015 (n=0)
2016 (n=107)
Influenza activity

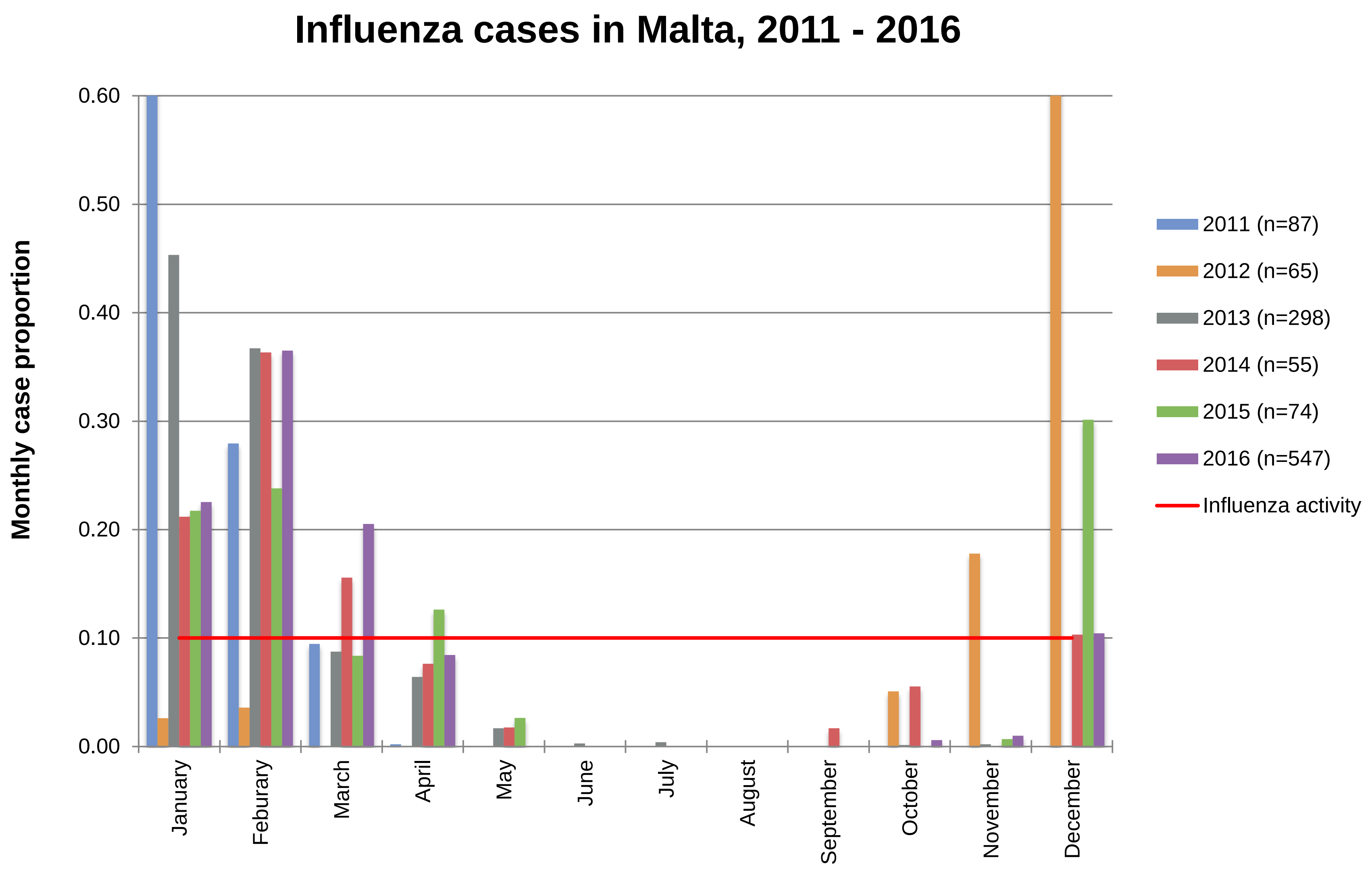

Influenza cases in Malta, 2011 - 2016
Monthly case proportion
0.60
0.50
0.40
0.30
0.20
0.10
0.00
January
Feburary
March
April
May
June
July
August
September
October
November
December
2011 (n=87)
2012 (n=65)
2013 (n=298)
2014 (n=55)
2015 (n=74)
2016 (n=547)
Influenza activity

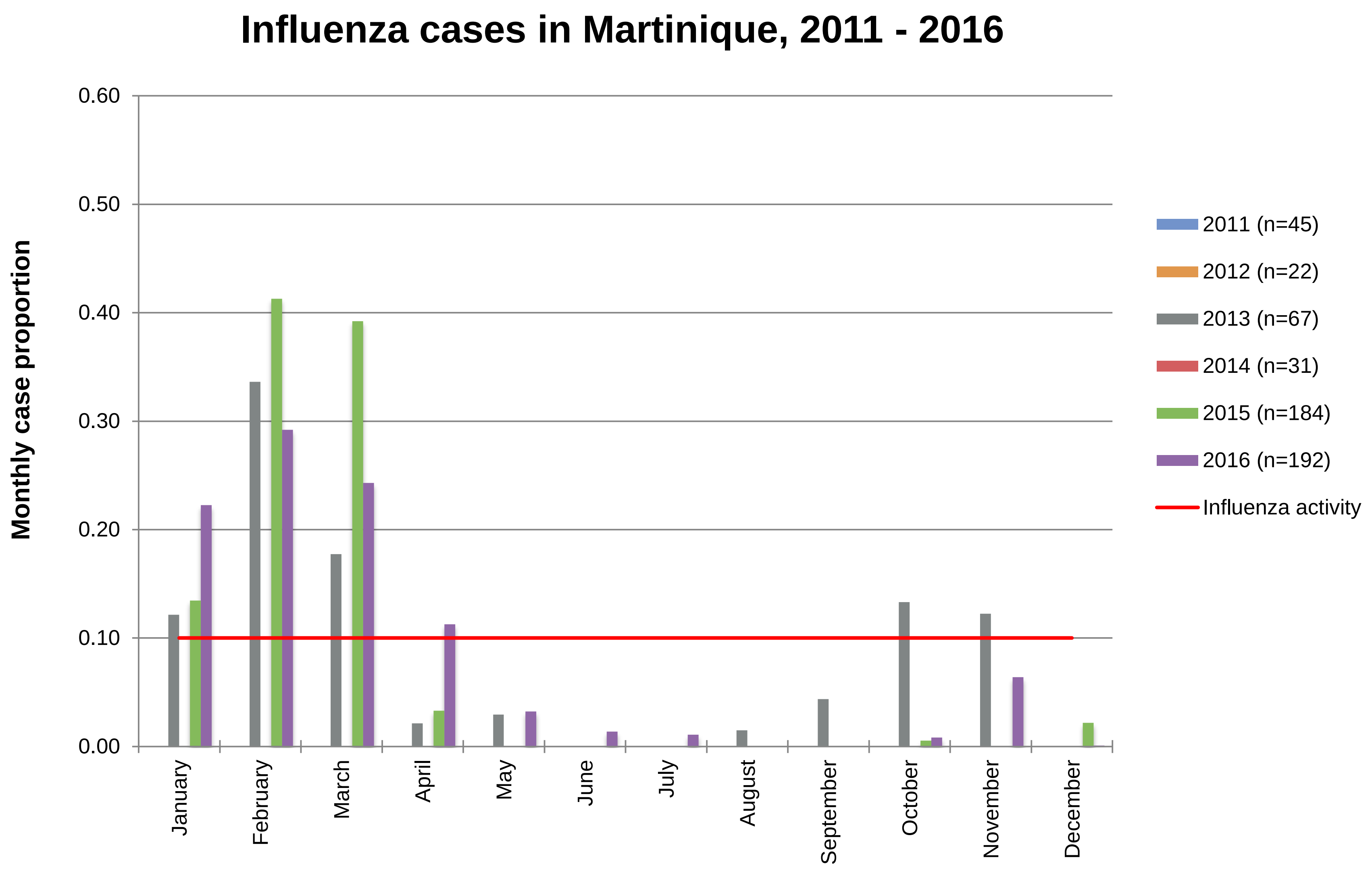
Influenza cases in Martinique, 2011 - 2016
Monthly case proportion
0.60
0.50
0.40
0.30
0.20
0.10
0.00
January
February
March
April
May
June
July
August
September
October
November
December
2011 (n=45)
2012 (n=22)
2013 (n=67)
2014 (n=31)
2015 (n=184)
2016 (n=192)
Influenza activity

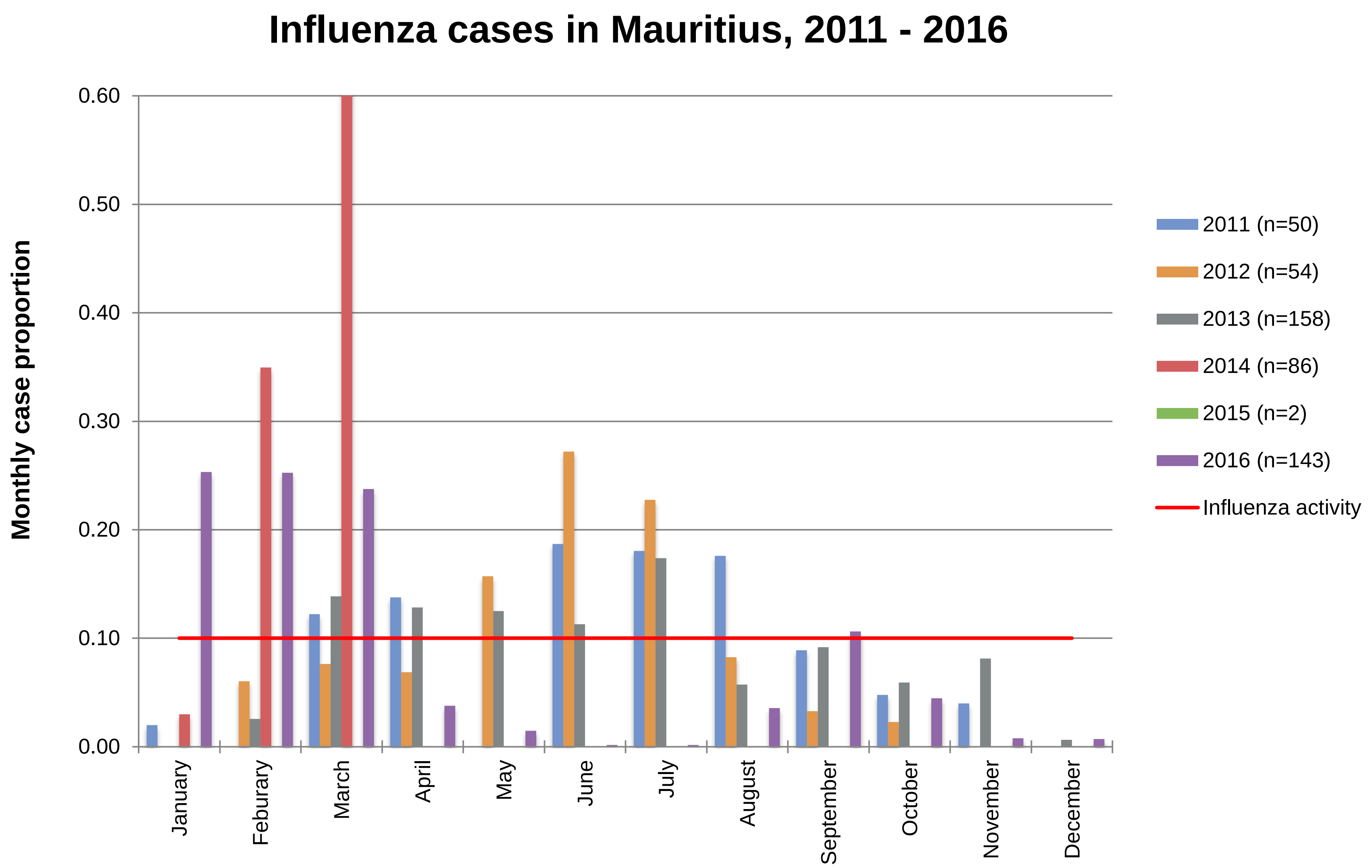

Influenza cases in Mauritius, 2011 - 2016
Monthly case proportion
0.60
0.50
0.40
0.30
0.20
0.10
0.00
January
Feburary
March
April
May
June
July
August
September
October
November
December
2011 (n=50)
2012 (n=54)
2013 (n=158)
2014 (n=86)
2015 (n=2)
2016 (n=143)
Influenza activity

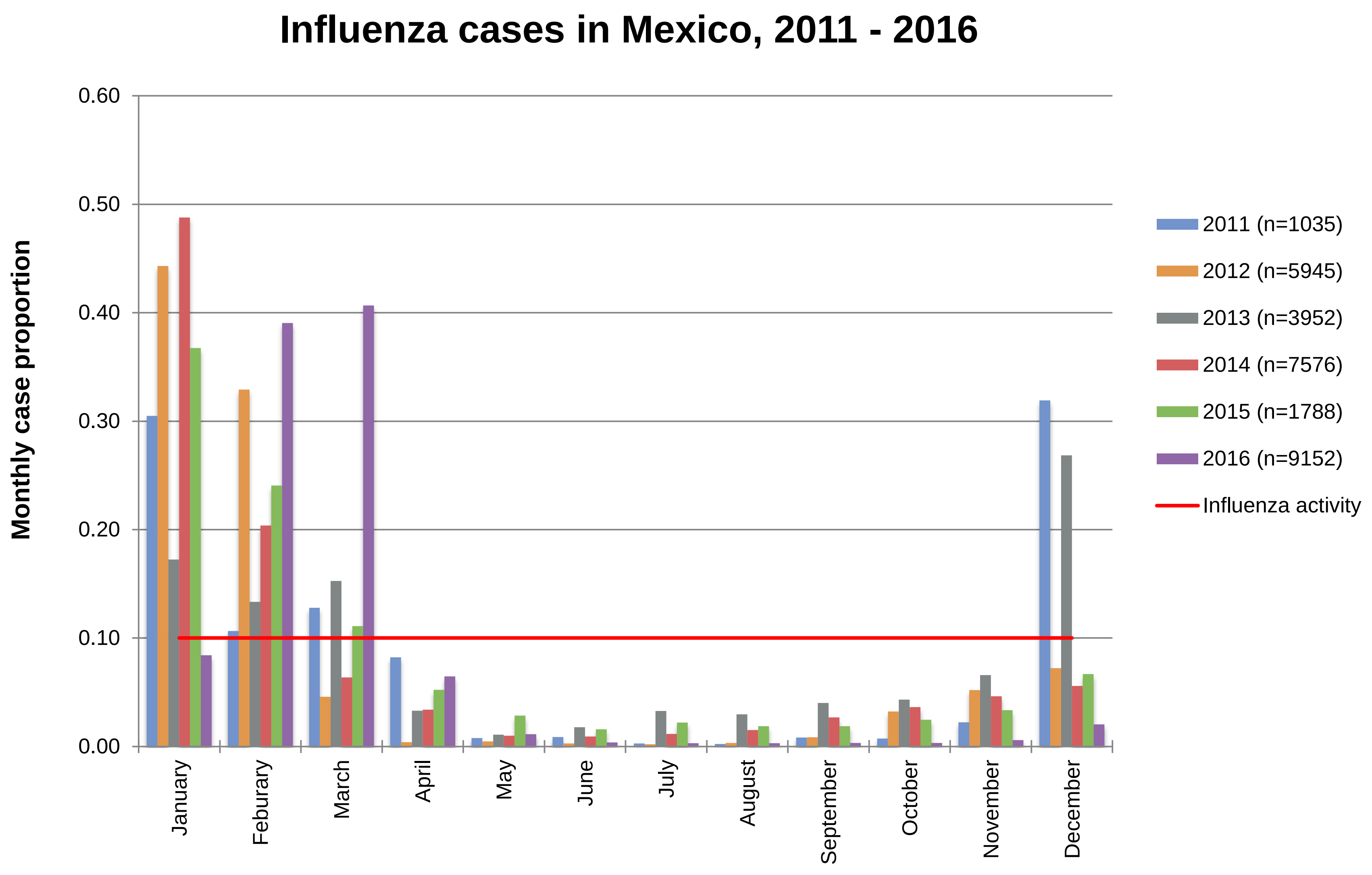
Influenza cases in Mexico, 2011 - 2016
Monthly case proportion
0.60
0.50
0.40
0.30
0.20
0.10
0.00
January
Feburary
March
April
May
June
July
August
September
October
November
December
2011 (n=1035)
2012 (n=5945)
2013 (n=3952)
2014 (n=7576)
2015 (n=1788)
2016 (n=9152)
Influenza activity

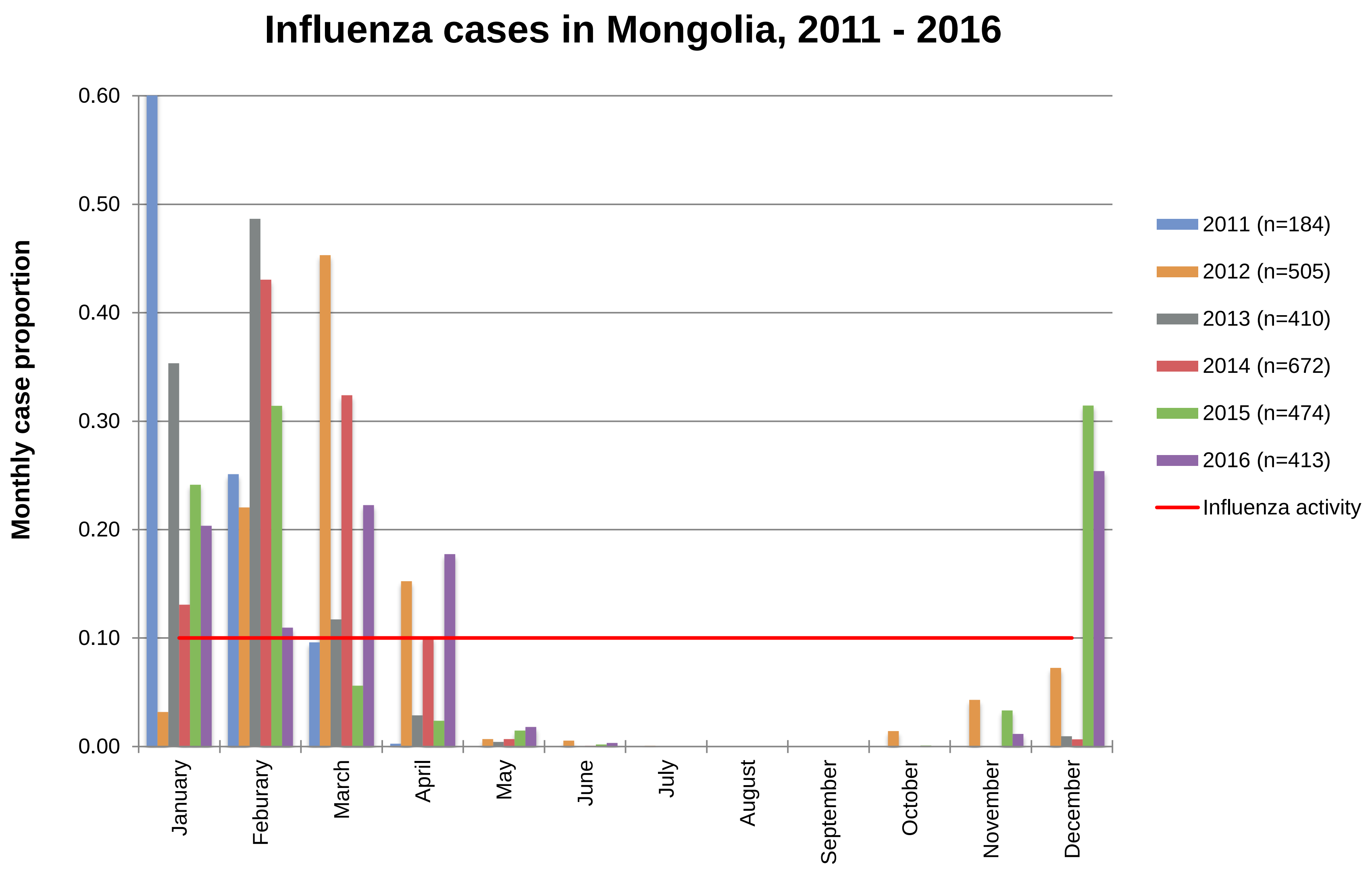

Influenza cases in Mongolia, 2011 - 2016
Monthly case proportion
0.60
0.50
0.40
0.30
0.20
0.10
0.00
January
Feburary
March
April
May
June
July
August
September
October
November
December
2011 (n=184)
2012 (n=505)
2013 (n=410)
2014 (n=672)
2015 (n=474)
2016 (n=413)
Influenza activity

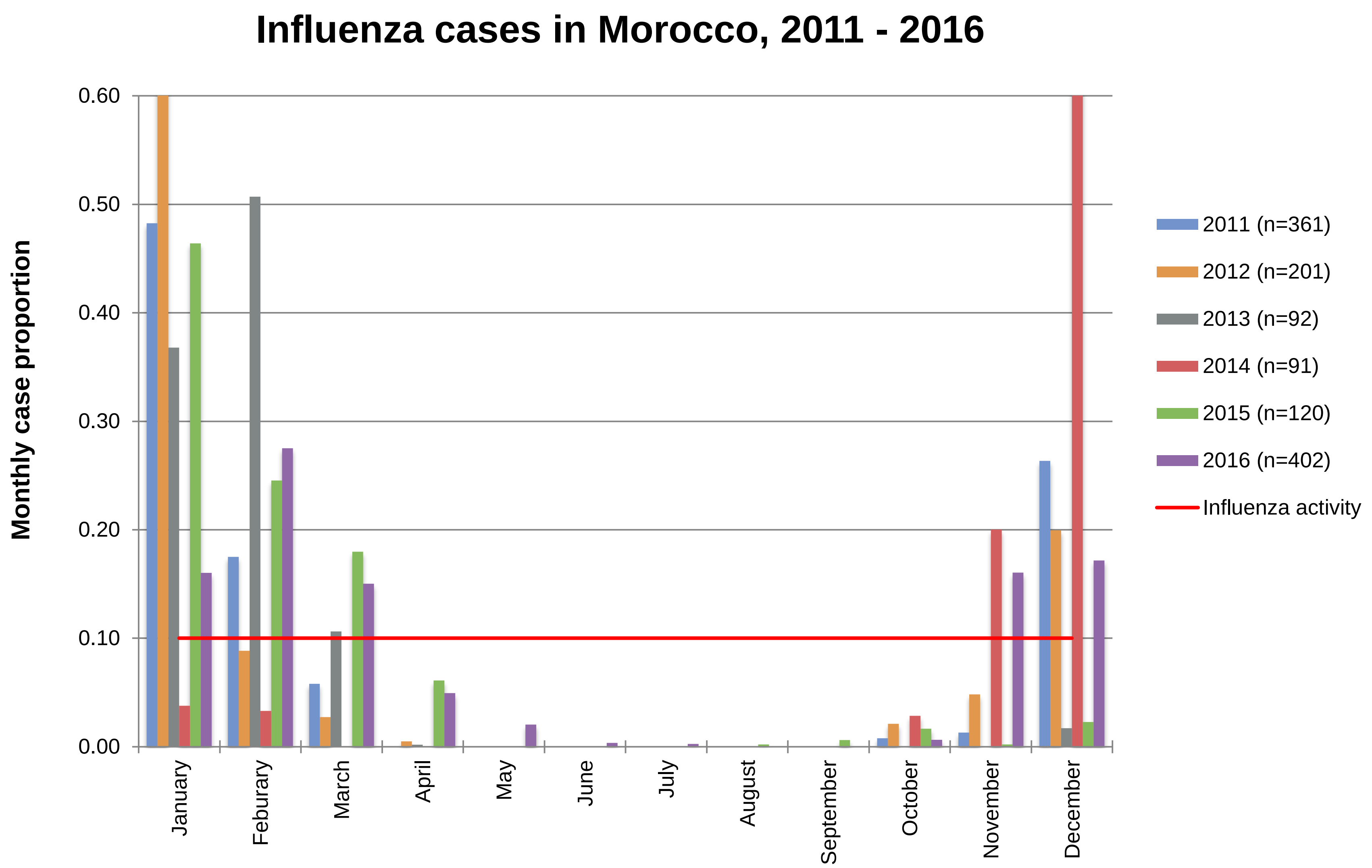
Influenza cases in Morocco, 2011 - 2016
Monthly case proportion
0.60
0.50
0.40
0.30
0.20
0.10
0.00
January
Feburary
March
April
May
June
July
August
September
October
November
December
2011 (n=361)
2012 (n=201)
2013 (n=92)
2014 (n=91)
2015 (n=120)
2016 (n=402)
Influenza activity

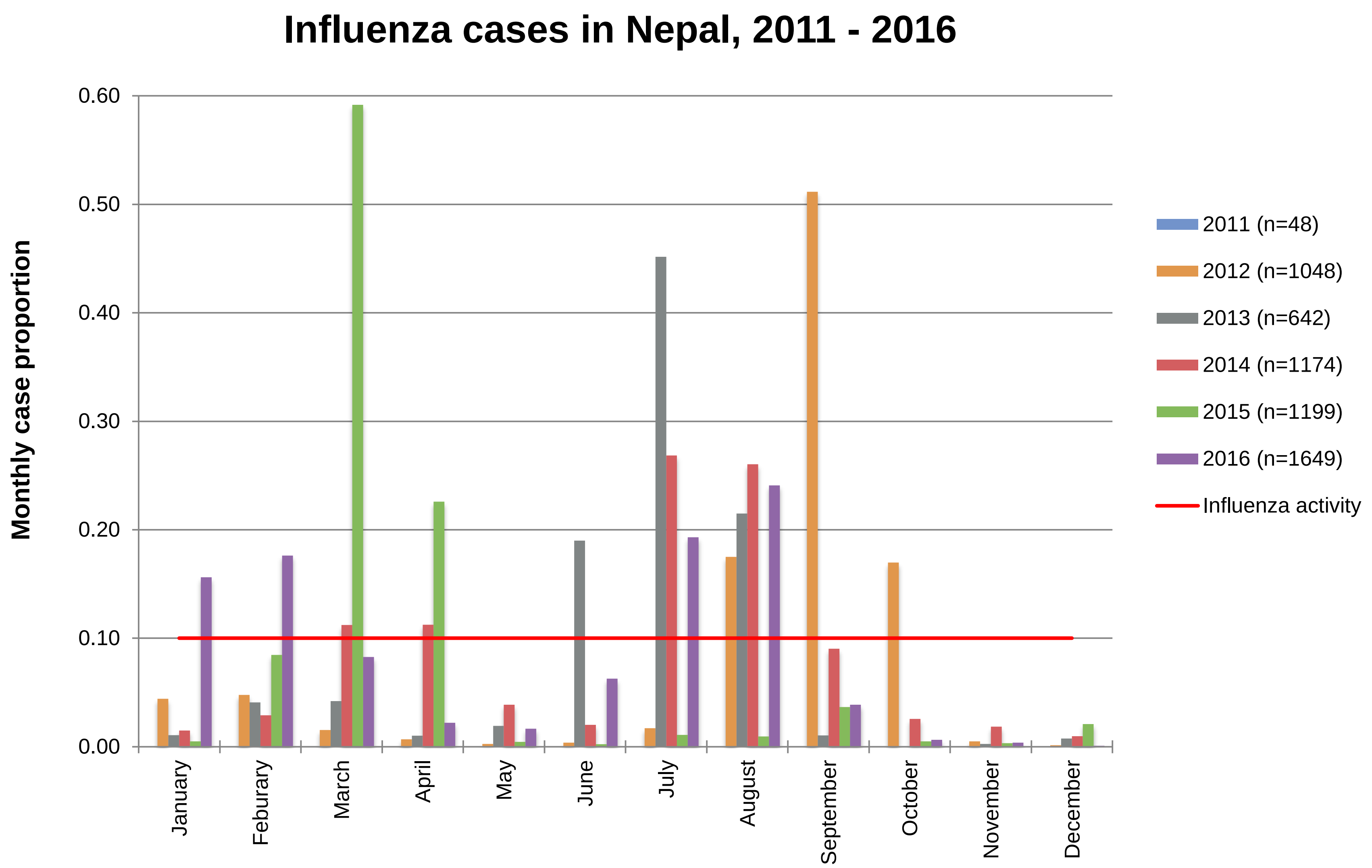
Influenza cases in Nepal, 2011 - 2016
Monthly case proportion
0.60
0.50
0.40
0.30
0.20
0.10
0.00
January
Feburary
March
April
May
June
July
August
September
October
November
December
2011 (n=48)
2012 (n=1048)
2013 (n=642)
2014 (n=1174)
2015 (n=1199)
2016 (n=1649)
Influenza activity

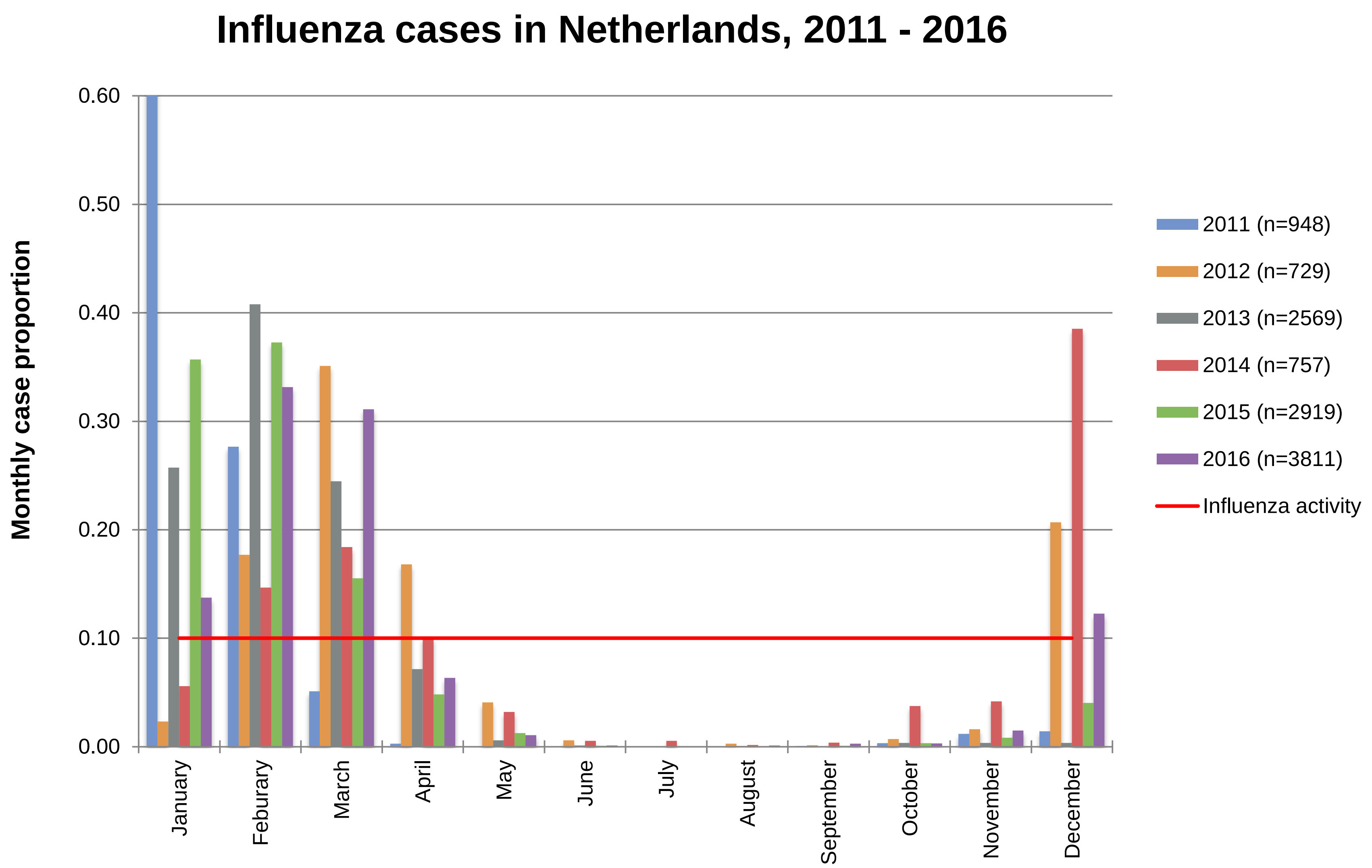

Influenza cases in Netherlands, 2011 - 2016
Monthly case proportion
0.60
0.50
0.40
0.30
0.20
0.10
0.00
January
Feburary
March
April
May
June
July
August
September
October
November
December
2011 (n=948)
2012 (n=729)
2013 (n=2569)
2014 (n=757)
2015 (n=2919)
2016 (n=3811)
Influenza activity

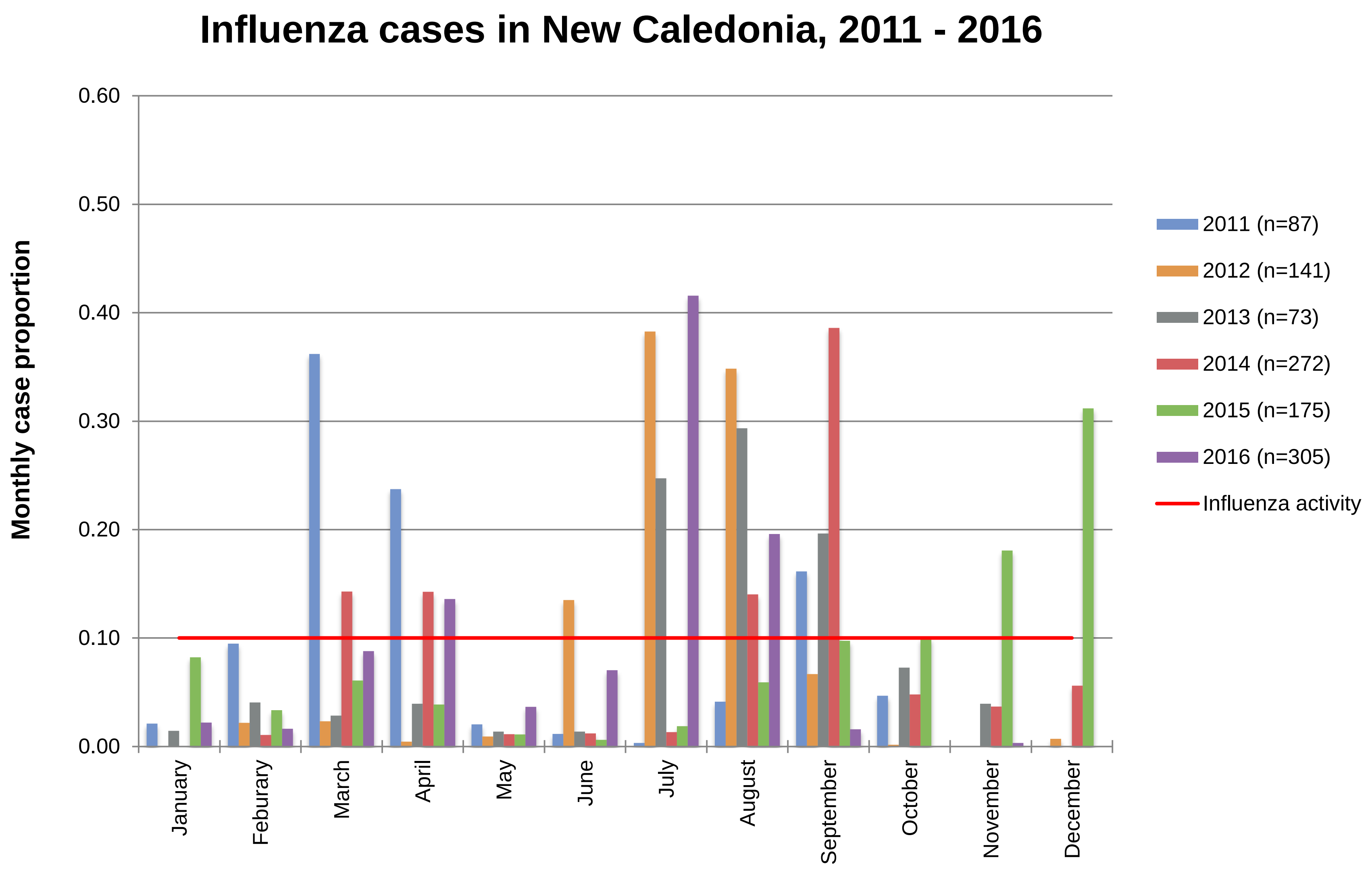
Influenza cases in New Caledonia, 2011 - 2016
Monthly case proportion
0.60
0.50
0.40
0.30
0.20
0.10
0.00
January
Feburary
March
April
May
June
July
August
September
October
November
December
2011 (n=87)
2012 (n=141)
2013 (n=73)
2014 (n=272)
2015 (n=175)
2016 (n=305)
Influenza activity

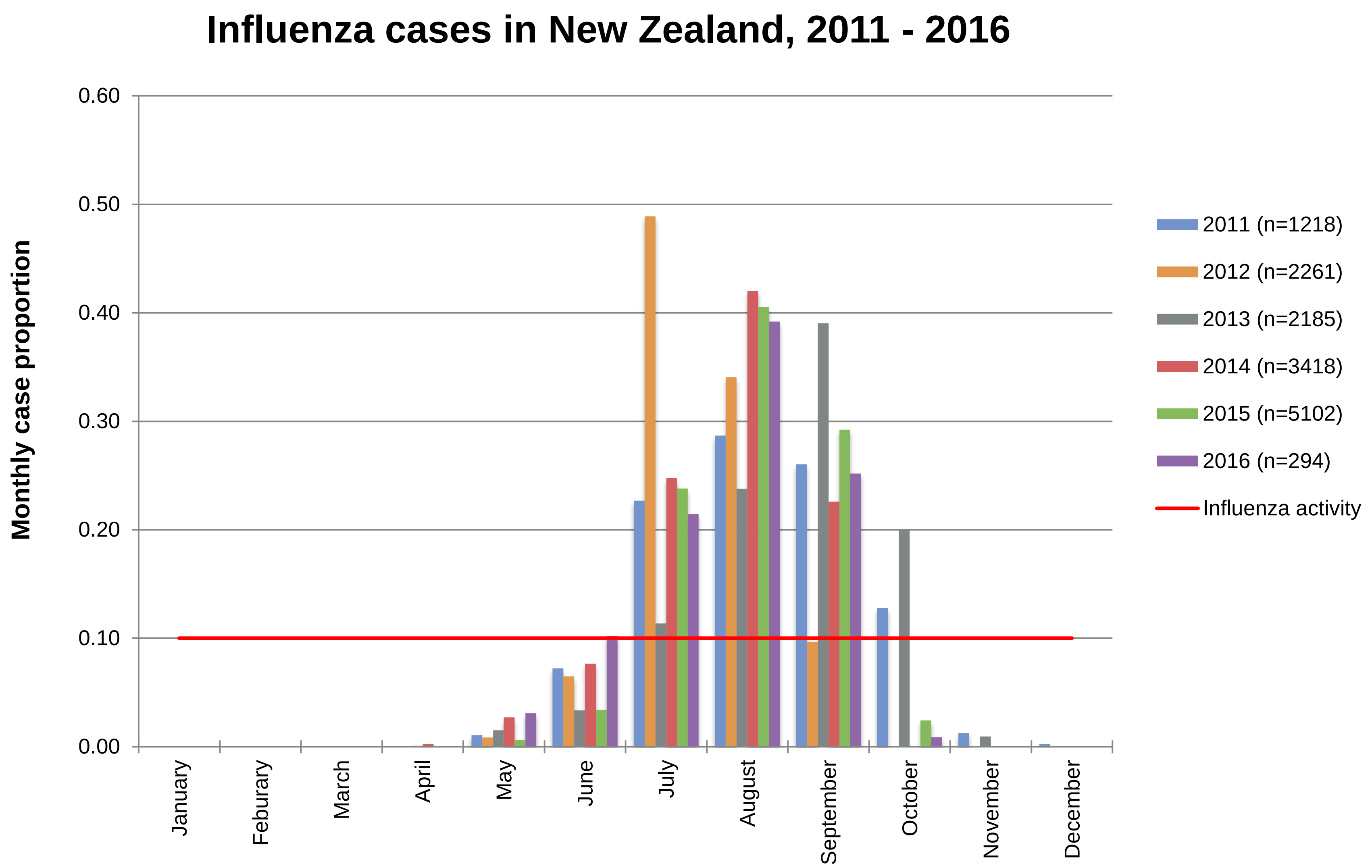

Influenza cases in New Zealand, 2011 - 2016
Monthly case proportion
0.60
0.50
0.40
0.30
0.20
0.10
0.00
January
Feburary
March
April
May
June
July
August
September
October
November
December
2011 (n=1218)
2012 (n=2261)
2013 (n=2185)
2014 (n=3418)
2015 (n=5102)
2016 (n=294)
Influenza activity

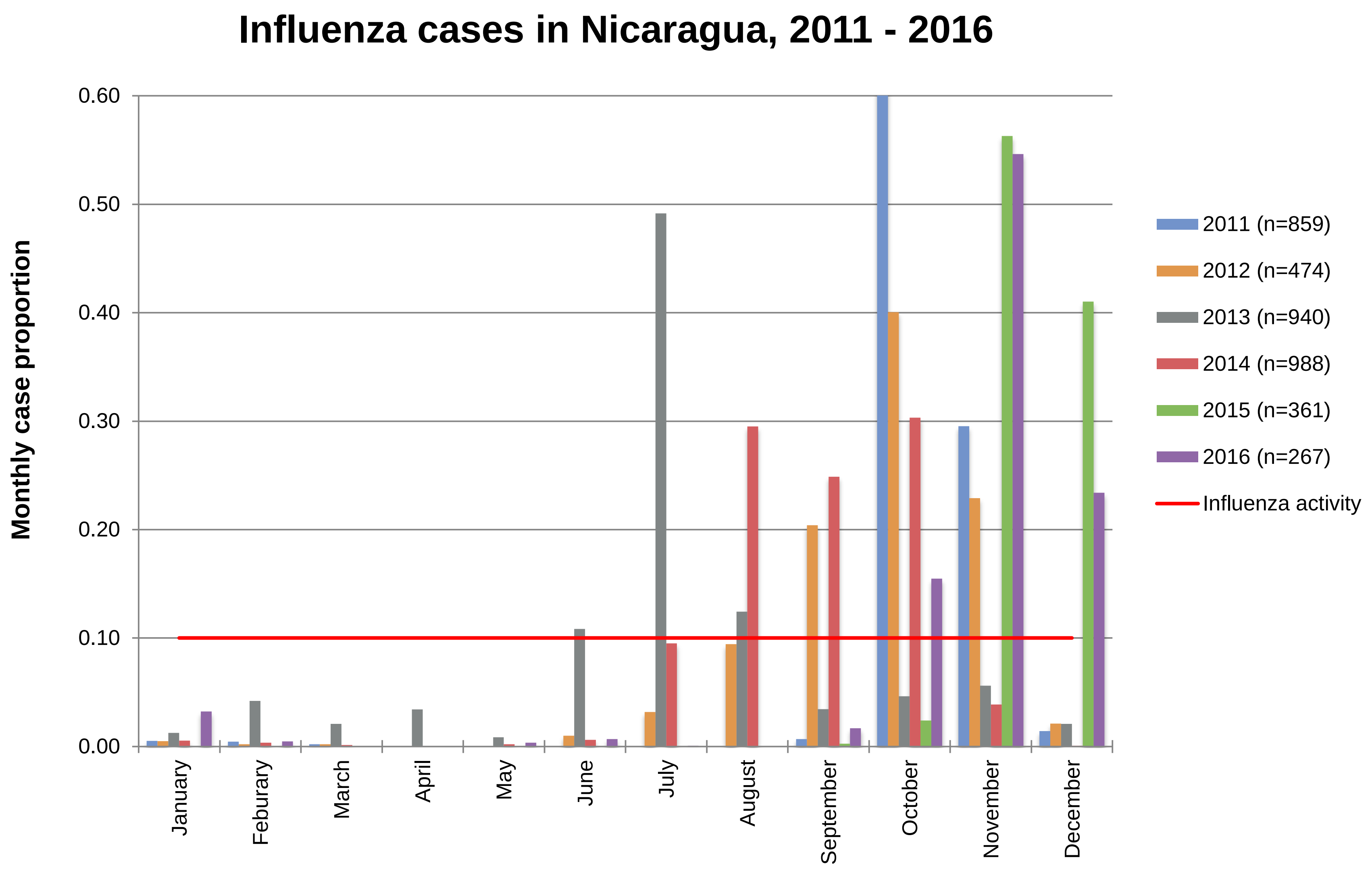

Influenza cases in Nicaragua, 2011 - 2016
Monthly case proportion
0.60
0.50
0.40
0.30
0.20
0.10
0.00
January
Feburary
March
April
May
June
July
August
September
October
November
December
2011 (n=859)
2012 (n=474)
2013 (n=940)
2014 (n=988)
2015 (n=361)
2016 (n=267)
Influenza activity

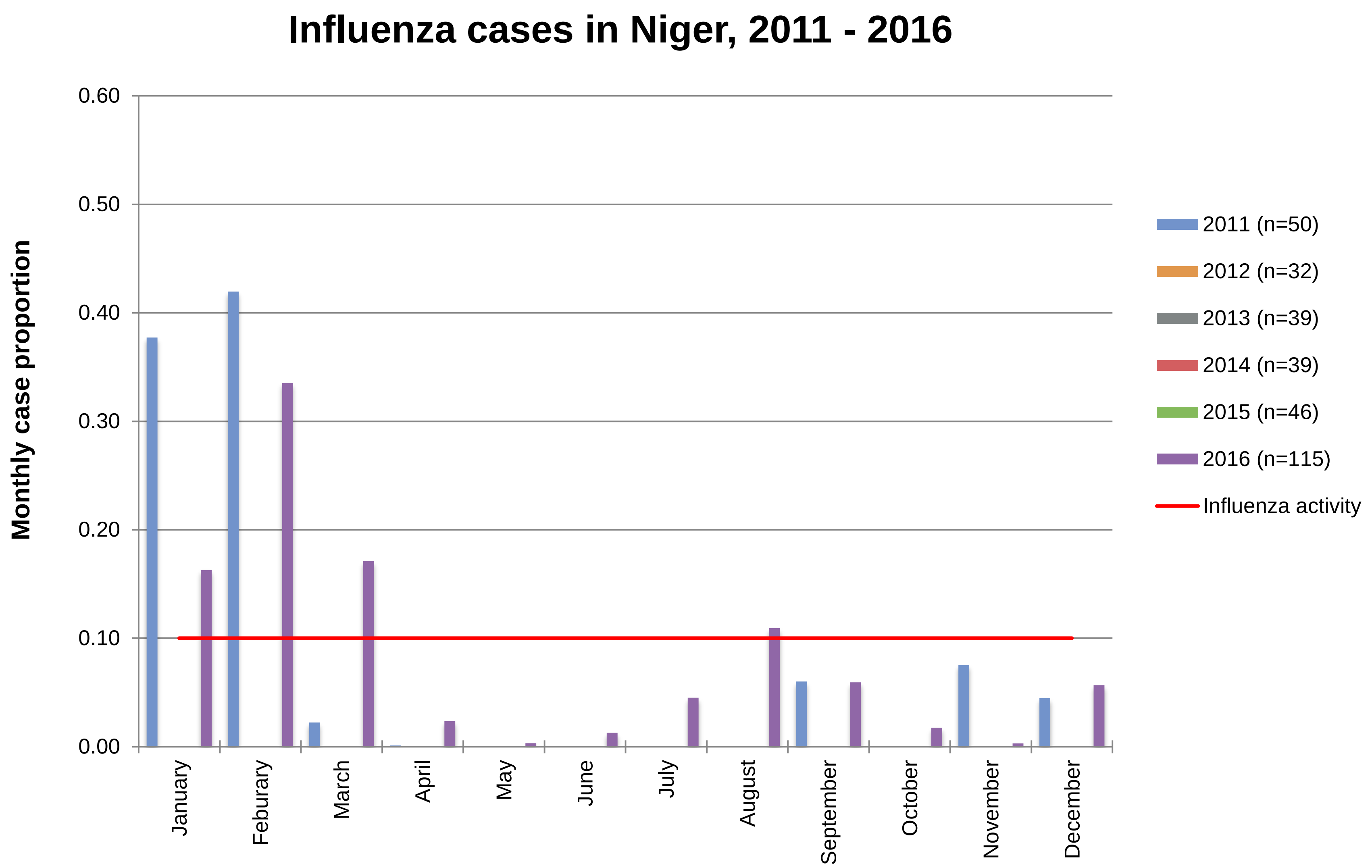

Influenza cases in Niger, 2011 - 2016
Monthly case proportion
0.60
0.50
0.40
0.30
0.20
0.10
0.00
January
Feburary
March
April
May
June
July
August
September
October
November
December
2011 (n=50)
2012 (n=32)
2013 (n=39)
2014 (n=39)
2015 (n=46)
2016 (n=115)
Influenza activity

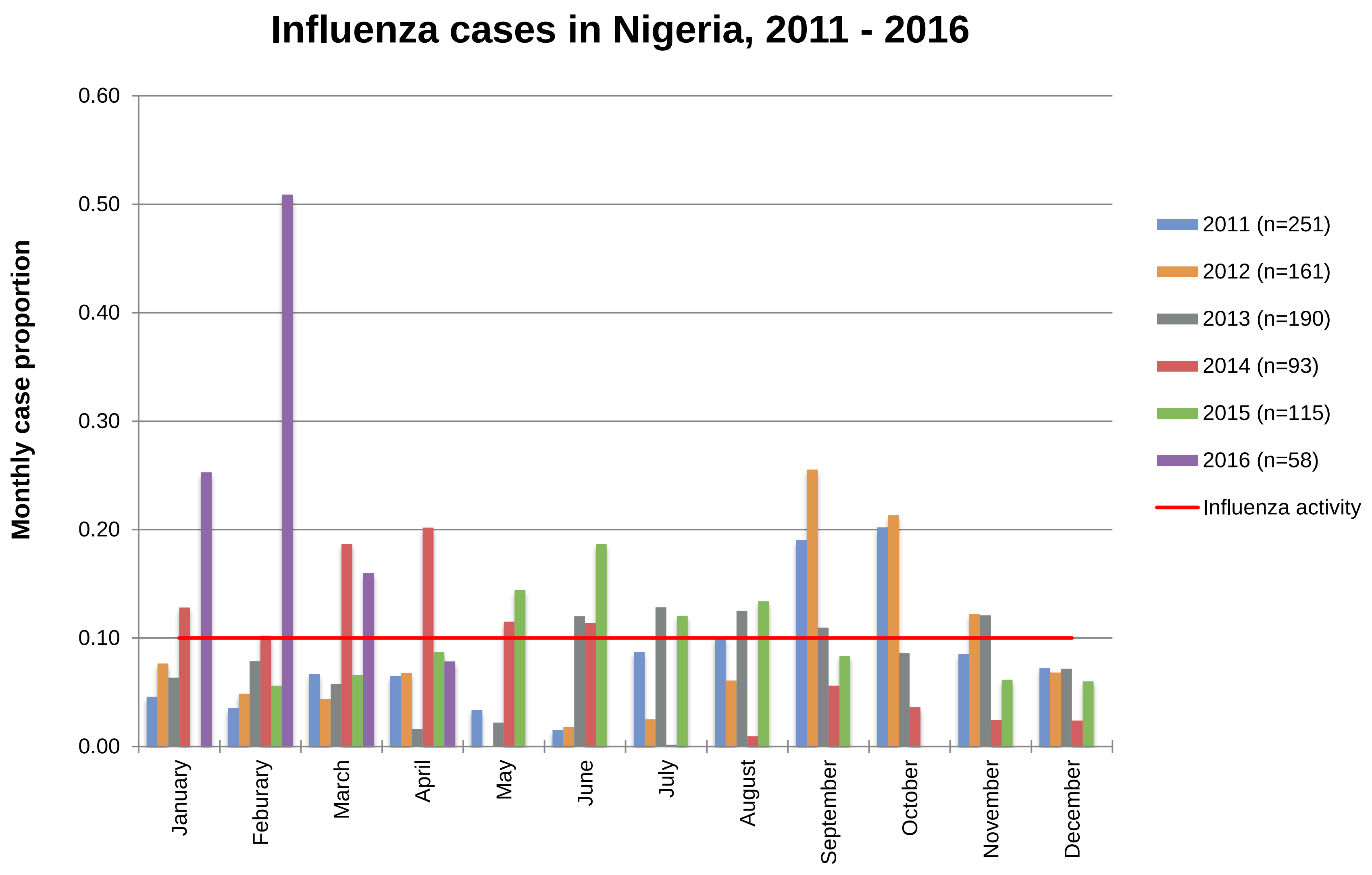

Influenza cases in Nigeria, 2011 - 2016
Monthly case proportion
0.60
0.50
0.40
0.30
0.20
0.10
0.00
January
Feburary
March
April
May
June
July
August
September
October
November
December
2011 (n=251)
2012 (n=161)
2013 (n=190)
2014 (n=93)
2015 (n=115)
2016 (n=58)
Influenza activity

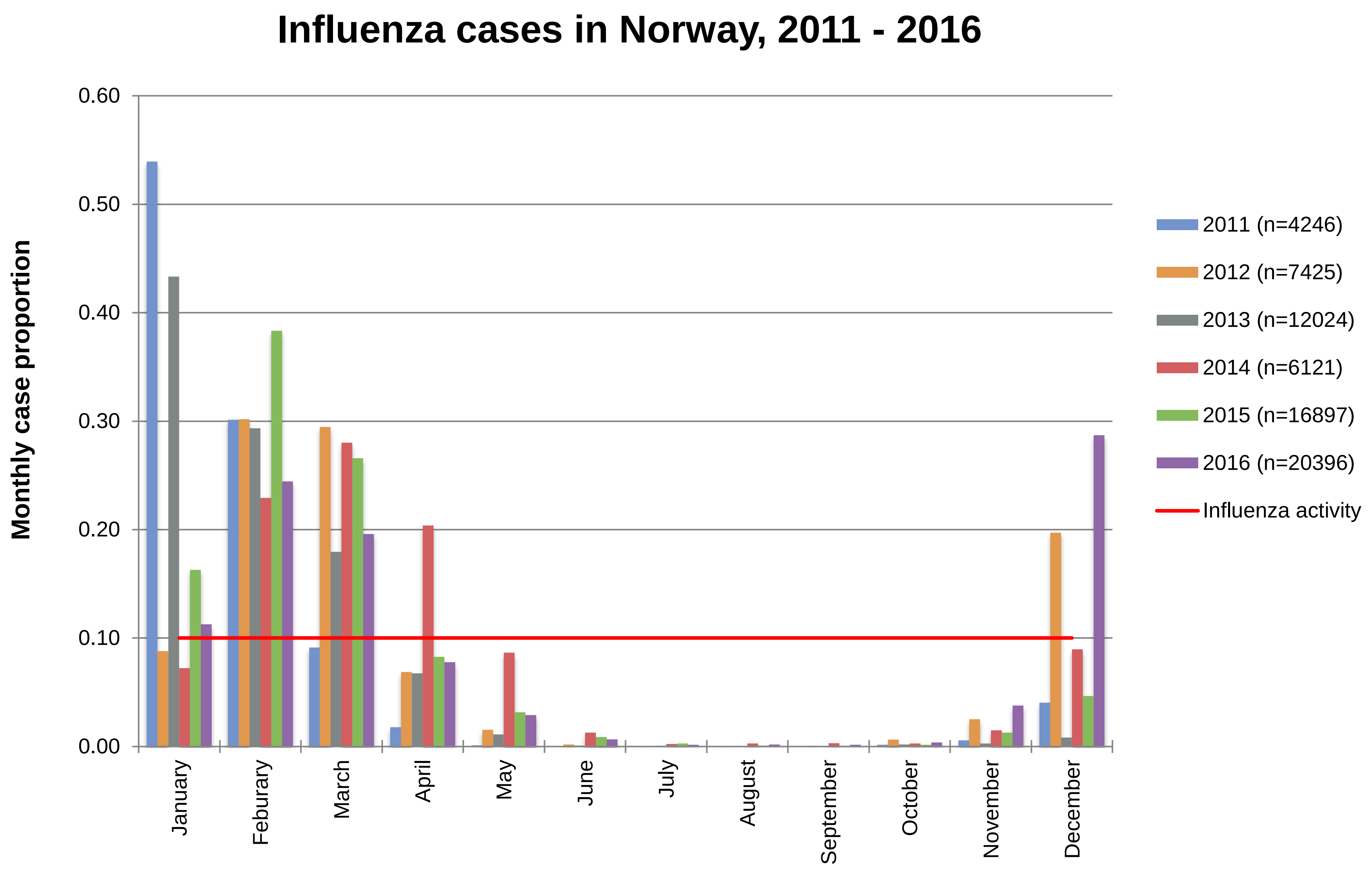
Influenza cases in Norway, 2011 - 2016
Monthly case proportion
0.60
0.50
0.40
0.30
0.20
0.10
0.00
January
Feburary
March
April
May
June
July
August
September
October
November
December
2011 (n=4246)
2012 (n=7425)
2013 (n=12024)
2014 (n=6121)
2015 (n=16897)
2016 (n=20396)
Influenza activity

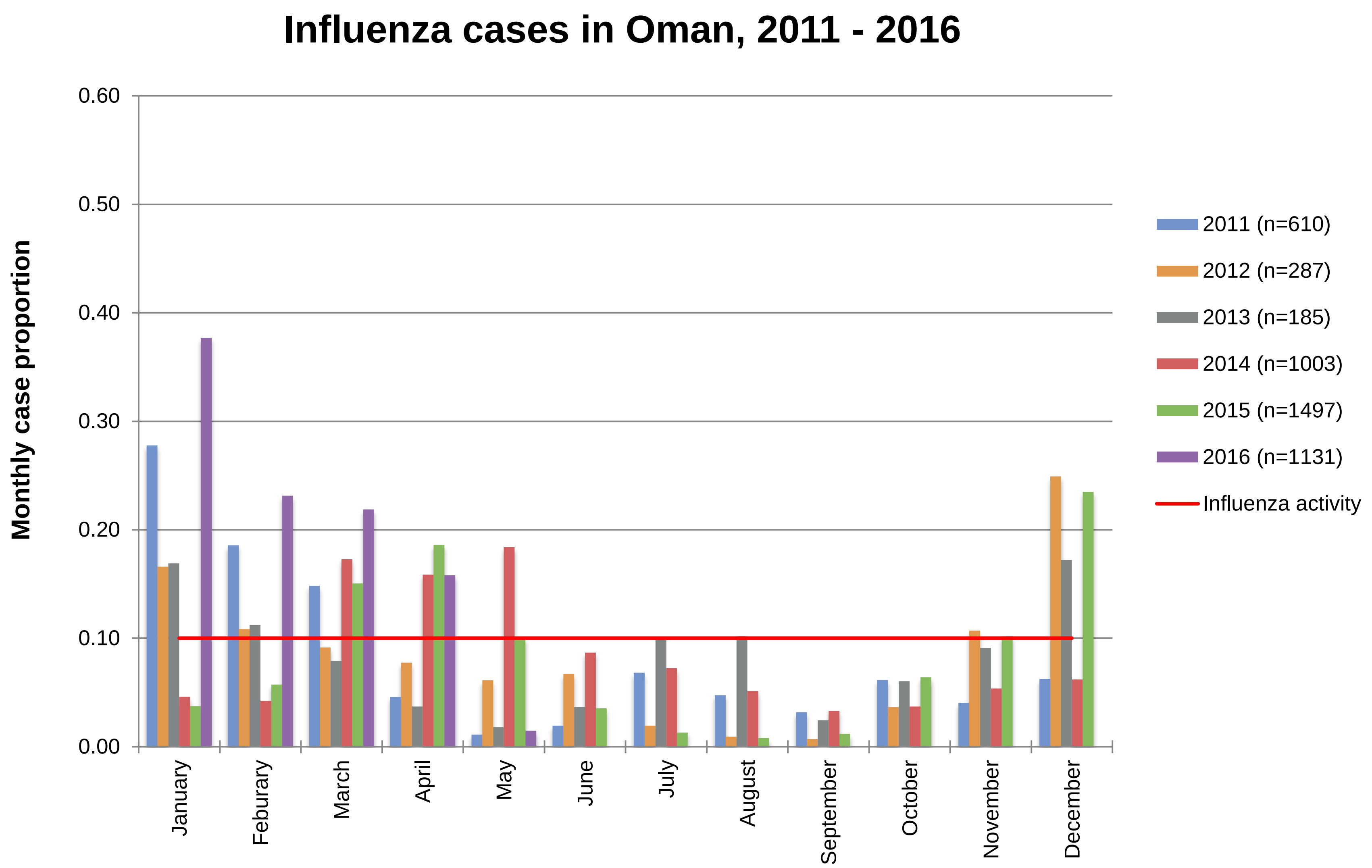

Influenza cases in Oman, 2011 - 2016
Monthly case proportion
0.60
0.50
0.40
0.30
0.20
0.10
0.00
January
Feburary
March
April
May
June
July
August
September
October
November
December
2011 (n=610)
2012 (n=287)
2013 (n=185)
2014 (n=1003)
2015 (n=1497)
2016 (n=1131)
Influenza activity

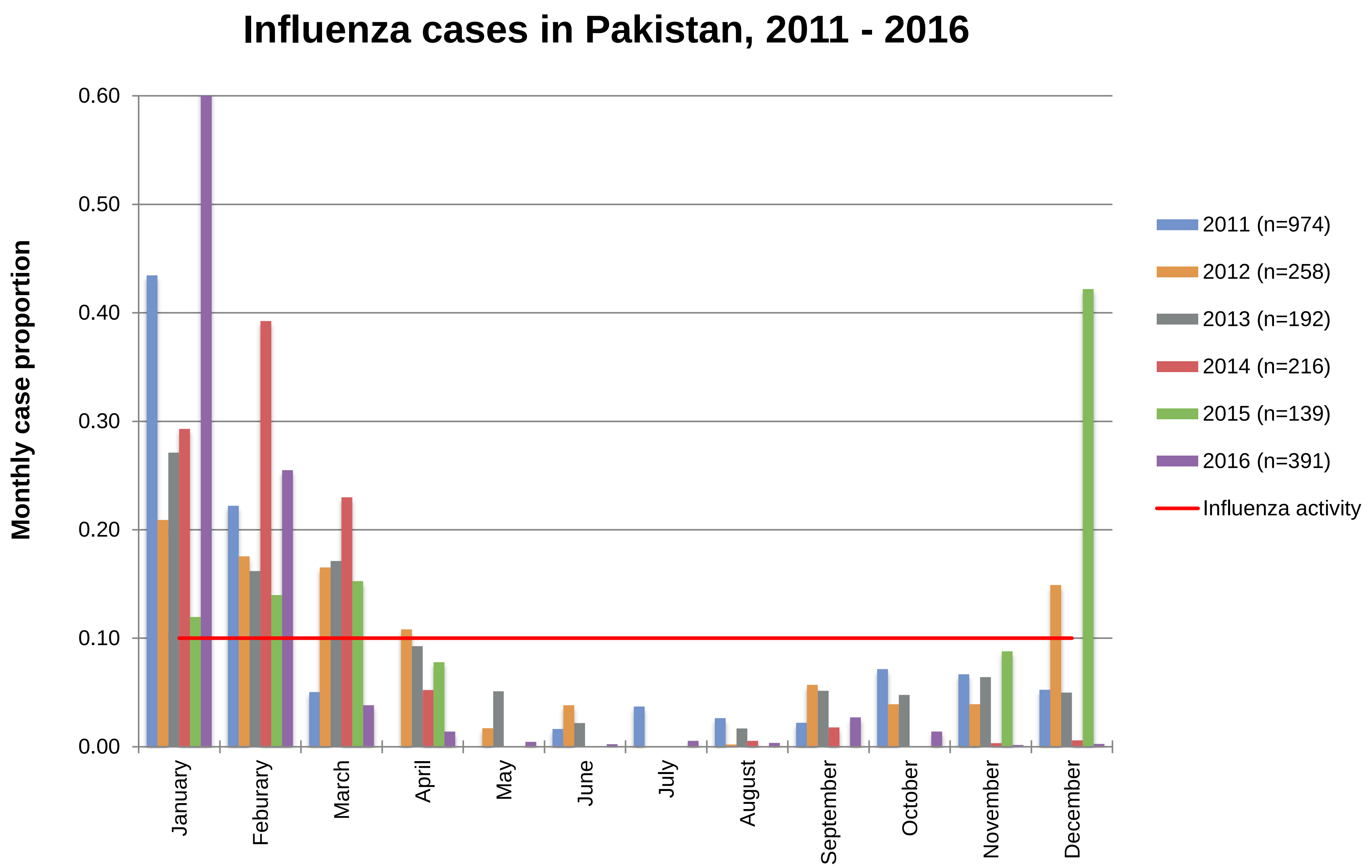

Influenza cases in Pakistan, 2011 - 2016
Monthly case proportion
0.60
0.50
0.40
0.30
0.20
0.10
0.00
January
Feburary
March
April
May
June
July
August
September
October
November
December
2011 (n=974)
2012 (n=258)
2013 (n=192)
2014 (n=216)
2015 (n=139)
2016 (n=391)
Influenza activity

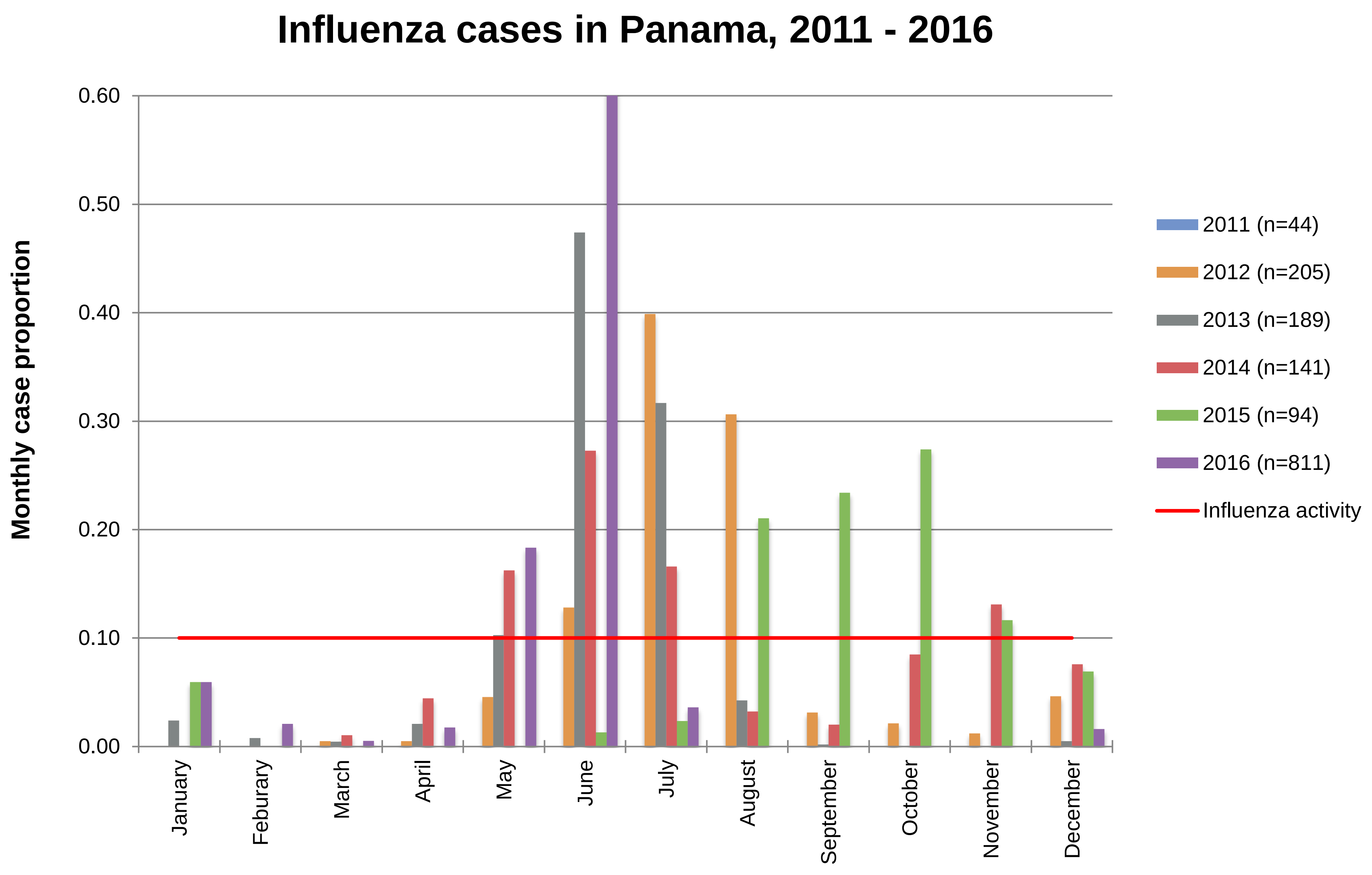

Influenza cases in Panama, 2011 - 2016
Monthly case proportion
0.60
0.50
0.40
0.30
0.20
0.10
0.00
January
Feburary
March
April
May
June
July
August
September
October
November
December
2011 (n=44)
2012 (n=205)
2013 (n=189)
2014 (n=141)
2015 (n=94)
2016 (n=811)
Influenza activity

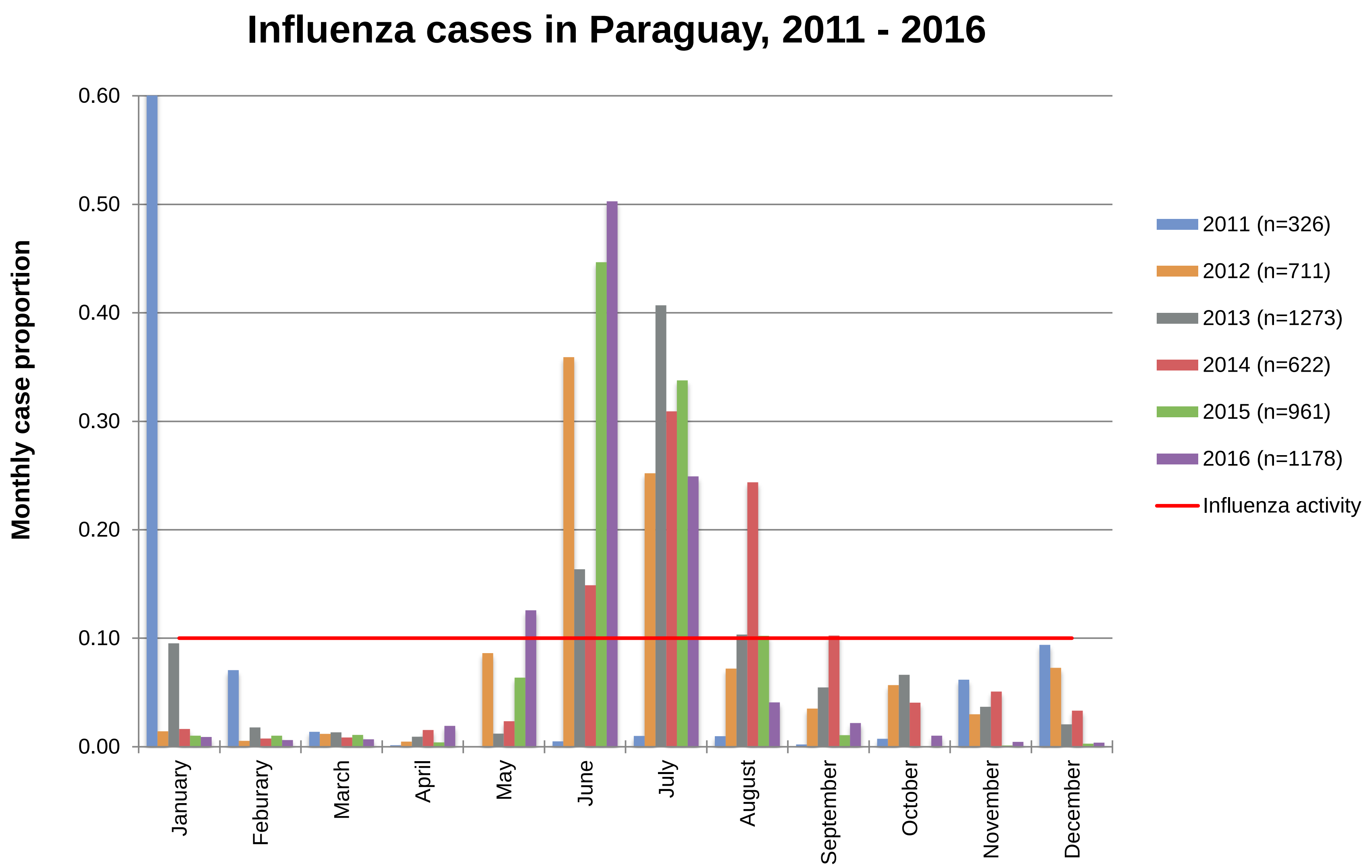

Influenza cases in Paraguay, 2011 - 2016
Monthly case proportion
0.60
0.50
0.40
0.30
0.20
0.10
0.00
January
Feburary
March
April
May
June
July
August
September
October
November
December
2011 (n=326)
2012 (n=711)
2013 (n=1273)
2014 (n=622)
2015 (n=961)
2016 (n=1178)
Influenza activity

Influenza cases in Peru, 2011 - 2016

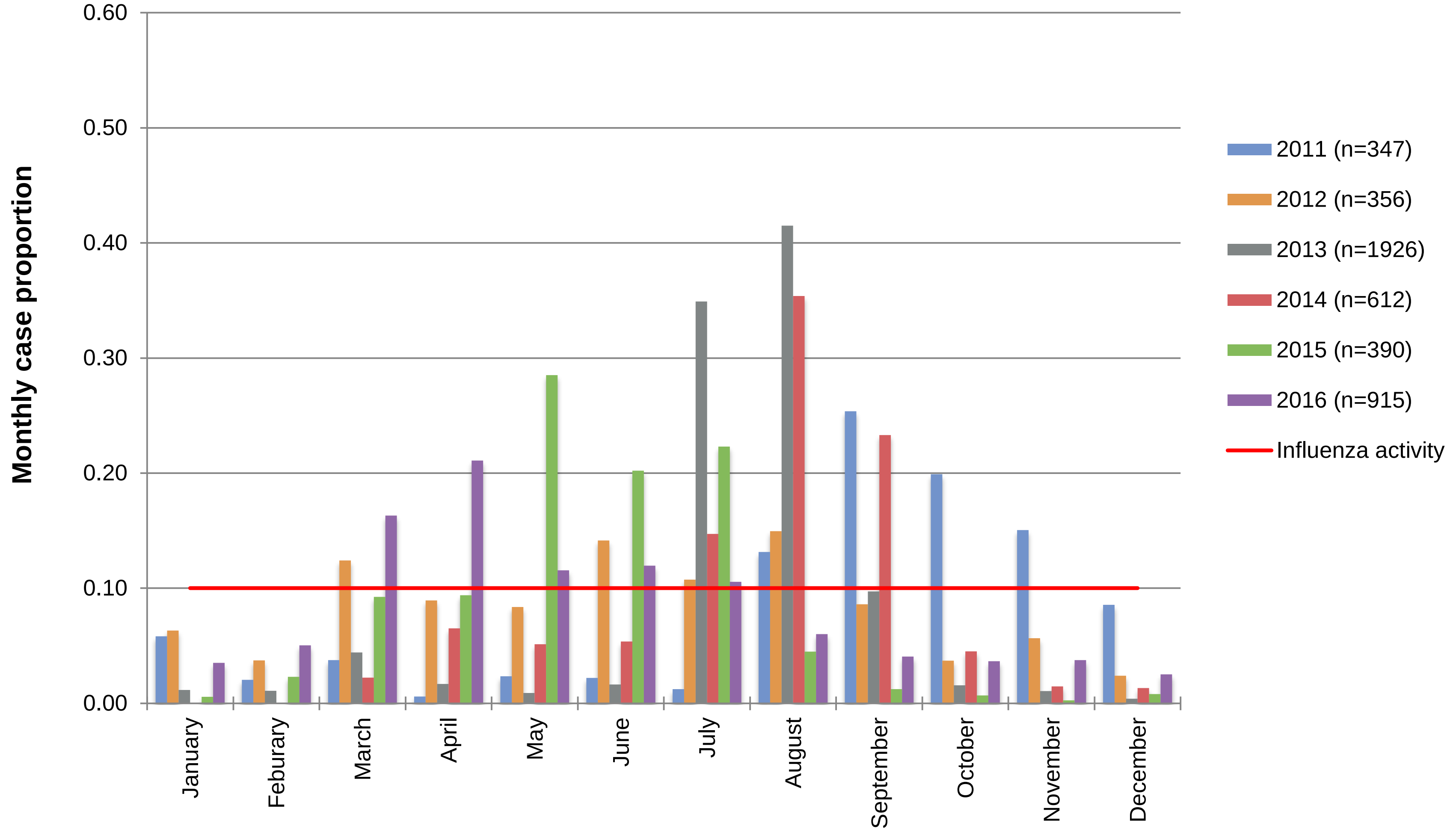

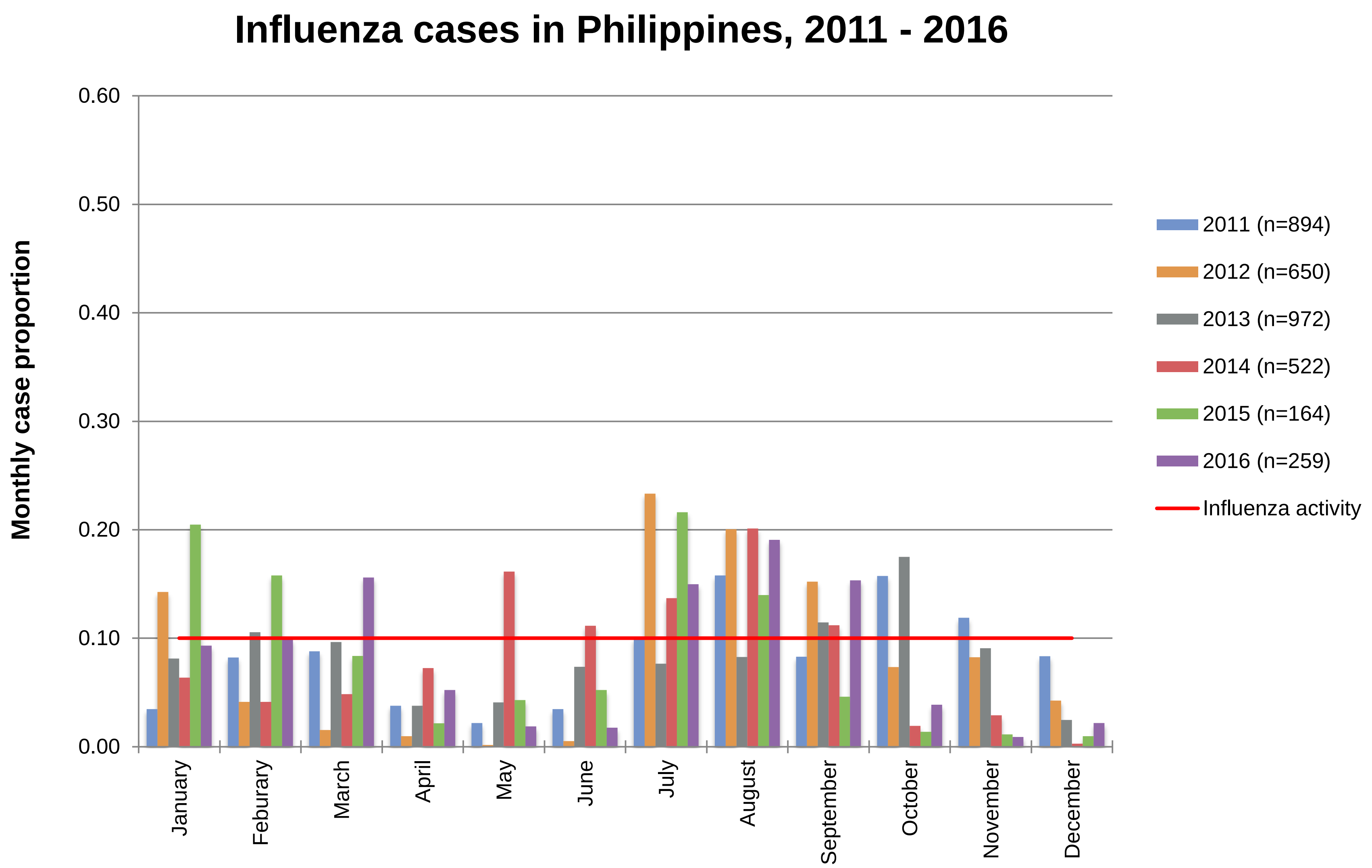

Influenza cases in Philippines, 2011 - 2016
Monthly case proportion
0.60
0.50
0.40
0.30
0.20
0.10
0.00
January
Feburary
March
April
May
June
July
August
September
October
November
December
2011 (n=894)
2012 (n=650)
2013 (n=972)
2014 (n=522)
2015 (n=164)
2016 (n=259)
Influenza activity

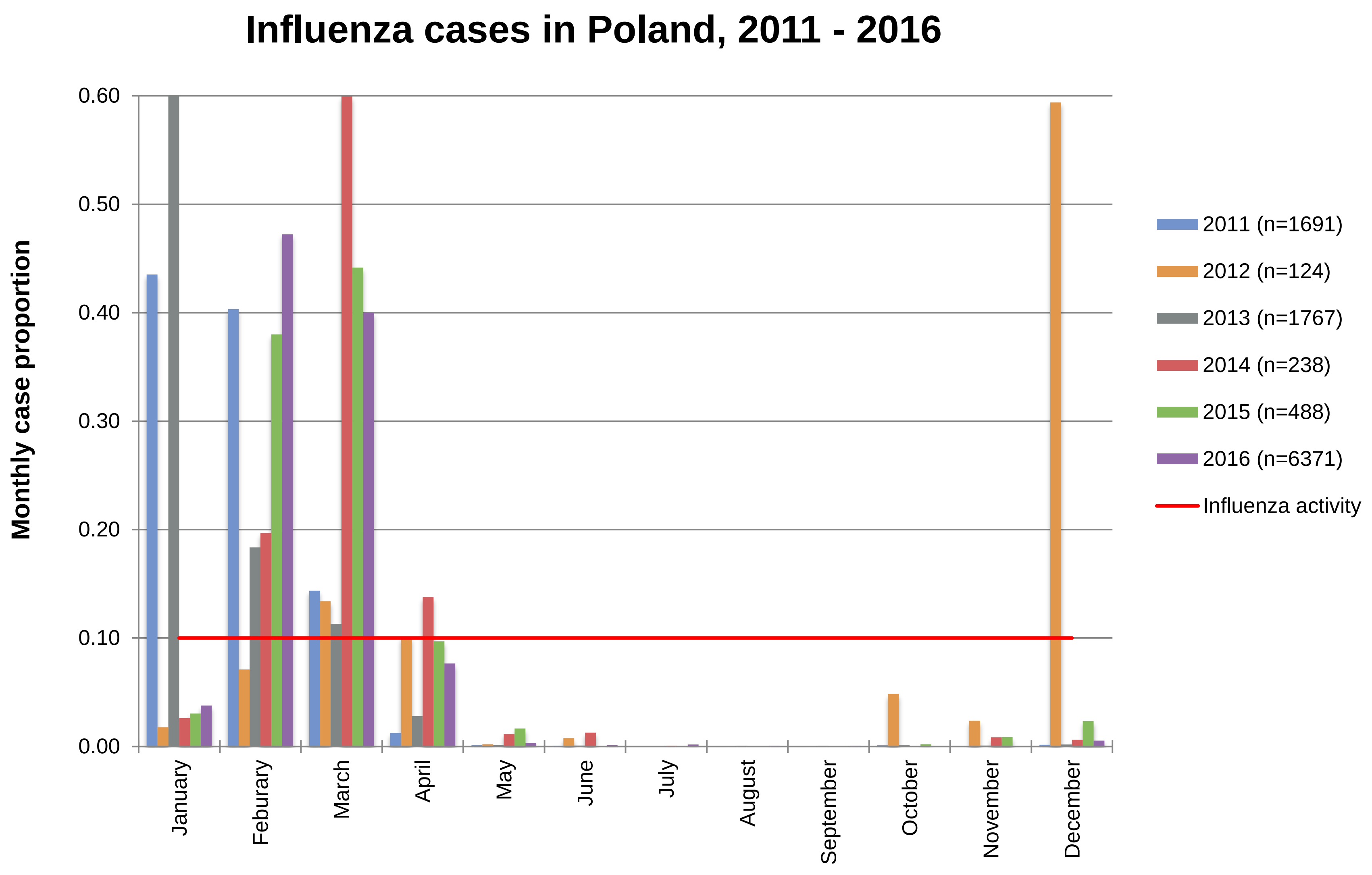

Influenza cases in Poland, 2011 - 2016
Monthly case proportion
0.60
0.50
0.40
0.30
0.20
0.10
0.00
January
Feburary
March
April
May
June
July
August
September
October
November
December
2011 (n=1691)
2012 (n=124)
2013 (n=1767)
2014 (n=238)
2015 (n=488)
2016 (n=6371)
Influenza activity

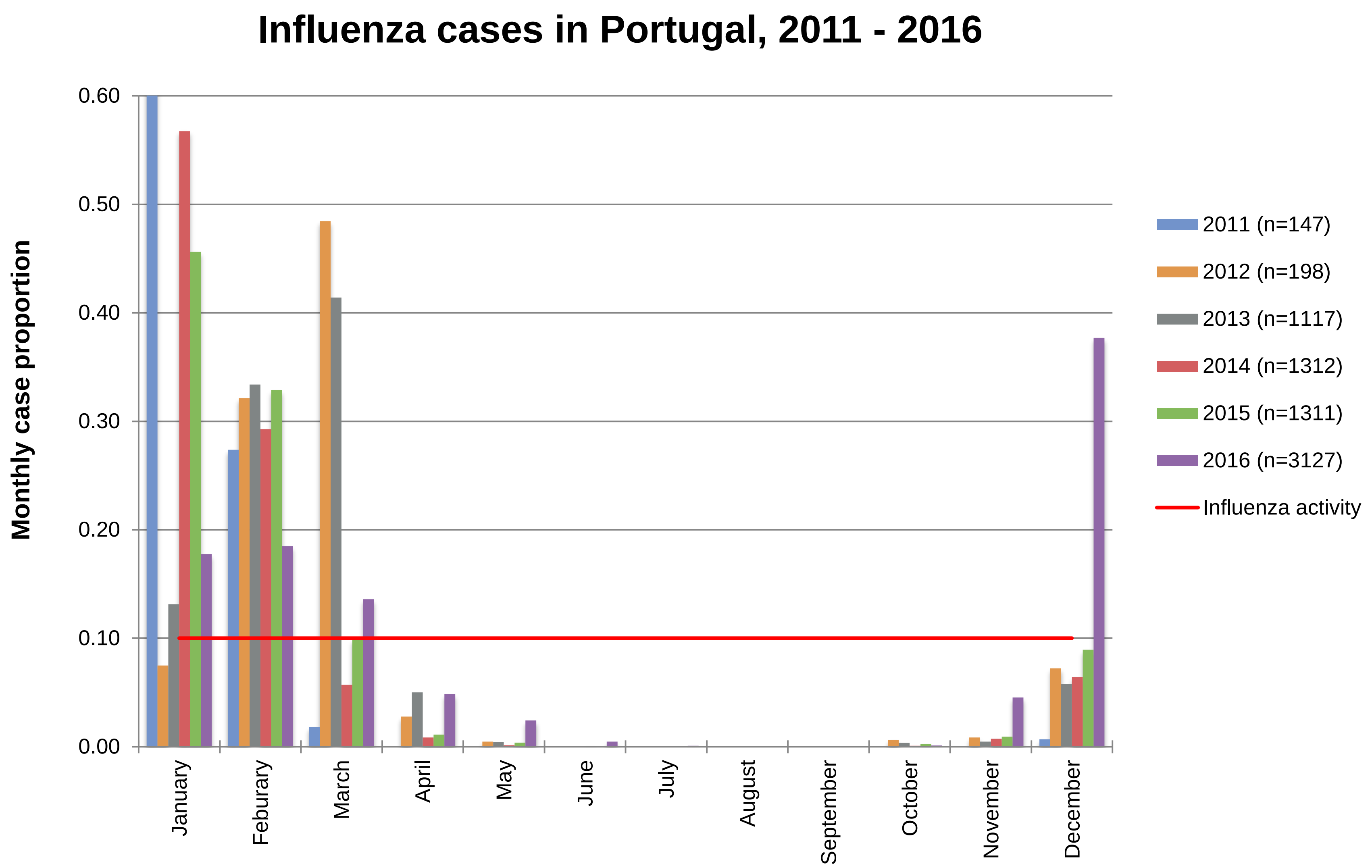
Influenza cases in Portugal, 2011 - 2016
Monthly case proportion
0.60
0.50
0.40
0.30
0.20
0.10
0.00
January
Feburary
March
April
May
June
July
August
September
October
November
December
2011 (n=147)
2012 (n=198)
2013 (n=1117)
2014 (n=1312)
2015 (n=1311)
2016 (n=3127)
Influenza activity

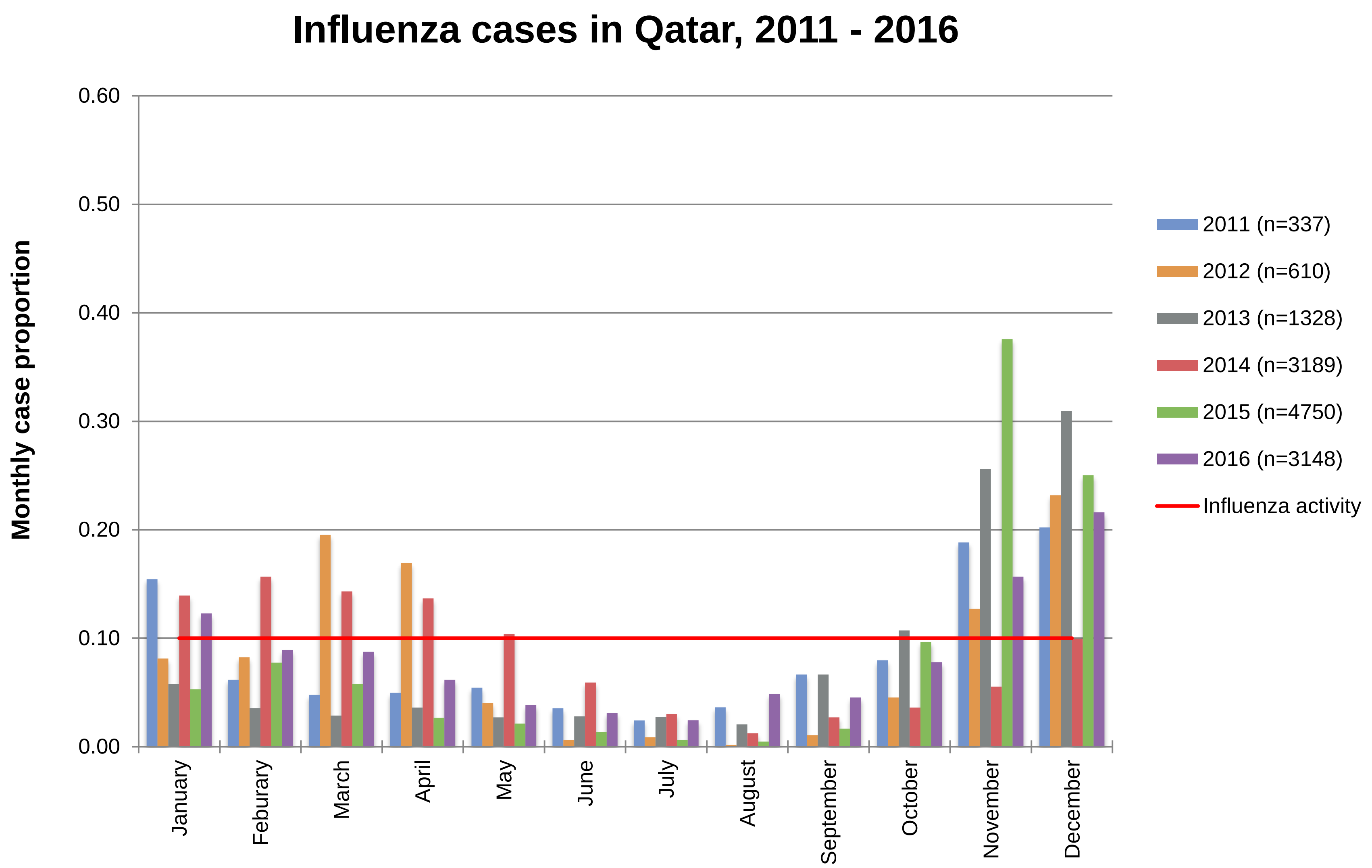
Influenza cases in Qatar, 2011 - 2016
Monthly case proportion
0.60
0.50
0.40
0.30
0.20
0.10
0.00
January
Feburary
March
April
May
June
July
August
September
October
November
December
2011 (n=337)
2012 (n=610)
2013 (n=1328)
2014 (n=3189)
2015 (n=4750)
2016 (n=3148)
Influenza activity

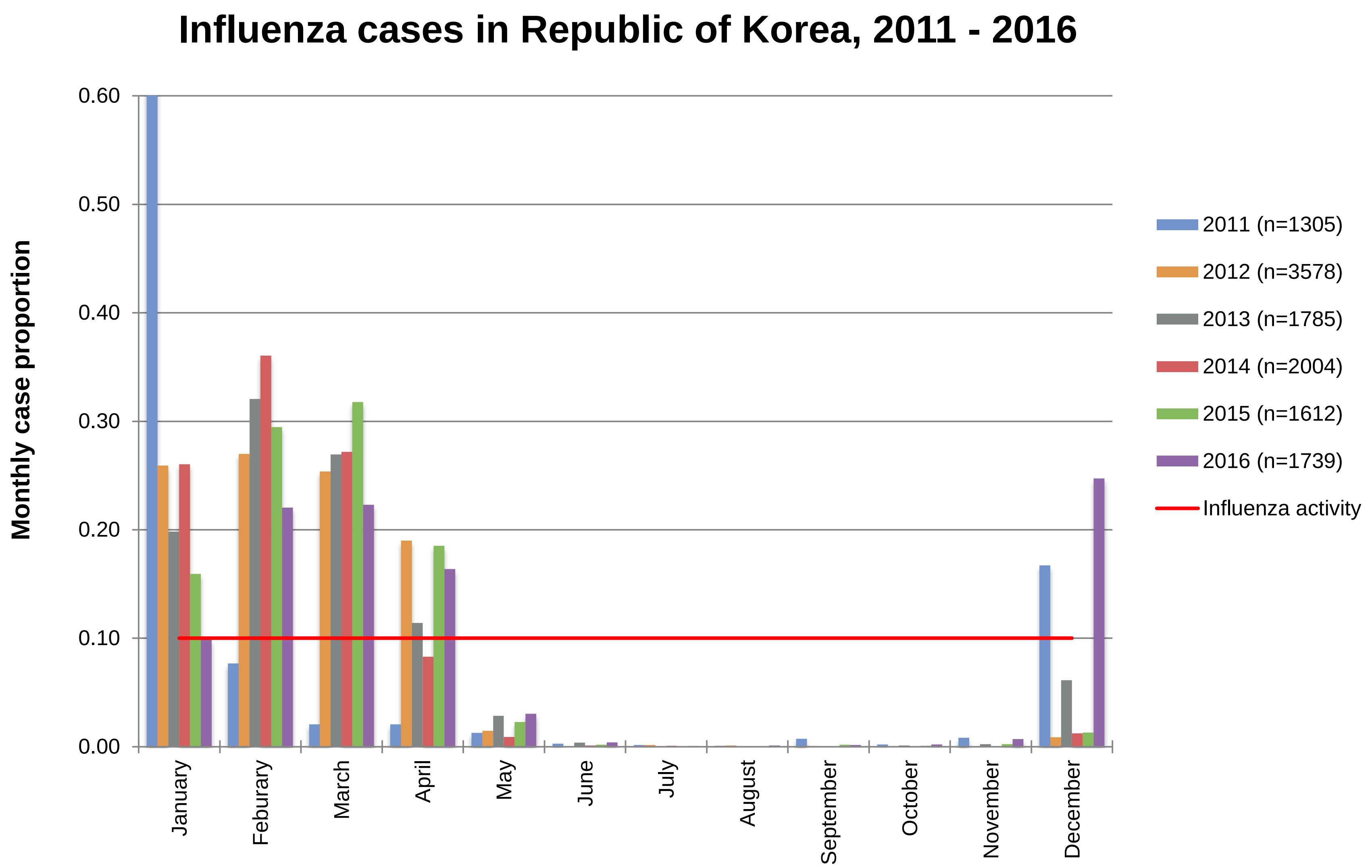
Influenza cases in Republic of Korea, 2011 - 2016
Monthly case proportion
0.60
0.50
0.40
0.30
0.20
0.10
0.00
January
Feburary
March
April
May
June
July
August
September
October
November
December
2011 (n=1305)
2012 (n=3578)
2013 (n=1785)
2014 (n=2004)
2015 (n=1612)
2016 (n=1739)
Influenza activity

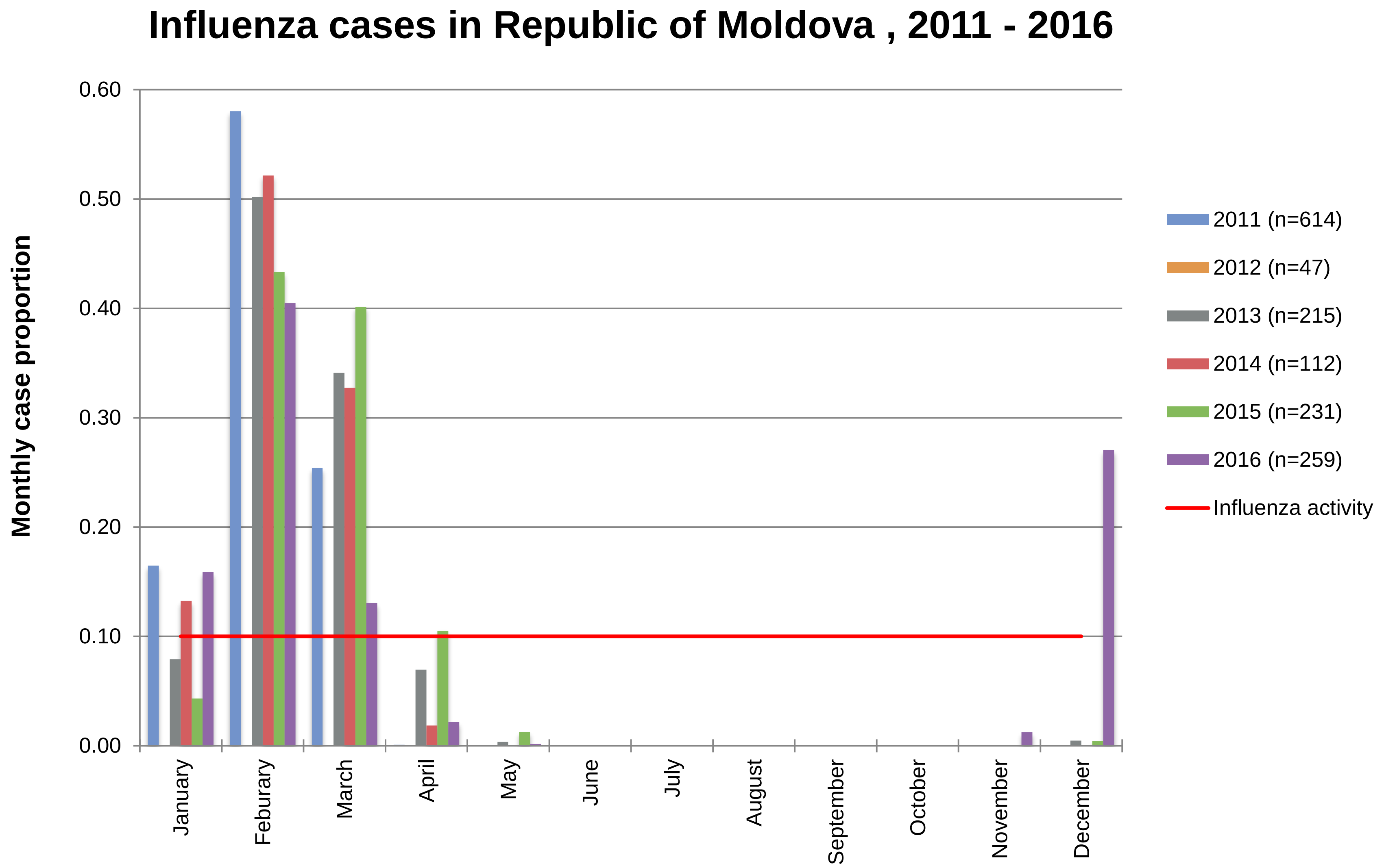
Influenza cases in Republic of Moldova , 2011 - 2016
Monthly case proportion
0.60
0.50
0.40
0.30
0.20
0.10
0.00
January
Feburary
March
April
May
June
July
August
September
October
November
December
2011 (n=614)
2012 (n=47)
2013 (n=215)
2014 (n=112)
2015 (n=231)
2016 (n=259)
Influenza activity

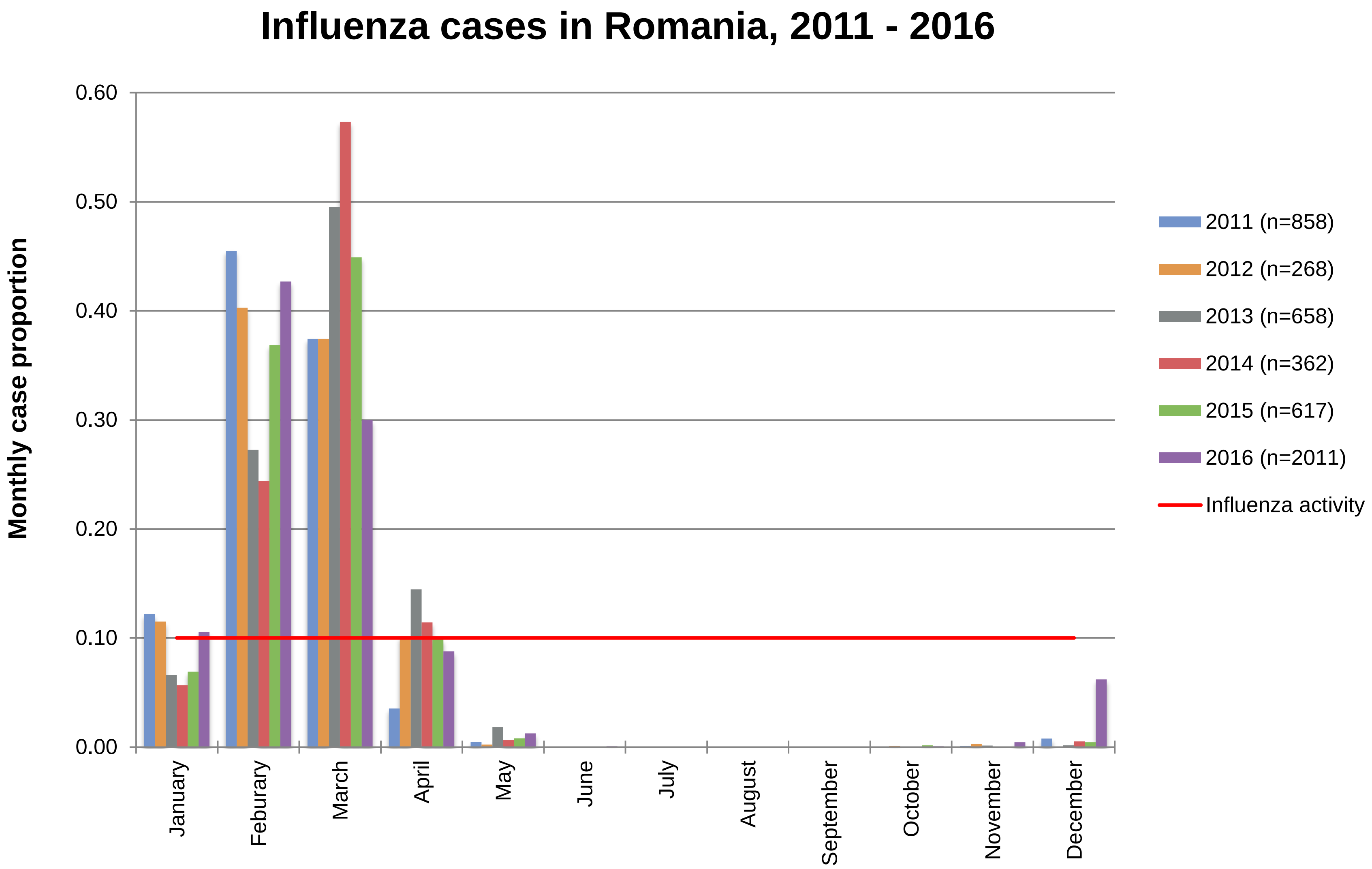

Influenza cases in Romania, 2011 - 2016
Monthly case proportion
0.60
0.50
0.40
0.30
0.20
0.10
0.00
January
Feburary
March
April
May
June
July
August
September
October
November
December
2011 (n=858)
2012 (n=268)
2013 (n=658)
2014 (n=362)
2015 (n=617)
2016 (n=2011)
Influenza activity

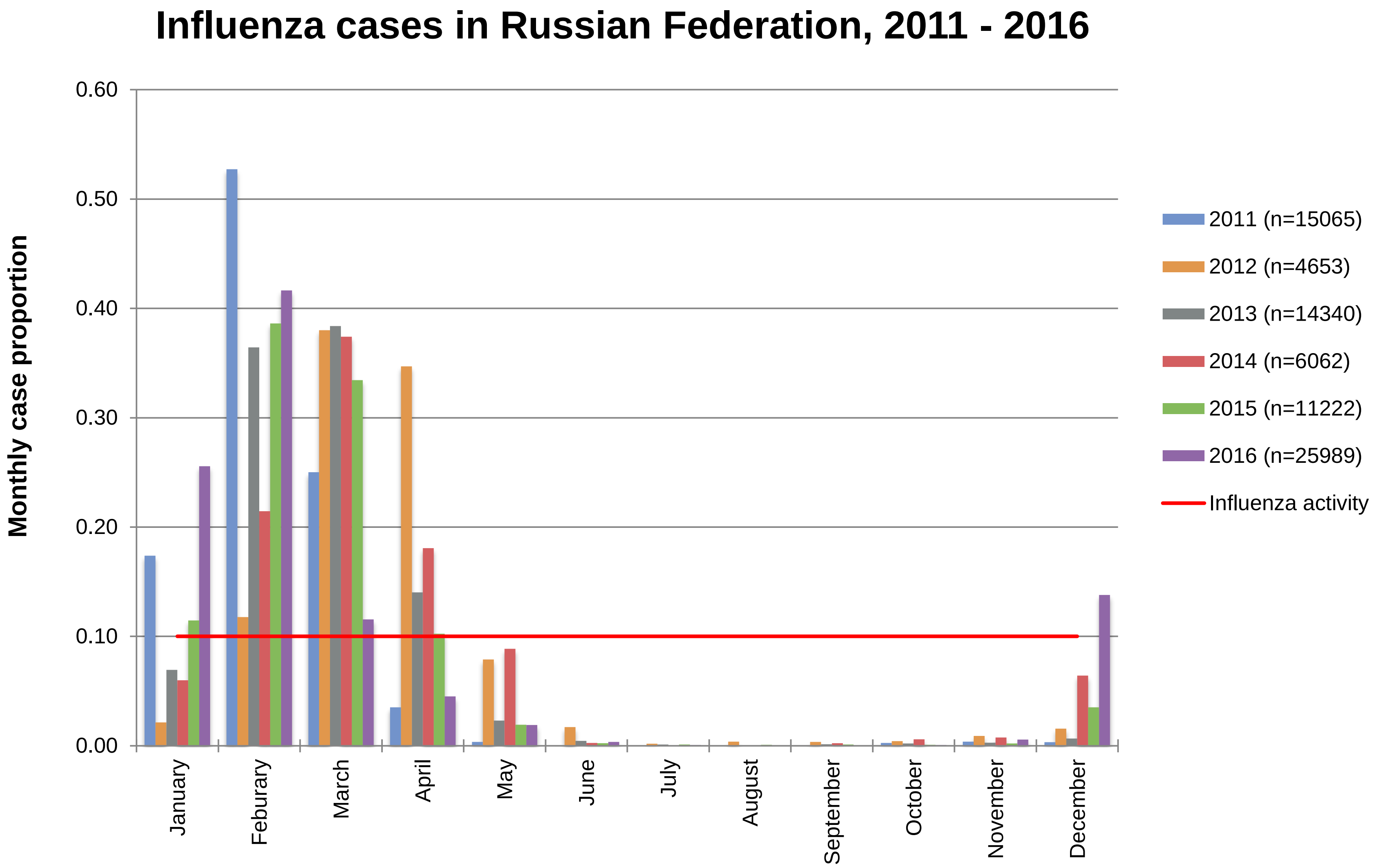
Influenza cases in Russian Federation, 2011 - 2016
Monthly case proportion
0.60
0.50
0.40
0.30
0.20
0.10
0.00
January
Feburary
March
April
May
June
July
August
September
October
November
December
2011 (n=15065)
2012 (n=4653)
2013 (n=14340)
2014 (n=6062)
2015 (n=11222)
2016 (n=25989)
Influenza activity

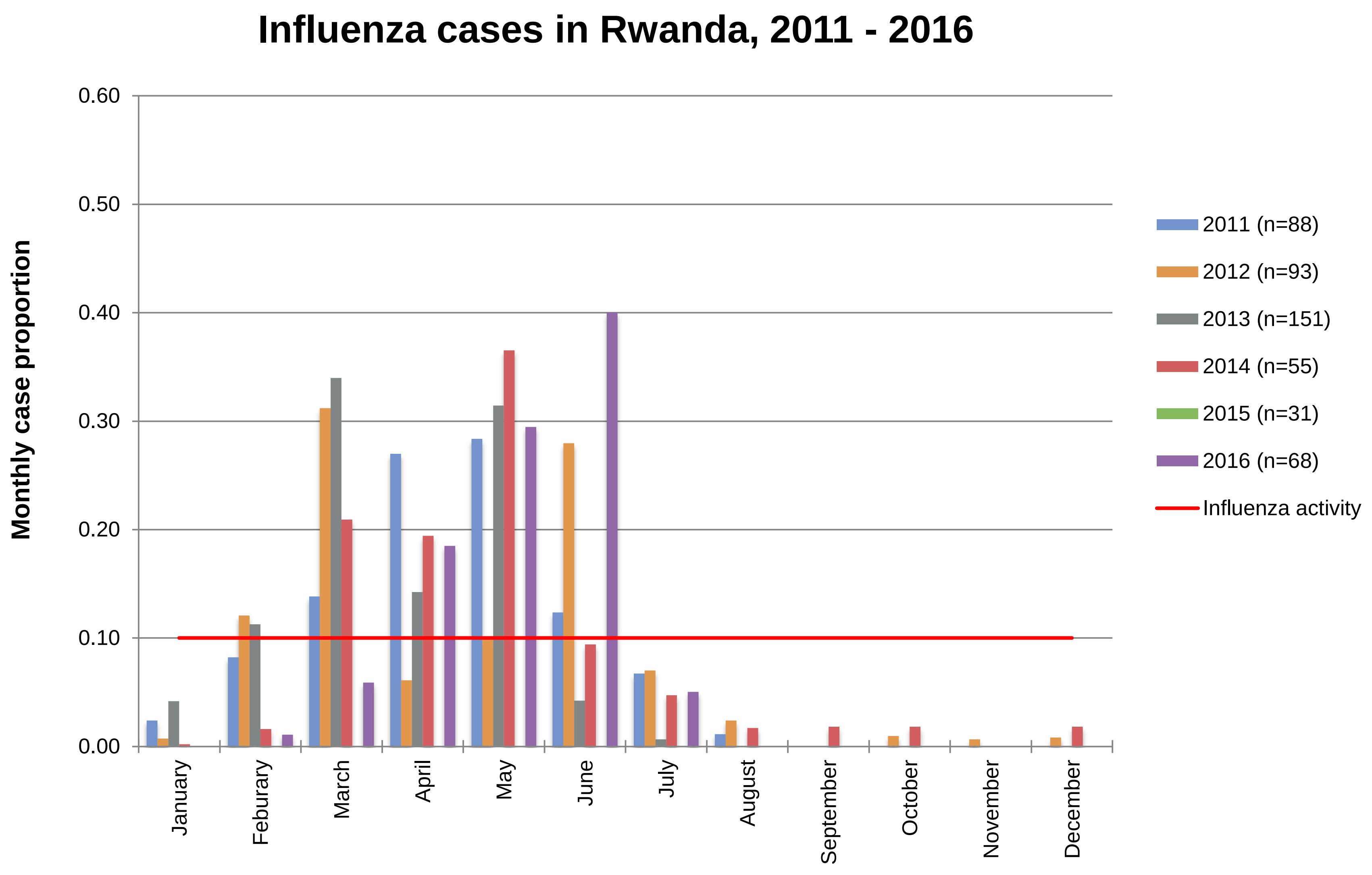
Influenza cases in Rwanda, 2011 - 2016
Monthly case proportion
0.60
0.50
0.40
0.30
0.20
0.10
0.00
January
Feburary
March
April
May
June
July
August
September
October
November
December
2011 (n=88)
2012 (n=93)
2013 (n=151)
2014 (n=55)
2015 (n=31)
2016 (n=68)
Influenza activity

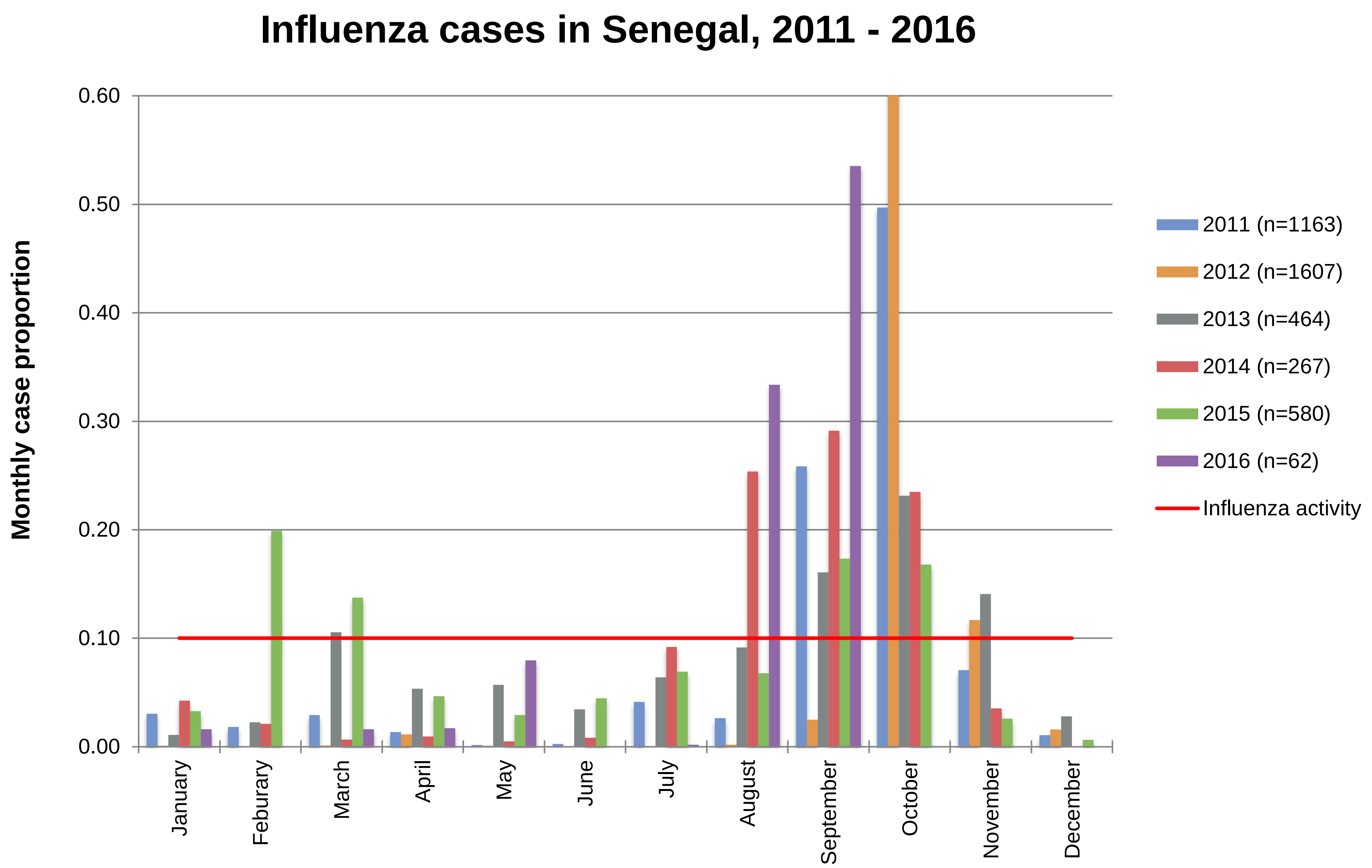

Influenza cases in Senegal, 2011 - 2016
Monthly case proportion
0.60
0.50
0.40
0.30
0.20
0.10
0.00
January
Feburary
March
April
May
June
July
August
September
October
November
December
2011 (n=1163)
2012 (n=1607)
2013 (n=464)
2014 (n=267)
2015 (n=580)
2016 (n=62)
Influenza activity

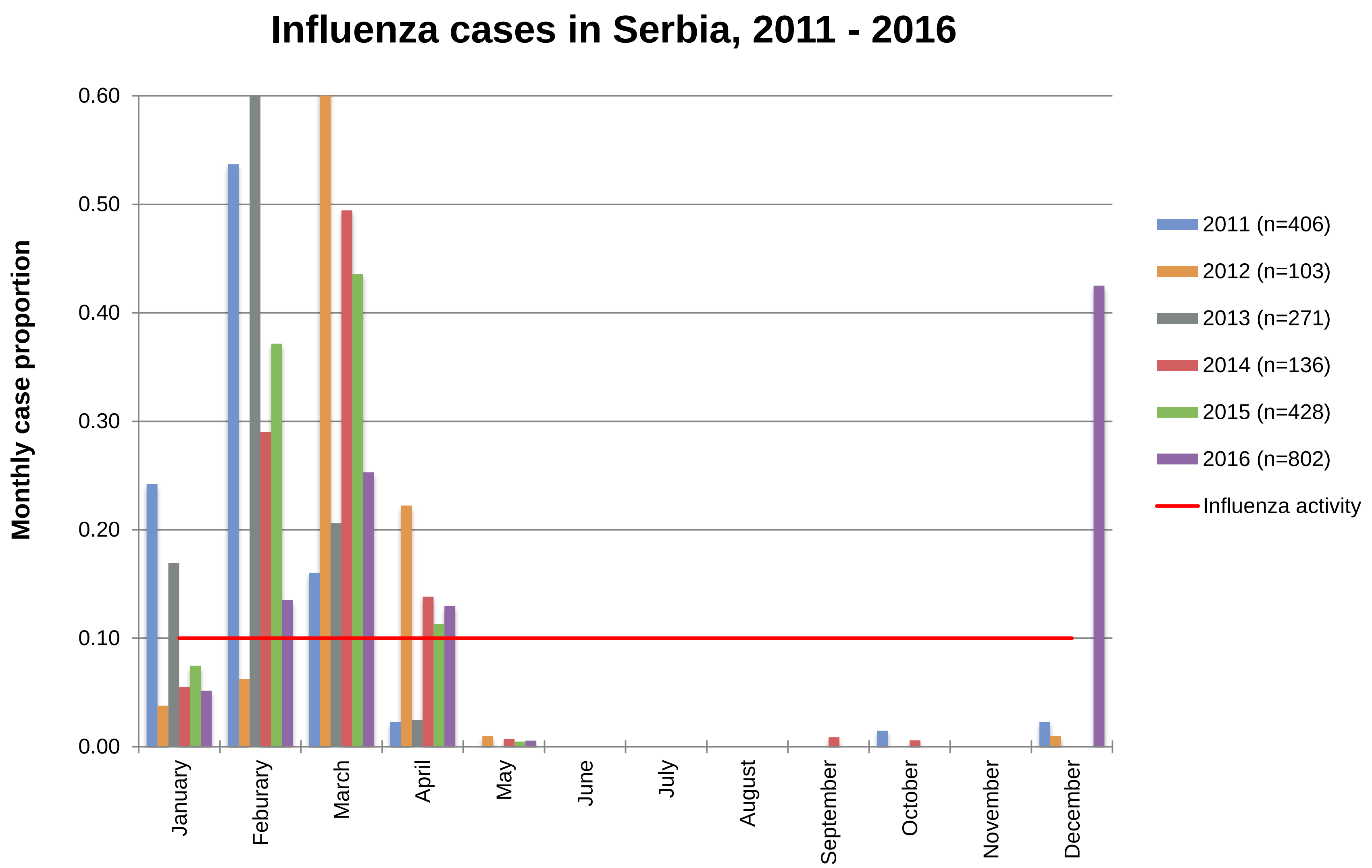
Influenza cases in Serbia, 2011 - 2016
Monthly case proportion
0.60
0.50
0.40
0.30
0.20
0.10
0.00
January
Feburary
March
April
May
June
July
August
September
October
November
December
2011 (n=406)
2012 (n=103)
2013 (n=271)
2014 (n=136)
2015 (n=428)
2016 (n=802)
Influenza activity

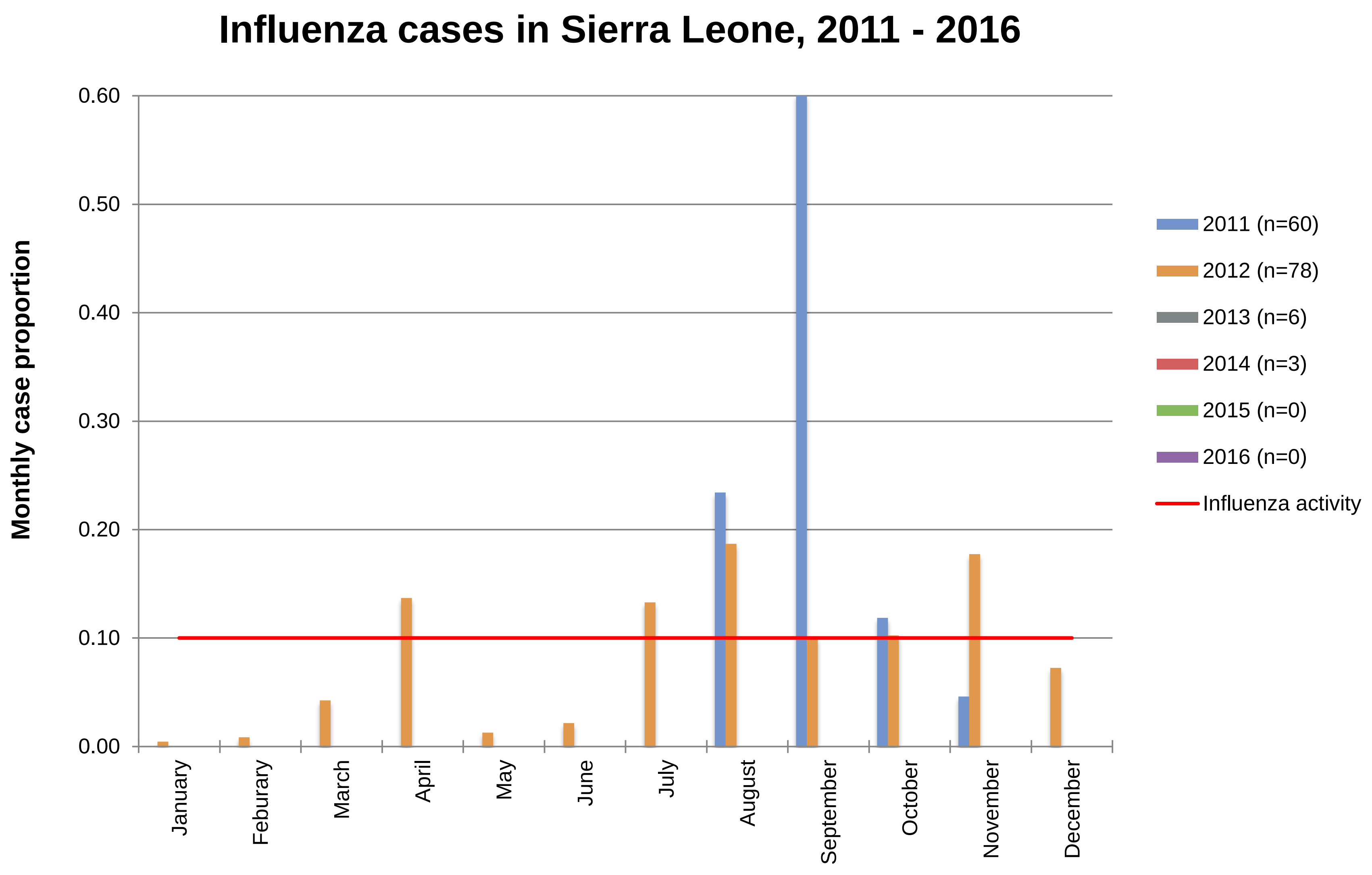

Influenza cases in Sierra Leone, 2011 - 2016
Monthly case proportion
0.60
0.50
0.40
0.30
0.20
0.10
0.00
January
Feburary
March
April
May
June
July
August
September
October
November
December
2011 (n=60)
2012 (n=78)
2013 (n=6)
2014 (n=3)
2015 (n=0)
2016 (n=0)
Influenza activity

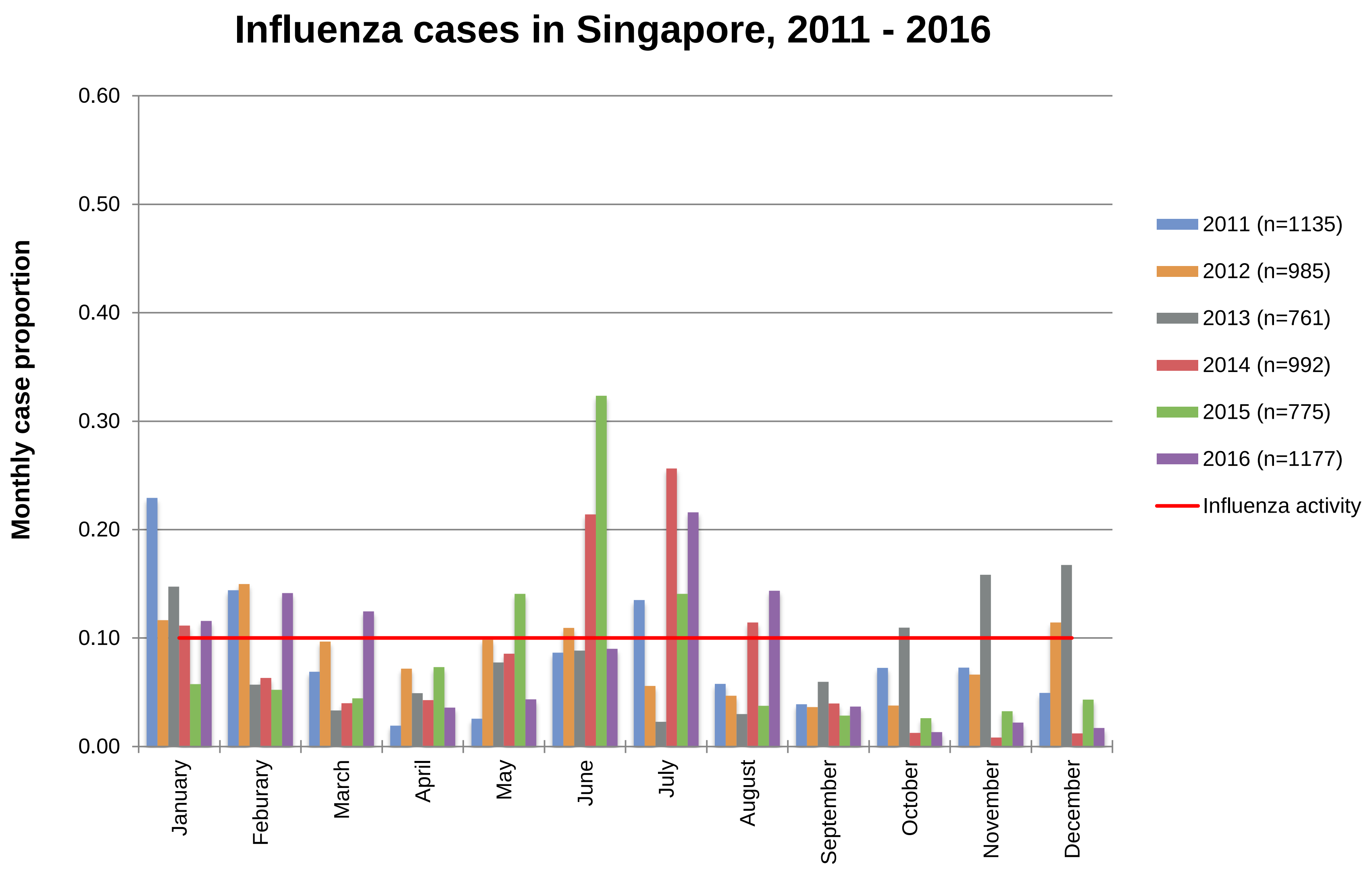

Influenza cases in Singapore, 2011 - 2016
Monthly case proportion
0.60
0.50
0.40
0.30
0.20
0.10
0.00
January
Feburary
March
April
May
June
July
August
September
October
November
December
2011 (n=1135)
2012 (n=985)
2013 (n=761)
2014 (n=992)
2015 (n=775)
2016 (n=1177)
Influenza activity

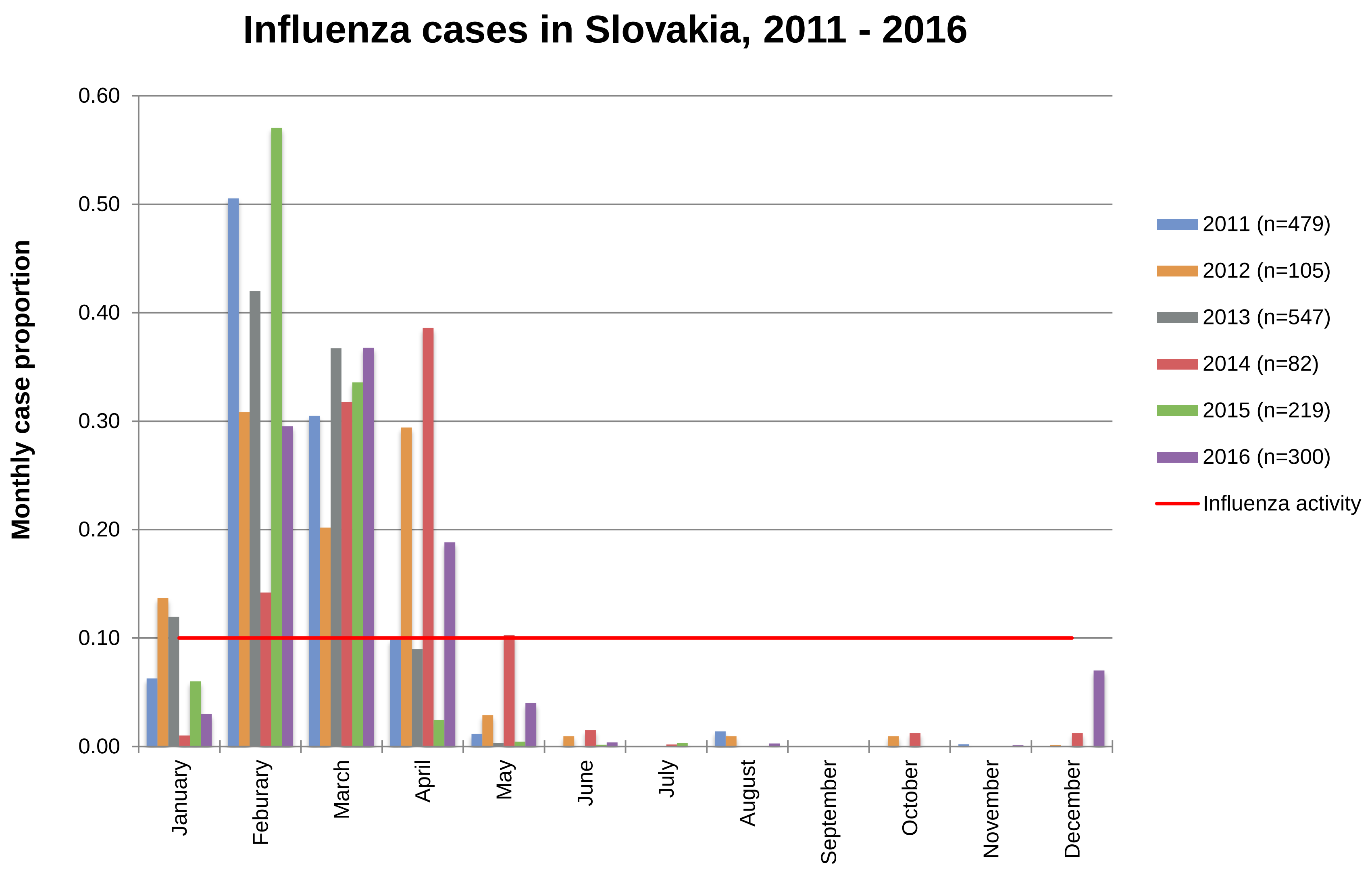

Influenza cases in Slovakia, 2011 - 2016
Monthly case proportion
0.60
0.50
0.40
0.30
0.20
0.10
0.00
January
Feburary
March
April
May
June
July
August
September
October
November
December
2011 (n=479)
2012 (n=105)
2013 (n=547)
2014 (n=82)
2015 (n=219)
2016 (n=300)
Influenza activity

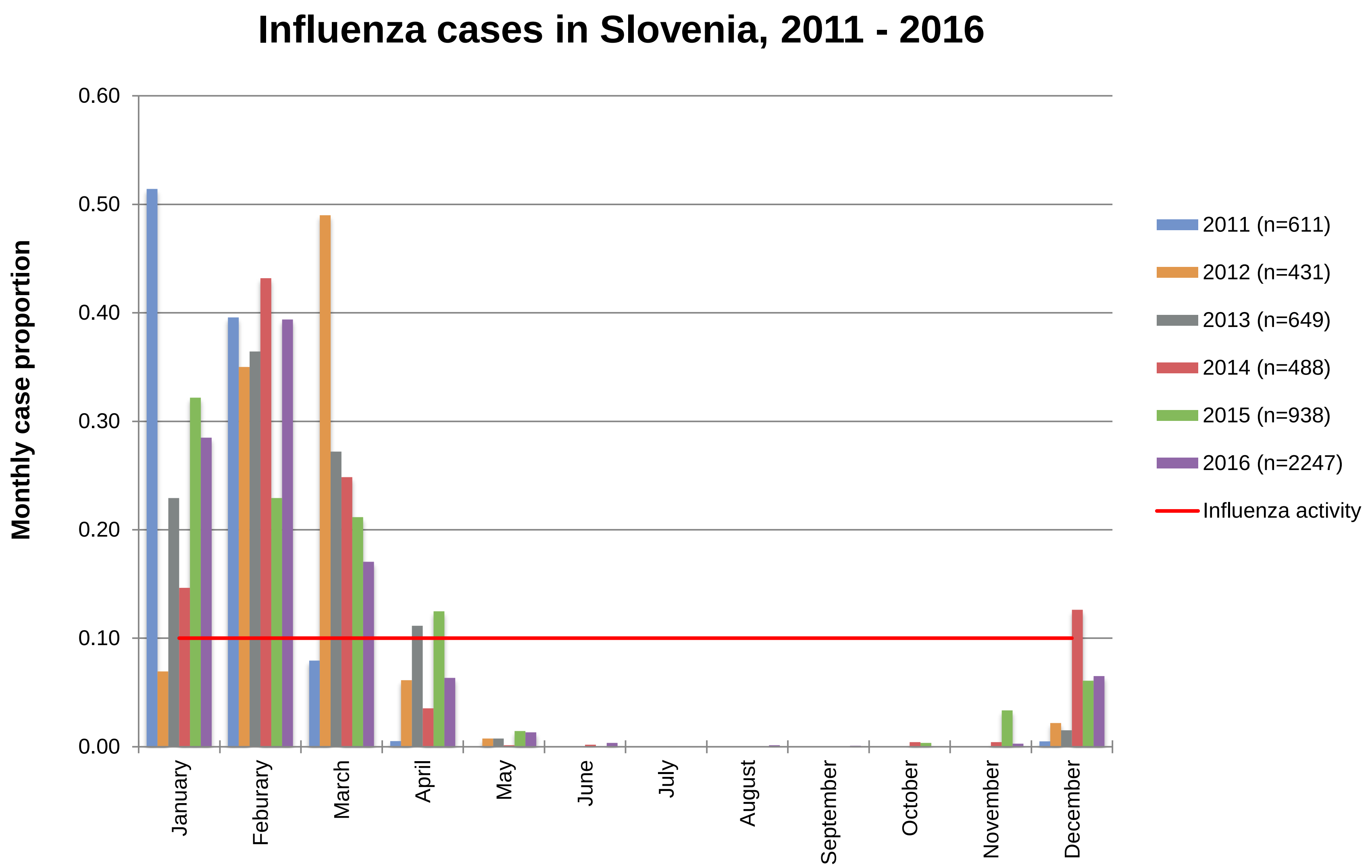
Influenza cases in Slovenia, 2011 - 2016
Monthly case proportion
0.60
0.50
0.40
0.30
0.20
0.10
0.00
January
Feburary
March
April
May
June
July
August
September
October
November
December
2011 (n=611)
2012 (n=431)
2013 (n=649)
2014 (n=488)
2015 (n=938)
2016 (n=2247)
Influenza activity

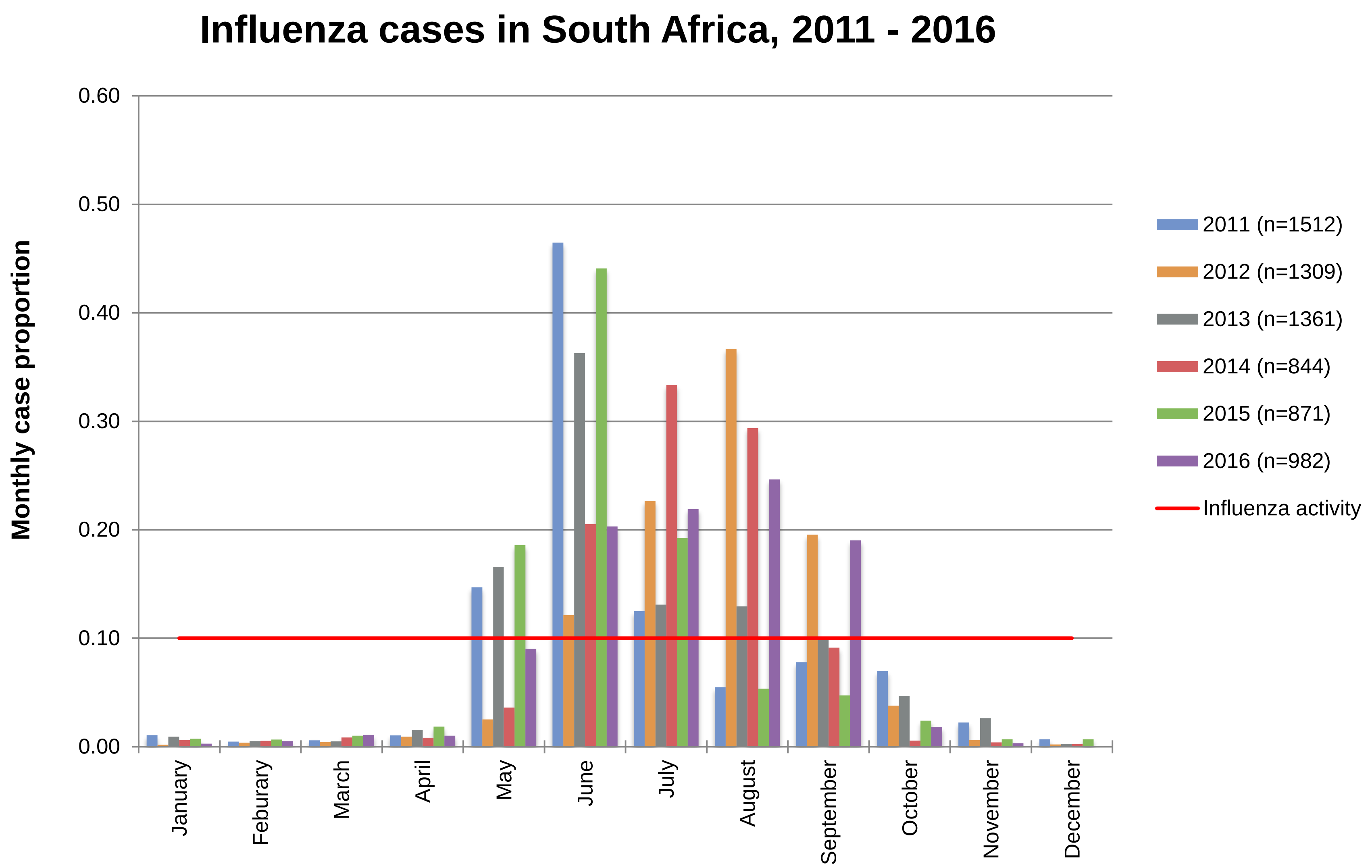

Influenza cases in South Africa, 2011 - 2016
Monthly case proportion
0.60
0.50
0.40
0.30
0.20
0.10
0.00
January
Feburary
March
April
May
June
July
August
September
October
November
December
2011 (n=1512)
2012 (n=1309)
2013 (n=1361)
2014 (n=844)
2015 (n=871)
2016 (n=982)
Influenza activity

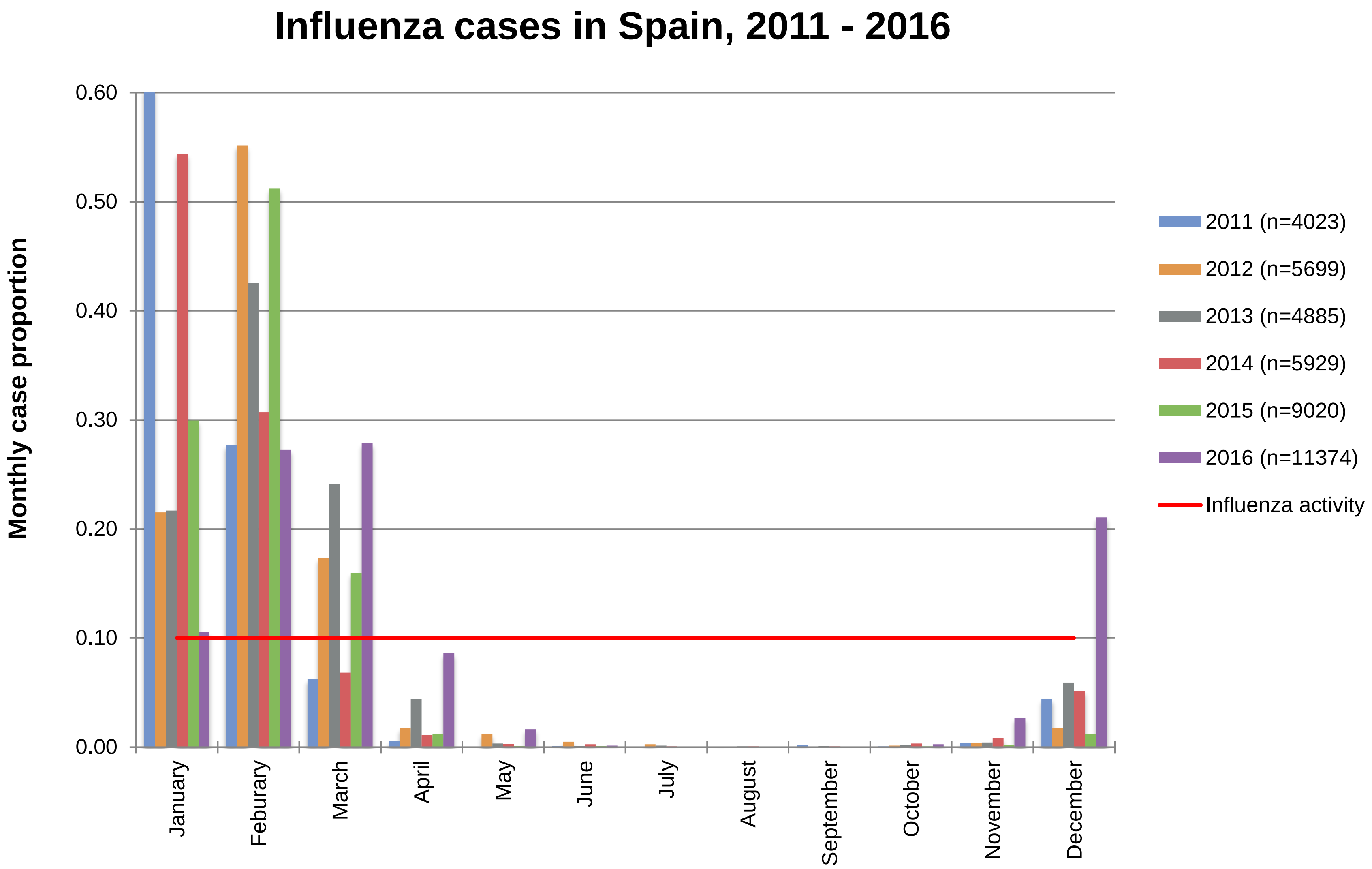
Influenza cases in Spain, 2011 - 2016
Monthly case proportion
0.60
0.50
0.40
0.30
0.20
0.10
0.00
January
Feburary
March
April
May
June
July
August
September
October
November
December
2011 (n=4023)
2012 (n=5699)
2013 (n=4885)
2014 (n=5929)
2015 (n=9020)
2016 (n=11374)
Influenza activity

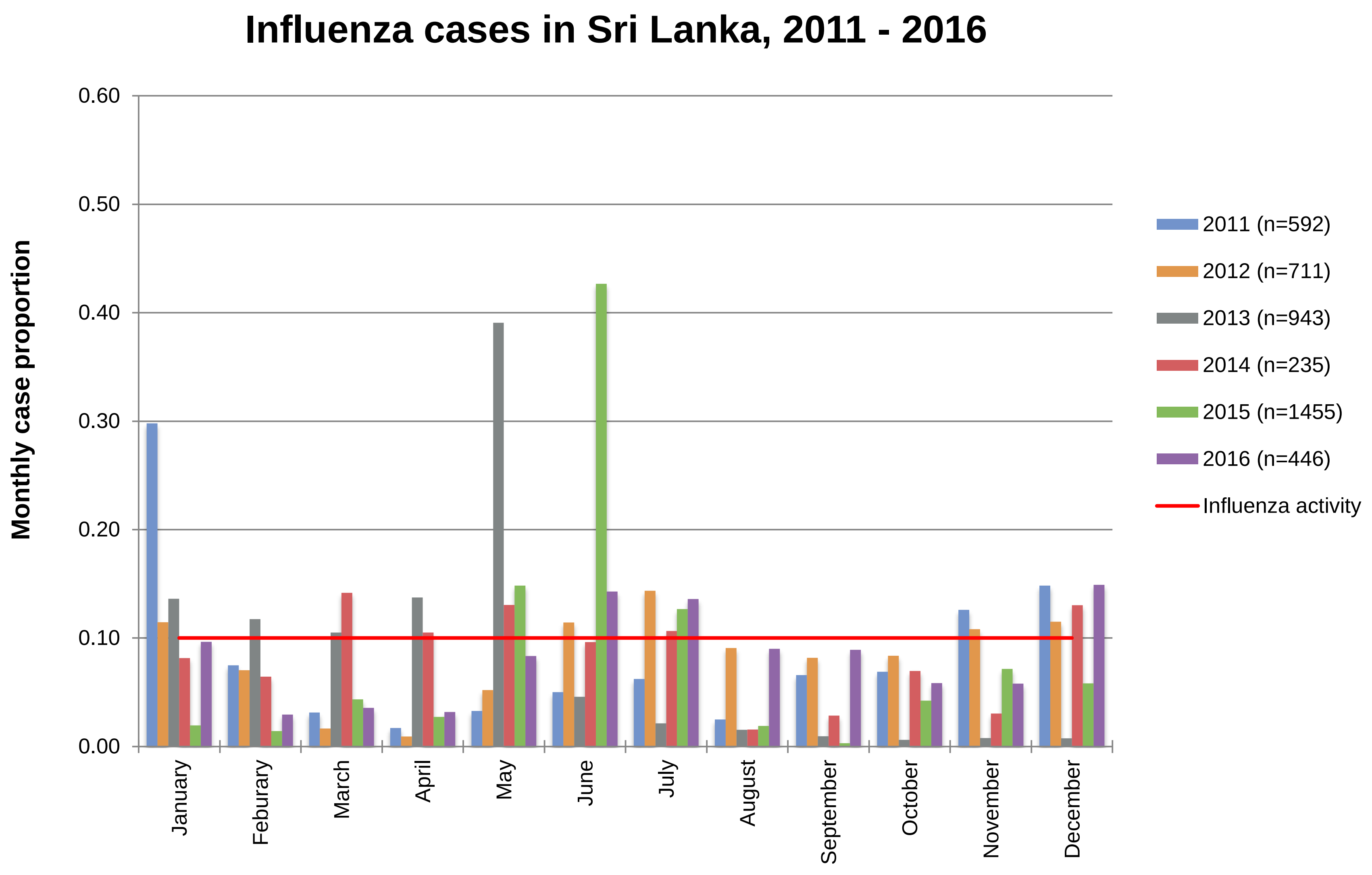
Influenza cases in Sri Lanka, 2011 - 2016
Monthly case proportion
0.60
0.50
0.40
0.30
0.20
0.10
0.00
January
Feburary
March
April
May
June
July
August
September
October
November
December
2011 (n=592)
2012 (n=711)
2013 (n=943)
2014 (n=235)
2015 (n=1455)
2016 (n=446)
Influenza activity

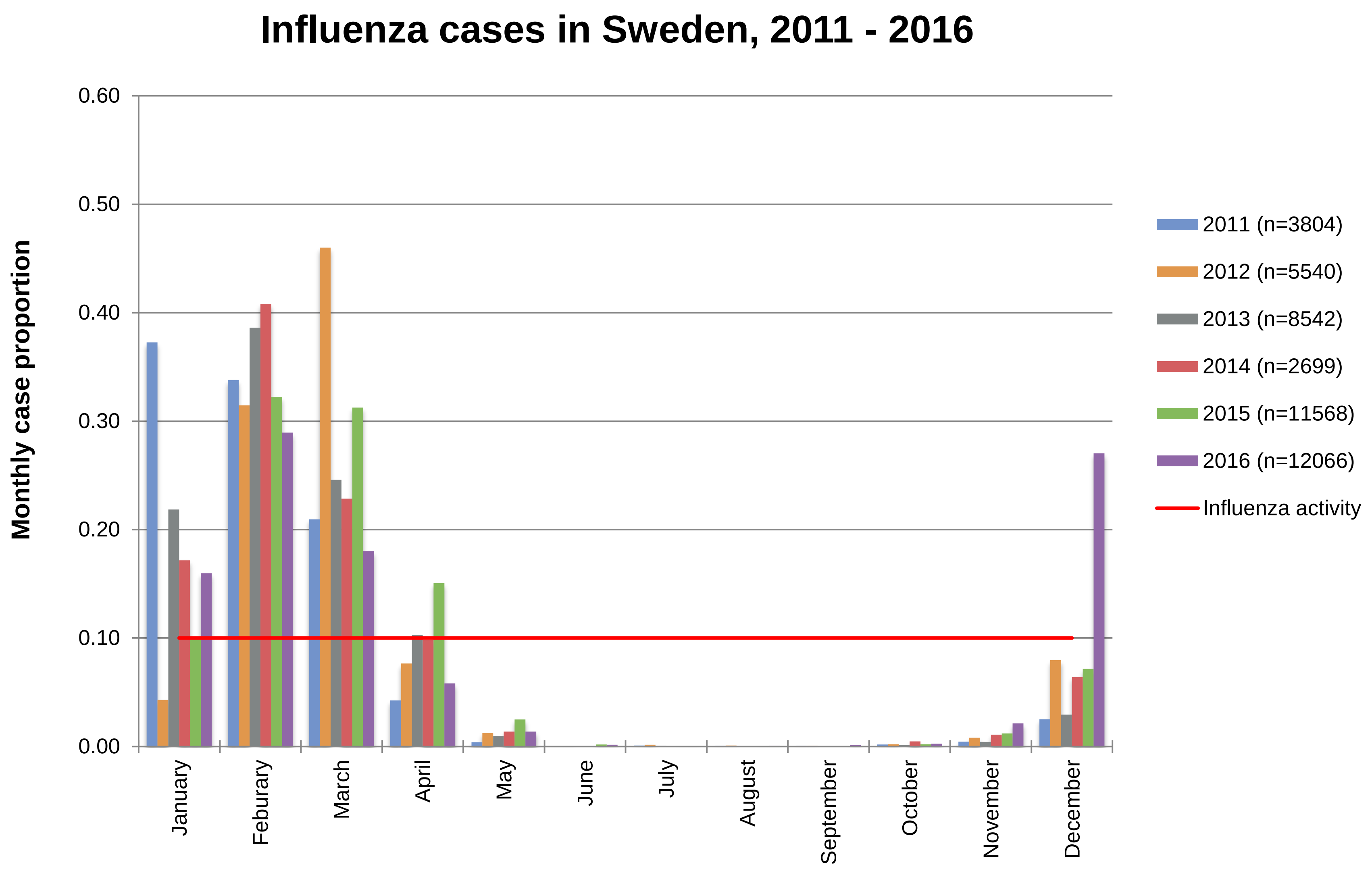

Influenza cases in Sweden, 2011 - 2016
Monthly case proportion
0.60
0.50
0.40
0.30
0.20
0.10
0.00
January
Feburary
March
April
May
June
July
August
September
October
November
December
2011 (n=3804)
2012 (n=5540)
2013 (n=8542)
2014 (n=2699)
2015 (n=11568)
2016 (n=12066)
Influenza activity

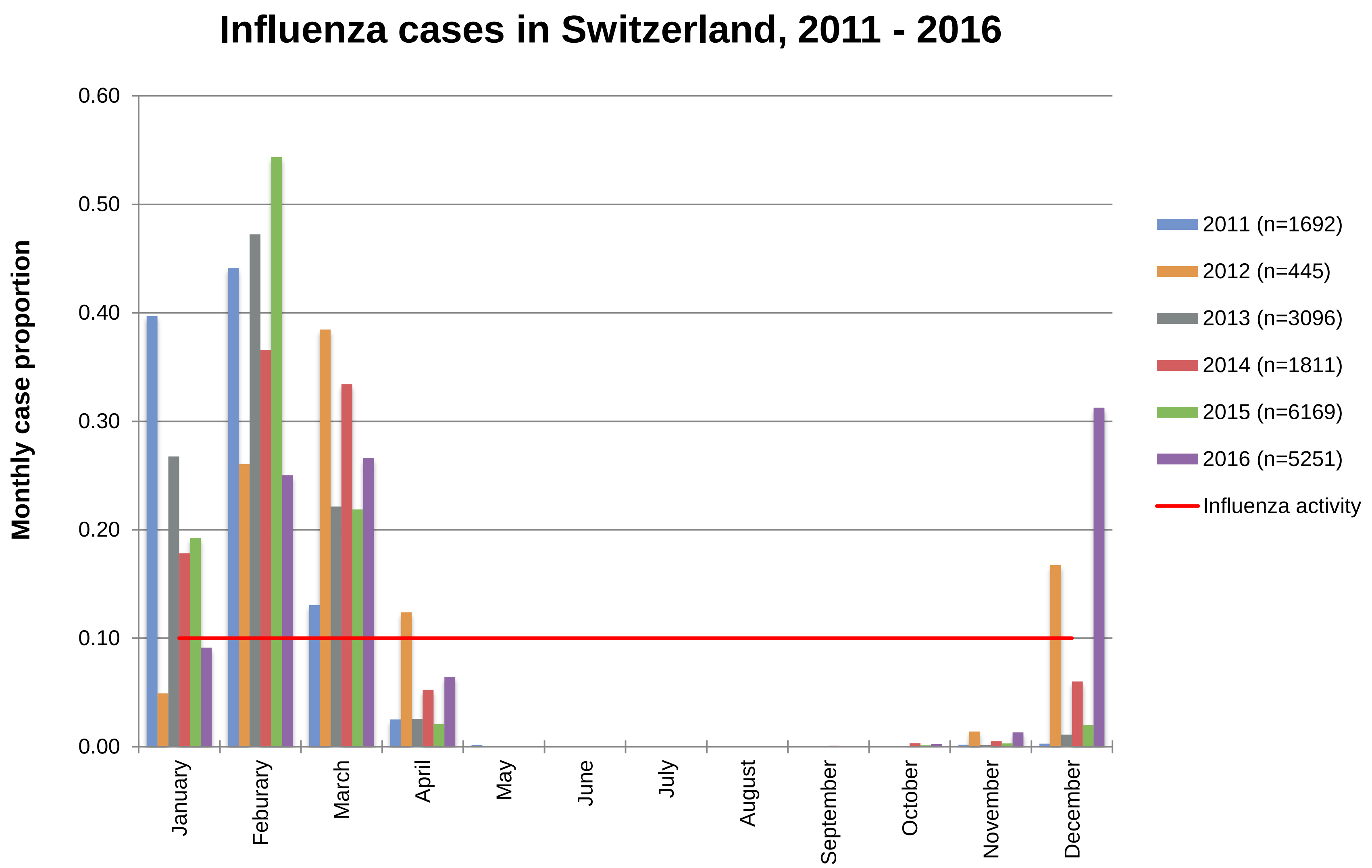
Influenza cases in Switzerland, 2011 - 2016
Monthly case proportion
0.60
0.50
0.40
0.30
0.20
0.10
0.00
January
Feburary
March
April
May
June
July
August
September
October
November
December
2011 (n=1692)
2012 (n=445)
2013 (n=3096)
2014 (n=1811)
2015 (n=6169)
2016 (n=5251)
Influenza activity

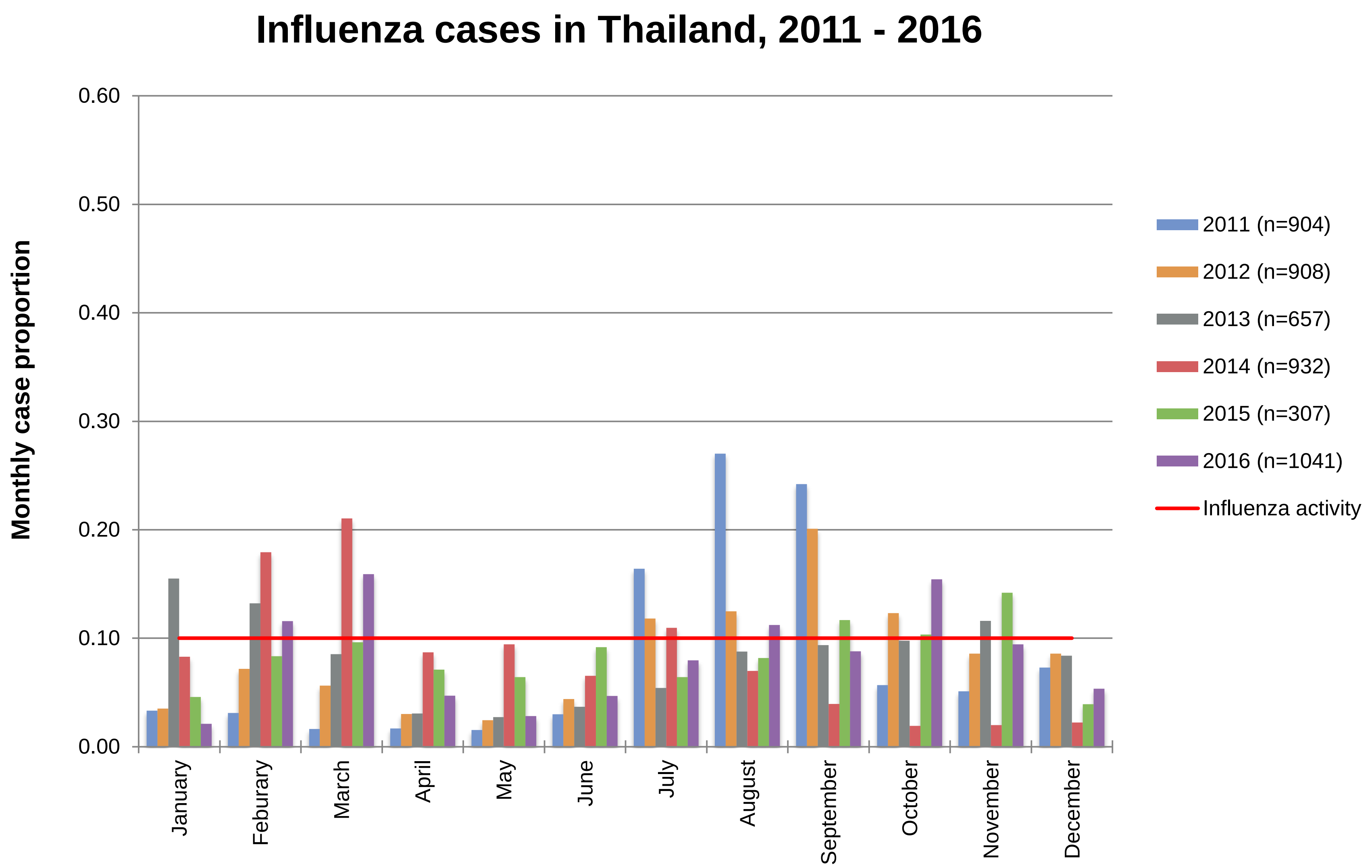

Influenza cases in Thailand, 2011 - 2016
Monthly case proportion
0.60
0.50
0.40
0.30
0.20
0.10
0.00
January
Feburary
March
April
May
June
July
August
September
October
November
December
2011 (n=904)
2012 (n=908)
2013 (n=657)
2014 (n=932)
2015 (n=307)
2016 (n=1041)
Influenza activity

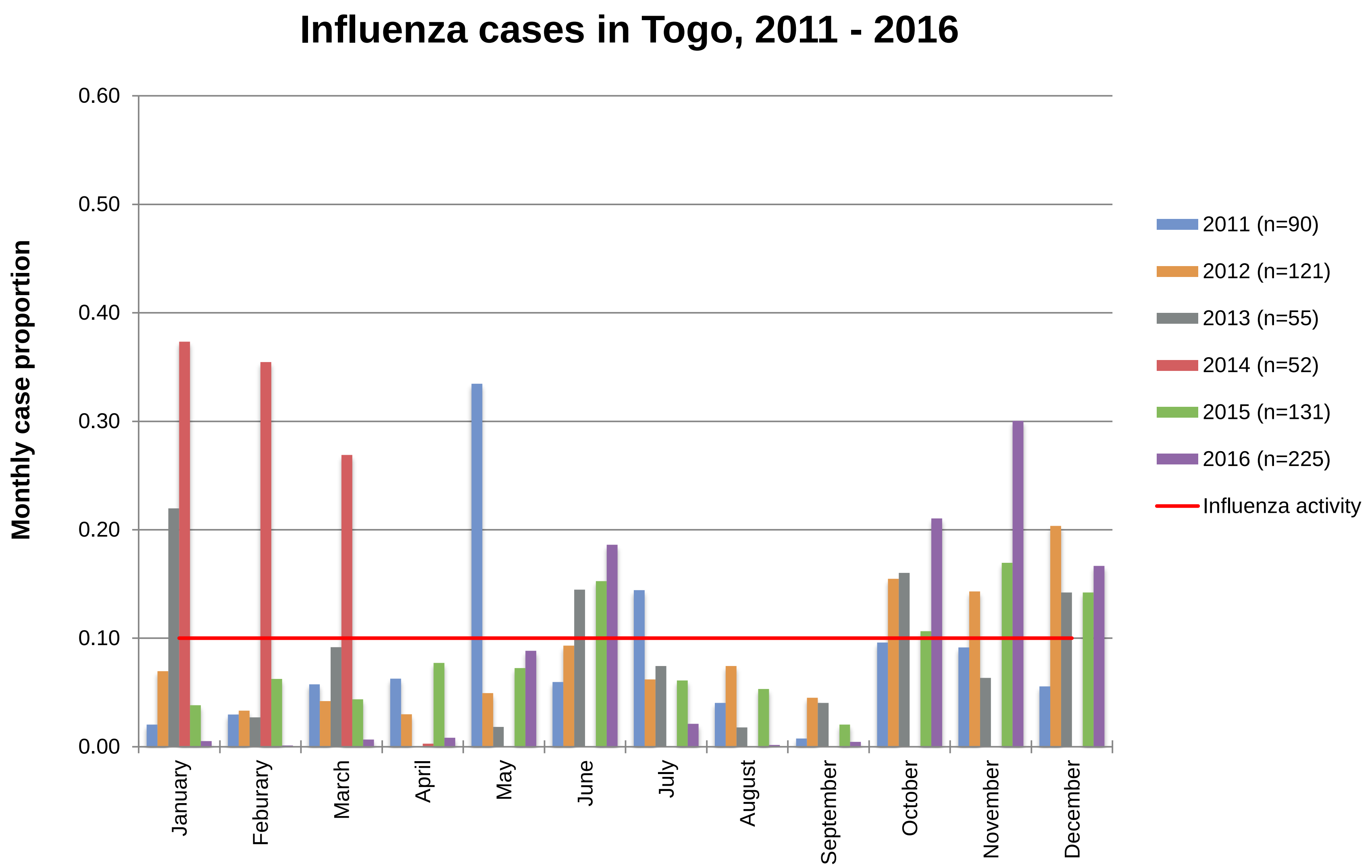

Influenza cases in Togo, 2011 - 2016
Monthly case proportion
0.60
0.50
0.40
0.30
0.20
0.10
0.00
January
Feburary
March
April
May
June
July
August
September
October
November
December
2011 (n=90)
2012 (n=121)
2013 (n=55)
2014 (n=52)
2015 (n=131)
2016 (n=225)
Influenza activity

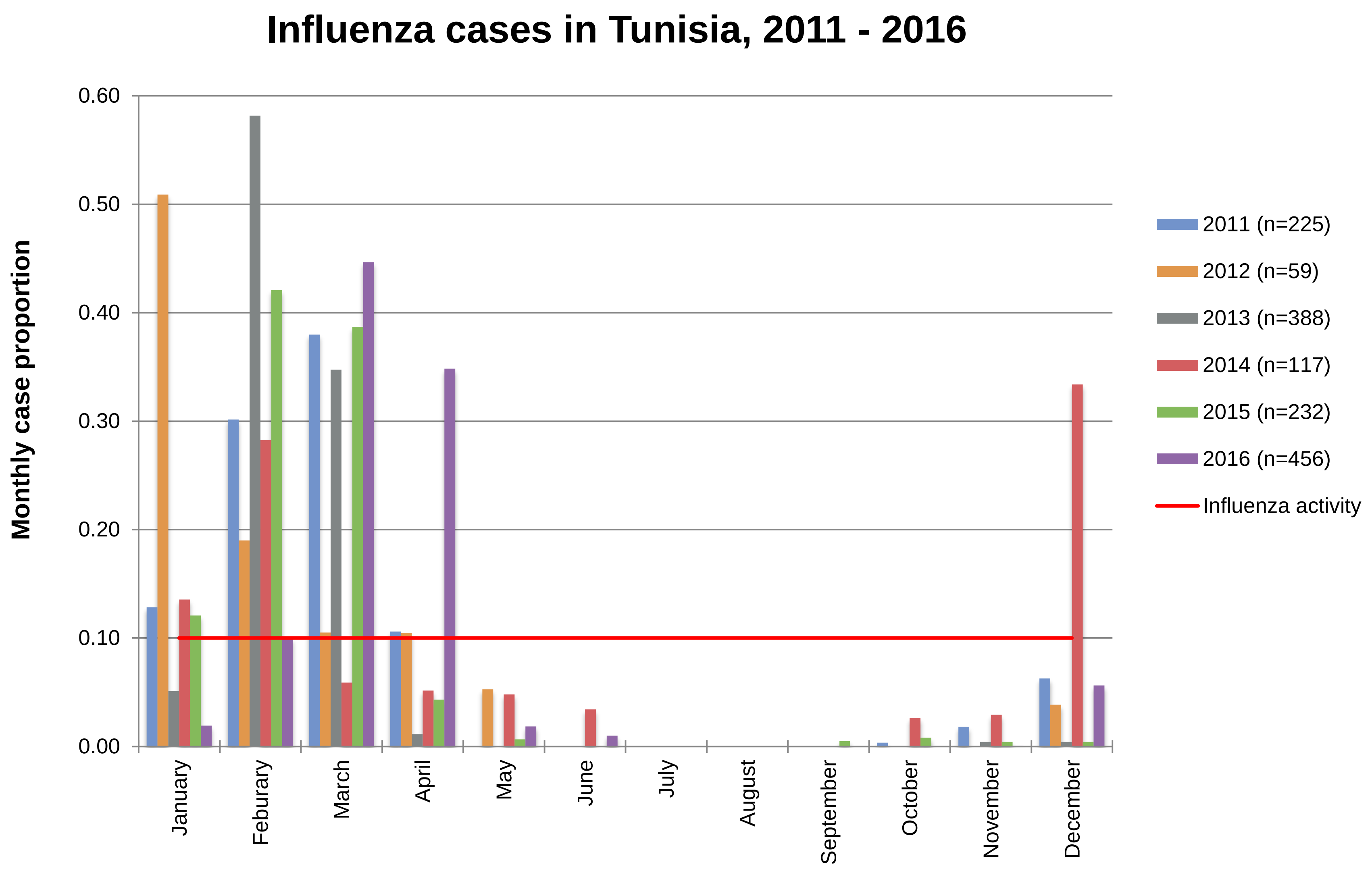
Influenza cases in Tunisia, 2011 - 2016
Monthly case proportion
0.60
0.50
0.40
0.30
0.20
0.10
0.00
January
Feburary
March
April
May
June
July
August
September
October
November
December
2011 (n=225)
2012 (n=59)
2013 (n=388)
2014 (n=117)
2015 (n=232)
2016 (n=456)
Influenza activity

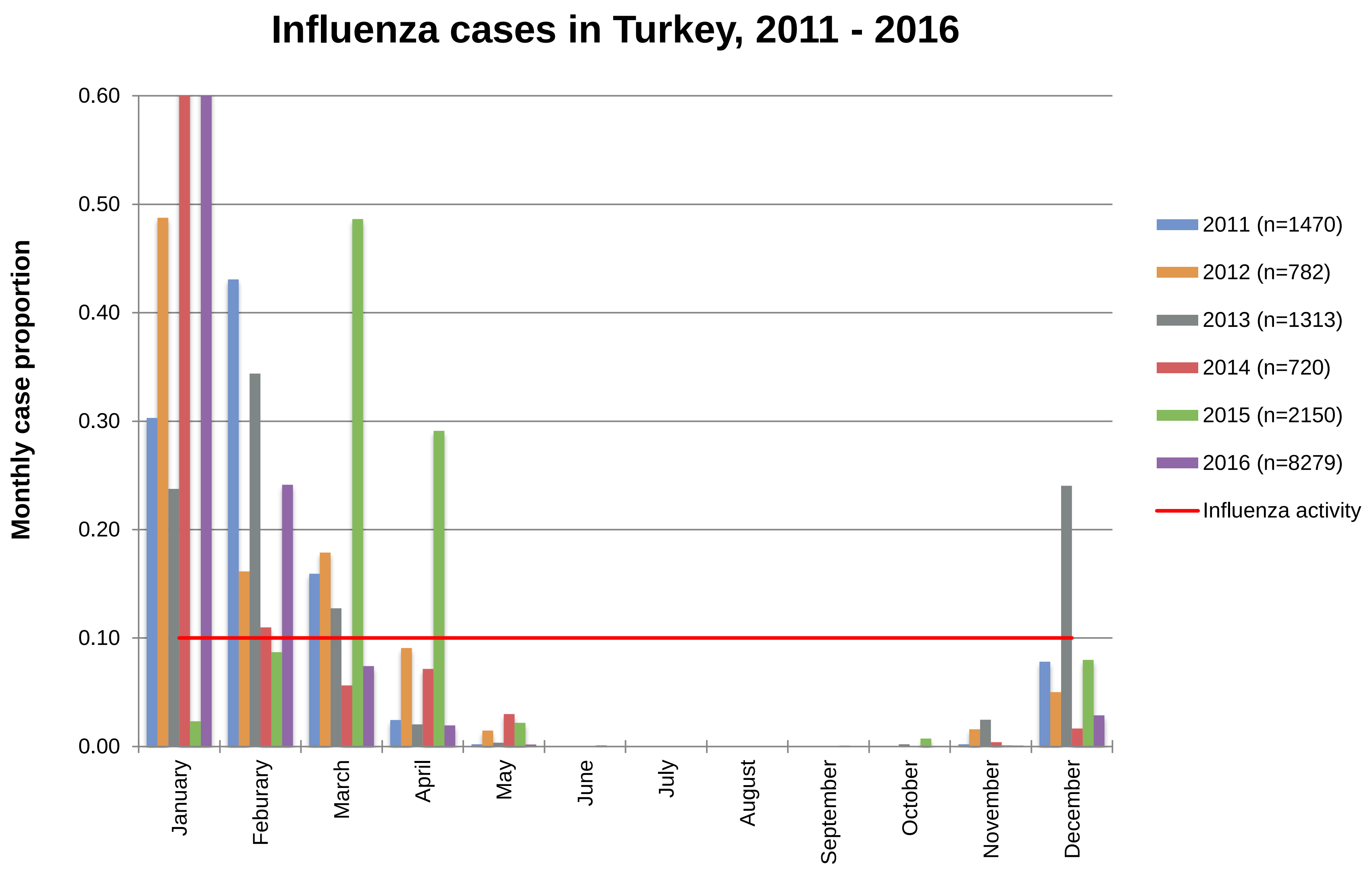

Influenza cases in Turkey, 2011 - 2016
Monthly case proportion
0.60
0.50
0.40
0.30
0.20
0.10
0.00
January
Feburary
March
April
May
June
July
August
September
October
November
December
2011 (n=1470)
2012 (n=782)
2013 (n=1313)
2014 (n=720)
2015 (n=2150)
2016 (n=8279)
Influenza activity

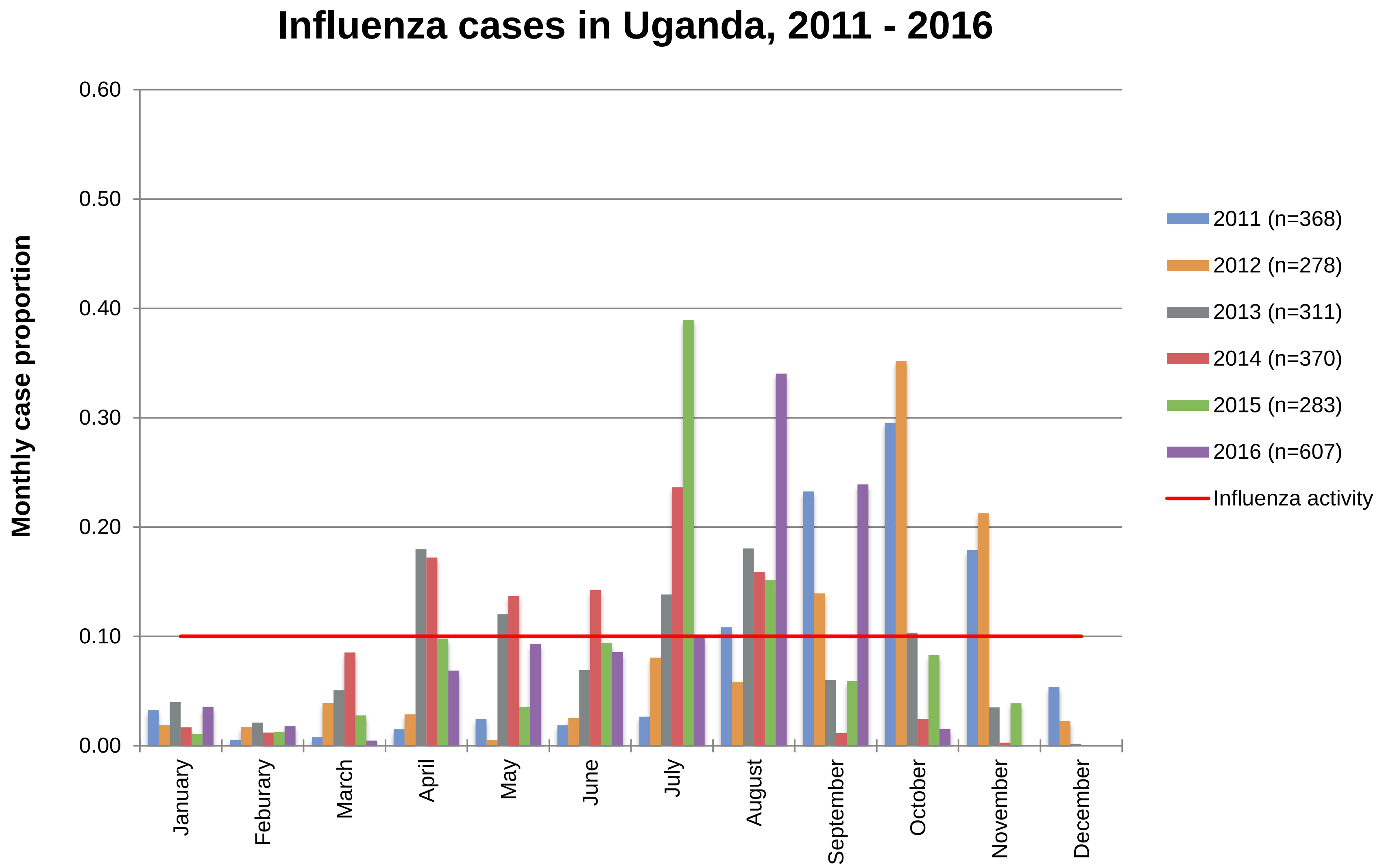

Influenza cases in Uganda, 2011 - 2016
Monthly case proportion
0.60
0.50
0.40
0.30
0.20
0.10
0.00
January
Feburary
March
April
May
June
July
August
September
October
November
December
2011 (n=368)
2012 (n=278)
2013 (n=311)
2014 (n=370)
2015 (n=283)
2016 (n=607)
Influenza activity

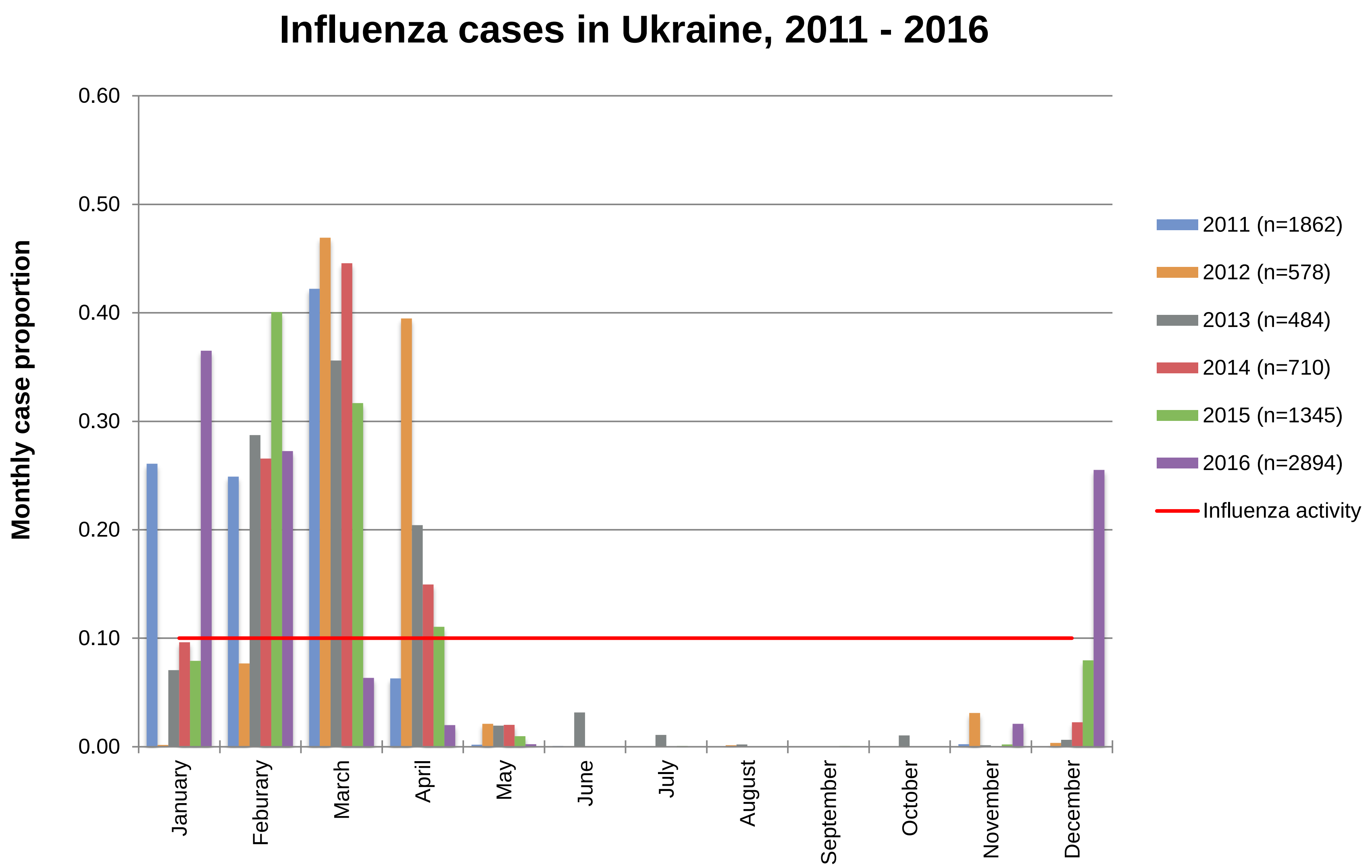
Influenza cases in Ukraine, 2011 - 2016
Monthly case proportion
0.60
0.50
0.40
0.30
0.20
0.10
0.00
January
Feburary
March
April
May
June
July
August
September
October
November
December
2011 (n=1862)
2012 (n=578)
2013 (n=484)
2014 (n=710)
2015 (n=1345)
2016 (n=2894)
Influenza activity

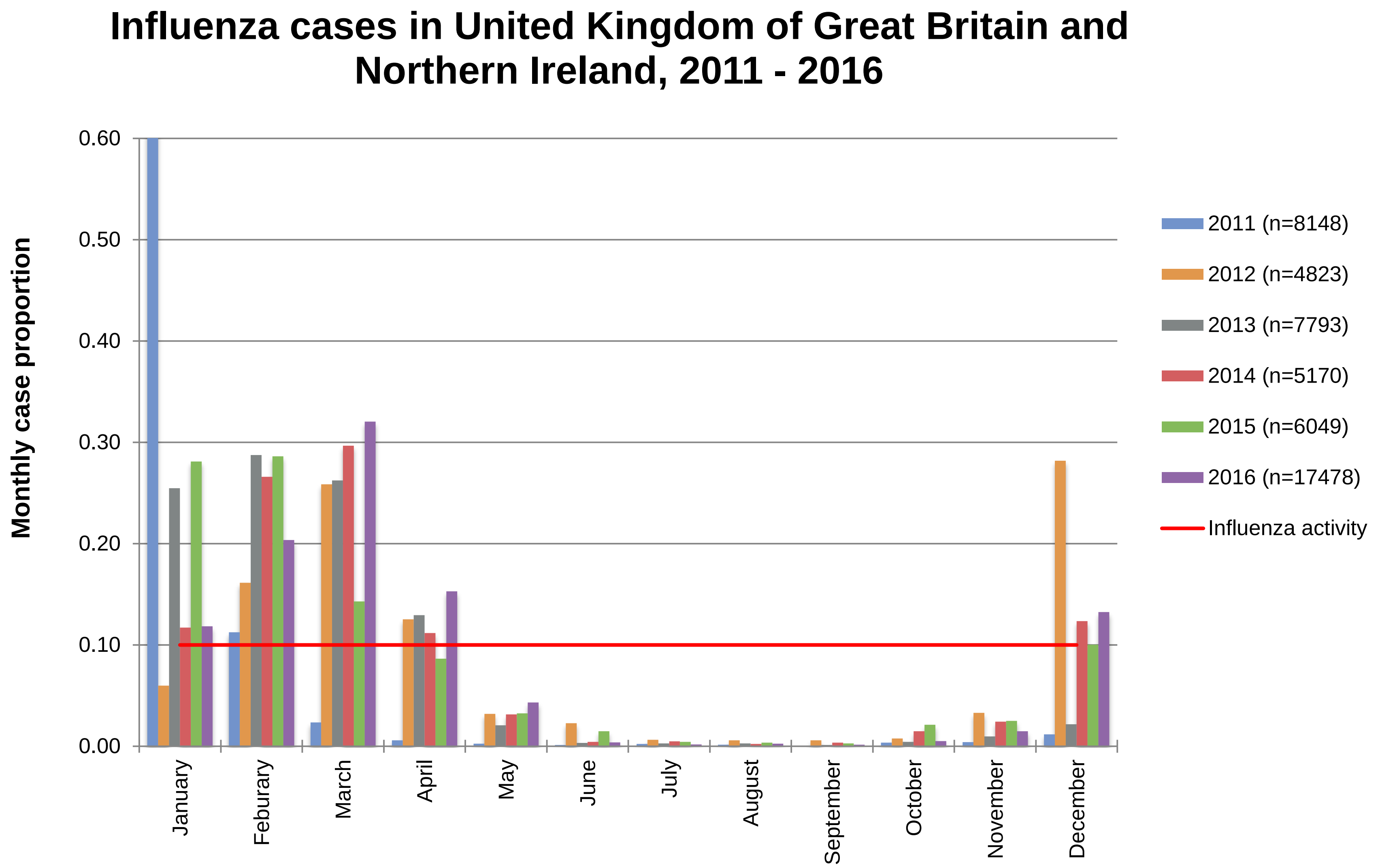
Influenza cases in United Kingdom of Great Britain and Northern Ireland, 2011 - 2016
Monthly case proportion
0.60
0.50
0.40
0.30
0.20
0.10
0.00
January
Feburary
March
April
May
June
July
August
September
October
November
December
2011 (n=8148)
2012 (n=4823)
2013 (n=7793)
2014 (n=5170)
2015 (n=6049)
2016 (n=17478)
Influenza activity

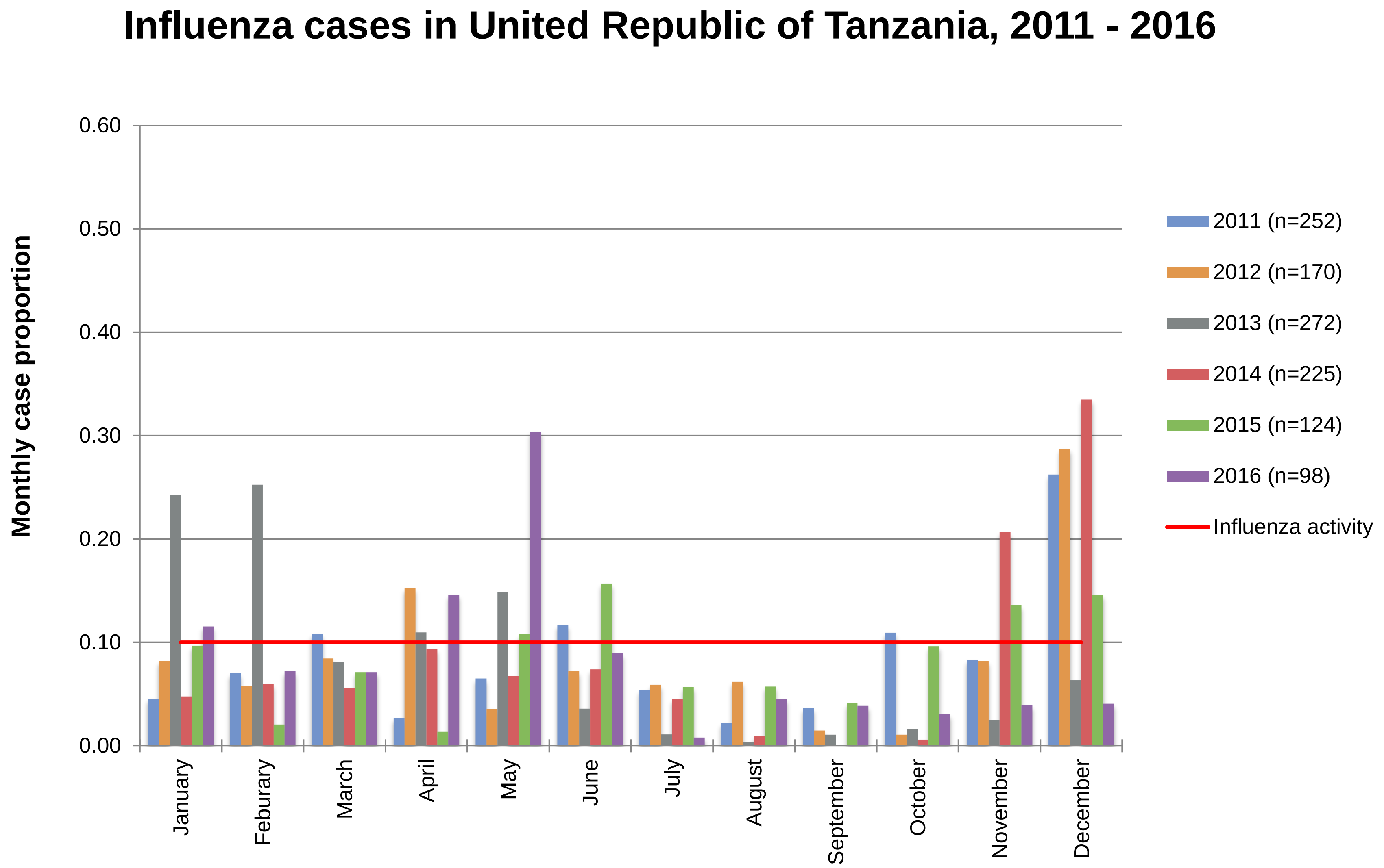
Influenza cases in United Republic of Tanzania, 2011 - 2016
Monthly case proportion
0.60
0.50
0.40
0.30
0.20
0.10
0.00
January
Feburary
March
April
May
June
July
August
September
October
November
December
2011 (n=252)
2012 (n=170)
2013 (n=272)
2014 (n=225)
2015 (n=124)
2016 (n=98)
Influenza activity

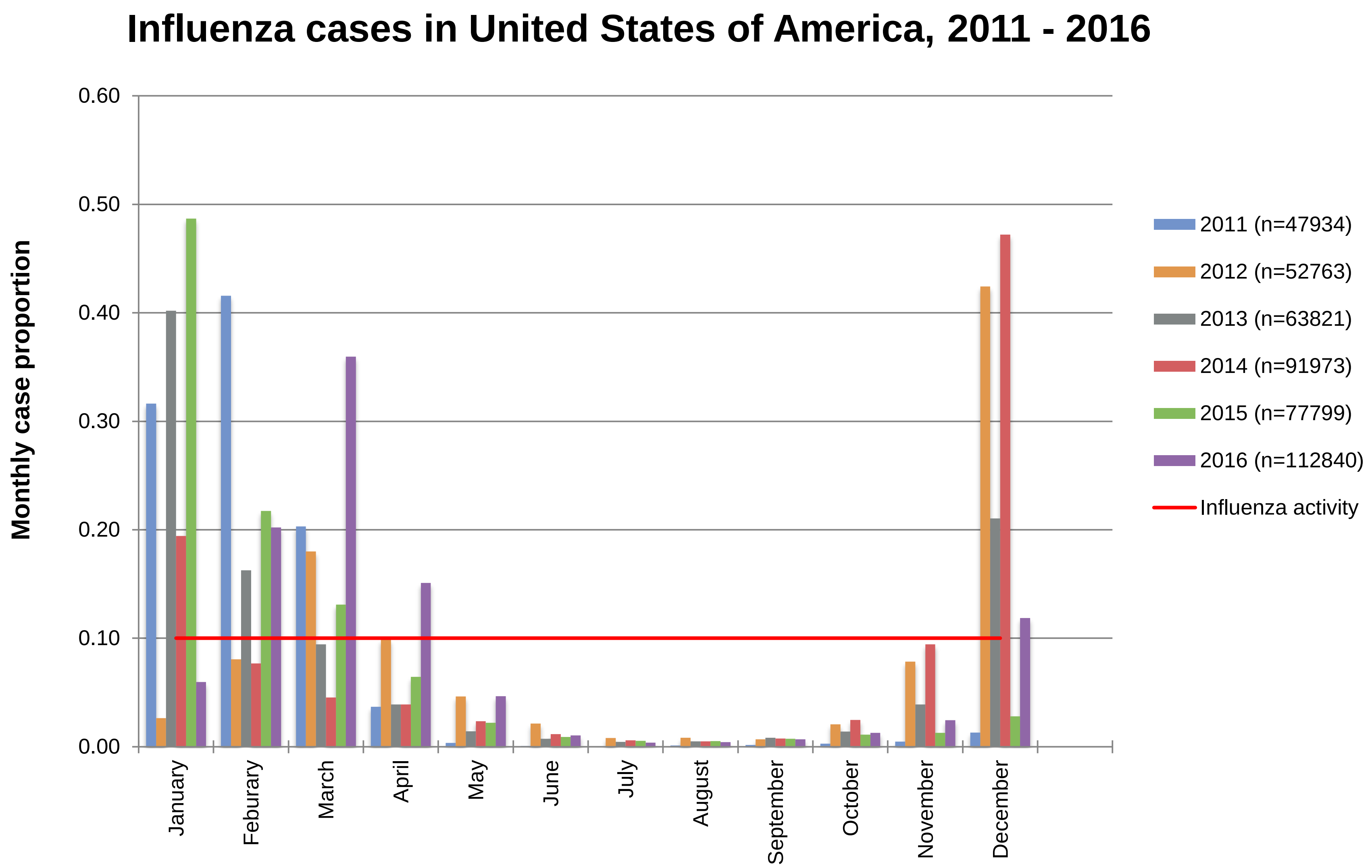
Influenza cases in United States of America, 2011 - 2016
Monthly case proportion
0.60
0.50
0.40
0.30
0.20
0.10
0.00
January
Feburary
March
April
May
June
July
August
September
October
November
December
2011 (n=47934)
2012 (n=52763)
2013 (n=63821)
2014 (n=91973)
2015 (n=77799)
2016 (n=112840)
Influenza activity

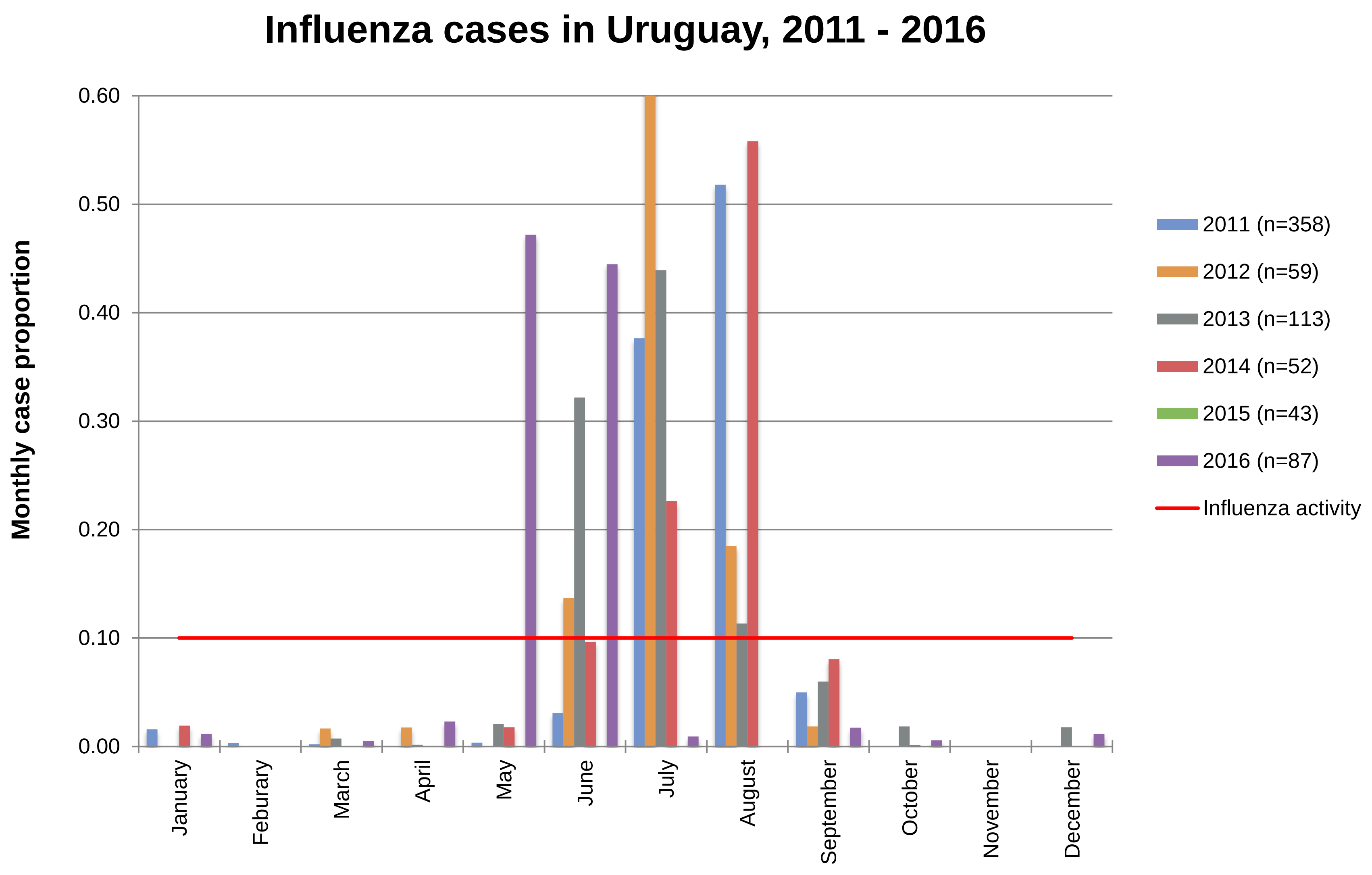

Influenza cases in Uruguay, 2011 - 2016
Monthly case proportion
0.60
0.50
0.40
0.30
0.20
0.10
0.00
January
Feburary
March
April
May
June
July
August
September
October
November
December
2011 (n=358)
2012 (n=59)
2013 (n=113)
2014 (n=52)
2015 (n=43)
2016 (n=87)
Influenza activity

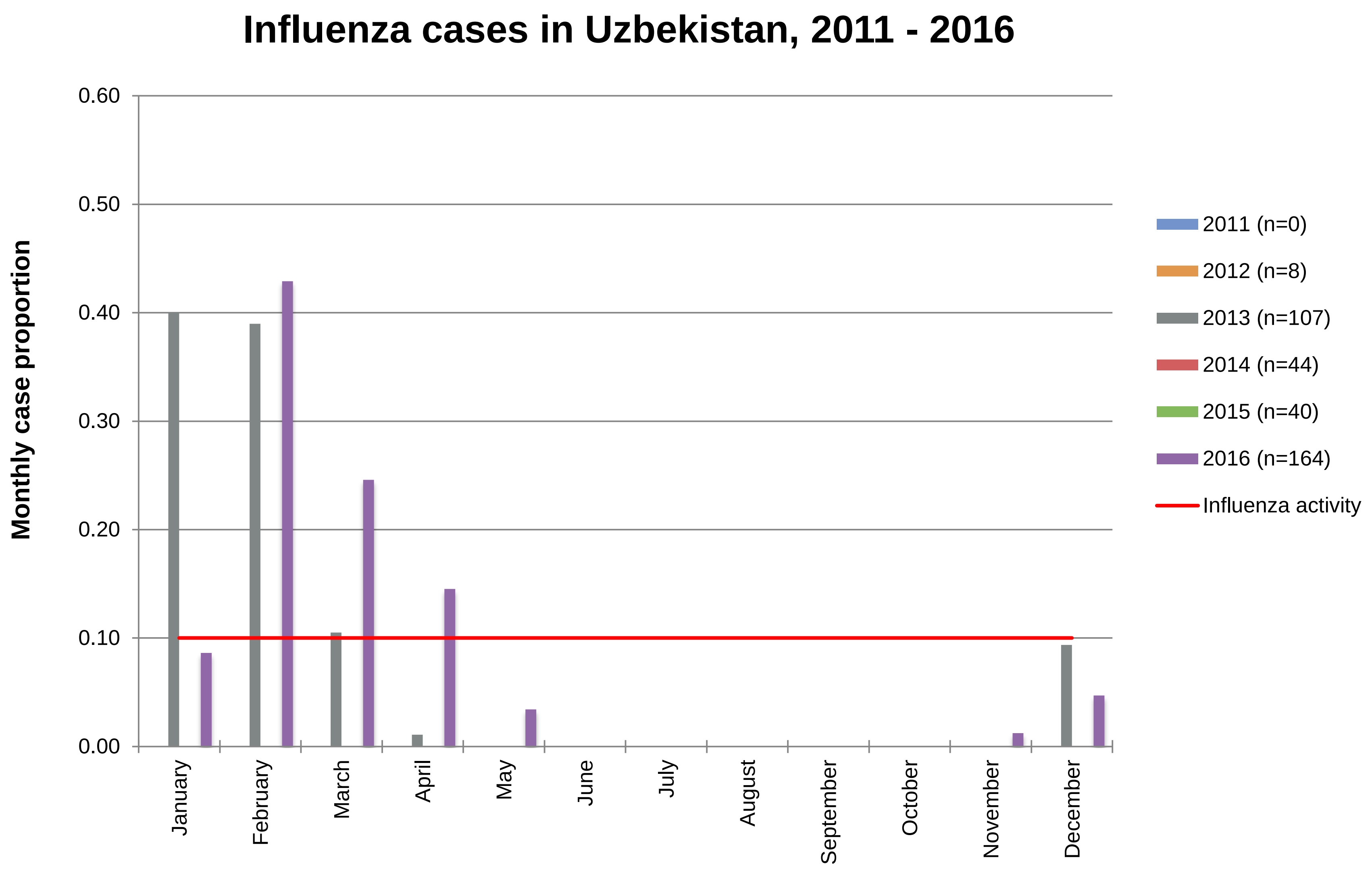
Influenza cases in Uzbekistan, 2011 - 2016
Monthly case proportion
0.60
0.50
0.40
0.30
0.20
0.10
0.00
January
February
March
April
May
June
July
August
September
October
November
December
2011 (n=0)
2012 (n=8)
2013 (n=107)
2014 (n=44)
2015 (n=40)
2016 (n=164)
Influenza activity

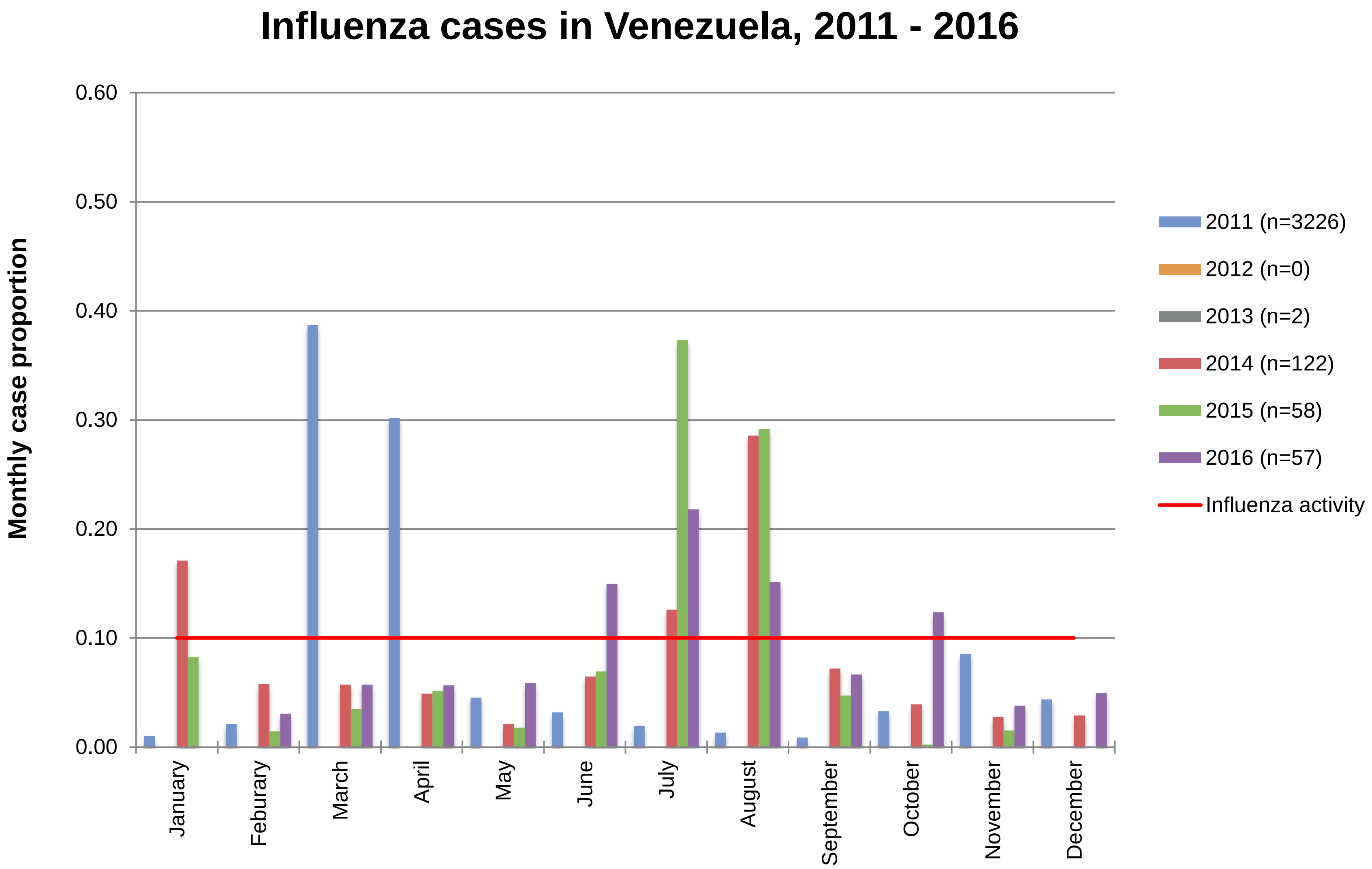

Influenza cases in Venezuela, 2011 - 2016
Monthly case proportion
0.60
0.50
0.40
0.30
0.20
0.10
0.00
January
Feburary
March
April
May
June
July
August
September
October
November
December
2011 (n=3226)
2012 (n=0)
2013 (n=2)
2014 (n=122)
2015 (n=58)
2016 (n=57)
Influenza activity

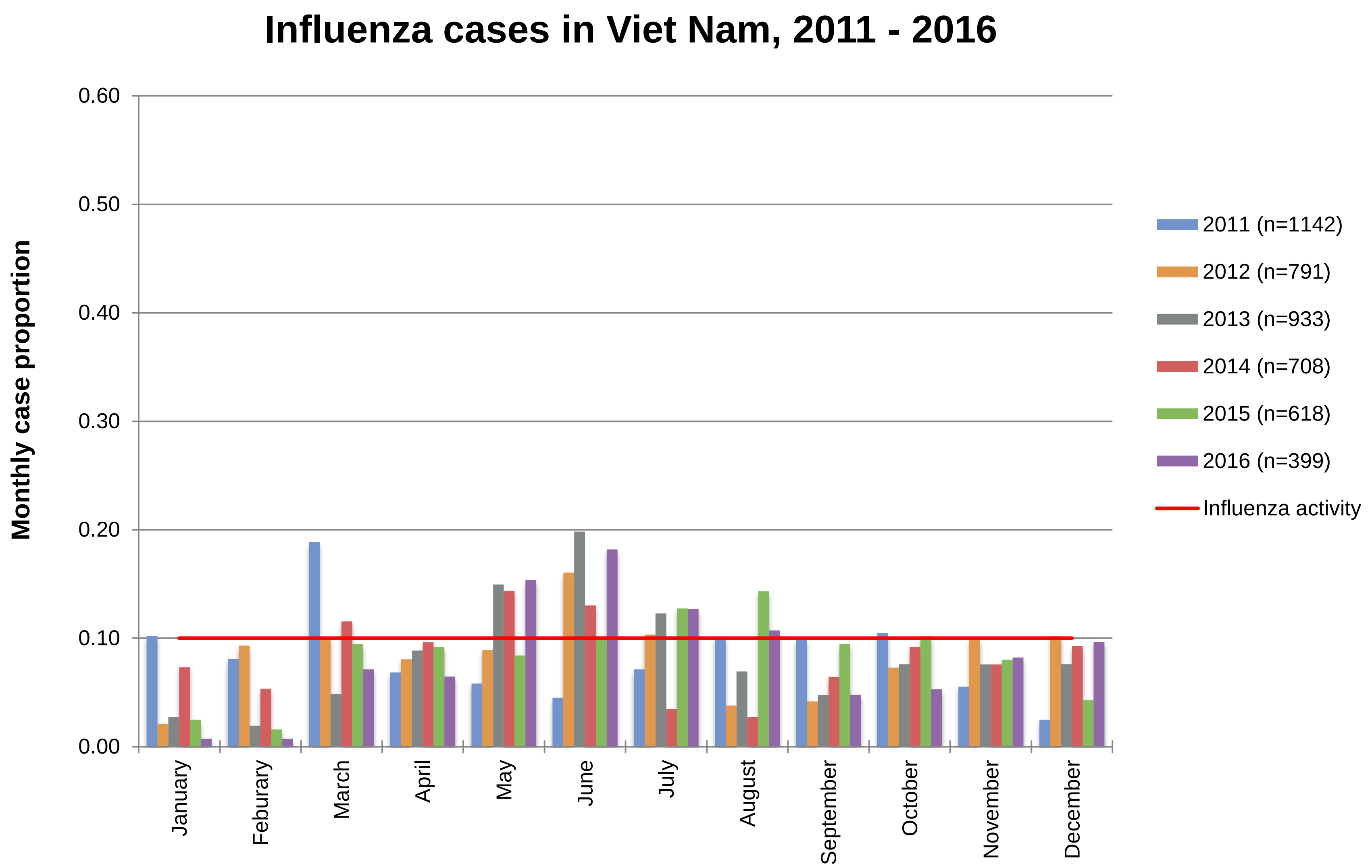

Influenza cases in Viet Nam, 2011 - 2016
Monthly case proportion
0.60
0.50
0.40
0.30
0.20
0.10
0.00
January
Feburary
March
April
May
June
July
August
September
October
November
December
2011 (n=1142)
2012 (n=791)
2013 (n=933)
2014 (n=708)
2015 (n=618)
2016 (n=399)
Influenza activity

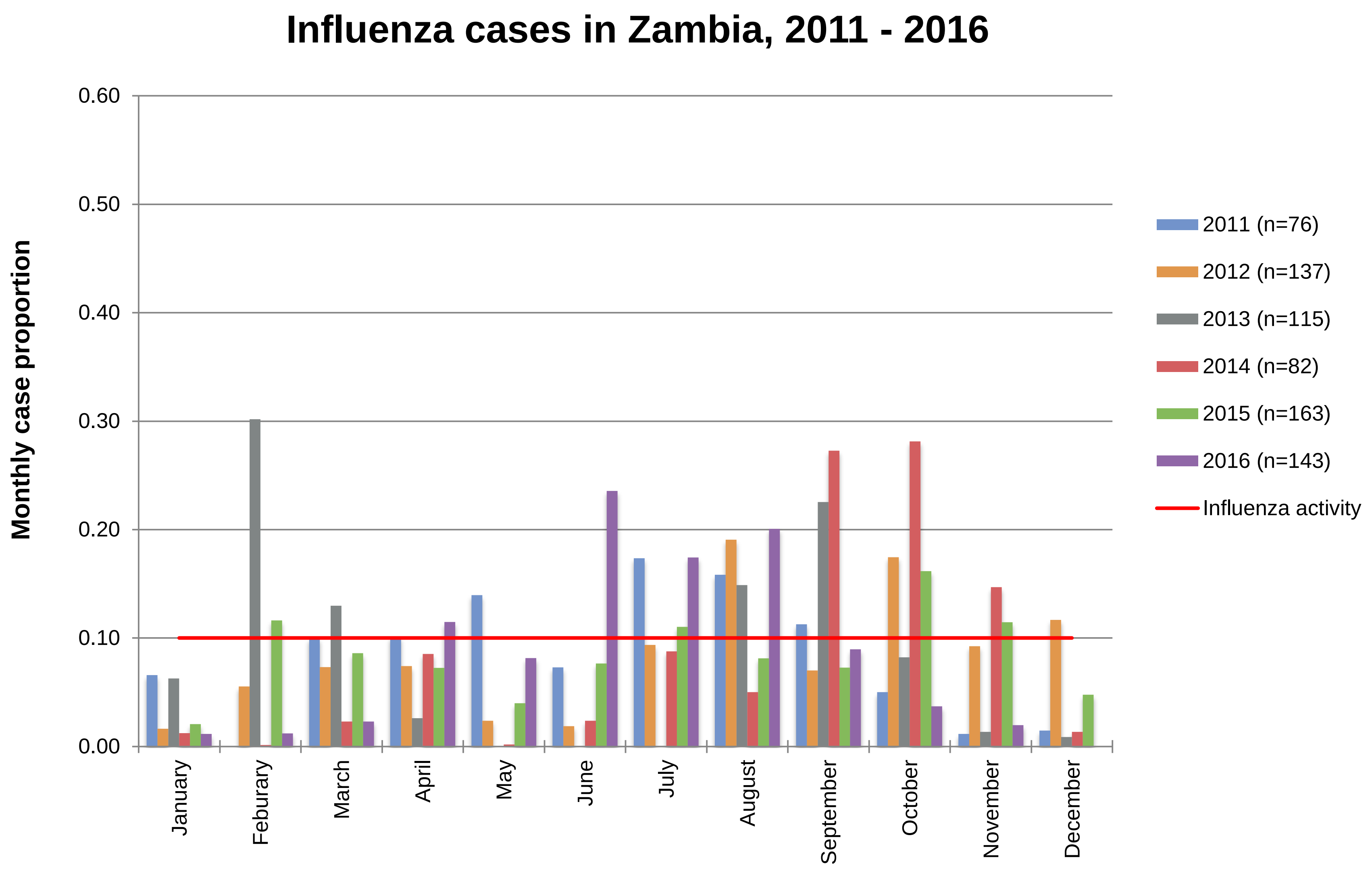
Influenza cases in Zambia, 2011 - 2016
Monthly case proportion
0.60
0.50
0.40
0.30
0.20
0.10
0.00
January
Feburary
March
April
May
June
July
August
September
October
November
December
2011 (n=76)
2012 (n=137)
2013 (n=115)
2014 (n=82)
2015 (n=163)
2016 (n=143)
Influenza activity
