# Supplementary material for: Global influenza seasonality to inform country-level vaccine programs: An analysis of WHO FluNet influenza surveillance data between 2011 and 2016
Source: PLoS One. 2018 Feb 21;13(2):e0193263. doi: 10.1371/journal.pone.0193263 (PMC5821378; doi:10.1371/journal.pone.0193263)
Supplement: S1 Table — (DOCX) [file pone.0193263.s002.docx]

|  |  |  |  |  |  |  |
| --- | --- | --- | --- | --- | --- | --- |
| **S1 Table.** Country-specific list of months with influenza activity^1^, number of months with influenza activity, and influenza patterns^2^ by World Health Organization region | | | | | |  |
| **African Region** | | **Months with Influenza Activity** | **Number of Months with Influenza Activity** | **Influenza Activity Pattern** | **Number of Years Reporting >49 Positive Influenza Cases** |  |
| Algeria | | December-March | 4 | 1 | 6 |  |
| Angola | | -- | -- | undefined | 0 |  |
| Burkina Faso | | January-March; July; October | 5 | year-round | 3 |  |
| Cameroon | | May-June; August-December | 7 | 1 | 6 |  |
| Cape Verde | | -- | -- | undefined | 0 |  |
| Central African Republic | | August-September | 2 | 1 | 3 |  |
| Chad | | -- | -- | undefined | 0 |  |
| Congo | | -- | -- | undefined | 0 |  |
| Côte d'Ivoire | | April-November | 8 | 1 | 6 |  |
| Democratic Republic of the Congo | | December-June | 7 | 1 | 6 |  |
| Ethiopia | | January-April; October-November | 6 | 1 | 4 |  |
| Ghana | | March-July; October-December | 8 | year-round | 6 |  |
| Guinea | | -- | -- | undefined | 0 |  |
| Kenya | | February-April; July-August; November | 6 | year-round | 5 |  |
| Madagascar | | January-March; May-August | 7 | 1 | 6 |  |
| Mali | | February-April; September-October | 5 | 2 | 4 |  |
| Mauritania | | -- | -- | undefined | 0 |  |
| Mauritius | | February-July | 6 | 1 | 5 |  |
| Mozambique | | -- | -- | undefined | 0 |  |
| Niger | | January-February | 2 | 1 | 2 |  |
| Nigeria | | January-March; May-November | 10 | year-round | 6 |  |
| Rwanda | | February-June | 5 | 1 | 5 |  |
| Senegal | | March; August-November | 5 | 2 | 6 |  |
| Sierra Leone | | August-October | 3 | 1 | 2 |  |
| South Africa | | May-September | 5 | 1 | 6 |  |
| Togo | | October-January; June | 5 | 2 | 6 |  |
| Uganda | | April-May; July-November | 7 | 1 | 6 |  |
| United Republic of Tanzania | | November-January; April-June | 6 | 2 | 6 |  |
| Zambia | | February; April; July-November | 7 | 2 | 6 |  |
|  | |  |  |  |  |  |
| **Eastern Mediterranean Region** | | **Months with Influenza Activity** | **Number of Months with Influenza Activity** | **Influenza Activity Pattern** | **Number of Years Reporting >49 Positive Influenza Cases** |  |
| Afghanistan | | -- | -- | undefined | 1 |  |
| Bahrain | | October-February | 5 | 1 | 5 |  |
| Egypt | | November-February | 5 | 1 | 6 |  |
| Iraq | | December-March | 4 | 1 | 4 |  |
| Islamic Republic of Iran | | December-February | 3 | 1 | 6 |  |
| Jordan | | December-May | 6 | 1 | 6 |  |
| Morocco | | November-March | 5 | 1 | 6 |  |
| Oman | | November-May | 7 | 1 | 6 |  |
| Pakistan | | December-March | 4 | 1 | 6 |  |
| Qatar | | November-January; March-April | 5 | 1 | 6 |  |
| Syrian Arab Republic | | -- | -- | undefined | 0 |  |
| Tunisia | | January-April | 4 | 1 | 6 |  |
|  | |  |  |  |  |  |
| **European Region** | | **Months with Influenza Activity** | **Number of Months with Influenza Activity** | **Influenza Activity Pattern** | **Number of Years Reporting >49 Positive Influenza Cases** |  |
| Albania | | January-March | 3 | 1 | 6 |  |
| Armenia | | -- | -- | undefined | 1 |  |
| Austria | | January-March | 3 | 1 | 6 |  |
| Azerbaijan | | -- | -- | undefined | 1 |  |
| Belarus | | January-April | 4 | 1 | 6 |  |
| Belgium | | January-March | 3 | 1 | 6 |  |
| Bosnia and Herzegovina | | January-March | 3 | 1 | 3 |  |
| Bulgaria | | January-March | 3 | 1 | 6 |  |
| Croatia | | January-April | 4 | 1 | 6 |  |
| Czech Republic | | -- | -- | undefined | 0 |  |
| Denmark | | January-March | 3 | 1 | 6 |  |
| Estonia | | January-April | 4 | 1 | 6 |  |
| Finland | | December-March | 4 | 1 | 6 |  |
| France | | December-March | 4 | 1 | 6 |  |
| Georgia | | January-April | 4 | 1 | 6 |  |
| Germany | | December-April | 5 | 1 | 6 |  |
| Greece | | January-March | 3 | 1 | 6 |  |
| Hungary | | January-April | 4 | 1 | 6 |  |
| Iceland | | January-April | 4 | 1 | 6 |  |
| Ireland | | December-April | 5 | 1 | 6 |  |
| Israel | | December-March | 4 | 1 | 6 |  |
| Italy | | December-March | 4 | 1 | 6 |  |
| Kazakhstan | | December-March | 4 | 1 | 6 |  |
| Kyrgyzstan | | December-February | 3 | 1 | 3 |  |
| Latvia | | January-April | 4 | 1 | 6 |  |
| Lithuania | | January-April | 4 | 1 | 6 |  |
| Luxembourg | | December-March | 4 | 1 | 6 |  |
| Malta | | December-March | 4 | 1 | 6 |  |
| Netherlands | | December-April | 5 | 1 | 6 |  |
| Norway | | December-March | 4 | 1 | 6 |  |
| Poland | | January-April | 4 | 1 | 6 |  |
| Portugal | | January-March | 3 | 1 | 6 |  |
| Republic of Moldova | | January-March | 3 | 1 | 5 |  |
| Romania | | January-April | 4 | 1 | 6 |  |
| Russian Federation | | January-April | 4 | 1 | 6 |  |
| Serbia | | January-April | 4 | 1 | 6 |  |
| Slovakia | | January-April | 4 | 1 | 6 |  |
| Slovenia | | January-April | 4 | 1 | 6 |  |
| Spain | | January-March | 3 | 1 | 6 |  |
| Sweden | | January-April | 4 | 1 | 6 |  |
| Switzerland | | December-March | 4 | 1 | 6 |  |
| Turkey | | January-March | 3 | 1 | 6 |  |
| Ukraine | | January-April | 4 | 1 | 6 |  |
| United Kingdom of Great Britain and Northern Ireland | | December-April | 5 | 1 | 6 |  |
| Uzbekistan | | February-March | 2 | 1 | 2 |  |
|  | |  |  |  |  |  |
| **Region of the Americas** | | **Months with Influenza Activity** | **Number of Months with Influenza Activity** | **Influenza Activity Pattern** | **Number of Years Reporting >49 Positive Influenza Cases** |  |
| Argentina | | June-September | 4 | 1 | 6 |  |
| Bolivia | | -- | -- | undefined | 0 |  |
| Brazil | | April-August | 5 | 1 | 6 |  |
| Canada | | December-April | 5 | 1 | 6 |  |
| Chile | | May-October | 6 | 1 | 6 |  |
| Colombia | | April-July | 5 | 1 | 6 |  |
| Costa Rica | | August-January | 6 | 1 | 6 |  |
| Cuba | | May-November | 7 | 1 | 6 |  |
| Dominican Republic | | April-July | 4 | 1 | 5 |  |
| Ecuador | | December-April; June-August | 8 | 1 | 6 |  |
| El Salvador | | May-July; December | 5 | 2 | 6 |  |
| Guatemala | | January-April | 4 | 1 | 6 |  |
| Honduras | | May; July; September-November | 5 | 1 | 5 |  |
| Jamaica | | February; October-November | 3 | 2 | 3 |  |
| Mexico | | December-March | 4 | 1 | 6 |  |
| Nicaragua | | August-December | 5 | 1 | 6 |  |
| Panama | | May-August; November | 5 | 2 | 5 |  |
| Paraguay | | June-August | 3 | 1 | 6 |  |
| Peru | | March; May-September | 6 | 1 | 6 |  |
| United States of America | | December-March | 4 | 1 | 6 |  |
| Uruguay | | June-August | 3 | 1 | 5 |  |
| Venezuela | | July-August | 2 | 1 | 4 |  |
|  | |  |  |  |  |  |
| **South-East Asia Region** | | **Months with Influenza Activity** | **Number of Months with Influenza Activity** | **Influenza Activity Pattern** | **Number of Years Reporting >49 Positive Influenza Cases** |  |
| Bangladesh | | April-September | 6 | 1 | 6 |  |
| Bhutan | | January; March-May; July-August | 6 | 1 | 6 |  |
| India | | February-April; November-December | 6 | 1 | 6 |  |
| Indonesia | | November-April | 6 | 1 | 6 |  |
| Nepal | | March-April; July-August | 4 | 2 | 5 |  |
| Sri Lanka | | November-January; March-July | 8 | year-round | 6 |  |
| Thailand | | February-March; July-November | 7 | 2 | 6 |  |
|  | |  |  |  |  |  |
| **Western Pacific Region** | | **Months with Influenza Activity** | **Number of Months with Influenza Activity** | **Influenza Activity Pattern** | **Number of Years Reporting >49 Positive Influenza Cases** |  |
| Australia | | July-October | 4 | 1 | 6 |  |
| Cambodia | | June-December | 7 | 1 | 6 |  |
| China | | December-March | 4 | 1 | 6 |  |
| Fiji | | -- | -- | undefined | 2 |  |
| Japan | | December-March | 4 | 1 | 6 |  |
| Republic of Korea | | December-April | 5 | 1 | 6 |  |
| Lao People's Democratic Republic | | September-February | 6 | 1 | 6 |  |
| Malaysia | | January-March; May-June | 5 | 1 | 4 |  |
| Mongolia | | December-April | 5 | 1 | 6 |  |
| New Zealand | | July-October | 4 | 1 | 6 |  |
| Philippines | | January-February; July-October | 6 | 2 | 6 |  |
| Singapore | | December-February; June-August | 6 | 2 | 6 |  |
| Viet Nam | | March; May-August; October | 6 | 1 | 6 |  |
|  | |  |  |  |  |  |
| **Non-member states** | | **Months with Influenza Activity** | **Number of Months with Influenza Activity** | **Influenza Activity Pattern** | **Number of Years Reporting >49 Positive Influenza Cases** |  |
| New Caledonia | | March-April; July-September | 5 | 2 | 6 |  |
| French Guiana | | February-June | 5 | 1 | 5 |  |
| Guadeloupe | | January-March | 3 | 1 | 4 |  |
| Martinique | | January-March | 3 | 1 | 3 |  |
| ^1^A month was considered to have influenza activity if it had 10% or more of total yearly cases of influenza for two or more years between 2011-2016. Countries that reported <50 influenza cases in a year were excluded for that year. Countries with <50 influenza cases reported in four or five years are noted as "undefined". No differentiation was made between countries that reported zero cases of influenza and reported no data. | | | | | |  |
| ^2^1 season: One to seven consecutive months of influenza activity; 2 seasons: Two sets of influenza activity separated by 2+ months of non-activity; year-round activity: Eight or more months of flu activity, or 3+ sets of influenza activity each separated by 2 months | | | | | |  |
|  | |  |  |  |  |  |
